# Applicability and Psychometric Properties of General Mental Health Assessment Tools in Autistic People: A Systematic Review

**DOI:** 10.1007/s10803-024-06324-3

**Published:** 2024-04-13

**Authors:** Marianne Berg Halvorsen, Arvid Nikolai Kildahl, Sabine Kaiser, Brynhildur Axelsdottir, Michael G. Aman, Sissel Berge Helverschou

**Affiliations:** 1 Department of Pediatric Rehabilitation, University Hospital of North Norway, P.O. Box 2, 9038 Tromsø, Norway; 2 NevSom Norwegian Centre of Expertise for Neurodevelopmental Disorders and Hypersomnias, Oslo University Hospital, Oslo, Norway; 3 Intellectual Disabilities/Autism, Regional Section Mental Health, Oslo University Hospital, Oslo, Norway; 4 Regional Centre for Child and Youth Mental Health and Child Welfare (RKBU North), UiT The Arctic University of Norway, Tromsø, Norway; 5 Regional Centre for Child and Adolescent Mental Health, Eastern and Southern Norway, Oslo, Norway; 6 Ohio State University, Columbus, OH USA; 7 Nisonger Center, University Center for Excellence in Developmental Disabilities, Ohio State University, Columbus, OH USA

**Keywords:** Assessment, Autism, Autism spectrum disorder, Mental disorders, Mental health, Psychometrics

## Abstract

In recent years, there has been a proliferation of instruments for assessing mental health (MH) among autistic people. This study aimed to review the psychometric properties of broadband instruments used to assess MH problems among autistic people. In accordance with the PRISMA guidelines (PROSPERO: CRD42022316571) we searched the APA PsycINFO via Ovid, Ovid MEDLINE, Ovid Embase and the Web of Science via Clarivate databases from 1980 to March 2022, with an updated search in January 2024, to identify very recent empirical studies. Independent reviewers evaluated the titles and abstracts of the retrieved records (*n* = 11,577) and full-text articles (*n* = 1000). Data were extracted from eligible studies, and the quality of the included papers was appraised. In all, 164empirical articles reporting on 35 instruments were included. The review showed variable evidence of reliability and validity of the various instruments. Among the instruments reported in more than one study, the Aberrant Behavior Checklist had consistently good or excellent psychometric evidence. The reliability and validity of other instruments, including: the Developmental Behavior Checklist, Emotion Dysregulation Inventory, Eyberg Child Behavior Inventory, Autism Spectrum Disorder-Comorbid for Children Scale, and Psychopathology in Autism Checklist, were less documented. There is a need for a greater evidence-base for MH assessment tools for autistic people.

**Supplementary Information:**

The online version contains supplementary material available at 10.1007/s10803-024-06324-3.

Autism spectrum disorder (autism) is a neurodevelopmental condition characterized by impaired reciprocal social interaction and communication, and by restricted and repetitive patterns of activities and interests (American Psychiatric Association [APA], 2013). Autistic people are a heterogeneous group, with considerable interindividual variation in autism symptoms and among co-occurring difficulties (APA, 2013; Lombardo et al., 2019). It is well documented that autistic people are at increased risk of developing mental health (MH) disorders (Hollocks et al., 2019; Lai et al., 2019). Co-occurring MH disorders, such as anxiety and depressive disorders, may affect up to 50% of autistic people (Howlin, 2021; Lord et al., 2020). Many of the MH conditions experienced by autistic people are treatable; therefore, early detection and diagnosis are important for improving the well-being of affected individuals and their families.

It can be challenging to assess MH disorders in autistic people (Halvorsen et al., 2022; Helverschou et al., 2011; Kildahl et al., 2023; Underwood et al., 2015). Symptom overlap, a lack of appropriate assessment tools and diagnostic criteria, atypical or idiosyncratic symptom manifestations, and bias among clinicians may result in diagnostic overshadowing (Jopp & Keys, 2001), where symptoms of a co-occurring MH disorder are misattributed to autism or a co-occurring intellectual disability (ID). Conversely, autistic people have described experiences in which their autism-related characteristics are misattributed to a cooccurring MH disorder (e.g., Au-Yeung et al., 2019). Co-occurring ID or limited verbal skills further complicate assessment in individuals with autism (Bakken et al., 2016; Shattuck et al., 2020).

Although an increased risk of co-occurring MH conditions has been widely acknowledged in *research*, this increased risk is not necessarily adequately addressed in clinical practice (Lord et al., 2020). One important challenge in addressing these issues in the clinic is the current lack of knowledge concerning the properties of standardized tools for assessing MH conditions in autistic people and the *dispersion of this knowledge* across national boundaries and languages (Halvorsen et al., 2022; in press; Lai et al., 2019). Indeed, very few instruments have been designed for the general assessment of MH conditions in autistic people (e.g., the Autism Comorbidity Interview Present and Lifetime Version; Leyfer et al., 2006; the Psychopathology in Autism Checklist; Helverschou et al., 2009). Accordingly, broad-band instruments that were not originally developed for autistic people are commonly used in the assessment of MH conditions (e.g., the Achenbach System of Empirically Based Assessment [ASEBA; Achenbach & Rescorla, 2001]; the Strengths and Difficulties Questionnaire [SDQ; Goodman, 1997]). In this review, we use the term “broad-band assessments” to refer to tools designed to capture multiple conditions or symptom clusters in autistic patients. However, current knowledge is limited regarding the applicability of these measures, including their sensitivity and specificity in autistic people.

MH assessment is recommended as an essential component of care for all autistic people (e.g., Lai et al., 2019; Lord et al., 2021); therefore, it is necessary to obtain more knowledge about the reliability and validity of instruments for assessing general MH in autistic people across the spectrum. Knowledge about the strengths and weaknesses of different instruments is important for making informed decisions about which instrument to use in the clinic. Such knowledge is also important for informing the research community about development needs in evidence-based assessment in this area. The use of general broad-band instruments is especially important, as these instruments facilitate differential diagnostic assessments in a more systematic way than single-disorder instruments. Moreover, while anxiety and depression are common in autistic people (Lai et al., 2019), the use of broad-band instruments may aid clinicians in systematically exploring symptoms of less common MH conditions to avoid overlooking them.

## Objective

The aim of the present systematic review was to provide an overview of broad-band instruments for assessing MH conditions in autistic people. Specifically, we aimed to determine the psychometric properties of broad-band instruments used to assess general MH conditions in autistic people. This approach holds utility for clinicians and researchers interested in assessing MH problems in this population. The psychometric properties examined for each instrument included reliability, validity and availability of normative data. Reliability was examined in terms of internal consistency (i.e., the extent to which items on a single scale are correlated with the same concept), test–retest reliability (i.e., the degree to which similar responses are obtained with the repeated administration of an instrument), and inter-rater reliability (i.e., the ability of independent raters to report similar phenomena on the same scale) (Terwee et al., 2007). We examined validity in terms of content validity (i.e., the degree to which an instrument’s item content reflects the constructs it is intended to measure), construct validity (i.e., the underlying factor structure/degree of overlap between an instrument and existing similar measures), criterion-related validity (e.g., the degree to which scores on an instrument relate to a clinical diagnosis), and normative data enabling the assessment of the severity of MH symptoms (Terwee et al., 2007).

## Methods

The protocol for this systematic review was registered in PROSPERO, an international register for systematic reviews with health-related outcomes (No. CRD42022316571). PRISMA-COSMIN for OMIs Guideline were used for the reporting process (Elsman et al., 2022). The PRISMA-COSMIN checklist is provided in Supplementary Appendix A.

### Search Strategy

In collaboration with the authors a medical librarian (BA) developed a peer-reviewed search strategy, including both subject headings and keyword terms for tools to assess general mental health in people with autism spectrum disorder (ASD). The APA PsycINFO via Ovid, Ovid Medline, Ovid EMBASE, and Web of Science via Clarivate databases were searched for articles published from 1980 until March 22, 2022 (a 42-year span). A subsequent search for each instrument was performed on January 11th, 2024. The search strategies are provided in Appendix B.

### Eligibility Criteria

The inclusion criteria were as follows: (a) at least 70% of the sample in the study was confirmed to have ASD by means of a clinical diagnosis of ASD, ADI-R/ADOS, parent report of a clinical ASD diagnosis, or placement in schools for autistic people; (b) in those instances where an autistic participant(s) had co-occurring ID, determination of ID occurred by means of a clinical diagnosis of ID, parent report of such a diagnosis, or by using a standardized intelligence scale/adaptive scale; (c) all age groups; (d) original data on psychometric outcomes for general MH measures published in a peer-reviewed journal; (e) published or available in English; (f) focused on the development, adaptation, or evaluation of an instrument for assessing MH. The inclusion criteria for MH conditions were derived from the International Statistical Classification of Disease and Related Health Problems, 10th Revision (World Health Organization, 2010). Eligible MH conditions and their key diagnostic symptoms were classified as follows: (1) F20–29: schizophrenia, schizotypal, and delusional disorders; (2) F30–39: mood disorders; (3) F40–48; neurotic, stress-related and somatoform disorders; and (4) F91–94 behavioral and emotional disorders.

The exclusion criteria were as follows: (a) disorders of adult personality and behavior (F60–69), organic mental disorders, substance use disorders, behavioral syndromes associated with physiological disturbances and physical factors, neurodevelopmental disorders (ID, attention-deficit/hyperactivity disorder (ADHD), ASD), and motor disorders (Tourette syndrome). The reason for excluding these conditions in this review was because we wanted to narrow the focus to common emotional and behavioral disorders and not focus on personality disorders or neurodevelopmental disorders per se. Such delimitation was necessary to make the literature search/data handling practically possible. (b) published before 1980, in accordance with Flynn et al. (2017); (c) gray literature (PhD dissertations, conference abstracts, book chapters); (d) instruments used for assessing MH limited to one MH condition (e.g., depression); (e) focused on evaluating psychotropic drugs or other interventions; (f) reporting only descriptive mean scores for ASD samples (e.g., genetic syndromes) with no other psychometric information; and (g) sample size *N* ≤ 20.

### Study Selection Process

After the initial database search, duplicates were removed by using EndNote and Covidence. All titles and abstracts were independently screened by at least two reviewers (MBH [screened all references], ANK, SK, SBH) in Covidence. After determining which studies were eligible for full-text assessment, the full-text review was independently conducted by two reviewers (MBH, SBH). An agreement rate of 86% was reached between the two reviewers, and disagreements were resolved via discussion. A list of the studies excluded at the full-text assessment stage is presented in Supplementary Appendix C.

### Data Extraction

The data were extracted into a table format consistent with (Halvorsen et al., 2022) by one reviewer (MBH), and checked for accuracy by a second reviewer (BA). The following data were extracted: study design, country, participant demographics (age and sex), clinical characteristics (i.e., ASD severity, ID severity, language level, co-occurring diagnosis), informant characteristics (i.e., parent/caregiver, teacher, health-care professional), and information about the data analyses/psychometric properties. Narrative synthesis was performed to summarize for all studies reporting on each instrument.

### Quality of Evidence

Using four items from the Quality Assessment of Diagnostic Accuracy Studies (Whiting et al., 2003, and later modified by Villalobos et al. (2022)), risk of bias was scored on a nine-item scale: (a) three items assessed sample selection bias (1. Were the participants representative of the participants who will receive the test in practice?; 2. Were the selection criteria clearly described?; and 3. Did the whole sample, or a random selection of the sample, receive verification of the autism diagnosis using a reference standard of diagnosis?), (b) four items assessed methodology (4. Was the execution of the scale under review described in sufficient detail to permit replication of the test?; 5. Were withdrawals from the study accounted for?; 6. Is it clearly stated where the sample was obtained?; and 7. Is it clearly specified when the sample was obtained?); and (c) two items assessed result bias (8. Are the statistical analyses fully described?; and 9. Are the limitations of the article specifically addressed?). Each of the nine items was scored as low risk (0) or high risk (1), such that higher scores reflected greater concern of bias. Two reviewers (ANK, SBH) developed examples and operationalized rating criteria to develop common practices (Supplementary Appendix D).

Then, 27 randomly chosen papers were assessed independently by the two reviewers, who reached an agreement rate of 92%. Due to a high degree of agreement in scoring, the remaining papers (*n* = 137) were then randomly divided between ANK and SBH. Disagreements or uncertainties in the scoring were resolved by discussion. For six papers, ANK and SBH had a potential conflict of interest; MBH independently scored the risk of bias for these papers.

### Psychometric Quality of Instruments for Assessing MH

We used the EFPA review model for the description and evaluation of psychological and educational tests (European Federation of Psychologists’ Association [EFPA], 2013), to evaluate the psychometric properties (i.e., reliability, validity, and normative data) of the instruments for assessing MH (Table 1), and consistent with Halvorsen, Helverschou et al. (2022). This model used a four-point scale (0 = not reported/not applicable; 1 = inadequate; 2 = adequate; 3 = excellent/good). We noted whether any investigation of construct validity was conducted via exploratory or confirmatory factor analysis (EFA; CFA), or by testing for invariance of structure and differential item functioning across groups (measurement invariance) was registered (0 = not reported/N/A; 1 = reported). The quality assessment was independently conducted by MBH and SK for 25 randomly chosen studies that reported psychometric properties. The interrater reliability of these assessments showed an excellent degree of agreement (*r* = 0.99) for the sum scores. Accordingly, the remaining articles (*n* = 139) were randomly distributed between MBH and SK. Disagreements were resolved by discussion. All studies pertaining to each individual measurement tool were then included in *the overall assessment of each instrument*, thus enabling the authors to determine the weight of evidence for each instrument across studies.

**Table 1:** Interpretation guidance from the EFPA review model (2013) to evaluate the psychometric quality of included instruments

	Range	Rating assigned
Sample size	Not reported/applicable *N* < 100 *N* = 100–200 *N* > 200	0 = not reported/applicable 1 = one inadequate study 2 = one adequate study 3 = large/more than one adequate study
Internal consistency: Cronbach’s alpha	Not reported/applicable < .70 = .70–.79 ≥ .80	0 = not reported/applicable 1 = inadequate 2 = adequate 3 = good/excellent
Test–retest/interrater: correlation coefficient	Not reported/applicable < .60 .60–.69 ≥ .70	0 = not reported/applicable 1 = inadequate 2 = adequate 3 = good/excellent
Convergent validity: correlation coefficient	Not reported/applicable < .55 .55–.64 ≥ .65	0 = not reported/applicable 1 = inadequate 2 = adequate 3 = good/excellent
Criterion-related Validity: correlation coefficient	Not reported/applicable < .20 .20-.34 ≥ .35	0 = not reported/applicable 1 = inadequate 2 = adequate 3 = good/excellent
Normative data: Sample size	Not reported N < 300 N = 300–399 N ≥ 400	0 = not reported/applicable 1 = inadequate 2 = adequate 3 = good/excellent
Normative data: Quality of information provided: ID (e.g., mild, moderate, severe, profound), age, and gender	Inferential statistics used to verify group differences and similarities	0 = not reported/applicable 1 = inadequate 2 = adequate information, with minimal analysis 3 = good/excellent descriptions and analyses of groups and differences, and discussion of relevant issues relating to use and interpretation

*Note.* ID = intellectual disability

## Results

### Literature Selection

**Fig. 1:**
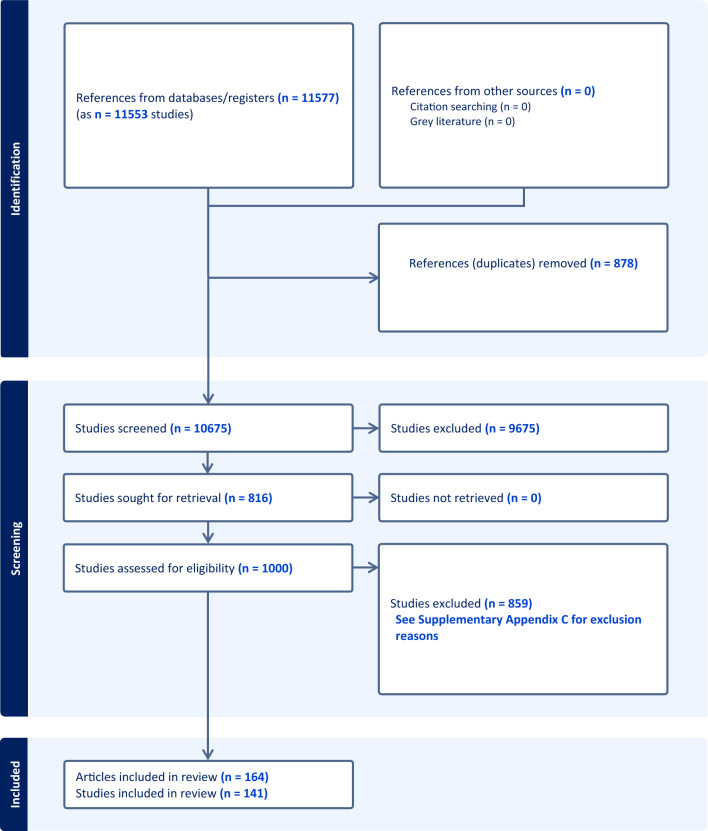
PRISMA flow diagram

The literature searches initially yielded 15,745 unique references. After removal of duplicates 11,577 studies were screened by titles and abstracts. Of these studies, 9675 were excluded. We assessed the full texts of 1000 articles. A total of 859 studies (860 articles) were excluded (see Appendix C for a listing of the excluded studies and the reasons for exclusion). Ultimately, 164 articles (141 studies) were included (see Appendix E for a listing of the included studies). Details of the study selection process and the reasons for exclusion are provided in Fig. 1 (PRISMA flow diagram).

### Assessment of Study Quality

In accordance with Villalobos et al. (2022), the studies were classified into four categories: very low risk of bias (0–2); low risk of bias (3–4); moderate risk of bias (5–6), and high risk of bias (7–9). The total scores for each study ranged from 0 to 7 (*M* = 3.31, *SD* = 1.28, *Mdn* = 3.00; see Appendix F for the risk of bias scores). All but one of the studies had a low (85%) or moderate risk of bias (14%). No articles were excluded from the review due to the risk of bias assessment.

### MH Assessment Instruments

Thirty-five unique instruments were examined across the 164 included papers (a summary of all studies and a description of the instruments are available in Appendixes G and H, respectively). The instruments used to assess multiple dimensions of MH problems in autistic people as follows:

- ***Toddlers.*** Seven instruments for use in toddlers (< 3 years of age). The most frequently reported were: the ASEBA– CBCL for individuals aged 1.5–5 years (Achenbach & Rescorla, 2001) (14 papers); the Baby and Infant Screen for Children with Autism Traits-Part 2 Comorbid Psychopathology (BISCUIT-Part2; Matson et al., 2009) (3 papers), and the Baby and Infant Screen for Children with Autism Traits-Part 3 Challenging Behaviors (BISCUIT-Part 3; Matson et al., 2009) (3 papers).
- ***Children and adolescents.*** Twenty-eight instruments for use in children and adolescents were identified, of which the seven most frequently reported instruments were: the ASEBA (31 papers), the Strengths and Difficulties Questionnaire (SDQ; Goodman, 1997) (24 papers); the Aberrant Behavior Checklist (ABC; Aman & Singh, 1986) (18 papers); the Nisonger CBRF Problem Behavior Section (NCBRF; Aman et al., 1996) (10 papers); the Autism Spectrum Disorder-Comorbid for Children (ASD-CC; Matson & González, 2007) (9 papers); the Developmental Behavior Checklist Primary Carer Version (DBC-P; Einfeld & Tonge, 1992) (6 papers), and the Emotion Dysregulation Inventory (EDI; Mazefsky et al., 2018) (6 papers).
- ***Adults*.** Ten instruments were identified for use in adults. The most frequently reported instruments were: the Psychopathology in Autism Checklist (PAC; Helverschou et al., 2009) (6 papers) and the Autism Spectrum Disorders-Comorbidity for Adults (ASD-CA; Matson & Boisjoli, 2008) (3 papers).

Among the instruments identified, there was a similar distribution between those developed or adapted for *autistic people and/or people with intellectual and developmental disabilities* (ASD/IDD-specific instruments *n* = 19: e.g., ABC; Autism Comorbidity Interview Present and Lifetime Version [ACI-PL; Leyfer et al., 2006]; DBC; EDI; NCBRF; PAC), and less specific instruments developed for the *general child or adult population* (conventional instruments *n* = 16: e.g., ASEBA; Child and Adolescent Symptom Inventory [CASI; Gadow & Sprafkin, 2010); Mini International Neuropsychiatric Interview [MINI; Sheehan et al., 1998, 2010]; Revised Child Anxiety and Depression Scale [RCADS; Chorpita et al., 2000]; Schedule for Affective Disorders and Schizophrenia for School-Age Children Present and Lifetime version [KSADS-PL; Kaufman et al., 1997]; SDQ) (see Appendix H for a description of the instruments). Overall, the instruments were based on descriptors of child/adult functioning by means of a descriptive-empirical approach (e.g., ABC; DBC; SDQ), diagnostic symptoms and/or criteria framework (e.g., ACI-PL; KSADS-PL; MINI), or a combination of the two approaches (e.g., ASD-CC; ASEBA; PAC).

The large majority of papers (*n* = 148) reported on informant-based measures (e.g., parent/caregiver reports), while 16 papers used self-report instruments, in which autistic participants’ intellectual functioning was in the normal range (self-report instruments reported in more than one study: ASEBA: Hurtig et al., 2009; Jepsen et al., 2012, Mazefsky et al., 2014; Pisula et al., 2017; RCADS: Khalfe et al., 2023; Sterling et al., 2015; SDQ: Findon et al., 2016; Khor et al., 2014) (see Appendix G for study characteristics). Moreover, all of the instruments used in the studies were originally developed in English, except for the Korean Comprehensive Scale for the Assessment of Challenging Behavior in Developmental Disorders (Kim et al., 2018) and the PAC (Norwegian; Helverschou et al., 2009).

Regarding the study population, there was a notable lack of population-based studies (only 7% of the investigations: ABC: Chua et al., 2023; Rohacek et al., 2023; ASEBA: La Buissonniere Ariza et al., 2022; DBC: Chandler et al., 2016; EDI: Day et al., 2024; KSADS-PL: Mattila et al., 2010; MINI Psychiatric Assessment Schedule for Adults with Developmental Disability: Buck et al., 2014; PAC: Bakken et al., 2010; SDQ: Deniz & Toseeb, 2023; Milosavljevic et al., 2016; Totsika et al., 2013), with the majority being convenience samples with participants recruited from clinics and other preexisting services. A significant proportion of the papers (26%; *n* = 43) relied on parent-reported clinical autism diagnoses or diagnoses derived from enrollment in schools for autistic people (see Appendix F for the risk of bias assessment). Furthermore, a small proportion of the papers included adult participants (12%; *n* = 20), and overall, there was a predominance of male participants. Intellectual functioning in the normal range (FSIQ ≥ 70) was observed in 20% (*n* = 33) of the papers (the ASEBA and SDQ were most frequently used in these samples; see Appendix G for study characteristics).

### Psychometric Quality of Instruments for Assessing MH

Based on the EFPA review model, all studies examining each individual measurement tool were included in the overall psychometric assessment of each instrument (i.e., reliability, validity, and normative data), thus enabling us to establish the weight of evidence for each instrument (see Table 2 and Appendix I for details regarding psychometric scores). The overall risk of bias scores (sample selection bias, methodological bias, and result bias) pertaining to each instrument are also shown in Table 2.

**Table 2:** Psychometric quality assessment of instruments

Assessment	Overall risk of bias score	Reliability			Validity				Norms	Overall psychometric quality assessment score
	*M (SD)*	Internal consistency	Test–retest	Inter-rater	Criterion	Content	Construct		Norms	*M (SD)*
							Factor structure	Convergent		
ABC	3.00 (1.19)	GE(13)	GE(1)	GE(1)	A(1)	NR	CFA/EFA(4)	GE(3)	GE(2)	8.07 (5.71)
ABI	6.00 (−)	IA(1)	A(1)	NR	NR	GE(1)	CFA(1)	GE(1)	NR	17.00 (−)
ACB	3.00 (0.00)	GE(3)	GE(1)	IA(1)	NR	GE(1)	CFA/EFA(2)	GE(2)	NR	14.66 (5.68)
ACI-PL	3.50 (0.70)	NR	NR	GE(1)	GE(1)	GE(1)	NR	A(1)	NR	10.00 (9.89)
ADIS-IV	2.50 (0.70)	NR	NR	GE(2)	NR	NR	NR	NR	NR	4.00 (0.00)
ASD-CA	5.00 (2.00)	GE(1) A(1)	A(1)	A(1)	IA(1)	NR	EFA(1)	NR	IA(1)	7.00 (5.19)
ASD-CC	4.62 (1.30)	GE(1) A(2)	A(1) IA(1)	IA(1)	GE(2)	NR	CFA/EFA(2)	GE(1) A(1) IA(1)	IA(1)	6.00 (3.29)
ASD-PBC	4.00 (−)	NR	NR	NR	NR	NR	NR	A(1)	NR	3.00 (−)
ASEBA	3.20 (1.22)	GE (15) A(4)	NR	IA(7)	A(2) IA(1)	NR	CFA/EFA/MI (9)	GE(1) A(1) IA(2)	NR	5.03 (2.40)
BASC-2	3.50 (2.12)	NR	NR	IA(2)	NR	NR	NR	IA(1)	NR	4.00 (2.82)
BISCUIT Part 2	4.50 (2.12)	GE(1)	NR	NR	NR	NR	EFA(1)	NR	A(1)	7.50 (3.53)
BISCUIT Part 3	4.00 (0.00)	A(1)	NR	NR	NR	NR	EFA(1)	NR	IA(1)	4.00 (1.00)
BPI	5.00 (0.81)	GE(3)	NR	NR	NR	NR	NR	GE(1)	NR	5.00 (0.81)
CASI	3.00 (0.00)	GE(1)	NR	A(1) IA(2)	NR	NR	CFA/EFA(3)	NR	NR	9.75 (7.04)
ChIPS	4.00 (−)	A(1)	NR	A(1)	NR	NR	NR	A(1)	NR	9.00 (−)
C-SHARP	4.50 (0.70)	GE(1) A(1)	NR	NR	NR	NR	MI(1)	NR	NR	6.50 (4.94)
CRS-R	3.00 (−)	NR	NR	IA(1)	NR	NR	NR	NR	NR	2.00 (−)
DASS-21	1.00 (−)	GE(1)	NR	NR	NR	NR	CFA(1)	GE(1)	NR	11.00 (−)
DBC	2.83 (1.47)	GE(5)	GE(1)	GE(1) IA(1)	NR	NR	NR	A(1)	NR	7.00 (3.09)
ECBI	2.33 (1.15)	GE(2)	NR	NR	NR	NR	EFA(2)	A(2)	NR	9.00 (4.35)
EDI	2.83 (0.75)	GE(2)	A(1)	NR	GE(1)	GE(1)	CFA/EFA(2)	GE(1) A(2) IA(1)	NR	7.00 (5.72)
HADS	6.00 (−)	GE(1)	NR	NR	NR	NR	EFA(1)	A(1)	NR	10.00 (−)
ITSEA	5.00 (−)	NR	NR	A(1)	NR	NR	NR	NR	NR	3.00 (−)
K-CSCB	3.00 (−)	GE(1)	IA(1)	NR	NR	NR	NR	GE(1)	NR	12.00 (−)
KSADS	2.33 (1.52)	NR	NR	GE(3)	NR	NR	NR	NR	NR	4.00 (0.00)
MCAS	3.00 (−)	GE(1)	NR	NR	GE(1)	GE(1)	CFA/EFA(1)	GE(1)	NR	22.00 (−)
MINI	2.33 (1.52)	NR	NR	GE(1)	A(1) IA(1)	NR	NR	A(1)	NR	4.67 (2.08)
MINI-PASS-ADD	1.00 (−)	NR	NR	NR	A(1)	NR	NR	NR	NR	4.00 (−)
Nisonger	3.11 (1.05)	GE(8)	NR	IA(1)	NR	NR	CFA/EFA(1)	NR	NR	6.33 (5.19)
OSCA-AB-P	3.00 (−)	NR	NR	GE(1)	NR	GE(1)	NR	IA(1)	NR	9.00 (−)
PAC	1.50 (0.57)	GE(1) A(1)	NR	A(2)	A(3)	NR	NR	IA(1)	NR	7.00 (2.94)
RCADS	3.66 (0.57)	GE(2) A(1)	GE(1)	IA(2)	NR	NR	NR	A(2) IA(1)	NR	12.33 (6.65)
SDQ	3.37 (1.17)	GE(7) A(10) IA(7)	NR	IA(2)	IA(1)	NR	NR	A(1)	NR	5.08 (3.74)
SIB-R	4.00 (−)	GE(1)	NR	A(1)	NR	NR	NR	IA(1)	NR	12.00 (−)
SSIS-RS	5.00 (−)	GE(1)	NR	NR	NR	NR	NR	NR	NR	4.00 (−)

*Note*. Overall *risk of bias score* = average risk of bias score for each instrument, in which *lower scores* indicate a *lower risk* of bias. Overall quality assessment score = average *psychometric quality score* for each instrument, in which *higher scores* indicate *better psychometric quality*. Numbers in parentheses indicate the number of studies that reported on a given psychometric property

Abbreviations: A = adequate; GE = good–excellent; IA = inadequate; NR = not reported; CFA = confirmatory factor analysis; EFA = exploratory factor analysis; MI = measurement invariance; ABC = Aberrant Behavior Checklist; ABI = Autism Behavior Inventory; ACB = Assessment of Concerning Behavior; ACI-PL = Autism Comorbidity Interview Present and Lifetime version; ADIS-IV C/P = Anxiety Disorders Interview Schedule for DSM-IV Child and Parent Version; ASD-CA = Autism Spectrum Disorders-Comorbidity for Adults; ASD-CC = Autism Spectrum Disorder-Comorbid for Children; ASD-PBC = Autism Spectrum Disorder-Problem Behavior for Children; ASEBA = Achenbach System of Empirically Based Assessment; BASC-2 = Behavior Assessment System for Children; BISCUIT Part 2 = Baby and Infant Screen for Children with Autism Traits – Comorbid Psychopathology; BISCUIT Part 3 = Baby and Infant Screen for Children with Autism Traits – Challenging Behaviors; BPI = Behavior Problem Checklist; CASI = Child and Adolescent Symptom Inventory; CBCL: Child Behavior Checklist; ChIPS = Children’s Interview for Psychiatric Syndromes; CRS-R = Conner’s Rating Scale-Revised; C-SHARP = Children’s Scale of Hostility and Aggression; DASS-21 = Depression, Anxiety, and Stress Scales; DBC = Developmental Behavior Checklist; ECBI = Eyberg Child Behavior Inventory; EDI = Emotion Dysregulation Inventory HADS = Hospital Anxiety and Depression Scale; K-CSCB = Korean Comprehensive Scale for the Assessment of Challenging Behavior in Developmental Disorders; KSADS = Schedule for Affective Disorders and Schizophrenia for School-age Children; MCAS = Mental Health Crisis Assessment Scale; MINI = Mini-International Neuropsychiatric Interview; MINI PAS-ADD = MINI Psychiatric Assessment Schedules for Adults with Developmental Disabilities; OSCA-ABP = Observation Schedule for Children with Autism-Anxiety, Behavior and Parenting; PAC = Psychopathology in Autism Checklist; RCADS = Revised children anxiety and depression scale; SIB-R = Scales of Independent Behavior-Revised; SSIS-RS = Social Skills Improvement System-Rating Scales

The ASEBA, SDQ and ABC were the most commonly used instruments in the reviewed studies (38, 26, and 18 papers, respectively). As shown in Table 2, the ABC was the only instrument with *all* aspects of reliability (internal consistency, test–retest, and interrater reliability) rated as good/excellent. Furthermore, its convergent validity was rated as good/excellent, and this finding was documented in multiple supporting studies. Moreover, in relation to the English language ABC factor structure, the two largest studies (Kaat et al., 2014: *N* = 1893; Norris et al., 2019: *N* = 470) recommended using the original factor structure in intellectually heterogeneous autism samples. Normative data were available for the ABC from two large studies (*N* ≥ 400) that reported information about its quality and analyzed differences in ID/FSIQ levels across sexes and ages (Kaat et al., 2014; Norris et al., 2019). The average risk of bias score was low across all studies that used the ABC.

In relation to the ASEBA and SDQ, the average risk of bias scores for these studies were low. For the ASEBA, only one aspect of reliability (internal consistency) was rated as good–excellent and was confirmed by supporting studies. There was inconsistent evidence across studies regarding the validity of this instrument. In relation to studies testing the CBCL factor structure using the English language version of the scale (N > 200), Medeiros et al. (2017) used data from an intellectually heterogeneous autism sample and found that the established CBCL factor structure (by means of CFAs) was the best fitting model for young children with autism (aged 1.5–5 years) but not for older children with autism (aged 6–18 years). Regarding differential item functioning, Schiltz and Magnus (2020) reported that only some CBCL items function differently for male and female autistic children aged 6–18 years (items flagged for sex-based differential functioning were on the Social Problems, Anxious/Depressed, Aggressive Behavior, and Thought Problems subscales). Finally, Dovgan et al. (2019) tested measurement invariance for the CBCL for autistic children aged 1–5 and 6–18 years with and without concurrent IDs. The findings showed that, among intellectually heterogeneous samples of autistic people, the item-level data from the CBCL should be used rather than broad-subscale-level data.

For the SDQ, only one aspect of reliability (internal consistency) was rated in the adequate-excellent range in the majority of studies, and there were no supporting studies that confirmed the validity of the instrument.

Other instruments with fewer studies documenting their reliability *and* validity (i.e., a lack of supporting studies) but with low average risk bias scores included the following: the Assessment of Concerning Behavior (ACB; Tarver et al., 2021) (two aspects of reliability rated as good/excellent, in addition to two aspects of validity, and factor structure reported); the DBC (two aspects of reliability exclusively rated as good/excellent, in addition to one aspect of validity rated as adequate); the EDI (two aspects of reliability rated as good/excellent and adequate, respectively, in addition to two aspects of validity rated exclusively as good/excellent, and factor structure reported); the Mental Health Crisis Assessment Scale (MCAS; Kalb et al., 2018) (one aspect of reliability, and all aspects of validity rated as good/excellent, in addition to factor structure reported); the Eyberg Child Behavior Inventory (ECBI; Eyberg & Pincus, 1999) (one aspect of reliability rated as good/excellent and one aspect of validity rated as adequate, and factor structure reported); the Children’s Interview for Psychiatric Syndromes (ChIPS; Weller et al., 1999) (two aspects of reliability rated as adequate, and one validity aspect); the ASD-CC (one aspect of reliability, and one aspect of validity assessed in the adequate-to-good/excellent rage documented by more than one study, in addition to factor structure), and the PAC (two aspects of reliability in the adequate-good/excellent range, and one aspect of validity rated as adequate).

In relation to the self-rating instruments, evidence of reliability (i.e., internal consistency) in the adequate to excellent range was reported for the ASEBA–Youth Self-Report (YSR) (Mazefsky et al., 2014), SDQ (Deniz & Toseeb, 2023; Khor et al., 2014), and RCADS (Khalfe et al., 2023; Sterling et al., 2015). Evidence of good or excellent internal consistency and convergent validity was observed for the Depression, Anxiety and Stress Scale (DASS-21; Lovibond & Lovibond, 1995; Park et al., 2020) and Hospital Anxiety and Depression Scale (HADS; Uljarevic et al., 2018; Zigmond & Snaith, 1983). However, the reported evidence for the self-report instruments was not confirmed by supporting studies (see Table 2).

In Table 2, the average psychometric quality (i.e., higher scores indicate better psychometric quality) is characterized, based on the sum score (maximum possible psychometric score = 40) for each instrument as they were scored during the psychometric quality assessment of the studies (see Appendix I for psychometric assessment scores). These indicated relatively large differences. Among the scores for instruments with five or more studies, the ABC had the highest average psychometric assessment score (*M* = 8.07), and the SDQ and the ASEBA had the lowest scores (*M* = 5.08 and *M* = 5.03, respectively).

Overall, the quality of the ASD/IDD-specific instruments (*M* = 8.58, *SD* = 4.80, *Mdn* = 7.00, *n* = 19) was somewhat better than that of the conventional instruments developed for the general child or adult populations (*M* = 6.80, *SD* = 3.49, *Mdn* = 5.05, *n* = 16). However, importantly the psychometric quality assessments varied within the ASD/IDD-instruments (range: 3 [poor] – 22 [good]), as did the sum scores within the conventional instruments (range: 2–12). The average risk of bias scores were low for studies using ASD/IDD-specific instruments (*M* = 3.54, *SD* = 1.22, *Mdn* = 3.11) and for conventional instruments (*M* = 3.39, *SD* = 1.24, *Mdn* = 3.29).

## Discussion

We identified many (*n* = 35) general broad-band instruments for assessing MH in autistic people. The instruments can be divided into two main groups. The first group comprises instruments developed for autistic people or people with IDDs (ASD/IDD-specific instruments: e.g., the ABC and DBC). The second group comprises conventional instruments developed for the general child or adult population (e.g., the ASEBA–CBCL and SDQ).

The main finding from the review was inconsistent evidence of the reliability and validity of the various instruments. Specifically, when examining the overall assessment of each instrument in detail, the conventional ASEBA-CBCL and SDQ were among the most widely used rating scales for assessing emotional and behavioral problems in children. The ASEBA-CBCL and SDQ were examined in most of the included papers (38 and 26 papers, respectively). However, the ASEBA-CBCL had only one aspect of reliability (internal consistency) in the good/excellent range as confirmed by supporting studies. Regarding, validity, there was conflicting evidence across studies. For the SDQ, evidence based on the majority of studies indicated internal consistency in the adequate/excellent range, and its validity was not confirmed by any supporting studies.

The ABC was the only instrument for which all aspects of reliability (internal consistency, test–retest, and interrater reliability) were rated as good/excellent; furthermore, its convergent validity, factor structure, and normative data were confirmed by supporting studies. Other instruments with less documentation of both reliability *and* validity, included the ACB, DBC, EDI, MCAS, ECBI, ChIPS, ASD-CC, and PAC; however, these instruments had fewer supporting studies. When we looked at the average psychometric quality scores, the ASD/IDD-specific instruments had overall a somewhat better score (*M* = 8.58, *SD* = 4.80) than did the instruments not designed or adapted for autistic people (*M* = 6.80, *SD* = 3.49). However, there was a relatively large range in the average psychometric scores for the different instruments, especially within the ASD/IDD-specific instruments (range: 3 [poor]–22 [good]). Due to the heterogeneity among autistic people in terms of clinical characteristics and levels of intellectual and verbal abilities, it is unlikely that a single tool will be able to detect MH problems across the entire autism population. Therefore, it is important to develop individualized, multimodal, and multi-informant approaches to assess MH conditions in autistic people (Halvorsen et al., in press; Lai et al., 2019; Underwood et al., 2015).

Based on this review, however, we can recommend several general-purpose scales with broad application that can be used among many autistic people. The ABC and DBC can work as screening measures for the initial assessment of MH problems in intellectually heterogeneous ASD samples in all age groups. These instruments were empirically developed based on descriptors of functioning in children and adults with ASD/IDDs (i.e., not diagnostic symptoms and criteria). The PAC, EDI and ACI-PL are potentially interesting instruments given that additional studies have evaluated their psychometric properties. The PAC is a screening instrument that seems to work among adults with autism and co-occurring IDs to distinguish between individuals with a MH disorder who need further assessment and those without a MH disorder (Helverschou et al., 2009, 2021).The EDI, which was designed to measure reactivity and dysphoria across the full range of verbal and cognitive abilities in autistic people, could aid in the differential diagnosis of conditions such as stress, anxiety, disruptive mood dysregulation disorder or intermittent explosive disorder; however, this needs to be investigated in future studies (Mazursky et al., 2018). The ACI-PL is an adaptation of the KSADS-PL in which the ADHD, depression and OCD modules have been examined among autistic school-aged children with intellectual functioning in the normal to mild ID range. However, the ACI-PL is demanding because the interviewer must be competent in distinguishing between autism symptoms and MH symptoms. There has, however, been no published psychometric data on the instrument in a decade (Leyfer et al., 2006; Mazefsky et al., 2012). The use of the ASEBA–CBCL is possible in autistic children with intellectual and verbal abilities in the normal range. If applied among intellectually heterogeneous ASD samples, the item-level data of the CBCL rather than the subscale-level data, should be used (Dovgan et al., 2019).

There is a debate in the literature regarding the use of measures originally developed for neurotypical populations and then applied with neurodiverse populations (e.g., Hanlon et al., 2022; Mandy, 2022). Some feel that this underserves the autistic community, and downplays the complex presentation of MH symptoms in individuals who are neurodiverse, which may lead to more misdiagnosis of MH symptoms (Halvorsen et al., in press; Hanlon et al., 2022). The evidence from this present review, indicates that primary use of instruments developed for ASD/IDD people (e.g., the ABC, DBC, EDI, and PAC) should be given priority in initial MH assessments. The ABC, DBC, and PAC include items assessing important MH domains, such as self-injurious behavior and psychotic symptoms; moreover, the DBC and PAC include subscales assessing anxiety and depressive symptoms. However, as ASD/IDD tools do not always have clear implications for diagnosis and are designed primarily for screening tools, they often need to be supplemented with conventional assessment tools in more comprehensive evaluations. As the use of multiple informants, including autistic people themselves when possible, is advised in the assessment of MH, the present review indicates that the self-report instruments DASS-21, HADS, ASEBA–YSR, SDQ, and RCADS can potentially be used in autistic adolescents and adults with intellectual and verbal abilities in the borderline and normal range. However, additional studies documenting the applicability of these instruments are needed.

This review highlights that the use of whole-population samples has been uncommon, with most studies relying on intellectually heterogeneous clinical or convenience samples predominated by boys. This approach may be appropriate for diagnostic interviews but constitutes a limitation for measures that are intended for broader use (e.g., screening tools). Clinical samples are likely to have more frequent and severe symptoms, and there may be sex differences in symptom manifestations associated with referral for clinical assessment. For instance, relying exclusively on clinical samples is associated with the risk of overemphasizing externalizing symptoms and underrecognizing internalizing symptoms, particularly for individuals with co-occurring IDs. Moreover, few studies have examined measurement properties of the instruments in adult samples (only 12%) or for self-report instruments (only 10%).

Our findings should be interpreted in the context of the strengths and limitations of our review itself. To our knowledge, this is the only recent review to examine the psychometric properties of broadband tools for assessing general MH problems in autistic people. Although the search strategy was broad and many potential studies were identified, there is always a risk of missing relevant studies. Moreover, study selection and full-text review were conducted independently by two reviewers. Parts of the quality/risk of bias assessments were also performed by two independent reviewers, thus ensuring adequate reliability for these assessments. We did not include a description of autism severity for each sample, as this information was not consistently reported across the papers. Additionally, in 26% of the studies autism diagnoses were not confirmed by a standardized assessment tool. However, these papers did rely on parent reports of autism diagnosis or diagnosis derived from enrollment in schools for autistic people.

## Conclusion

This review contributed to the field of MH assessment among autistic people by examining the psychometric properties of various tools used to assess general MH problems. Overall, we found variable evidence regarding the reliability and validity of the various instruments. Additionally, little research has examined the applicability of these instruments among adults and/or self-reports. Future research should focus on the development of evidence-based tools to assess MH in autistic people, as well as further development and improvement of existing measures in appropriate samples.

## Supplementary Information

Below is the link to the electronic supplementary material.

Supplementary file1 (DOCX 72 KB)

Supplementary file2 (DOCX 50 KB)

Supplementary file3 (DOCX 132 KB)

Supplementary file4 (DOCX 16 KB)

Supplementary file5 (DOCX 43 KB)

Supplementary file6 (DOCX 47 KB)

Supplementary file7 (DOCX 150 KB)

Supplementary file8 (DOCX 39 KB)

Supplementary file9 (DOCX 126 KB)
