# Supplementary material for: Applicability and Psychometric Properties of General Mental Health Assessment Tools in Autistic People: A Systematic Review
Source: J Autism Dev Disord. 2024 Apr 13;55(5):1713–26. doi: 10.1007/s10803-024-06324-3 (PMC12021962; doi:10.1007/s10803-024-06324-3)
Supplement: Supplementary file 1 — Supplementary file1 (DOCX 72 KB) [file 10803_2024_6324_MOESM1_ESM.docx]

| **Section and Topic** | **Item #** | **Checklist item** | **Location where item**  **is reported** |
| --- | --- | --- | --- |
| **TITLE** | | | |
| Title | 1 | Identify the report as a systematic review and include as applicable the following (in any order): outcome of interest, population of interest, name/type of OMIs of interest, and measurement properties of interest. | In the Title |
| **ABSTRACT** | | | |
| Abstract | 2 | See the PRISMA-COSMIN for Outcome Measurement Instruments Abstracts checklist. | Done |
| **INTRODUCTION** | | | |
| Rationale | 3 | Describe the rationale for the review in the context of existing knowledge. | Introduction p. 3-4 |
| Objectives | 4 | Provide an explicit statement of the objective(s) or question(s) the review addresses and include as applicable the following (in any order): outcome of interest, population of interest, name/type of OMIs of interest, and measurement properties of interest. | p. 4 |
| **METHODS** | | | |
| Followed guidelines | PC2 | Specify, with citations, the methodology and/or guidelines used to conduct the systematic review. | p. 5 |
| Eligibility criteria | 5 | Specify the inclusion and exclusion criteria for the review. | p. 5-7 |
| Information sources | 6 | Specify all databases, registers, websites, organisations, reference lists and other sources searched or consulted to identify studies. Specify the date when each source was last searched or consulted. | p. 5 |
| Search strategy | 7 | Present the full search strategies for all databases, registers and websites, including any filters and limits used. | Appendix B |
| Selection process | 8 | Specify the methods used to decide whether a study met the inclusion criteria of the review, including how many reviewers screened each record and each report retrieved, whether they worked independently, and if applicable, details of automation tools used in the process. | p. 7 |
| Data collection process | 9 | Specify the methods used to collect data from reports, including how many reviewers collected data from each report, whether they worked independently, any processes for obtaining or confirming data from study investigators, and if applicable, details of automation tools used in the process. | p. 7 |
| Data items | 10b | List and define which data were extracted (e.g. characteristics of study populations and OMIs, results of measurement properties, and aspects of feasibility and interpretability). Describe any assumptions made about any missing or unclear information. | p. 7 |
| Study risk of bias assessment | 11 | Specify the methods used to assess risk of bias in the included studies, including details of the tool(s) used, how many reviewers assessed each study and whether they worked independently, and if applicable, details of automation tools used in the process. | p.7-8 and Appendix D |
| Measurement properties | PC3 | Specify the methods used to rate the results of a measurement property for each individual study and for the summarized or pooled results. Specify how many reviewers rated each study and whether they worked independently. | p. 8-9 |
| Synthesis methods | 13a | Describe the processes used to decide which studies were eligible for each synthesis. | N/A |
|  | 13d | Describe any methods used to synthesize results and provide a rationale for the choice(s). | N/A |
|  | 13e | Describe any methods used to explore possible causes of inconsistency among study results (e.g. subgroup analysis). | N/A |
|  | 13f | If applicable, describe any sensitivity analyses conducted to assess robustness of the synthesized results. | N/A |
| Certainty assessment | 15 | Describe any methods used to assess certainty (or confidence) in the body of evidence. | N/A |
| Formulating recommendations | PC4 | If appropriate, describe any methods used to formulate recommendations regarding the suitability of an OMI for a particular use. | N/A |

| **Section and Topic** | **Item #** | **Checklist item** | **Location where item**  **is reported** |
| --- | --- | --- | --- |
| **RESULTS** | | | |
| Study selection | 16a | Describe the results of the search and selection process, from the number of records identified in the search to the number of reports included in the review, ideally using a flow diagram. If applicable, also report the final number of OMIs included and the number of reports relevant to each OMI. | p. 9-10 Figure 1. and Appendix E |
|  | 16b | Cite reports that might appear to meet the inclusion criteria, but which were excluded, and explain why they were excluded. | Appendix C |
| OMI characteristics | PC5 | Present characteristics of each included OMI, with appropriate citations. | Appendix G and H |
| Study characteristics | 17 | Cite each included report evaluating one or more measurement properties and present its characteristics. | Appendix G |
| Risk of bias in studies | 18 | Present assessments of risk of bias for each included study. | Appendix F |
| Results of individual studies | 19 | For all measurement properties, present, for each study: (a) the reported result and (b) the rating against quality criteria, ideally using structured tables or plots. | Appendix I |
| Results of syntheses | 20b | Present results of all syntheses conducted. For each measurement property of an OMI, present: (a) the summarized or pooled result and (b) the overall rating against quality criteria. | Table 2 |
|  | 20c | Present results of all investigations of possible causes of inconsistency among study results. | N/A |
|  | 20d | If applicable, present results of all sensitivity analyses conducted to assess the robustness of the synthesized results. | N/A |
| Certainty of evidence | 22 | Present assessments of certainty (or confidence) in the body of evidence for each measurement property of an OMI assessed. | N/A |
| Interpretability and feasibility | PC6 | Describe interpretability and feasibility aspects for each OMI. | N/A |
| Recommendations | PC7 | If appropriate, make recommendations for suitable OMIs for a particular use. | p. 13-16 and p. 18 |
| **DISCUSSION** | | | |
| Discussion | 23a | Provide a general interpretation of the results in the context of other evidence. | p. 16-19 |
|  | 23b | Discuss any limitations of the evidence included in the review. | p. 16-17 |
|  | 23c | Discuss any limitations of the review processes used. | p. 20 |
|  | 23d | Discuss implications of the results for practice, policy, and future research. | p. 20 Conclusion |
| **OTHER INFORMATION** | | | |
| Registration and protocol | 24a | Provide registration information for the review, including register name and registration number, or state that the review was not registered. | p. 5 |
|  | 24b | Indicate where the review protocol can be accessed, or state that a protocol was not prepared. | p. 5 |
|  | 24c | Describe and explain any amendments to information provided at registration or in the protocol. | N/A |
| Support | 25 | Describe sources of financial or non-financial support for the review, and the role of the funders or sponsors in the review. | Title page with author information |
| Competing interests | 26 | Declare any competing interests of review authors. | Title page with author information |
| Availability of data, code and other materials | 27 | Report which of the following are publicly available and where they can be found: template data collection forms; data extracted from included studies; data used for all analyses; analytic code; any other materials used in the review. | N/A all in Appendices |
