# Supplementary material for: Applicability and Psychometric Properties of General Mental Health Assessment Tools in Autistic People: A Systematic Review
Source: J Autism Dev Disord. 2024 Apr 13;55(5):1713–26. doi: 10.1007/s10803-024-06324-3 (PMC12021962; doi:10.1007/s10803-024-06324-3)
Supplement: Supplementary file 2 — Supplementary file2 (DOCX 50 KB) [file 10803_2024_6324_MOESM2_ESM.docx]

**Appendix B**

Applicability and psychometric properties of general mental health assessment tools in autistic people : A systematic Review.

**Search strategies**
Date of search: 22nd of March 2022
Date of updated search: 3^rd^ and 11^th^ of January 2024
Information specialist: Brynhildur Axelsdottir
 **Total number of hits from all databases: 14.929**Medline (Ovid): **2930**
PsycINFO (Ovid): **5446**
Embase (Ovid): **5072**
Web of Science (Clarivate): **1481**

**Total number of hits from all databases of the updated search in 2024:**Medline (Ovid): **289**
PsycINFO (Ovid): **505**
Embase (Ovid): **No access to the database**
Web of Science (Clarivate): **22**

**Ovid MEDLINE(R) and Epub Ahead of Print, In-Process, In-Data-Review & Other Non-Indexed Citations, Daily and Versions <1946 to March 21, 2022>**

| # | Searches | Results |  |  |  |
| --- | --- | --- | --- | --- | --- |
| 1 | autism spectrum disorder/ or asperger syndrome/ or autistic disorder/ or exp child development disorders, pervasive/ or rett syndrome/ | 45208 |  |  |  |
| 2 | (autis* or asperg* or kanner* or rett*).tw. | 118996 |  |  |  |
| 3 | (pervasiv* adj3 develop* adj3 disord*).tw. | 2081 |  |  |  |
| 4 | or/1-3 | 123712 |  |  |  |
| 5 | exp anxiety disorders/ or exp obsessive-compulsive disorder/ or exp panic disorder/ or phobic disorders/ or depressive disorder/ or depressive disorder, major/ or depressive disorder, treatment-resistant/ or dysthymic disorder/ or seasonal affective disorder/ or bipolar disorder/ or exp "disruptive, impulse control, and conduct disorders"/ or psychotic disorders/ or exp schizophrenia/ or stress disorders, traumatic/ or psychological trauma/ or stress disorders, post-traumatic/ or adjustment disorders/ | 335087 |  |  |  |
| 6 | (mental* or psych* or internali*ing* or externali*ing* or anxi* or depress* or ocd or obsessive* or mutism* or ptsd or post-traumatic* or ((conduct* or defian* or behavio* or affect* or challenging*) adj3 disorder*) or bipolar* or mania* or manic* or schizophreni* or (behavio* adj3 adjust*)).tw. | 1780076 |  |  |  |
| 7 | or/5-6 | 1837146 |  |  |  |
| 8 | Psychometrics/ or behavior rating scale/ or Psychological tests/ or Interview, Psychological/ or Interviews as Topic/ | 195800 |  |  |  |
| 9 | ((diagnos* or screen*) adj3 (algorithm* or assess* or interview* or instrument* or observation* or questionnaire* or schedule* or test* or tool*)).tw. | 317648 |  |  |  |
| 10 | or/8-9 | 503325 |  |  |  |
| 11 | 4 and 7 and 10 | 2113 |  |  |  |
| 12 | (aberrant* adj2 behavio* adj2 checklist*).ti,ab. | 498 |  |  |  |
| 13 | (behavio* adj2 problem* adj2 inventor*).ti,ab. | 84 |  |  |  |
| 14 | (challeng* adj2 behavio* adj2 inventor*).ti,ab. | 3 |  |  |  |
| 15 | (developmental* adj2 behavio* adj2 checklist*).ti,ab. | 108 |  |  |  |
| 16 | (nisonger* adj2 child* adj2 behavio* adj2 rating*).ti,ab. | 53 |  |  |  |
| 17 | (reiss* adj2 scale* adj2 child*).ti,ab. | 4 |  |  |  |
| 18 | (well* adj2 special* adj2 education* adj2 question*).ti,ab. | 2 |  |  |  |
| 19 | (achenbach* adj2 system* adj2 empirical*).ti,ab. | 97 |  |  |  |
| 20 | (behavio* adj2 problem* adj2 checklist*).ti,ab. | 270 |  |  |  |
| 21 | (strength* adj2 difficult* adj2 question*).ti,ab. | 2899 |  |  |  |
| 22 | (autism* adj2 comorbidit* adj2 interview*).ti,ab. | 4 |  |  |  |
| 23 | (autism* adj2 spectrum* adj2 disorder* adj2 comorbid* adj2 adult*).ti,ab. | 3 |  |  |  |
| 24 | (autism* adj2 spectrum* adj2 disorder* adj2 comorbid* adj2 child*).ti,ab. | 30 |  |  |  |
| 25 | (baby* adj2 infant* adj2 screen*).ti,ab. | 31 |  |  |  |
| 26 | (psychopathol* adj2 autism* adj2 checklist*).ti,ab. | 3 |  |  |  |
| 27 | (schedule* adj3 assess* adj2 psychiatric* adj2 problem*).ti,ab. | 0 |  |  |  |
| 28 | (child* adj2 behavio* adj2 checklist*).ti,ab. | 4601 |  |  |  |
| 29 | (assessment* adj2 concern* adj2 behavio*).ti,ab. | 9 |  |  |  |
| 30 | (reiss* adj2 screen* adj2 maladaptiv* adj2 behavio*).ti,ab. | 27 |  |  |  |
| 31 | (psychopatholog* adj2 checklist* adj2 adult* adj2 intellect* adj2 disabilit*).ti,ab. | 3 |  |  |  |
| 32 | (psychopatholog* adj2 checklist* adj2 adult*).ti,ab. | 4 |  |  |  |
| 33 | (child* adj2 adoles* adj2 symptom* adj2 inventor*).ti,ab. | 48 |  |  |  |
| 34 | (schedule* adj2 affectiv* adj2 disorder* adj2 schizophren*).ti,ab. | 1292 |  |  |  |
| 35 | (mini* adj2 neuropsychiatr* adj2 interview*).ti,ab. | 2336 |  |  |  |
| 36 | (structured* adj2 clinical* adj2 assessment*).ti,ab. | 230 |  |  |  |
| 37 | (pas* adj2 add* adj2 checklist*).ti,ab. | 17 |  |  |  |
| 38 | (assessment* adj2 dual* adj2 diagnosis*).ti,ab. | 8 |  |  |  |
| 39 | (diagnostic* adj2 assessment* adj2 severe* adj2 handicap*).ti,ab. | 0 |  |  |  |
| 40 | (psychopatholog* adj2 instrument* adj2 mental* adj2 retard*).ti,ab. | 23 |  |  |  |
| 41 | (bpi* or cbi* or wellseq* or aseba* or cbcl* or bpc* or sdq* or aci-pl* or asd-ca or asd-cc or biscuit or pac or saapa* or acb or casi or kiddie-sad* or scid or pas-add* or rsmb or pimra* or p-aid*).ti,ab. | 51045 |  |  |  |
| 42 | or/12-41 | 59209 |  |  |  |
| 43 | 4 and 42 | 1230 |  |  |  |
| 44 | 11 or 43 | 3143 |  |  |  |
| 45 | limit 44 to yr="1980 -Current" | 3102 |  |  |  |
| 46 | limit 45 to english | 2930 |  |  |  |

**APA PsycInfo <1967 to March Week 2 2022>**

| # | Searches | Results |  |  |  |
| --- | --- | --- | --- | --- | --- |
| 1 | autism spectrum disorders/ or autistic traits/ or rett syndrome/ | 50374 |  |  |  |
| 2 | (autis* or asperg* or kanner* or rett*).tw. | 62271 |  |  |  |
| 3 | (pervasiv* adj3 develop* adj3 disord*).tw. | 3177 |  |  |  |
| 4 | or/1-3 | 63678 |  |  |  |
| 5 | exp anxiety disorders/ or depressive disorder/ or depressive disorder, major/ or depressive disorder, treatment-resistant/ or dysthymic disorder/ or seasonal affective disorder/ or exp bipolar disorder/ or disruptive behavior disorders/ or conduct disorder/ or oppositional defiant disorder/ or psychosis/ or acute psychosis/ or affective psychosis/ or exp childhood psychosis/ or chronic psychosis/ or exp schizophrenia/ or traumatic experiences/ or posttraumatic stress/ or exp posttraumatic stress disorder/ or adjustment disorders/ | 240975 |  |  |  |
| 6 | (mental* or psych* or internali*ing* or externali*ing* or anxi* or depress* or ocd or obsessive* or mutism* or ptsd or post-traumatic* or ((conduct* or defian* or behavio* or affect* or challenging*) adj3 disorder*) or bipolar* or mania* or manic* or schizophreni* or (behavio* adj3 adjust*)).tw. | 1738082 |  |  |  |
| 7 | or/5-6 | 1746533 |  |  |  |
| 8 | exp psychometrics/ or psychological assessment/ or exp behavioral assessment/ or exp psychodiagnostic interview/ | 226710 |  |  |  |
| 9 | ((diagnos* or screen*) adj3 (algorithm* or assess* or interview* or instrument* or observation* or questionnaire* or schedule* or test* or tool*)).tw. | 63795 |  |  |  |
| 10 | or/8-9 | 276846 |  |  |  |
| 11 | 4 and 7 and 10 | 3028 |  |  |  |
| 12 | child behavior checklist/ | 496 |  |  |  |
| 13 | (aberrant* adj2 behavio* adj2 checklist*).ti,ab,tm. | 1037 |  |  |  |
| 14 | (behavio* adj2 problem* adj2 inventor*).ti,ab,tm. | 271 |  |  |  |
| 15 | (challeng* adj2 behavio* adj2 inventor*).ti,ab,tm. | 3 |  |  |  |
| 16 | (developmental* adj2 behavio* adj2 checklist*).ti,ab,tm. | 299 |  |  |  |
| 17 | (nisonger* adj2 child* adj2 behavio* adj2 rating*).ti,ab,tm. | 149 |  |  |  |
| 18 | (reiss* adj2 scale* adj2 child*).ti,ab,tm. | 19 |  |  |  |
| 19 | (well* adj2 special* adj2 education* adj2 question*).ti,ab,tm. | 2 |  |  |  |
| 20 | (achenbach* adj2 system* adj2 empirical*).ti,ab,tm. | 586 |  |  |  |
| 21 | (behavio* adj2 problem* adj2 checklist*).ti,ab,tm. | 1156 |  |  |  |
| 22 | (strength* adj2 difficult* adj2 question*).ti,ab,tm. | 7592 |  |  |  |
| 23 | (autism* adj2 comorbidit* adj2 interview*).ti,ab,tm. | 37 |  |  |  |
| 24 | (autism* adj2 spectrum* adj2 disorder* adj2 comorbid* adj2 adult*).ti,ab,tm. | 17 |  |  |  |
| 25 | (autism* adj2 spectrum* adj2 disorder* adj2 comorbid* adj2 child*).ti,ab,tm. | 70 |  |  |  |
| 26 | (baby* adj2 infant* adj2 screen*).ti,ab,tm. | 139 |  |  |  |
| 27 | (psychopathol* adj2 autism* adj2 checklist*).ti,ab,tm. | 21 |  |  |  |
| 28 | (schedule* adj3 assess* adj2 psychiatric* adj2 problem*).ti,ab,tm. | 7 |  |  |  |
| 29 | (child* adj2 behavio* adj2 checklist*).ti,ab,tm. | 18629 |  |  |  |
| 30 | (assessment* adj2 concern* adj2 behavio*).ti,ab,tm. | 11 |  |  |  |
| 31 | (reiss* adj2 screen* adj2 maladaptiv* adj2 behavio*).ti,ab,tm. | 89 |  |  |  |
| 32 | (psychopatholog* adj2 checklist* adj2 adult* adj2 intellect* adj2 disabilit*).ti,ab,tm. | 10 |  |  |  |
| 33 | (psychopatholog* adj2 checklist* adj2 adult*).ti,ab,tm. | 11 |  |  |  |
| 34 | (child* adj2 adoles* adj2 symptom* adj2 inventor*).ti,ab,tm. | 203 |  |  |  |
| 35 | (schedule* adj2 affectiv* adj2 disorder* adj2 schizophren*).ti,ab,tm. | 8808 |  |  |  |
| 36 | (mini* adj2 neuropsychiatr* adj2 interview*).ti,ab,tm. | 13216 |  |  |  |
| 37 | (structured* adj2 clinical* adj2 assessment*).ti,ab,tm. | 138 |  |  |  |
| 38 | (pas* adj2 add* adj2 checklist*).ti,ab,tm. | 41 |  |  |  |
| 39 | (assessment* adj2 dual* adj2 diagnosis*).ti,ab,tm. | 45 |  |  |  |
| 40 | (diagnostic* adj2 assessment* adj2 severe* adj2 handicap*).ti,ab,tm. | 0 |  |  |  |
| 41 | (psychopatholog* adj2 instrument* adj2 mental* adj2 retard*).ti,ab,tm. | 59 |  |  |  |
| 42 | (bpi* or cbi* or wellseq* or aseba* or cbcl* or bpc* or sdq* or aci-pl* or asd-ca or asd-cc or biscuit or pac or saapa* or acb or casi or kiddie-sad* or scid or pas-add* or rsmb or pimra* or p-aid*).ti,ab,tm. | 12683 |  |  |  |
| 43 | or/12-42 | 56878 |  |  |  |
| 44 | 4 and 43 | 3232 |  |  |  |
| 45 | 11 or 44 | 5785 |  |  |  |
| 46 | limit 45 to yr="1980 -Current" | 5774 |  |  |  |
| 47 | limit 46 to english | 5446 |  |  |  |

**Embase <1974 to 2022 March 21>**

| # | Searches | Results |  |  |  |
| --- | --- | --- | --- | --- | --- |
| 1 | autism/ or asperger syndrome/ or "pervasive developmental disorder not otherwise specified"/ or rett syndrome/ | 82153 |  |  |  |
| 2 | (autis* or asperg* or kanner* or rett*).tw. | 151493 |  |  |  |
| 3 | (pervasiv* adj3 develop* adj3 disord*).tw. | 2908 |  |  |  |
| 4 | or/1-3 | 167303 |  |  |  |
| 5 | anxiety disorder/ or generalized anxiety disorder/ or "mixed anxiety and depression"/ or exp obsessive compulsive disorder/ or panic/ or exp posttraumatic stress disorder/ or panic/ or phobia/ or depression/ or adolescent depression/ or chronic depression/ or dysphoria/ or dysthymia/ or major depression/ or seasonal affective disorder/ or treatment resistant depression/ or bipolar disorder/ or disruptive behavior/ or conduct disorder/ or psychosis/ or exp schizophrenia/ or psychotrauma/ or posttraumatic stress disorder/ or adjustment disorder/ | 848256 |  |  |  |
| 6 | (mental* or psych* or internali*ing* or externali*ing* or anxi* or depress* or ocd or obsessive* or mutism* or ptsd or post-traumatic* or ((conduct* or defian* or behavio* or affect* or challenging*) adj3 disorder*) or bipolar* or mania* or manic* or schizophreni* or (behavio* adj3 adjust*)).tw. | 2282115 |  |  |  |
| 7 | or/5-6 | 2434924 |  |  |  |
| 8 | psychometry/ or behavior assessment/ or psychologic test/ or psychological interview/ | 109238 |  |  |  |
| 9 | ((diagnos* or screen*) adj3 (algorithm* or assess* or interview* or instrument* or observation* or questionnaire* or schedule* or test* or tool*)).tw. | 456017 |  |  |  |
| 10 | or/8-9 | 558647 |  |  |  |
| 11 | 4 and 7 and 10 | 3529 |  |  |  |
| 12 | aberrant behavior checklist/ or child behavior checklist/ | 5492 |  |  |  |
| 13 | (aberrant* adj2 behavio* adj2 checklist*).ti,ab. | 695 |  |  |  |
| 14 | (behavio* adj2 problem* adj2 inventor*).ti,ab. | 114 |  |  |  |
| 15 | (challeng* adj2 behavio* adj2 inventor*).ti,ab. | 3 |  |  |  |
| 16 | (developmental* adj2 behavio* adj2 checklist*).ti,ab. | 157 |  |  |  |
| 17 | (nisonger* adj2 child* adj2 behavio* adj2 rating*).ti,ab. | 58 |  |  |  |
| 18 | (reiss* adj2 scale* adj2 child*).ti,ab. | 6 |  |  |  |
| 19 | (well* adj2 special* adj2 education* adj2 question*).ti,ab. | 2 |  |  |  |
| 20 | (achenbach* adj2 system* adj2 empirical*).ti,ab. | 166 |  |  |  |
| 21 | (behavio* adj2 problem* adj2 checklist*).ti,ab. | 320 |  |  |  |
| 22 | (strength* adj2 difficult* adj2 question*).ti,ab. | 3666 |  |  |  |
| 23 | (autism* adj2 comorbidit* adj2 interview*).ti,ab. | 8 |  |  |  |
| 24 | (autism* adj2 spectrum* adj2 disorder* adj2 comorbid* adj2 adult*).ti,ab. | 4 |  |  |  |
| 25 | (autism* adj2 spectrum* adj2 disorder* adj2 comorbid* adj2 child*).ti,ab. | 50 |  |  |  |
| 26 | (baby* adj2 infant* adj2 screen*).ti,ab. | 55 |  |  |  |
| 27 | (psychopathol* adj2 autism* adj2 checklist*).ti,ab. | 11 |  |  |  |
| 28 | (schedule* adj3 assess* adj2 psychiatric* adj2 problem*).ti,ab. | 0 |  |  |  |
| 29 | (child* adj2 behavio* adj2 checklist*).ti,ab. | 6199 |  |  |  |
| 30 | (assessment* adj2 concern* adj2 behavio*).ti,ab. | 9 |  |  |  |
| 31 | (reiss* adj2 screen* adj2 maladaptiv* adj2 behavio*).ti,ab. | 30 |  |  |  |
| 32 | (psychopatholog* adj2 checklist* adj2 adult* adj2 intellect* adj2 disabilit*).ti,ab. | 4 |  |  |  |
| 33 | (psychopatholog* adj2 checklist* adj2 adult*).ti,ab. | 5 |  |  |  |
| 34 | (child* adj2 adoles* adj2 symptom* adj2 inventor*).ti,ab. | 55 |  |  |  |
| 35 | (schedule* adj2 affectiv* adj2 disorder* adj2 schizophren*).ti,ab. | 1789 |  |  |  |
| 36 | (mini* adj2 neuropsychiatr* adj2 interview*).ti,ab. | 3372 |  |  |  |
| 37 | (structured* adj2 clinical* adj2 assessment*).ti,ab. | 323 |  |  |  |
| 38 | (pas* adj2 add* adj2 checklist*).ti,ab. | 25 |  |  |  |
| 39 | (assessment* adj2 dual* adj2 diagnosis*).ti,ab. | 14 |  |  |  |
| 40 | (diagnostic* adj2 assessment* adj2 severe* adj2 handicap*).ti,ab. | 0 |  |  |  |
| 41 | (psychopatholog* adj2 instrument* adj2 mental* adj2 retard*).ti,ab. | 25 |  |  |  |
| 42 | (bpi* or cbi* or wellseq* or aseba* or cbcl* or bpc* or sdq* or aci-pl* or asd-ca or asd-cc or biscuit or pac or saapa* or acb or casi or kiddie-sad* or scid or pas-add* or rsmb or pimra* or p-aid*).ti,ab. | 72293 |  |  |  |
| 43 | or/12-42 | 84523 |  |  |  |
| 44 | 4 and 43 | 2228 |  |  |  |
| 45 | 11 or 44 | 5314 |  |  |  |
| 46 | limit 45 to yr="1980 -Current" | 5286 |  |  |  |
| 47 | limit 46 to english | 5072 |  |  |  |

**Web of Science (Clarivate)**

(TS=((autis* or asperg* or kanner* or rett*)) OR TS=((pervasiv* NEAR/3 develop* NEAR/3 disord*)) AND TS=((mental* or psych* or internali*ing* or externali*ing* or anxi* or depress* or ocd or obsessive* or mutism* or ptsd or post-traumatic* or ((conduct* or defian* or behavio* or affect* or challenging*) NEAR/3 disorder*) or bipolar* or mania* or manic* or schizophreni* or (behavio* NEAR/3 adjust*))) AND TS=(((diagnos* or screen*) NEAR/3 (algorithm* or assess* or interview* or instrument* or observation* or questionnaire* or schedule* or test* or tool*))))

OR (TS=(aberrant* NEAR/2 behavio* NEAR/2 checklist*) OR TS=(behavio* NEAR/2 problem* NEAR/2 inventor*) OR TS=(challeng* NEAR/2 behavio* NEAR/2 inventor*) OR TS=(developmental* NEAR/2 behavio* NEAR/2 checklist*) OR TS=(nisonger* NEAR/2 child* NEAR/2 behavio* NEAR/2 rating*) OR TS=(reiss* NEAR/2 scale* NEAR/2 child*) OR TS=(well* NEAR/2 special* NEAR/2 education* NEAR/2 question*) OR TS=(achenbach* NEAR/2 system* NEAR/2 empirical*) OR TS=(behavio* NEAR/2 problem* NEAR/2 checklist*) OR TS=(strength* NEAR/2 difficult* NEAR/2 question*) OR TS= (autism* NEAR/2 comorbidit* NEAR/2 interview*) OR TS=(autism* NEAR/2 spectrum* NEAR/2 disorder* NEAR/2 comorbid* NEAR/2 adult*) OR TS=(autism* NEAR/2 spectrum* NEAR/2 disorder* NEAR/2 comorbid* NEAR/2 child*) OR TS=(baby* NEAR/2 infant* NEAR/2 screen*) OR TS=(psychopathol* NEAR/2 autism* NEAR/2 checklist*) OR TS=(schedule* NEAR/3 assess* NEAR/2 psychiatric* NEAR/2 problem*) OR TS=(child* NEAR/2 behavio* NEAR/2 checklist*) OR TS=(assessment* NEAR/2 concern* NEAR/2 behavio*) OR TS=(reiss* NEAR/2 screen* NEAR/2 maladaptiv* NEAR/2 behavio*) OR TS=(psychopatholog* NEAR/2 checklist* NEAR/2 adult* NEAR/2 intellect* NEAR/2 disabilit*) OR TS=(psychopatholog* NEAR/2 checklist* NEAR/2 adult*) OR TS=(child* NEAR/2 adoles* NEAR/2 symptom* NEAR/2 inventor*) OR TS=(schedule* NEAR/2 affectiv* NEAR/2 disorder* NEAR/2 schizophren*) OR TS=(mini* NEAR/2 neuropsychiatr* NEAR/2 interview*) OR TS=(structured* NEAR/2 clinical* NEAR/2 assessment*) OR TS=(pas* NEAR/2 add* NEAR/2 checklist*) OR TS=(assessment* NEAR/2 dual* NEAR/2 diagnosis*) OR TS=(diagnostic* NEAR/2 assessment* NEAR/2 severe* NEAR/2 handicap*) OR TS=(psychopatholog* NEAR/2 instrument* NEAR/2 mental* NEAR/2 retard*) OR TS=(bpi* or cbi* or wellseq* or aseba* or cbcl* or bpc* or sdq* or aci-pl* or asd-ca or asd-cc or biscuit or pac or saapa* or acb or casi or kiddie-sad* or scid or pas-add* or rsmb or pimra* or p-aid*))
**Hits: 1481**

**Updated searches January 3^rd^  and 11^th^ 2024**

**APA PsycInfo <1806 to January Week 1 2024>**

Searches Results

1 autism spectrum disorders/ or autistic traits/ or rett syndrome/ 57634

2 (autis* or asperg* or kanner* or rett*).tw. 70422

3 (pervasiv* adj3 develop* adj3 disord*).tw. 3223

4 or/1-3 71890

5 child behavior checklist/ 551

6 (aberrant* adj2 behavio* adj2 checklist*).ti,ab,tm. 1129

7 (behavio* adj2 problem* adj2 inventor*).ti,ab,tm. 283

8 (challeng* adj2 behavio* adj2 inventor*).ti,ab,tm. 3

9 (developmental* adj2 behavio* adj2 checklist*).ti,ab,tm. 314

10 (nisonger* adj2 child* adj2 behavio* adj2 rating*).ti,ab,tm. 154

11 (reiss* adj2 scale* adj2 child*).ti,ab,tm. 19

12 (well* adj2 special* adj2 education* adj2 question*).ti,ab,tm. 2

13 (achenbach* adj2 system* adj2 empirical*).ti,ab,tm. 665

14 (behavio* adj2 problem* adj2 checklist*).ti,ab,tm. 1192

15 (strength* adj2 difficult* adj2 question*).ti,ab,tm. 8855

16 (autism* adj2 comorbidit* adj2 interview*).ti,ab,tm. 37

17 (autism* adj2 spectrum* adj2 disorder* adj2 comorbid* adj2 adult*).ti,ab,tm. 21

18 (autism* adj2 spectrum* adj2 disorder* adj2 comorbid* adj2 child*).ti,ab,tm. 82

19 (baby* adj2 infant* adj2 screen*).ti,ab,tm. 156

20 (psychopathol* adj2 autism* adj2 checklist*).ti,ab,tm. 22

21 (schedule* adj3 assess* adj2 psychiatric* adj2 problem*).ti,ab,tm. 7

22 (child* adj2 behavio* adj2 checklist*).ti,ab,tm. 19945

23 (assessment* adj2 concern* adj2 behavio*).ti,ab,tm. 11

24 (reiss* adj2 screen* adj2 maladaptiv* adj2 behavio*).ti,ab,tm. 90

25 (psychopatholog* adj2 checklist* adj2 adult* adj2 intellect* adj2 disabilit*).ti,ab,tm. 10

26 (psychopatholog* adj2 checklist* adj2 adult*).ti,ab,tm. 11

27 (child* adj2 adoles* adj2 symptom* adj2 inventor*).ti,ab,tm. 240

28 (schedule* adj2 affectiv* adj2 disorder* adj2 schizophren*).ti,ab,tm. 9493

29 (mini* adj2 neuropsychiatr* adj2 interview*).ti,ab,tm. 14828

30 (structured* adj2 clinical* adj2 assessment*).ti,ab,tm. 143

31 (pas* adj2 add* adj2 checklist*).ti,ab,tm. 42

32 (assessment* adj2 dual* adj2 diagnosis*).ti,ab,tm. 46

33 (diagnostic* adj2 assessment* adj2 severe* adj2 handicap*).ti,ab,tm. 0

34 (psychopatholog* adj2 instrument* adj2 mental* adj2 retard*).ti,ab,tm. 59

35 (revised* adj2 child* adj2 anxiet* adj2 depress*).ti,ab,tm. 907

36(emotion* adj2 dysregulat* adj2 inventor*).ti,ab,tm. 30

37 (bpi* or cbi* or wellseq* or aseba* or cbcl* or bpc* or sdq* or aci-pl* or asd-ca or asd-cc or biscuit or pac or saapa* or acb or casi or kiddie-sad* or scid or pas-add* or rsmb or pimra* or p-aid* or rcad* or edi).ti,ab,tm. 15333

38 or/5-37 64551

39 4 and 38 367

40 (2022* or 2023* or 2024*).dp,up. 399142

41 39 and 40 505

**Ovid MEDLINE(R) ALL <1946 to January 02, 2024>**

Searches Results

1 autism spectrum disorder/ or asperger syndrome/ or autistic disorder/
or exp child development disorders, pervasive/ or rett syndrome/ 51990

2 (autis* or asperg* or kanner* or rett*).tw. 135295

3 (pervasiv* adj3 develop* adj3 disord*).tw. 2131

4 or/1-3 140192

5 (aberrant* adj2 behavio* adj2 checklist*).ti,ab. 558

6 (behavio* adj2 problem* adj2 inventor*).ti,ab. 85

7 (challeng* adj2 behavio* adj2 inventor*).ti,ab. 3

8 (developmental* adj2 behavio* adj2 checklist*).ti,ab. 111

9 (nisonger* adj2 child* adj2 behavio* adj2 rating*).ti,ab. 56

10 (reiss* adj2 scale* adj2 child*).ti,ab. 4

11 (well* adj2 special* adj2 education* adj2 question*).ti,ab. 2

12 (achenbach* adj2 system* adj2 empirical*).ti,ab. 116

13 (behavio* adj2 problem* adj2 checklist*).ti,ab. 277

14 (strength* adj2 difficult* adj2 question*).ti,ab. 3494

15 (autism* adj2 comorbidit* adj2 interview*).ti,ab. 4

16 (autism* adj2 spectrum* adj2 disorder* adj2 comorbid* adj2 adult*).ti,ab. 4

17 (autism* adj2 spectrum* adj2 disorder* adj2 comorbid* adj2 child*).ti,ab. 35

18 (baby* adj2 infant* adj2 screen*).ti,ab. 31

19 (psychopathol* adj2 autism* adj2 checklist*).ti,ab. 5

20 (schedule* adj3 assess* adj2 psychiatric* adj2 problem*).ti,ab. 0

21 (child* adj2 behavio* adj2 checklist*).ti,ab. 5104

22 (assessment* adj2 concern* adj2 behavio*).ti,ab. 11

23 (reiss* adj2 screen* adj2 maladaptiv* adj2 behavio*).ti,ab. 27

24 (psychopatholog* adj2 checklist* adj2 adult* adj2 intellect* adj2 disabilit*).ti,ab. 3

25 (psychopatholog* adj2 checklist* adj2 adult*).ti,ab. 4

26 (child* adj2 adoles* adj2 symptom* adj2 inventor*).ti,ab. 57

27 (schedule* adj2 affectiv* adj2 disorder* adj2 schizophren*).ti,ab. 1393

28 (mini* adj2 neuropsychiatr* adj2 interview*).ti,ab. 2625

29 (structured* adj2 clinical* adj2 assessment*).ti,ab. 249

30 (pas* adj2 add* adj2 checklist*).ti,ab. 17

31 (assessment* adj2 dual* adj2 diagnosis*).ti,ab. 8

32 (diagnostic* adj2 assessment* adj2 severe* adj2 handicap*).ti,ab. 0

33 (psychopatholog* adj2 instrument* adj2 mental* adj2 retard*).ti,ab. 23

34 (revised* adj2 child* adj2 anxiet* adj2 depress*).ti,ab. 206

35 (emotion* adj2 dysregulat* adj2 inventor*).ti,ab. 18

36 (bpi* or cbi* or wellseq* or aseba* or cbcl* or bpc* or sdq* or aci-pl* or asd-ca or asd-cc or biscuit or pac or saapa* or acb or casi or kiddie-sad* or scid or pas-add* or rsmb or pimra* or p-aid* or rcad* or edi).ti,ab. 62145

37 or/5-36 71320

38 4 and 37 1470

39 (2022* or 2023* or 2024*).dt,dp,ed. 3659080

40 38 and 39 289

**Web of Science (Clarivate)**

TS=(autis* or asperger* or kanner* or rett* or pervasiv*) AND TS=(diagnos* or assess* or interview* or instrument* or observation* or questionnaire* or schedule* or test* or tool*)

Date range: 2022-2024 Hits: 22
