# Supplementary material for: Applicability and Psychometric Properties of General Mental Health Assessment Tools in Autistic People: A Systematic Review
Source: J Autism Dev Disord. 2024 Apr 13;55(5):1713–26. doi: 10.1007/s10803-024-06324-3 (PMC12021962; doi:10.1007/s10803-024-06324-3)
Supplement: Supplementary file 3 — Supplementary file3 (DOCX 132 KB) [file 10803_2024_6324_MOESM3_ESM.docx]

**Appendix C**

**Excluded references with reasons for exclusion.**

**Excluded references from the updated search January 2024:**

Alateyat, H., Cruz, S., Cernadas, E., Tubio-Fungueirino, M., Sampaio, A., Gonzalez-Villar, A., . . . Fernandez-Prieto, M. (2022). A Machine Learning Approach in Autism Spectrum Disorders: From Sensory Processing to Behavior Problems. *Frontiers in Molecular Neuroscience, 15*, 889641. doi: https://dx.doi.org/10.3389/fnmol.2022.889641 Exclusion reason: No psychometric information.

Alder, M., Johnson, C., Zauszniewski, J., Malow, B., Burant, C., & Scahill, L. (2023). Feasibility of actigraphy for evaluating sleep and daytime physical activity in children with autism spectrum disorder. *Journal of Autism and Developmental Disorders, 53*(9), 3670-3682. doi:https://dx.doi.org/10.1007/s10803-022-05661-5. Exclusion reason: No psychometric information

Andersen, P. N., Orm, S., Fossum, I. N., Oie, M. G., & Skogli, E. W. (2023). Adolescence internalizing problems as a mediator between autism diagnosis in childhood and quality of life in emerging adults with and without autism: A 10-year longitudinal study. *BMC Psychiatry Vol 23,(1), 2023, ArtID 149, 23*(1). doi:https://dx.doi.org/10.1186/s12888-023-04635-w. Exclusion reason: No psychometric information

Andrzejewski, T., DeLucia, E. A., Semones, O., Khan, S., & McDonnell, C. G. (2023). Adverse childhood experiences in autistic children and their caregivers: Examining intergenerational continuity. *Journal of Autism and Developmental Disorders, 53*(7), 2663-2679. doi:https://dx.doi.org/10.1007/s10803-022-05551-w. Exclusion reason: Non-ASD sample

Aslan Genc, H., Doenyas, C., Aksu, Y., Musaoglu, M. N., Uzunay, S., & Mutluer, T. (2023). Long-term behavioral consequences of the covid-19 pandemic for autistic individuals and their mothers. *Journal of Autism and Developmental Disorders*, No Pagination Specified. doi:https://dx.doi.org/10.1007/s10803-023-05933-8. Exclusion reason: No psychometric information

Bellato, A., Arora, I., Kochhar, P., Ropar, D., Hollis, C., & Groom, M. J. (2022). Heart rate variability in children and adolescents with autism, ADHD and co-occurring autism and ADHD, during passive and active experimental conditions. *Journal of Autism and Developmental Disorders, 52*(11), 4679-4691. doi:https://dx.doi.org/10.1007/s10803-021-05244-w. Exclusion reason: No psychometric information

Bellato, A., Arora, I., Kochhar, P., Ropar, D., Hollis, C., & Groom, M. J. (2023). Relationship between autonomic arousal and attention orienting in children and adolescents with ADHD, autism and co-occurring ADHD and autism. *Cortex: A Journal Devoted to the Study of the Nervous System and Behavior, 166*, 306-321. doi:https://dx.doi.org/10.1016/j.cortex.2023.06.002. Exclusion reason: No psychometric information;

Benson, A. A., Mughal, R., Dimitriou, D., & Halstead, E. J. (2023). Towards a Distinct Sleep and Behavioural Profile of Fetal Alcohol Spectrum Disorder (FASD): A Comparison between FASD, Autism and Typically Developing Children. *Journal of Integrative Neuroscience, 22*(3), 77. doi:https://dx.doi.org/10.31083/j.jin2203077. Exclusion reason: No psychometric information

Bitsika, V., Sharpley, C., & Heyne, D. (2022). Risk for school refusal among autistic boys bullied at school: Investigating associations with social phobia and separation anxiety. *International Journal of Disability, Development and Education, 69*(1), 190-203. doi:https://dx.doi.org/10.1080/1034912X.2021.1969544. Exclusion reason: Not relevant measurement tool

Bortoletto, R., Bassani, L., Garzitto, M., Lamberti, M., Simonati, A., Darra, F., . . . Colizzi, M. (2023). Risk of psychosis in autism spectrum disorder individuals exposed to psychosocial stressors: A 9-year chart review study. *Autism Research, 16*(11), 2139-2149. doi:https://dx.doi.org/10.1002/aur.3042. Exclusion reason: No psychometric information

Boulton, K. A., Guastella, A. J., Hodge, M. A., Demetriou, E. A., Ong, N., & Silove, N. (2023). Mental health concerns in children with neurodevelopmental conditions attending a developmental assessment service. *Journal of Affective Disorders, 335*, 264-272. doi:https://dx.doi.org/10.1016/j.jad.2023.04.098. Exclusion reason: Non-ASD sample;.

Bradbury, K. R., Anderberg, E. I., Huang-Storms, L., Vasile, I., Greene, R. K., & Duvall, S. W. (2022). Co-occurring Down syndrome and autism spectrum disorder: Cognitive, adaptive, and behavioral characteristics. *Journal of Autism and Developmental Disorders, 52*(3), 1235-1246. doi:https://dx.doi.org/10.1007/s10803-021-05016-6. Exclusion reason: No psychometric information

Chiu, H. T., Ip, I. N., Ching, F. N. Y., Wong, B. P., Lui, W. H., Tse, C. S., & Wong, S. W. H. (2023). Resting Heart Rate Variability and Emotion Dysregulation in Adolescents with Autism Spectrum Disorder. *Journal of Autism & Developmental Disorders, 30*, 30. doi:https://dx.doi.org/10.1007/s10803-022-05847-x. Exclusion reason: No psychometric information

Choi, H., Kim, J. H., Kim, H., & Cheon, K. A. (2023). Identifying major predictors for parenting stress in a caregiver of autism spectrum disorder using machine learning models. *Frontiers in Neuroscience, 17*, 1229155. doi:https://dx.doi.org/10.3389/fnins.2023.1229155. Exclusion reason: No psychometric information

Davico, C., Marcotulli, D., Cudia, V. F., Arletti, L., Ghiggia, A., Svevi, B., . . . Vitiello, B. (2022). Emotional Dysregulation and Adaptive Functioning in Preschoolers With Autism Spectrum Disorder or Other Neurodevelopmental Disorders. *Frontiers in psychiatry Frontiers Research Foundation, 13*, 846146. doi:https://dx.doi.org/10.3389/fpsyt.2022.846146. Exclusion reason: No psychometric information

Davies, J., Glinn, L., Osborne, L. A., & Reed, P. (2023). Exploratory study of parenting differences for autism spectrum disorder and attachment disorder. *Journal of Autism and Developmental Disorders, 53*(5), 2143-2152. doi:https://dx.doi.org/10.1007/s10803-022-05531-0. Exclusion reason: Non-ASD sample

Day, T. C., Gerber, A., McNair, M. L., Reicher, D., & Lerner, M. D. (2023). Trajectories of internalizing symptoms among autistic and nonautistic youth during the COVID-19 pandemic. *Autism research : Official Journal of the International Society for Autism Research, 16*(7), 1403-1412. doi:https://dx.doi.org/10.1002/aur.2959. Exclusion reason: No psychometric information

Day, T. N., Northrup, J. B., & Mazefsky, C. A. (2023). A PROMISing new measure for quantifying emotion dysregulation in toddlers and preschoolers: Development of the emotion dysregulation inventory-young child. *Journal of Autism and Developmental Disorders, 53*(6), 2261-2273. doi:https://dx.doi.org/10.1007/s10803-022-05536-9. Exclusion reason: Sample size = < 20

Dellapiazza, F., Michelon, C., Picot, M. C., & Baghdadli, A. (2022). Early risk factors for anxiety disorders in children with autism spectrum disorders: results from the ELENA Cohort. *Scientific Reports, 12*(1), 10914. doi:https://dx.doi.org/10.1038/s41598-022-15165-y. Exclusion reason: No psychometric information;

DeLucia, E. A., McKenna, M. P., Andrzejewski, T. M., Valentino, K., & McDonnell, C. G. (2022). A pilot study of self-regulation and behavior problems in preschoolers with ASD: Parent broader autism phenotype traits relate to child emotion regulation and inhibitory control. *Journal of Autism and Developmental Disorders, 52*(10), 4397-4411. doi:https://dx.doi.org/10.1007/s10803-021-05322-z. Exclusion reason: No psychometric information

Dimachkie Nunnally, A., Factor, R. S., Sturm, A., Valluripalli Soorya, L., Wainer, A., Taylor, S., . . . Gulsrud, A. C. (2023). Examining indicators of psychosocial risk and resilience in parents of autistic children. *Frontiers in Behavioral Neuroscience Vol 17, 2023, ArtID 1102516, 17*. doi:https://dx.doi.org/10.3389/fnbeh.2023.1102516. Exclusion reason: No psychometric information

Distefano, G., Calderoni, S., Apicella, F., Cosenza, A., Igliozzi, R., Palermo, G., . . . Turi, M. (2023). Impact of sleep disorders on behavioral issues in preschoolers with autism spectrum disorder. *Frontiers in psychiatry Frontiers Research Foundation, 14*, 1181466. doi:https://dx.doi.org/10.3389/fpsyt.2023.1181466. Exclusion reason: No psychometric information

Dong, H., Wang, T., Feng, J., Xue, Y., & Jia, F. (2023). The relationship between screen time before bedtime and behaviors of preschoolers with autism spectrum disorder and the mediating effects of sleep. *BMC Psychiatry Vol 23,(1), 2023, ArtID 635, 23*(1). doi:https://dx.doi.org/10.1186/s12888-023-05128-6. Exclusion reason: No psychometric information

Dooley, N., Clarke, M., Cotter, D., & Cannon, M. (2022). Birth weight and childhood psychopathology in the ABCD cohort: Association is strongest for attention problems and is moderated by sex. *Research on Child and Adolescent Psychopathology, 50*(5), 563-575. doi:https://dx.doi.org/10.1007/s10802-021-00859-0. Exclusion reason: Non-ASD sample

Dovgan, K., Gynegrowski, K., & Ferguson, B. J. (2023). Bidirectional relationship between internalizing symptoms and gastrointestinal problems in youth with autism spectrum disorder. *Journal of Autism and Developmental Disorders, 53*(11), 4488-4494. doi:https://dx.doi.org/10.1007/s10803-022-05539-6. Exclusion reason: No psychometric information

Drusedau, L. L., Gotz, A., Kleine Buning, L., Conzelmann, A., Renner, T. J., & Barth, G. M. (2023). Tubinger training for autism spectrum disorders (TuTASS): A structured group intervention on self-perception and social skills of children with autism spectrum disorder (ASD). *European Archives of Psychiatry and Clinical Neuroscience, 273*(7), 1599-1613. doi:https://dx.doi.org/10.1007/s00406-022-01537-y. Exclusion reason: No psychometric information

Dwyer, P., Ferrer, E., Saron, C. D., & Rivera, S. M. (2022). Exploring sensory subgroups in typical development and autism spectrum development using factor mixture modelling. *Journal of Autism and Developmental Disorders, 52*(9), 3840-3860. doi:https://dx.doi.org/10.1007/s10803-021-05256-6. Exclusion reason: No psychometric information

Eapen, V., Islam, R., Azim, S. I., Masi, A., Klein, L., & Karlov, L. (2022). Factors impacting parental quality of life in preschool children on the autism spectrum. *Journal of Autism and Developmental Disorders*, No Pagination Specified. doi:https://dx.doi.org/10.1007/s10803-022-05848-w. Exclusion reason: No psychometric information

Ebrahimi, P., Seyedmirzaei, H., Moradi, K., Bagheri, S., Moeini, M., Mohammadi, M. R., & Akhondzadeh, S. (2023). Cilostazol as adjunctive therapy in treatment of children with autism spectrum disorders: a double-blind and placebo-controlled randomized trial. *International Clinical Psychopharmacology, 38*(2), 89-95. doi:https://dx.doi.org/10.1097/YIC.0000000000000431. Exclusion reason: No psychometric information;

Erden, S., Nalbant, K., & Kilinc, I. (2022). Investigation of relaxin-3 serum levels in terms of social interaction, communication, and appetite as a biomarker in children with autism. *Clinical Psychopharmacology and Neuroscience, 20*(1), 135-142. doi:https://dx.doi.org/10.9758/cpn.2022.20.1.135. Exclusion reason: No psychometric information

Fabbri-Destro, M., Maugeri, F., Ianni, C., Corsini, S., Di Stefano, E., Scatigna, S., . . . Narzisi, A. (2022). Early Sensory Profile in Autism Spectrum Disorders Predicts Emotional and Behavioral Issues. *Journal of Personalized Medicine, 12*(10), 27. doi:https://dx.doi.org/10.3390/jpm12101593. Exclusion reason: No psychometric information

Favole, I., Davico, C., Marcotulli, D., Sodero, R., Svevi, B., Amianto, F., . . . Vitiello, B. (2023). Sleep disturbances and emotional dysregulation in young children with autism spectrum, intellectual disability, or global developmental delay. *Sleep Medicine, 105*, 45-52. doi:https://dx.doi.org/10.1016/j.sleep.2023.02.026. Exclusion reason: No psychometric information

Ferguson, E. F., Jimenez-Munoz, M., Feerst, H., & Vernon, T. W. (2022). Predictors of satisfaction with autism treatment services during COVID-19. *Journal of Autism and Developmental Disorders, 52*(8), 3686-3697. doi:https://dx.doi.org/10.1007/s10803-021-05232-0. Exclusion reason: No psychometric information;

Fombonne, E., & Zuckerman, K. E. (2022). Clinical profiles of Black and White children referred for autism diagnosis. *Journal of Autism and Developmental Disorders, 52*(3), 1120-1130. doi:https://dx.doi.org/10.1007/s10803-021-05019-3. Exclusion reason: No psychometric information

Fossum, I. N., Orm, S., Andersen, P. N., Geurts, H. M., Oie, M. G., & Skogli, E. W. (2023). Childhood executive function predicts internalizing and externalizing symptoms in emerging adults with and without autism: A 10-year longitudinal study. *Developmental Neuropsychology, 48*(3), 97-111. doi:https://dx.doi.org/10.1080/87565641.2023.2206663. Exclusion reason: No psychometric information;

Frazier, T. W., Khaliq, I., Scullin, K., Uljarevic, M., Shih, A., & Karpur, A. (2023). Development and psychometric evaluation of the Open-Source Challenging Behavior Scale (OS-CBS). *Journal of Autism and Developmental Disorders, 53*(12), 4655-4670. doi:https://dx.doi.org/10.1007/s10803-022-05750-5. Exclusion reason: Non-ASD sample

Gao, L., Wang, C., Song, X.-r., Tian, L., Qu, Z.-y., Han, Y., & Zhang, X. (2022). The sensory abnormality mediated partially the efficacy of repetitive transcranial magnetic stimulation on treating comorbid sleep disorder in autism spectrum disorder children. *Frontiers in Psychiatry Vol 12, 2022, ArtID 820598, 12*. doi:https://dx.doi.org/10.3389/fpsyt.2021.820598. Exclusion reason: No psychometric information

Geoffray, M. M., Baltazar, M., Michelon, C., Jurek, L., & Baghdadli, A. (2023). Clinical predictors of psychotropic medication prescription in children with ASD of the ELENA cohort. *Frontiers in psychiatry Frontiers Research Foundation, 14*, 1153543. doi:https://dx.doi.org/10.3389/fpsyt.2023.1153543. Exclusion reason: No psychometric information

Grasso, M., Lazzaro, G., Demaria, F., Menghini, D., & Vicari, S. (2022). The Strengths and Difficulties Questionnaire as a Valuable Screening Tool for Identifying Core Symptoms and Behavioural and Emotional Problems in Children with Neuropsychiatric Disorders. *International Journal of Environmental Research & Public Health [Electronic Resource], 19*(13), 23. doi:https://dx.doi.org/10.3390/ijerph19137731. Exclusion reason: No psychometric information;.

Graucher, T., Sinai-Gavrilov, Y., Mor, Y., Netzer, S., Cohen, E. Y., Levi, L., . . . Koller, J. (2022). From clinic room to Zoom: Delivery of an evidence-based, parent-mediated intervention in the community before and during the pandemic. *Journal of Autism and Developmental Disorders, 52*(12), 5222-5231. doi:https://dx.doi.org/10.1007/s10803-022-05592-1. Exclusion reason: No psychometric information;

Green, J., Leadbitter, K., Ellis, C., Taylor, L., Moore, H. L., Carruthers, S., . . . Pickles, A. (2022). Combined social communication therapy at home and in education for young autistic children in England (PACT-G): A parallel, single-blind, randomised controlled trial. *The Lancet Psychiatry, 9*(4), 307-320. doi:https://dx.doi.org/10.1016/S2215-0366%2822%2900029-3. Exclusion reason: Not relevant measurement tool

Guzick, A. G., Schneider, S. C., Perozo Garcia, A. B., Kook, M., Greenberg, R. L., Riddle, D., . . . Storch, E. A. (2023). Development and pilot testing of internet-delivered, family-based cognitive behavioral therapy for anxiety and obsessive-compulsive disorders in autistic youth. *Journal of Obsessive-Compulsive and Related Disorders, 37*, 1-9. doi:https://dx.doi.org/10.1016/j.jocrd.2023.100789. Exclusion reason: Sample size = < 20

Habayeb, S., Kenworthy, L., De La Torre, A., & Ratto, A. (2022). Still left behind: Fewer Black school-aged youth receive ASD diagnoses compared to White youth. *Journal of Autism and Developmental Disorders, 52*(5), 2274-2283. doi:https://dx.doi.org/10.1007/s10803-021-05118-1. Exclusion reason: No psychometric information;

Hall, C. L., Partlett, C., Valentine, A. Z., Pearcey, S., & Sayal, K. (2023). Understanding the impact of home confinement on children and young people with adhd and asd during the covid-19 pandemic. *Child Psychiatry and Human Development*, No Pagination Specified. doi:https://dx.doi.org/10.1007/s10578-022-01490-w. Exclusion reason: No psychometric information

Harkins, C., Menezes, M., Sadikova, E., & Mazurek, M. (2023). Friendship and anxiety/depression symptoms in boys with and without autism spectrum disorder. *American Journal on Intellectual and Developmental Disabilities, 128*(2), 119-133. doi:https://dx.doi.org/10.1352/1944-7558-128.2.119. Exclusion reason: No psychometric information

He, Q., Wang, Y., Liu, Z., Xia, J., Yin, H., Qiu, Z., . . . Xie, J. (2023). Analysis of salivary steroid hormones in boys with autism spectrum disorder. *BMC Psychiatry Vol 23, 2023, ArtID 105, 23*. doi:https://dx.doi.org/10.1186/s12888-023-04586-2. Exclusion reason: No psychometric information

Hermans, R. A., Storm, A. E. M., Kloosterboer, S. M., Hillegers, M. H. J., Koch, B. C. P., Dierckx, B., & de Winter, B. C. M. (2023). Therapeutic Drug Monitoring to Optimize Risperidone Treatment in Children with Autism Spectrum Disorder. *Therapeutic Drug Monitoring, 27*, 27. doi:https://dx.doi.org/10.1097/FTD.0000000000001161. Exclusion reason: No psychometric information

Holtman, S. J., Winans, K. S., & Hoch, J. D. (2022). Utility of diagnostic classification for children 0-5 to assess features of autism: Comparing in-person and COVID-19 telehealth evaluations. *Journal of Autism and Developmental Disorders, 52*(12), 5114-5125. doi:https://dx.doi.org/10.1007/s10803-022-05606-y. Exclusion reason: No psychometric information

Horwitz, E., Vos, M., De Bildt, A., Greaves-Lord, K., Rommelse, N., Schoevers, R., & Hartman, C. (2023). Sex differences in the course of autistic and co-occurring psychopathological symptoms in adolescents with and without autism spectrum disorder. *Autism, 27*(6), 1716-1729. doi:https://dx.doi.org/10.1177/13623613221146477. Exclusion reason: No psychometric information

Jasim, S., & Perry, A. (2023). Repetitive and restricted behaviors and interests in autism spectrum disorder: Relation to individual characteristics and mental health problems. *BMC Psychiatry Vol 23,(1), 2023, ArtID 356, 23*(1). doi:https://dx.doi.org/10.1186/s12888-023-04766-0. Exclusion reason: No psychometric information;

Kaur, H., Chinna, K., & Sivanesom, R. (2023). Sleep disturbances and their association with behaviour in children with autism spectrum disorder: A cross-sectional study. *Singapore Medical Journal, 14*, 14. doi:https://dx.doi.org/10.4103/singaporemedj.SMJ-2021-425. Exclusion reason: No psychometric information

Kim, B. U., Kim, H. W., Park, E. J., Kim, J. H., Boon-Yasidhi, V., Tarugsa, J., . . . Joung, Y. S. (2022). Long-Term Improvement and Safety of Aripiprazole for Irritability and Adaptive Function in Asian Children and Adolescents with Autistic Disorder: A 52-Week, Multinational, Multicenter, Open-Label Study. *Journal of Child & Adolescent Psychopharmacology, 32*(7), 390-399. doi:https://dx.doi.org/10.1089/cap.2022.0004. Exclusion reason: No psychometric information

Kim, Y. R., Song, D. Y., Bong, G., Han, J. H., Kim, J. H., & Yoo, H. J. (2023). Clinical characteristics of comorbid tic disorders in autism spectrum disorder: exploratory analysis. *Child & Adolescent Psychiatry & Mental Health [Electronic Resource], 17*(1), 71. doi:https://dx.doi.org/10.1186/s13034-023-00625-8. Exclusion reason: No psychometric information

Kong, X. J., Kang, J., & Liu, K. (2023). Probiotic and intra-nasal oxytocin combination therapy on autonomic function and gut-brain axis signaling in young children and teens with autism spectrum disorder. *Journal of Psychiatric Research, 166*, 1-9. doi:https://dx.doi.org/10.1016/j.jpsychires.2023.08.006. Exclusion reason: No psychometric information

Lau, B. K., Emmons, K. A., Lee, A. K., Munson, J., Dager, S. R., & Estes, A. M. (2023). The prevalence and developmental course of auditory processing differences in autistic children. *Autism Research, 16*(7), 1413-1424. doi:https://dx.doi.org/10.1002/aur.2961. Exclusion reason: No psychometric information

Lestarevic, S., Kalanj, M., Milutinovic, L., Grujicic, R., Vasic, J., Maslak, J., . . . Pejovic-Milovancevic, M. (2023). Internal consistency of the serbian translation of the stanford social dimensions scale and association to strengths and difficulties questionnaire scores in male and female individuals on the autism spectrum and non-autistic individuals. *Journal of Autism and Developmental Disorders*, No Pagination Specified. doi:https://dx.doi.org/10.1007/s10803-023-06061-z. Exclusion reason: No psychometric information

Li, H., Shi, B., Wang, X., Cao, M., Chen, J., Liu, S., . . . Zhu, Y. (2022). Associations of emotional/behavioral problems with accelerometer-measured sedentary behavior, physical activity and step counts in children with autism spectrum disorder. *Frontiers in Public Health, 10*, 981128. doi:https://dx.doi.org/10.3389/fpubh.2022.981128. Exclusion reason: No psychometric information

Lin, X., Lin, L., Wang, X., Li, X., Cao, M., & Jing, J. (2023). Association between Mothers' Emotional Problems and Autistic Children's Behavioral Problems: The Moderating Effect of Parenting Style. *International Journal of Environmental Research & Public Health [Electronic Resource], 20*(5), 05. doi:https://dx.doi.org/10.3390/ijerph20054593. Exclusion reason: No psychometric information

Lin, X., Su, X., Huang, S., Liu, Z., Yu, H., Wang, X., . . . Jing, J. (2023). Association between maternal parenting styles and behavioral problems in children with ASD: Moderating effect of maternal autistic traits. *Frontiers in psychiatry Frontiers Research Foundation, 14*, 1107719. doi:https://dx.doi.org/10.3389/fpsyt.2023.1107719. Exclusion reason: No psychometric information

Ma, C. H., Chen, L. Y., Li, M. F., Wu, D., Wang, S. S., Zhao, Y. J., . . . Wang, Y. (2022). Treatment of Preschool Children With Autism Spectrum Disorder: A Trial to Evaluate a Learning Style Profile Intervention Program in China. *Frontiers in Pediatrics, 10*, 831621. doi:https://dx.doi.org/10.3389/fped.2022.831621. Exclusion reason: No psychometric information

Madarevic, M., van Esch, L., Lambrechts, G., Ceulemans, E., Van Leeuwen, K., & Noens, I. (2022). Parenting behaviours among mothers of pre-schoolers on the autism spectrum: Associations with parenting stress and children's externalising behaviour problems. *Research in Autism Spectrum Disorders Vol 90, 2022, ArtID 101901, 90*. doi:https://dx.doi.org/10.1016/j.rasd.2021.101901. Exclusion reason: No psychometric information

Martinez-Cayuelas, E., Gavela-Perez, T., Rodrigo-Moreno, M., Losada-Del Pozo, R., Moreno-Vinues, B., Garces, C., & Soriano-Guillen, L. (2023). Sleep problems, circadian rhythms, and their relation to behavioral difficulties in children and adolescents with autism spectrum disorder. *Journal of Autism and Developmental Disorders*, No Pagination Specified. doi:https://dx.doi.org/10.1007/s10803-023-05934-7. Exclusion reason: No psychometric information

Martini, M. I., Merkelbach, I., & Begeer, S. (2022). Gestational Age in Autistic Children and Adolescents: Prevalence and Effects on Autism Phenotype. *Journal of Autism & Developmental Disorders, 07*, 07. doi:https://dx.doi.org/10.1007/s10803-022-05466-6. Exclusion reason: No psychometric information;

May, T., & Williams, K. (2022). Mother and child mental health over time in children with Autism and/or ADHD in the Longitudinal Study of Australian Children. *Development & Psychopathology*, 1-11. doi:https://dx.doi.org/10.1017/S0954579422001067. Exclusion reason: No psychometric information;

McLay, L. L., France, K. G., Blampied, N. M., Hunter, J. E., van Deurs, J. R., Woodford, E. C., . . . Lang, R. (2022). Collateral child and parent effects of function-based behavioral interventions for sleep problems in children and adolescents with autism. *Journal of Autism and Developmental Disorders, 52*(5), 2258-2273. doi:https://dx.doi.org/10.1007/s10803-021-05116-3. Exclusion reason: No psychometric information;

Mensi, M. M., Gasparini, L., Chiappedi, M., Guerini, F. R., Orlandi, M., Rogantini, C., & Balottin, U. (2023). How parental levels of empathy and alexithymia influence their perception of child's behavior. *Minerva Pediatrics, 75*(5), 719-726. doi:https://dx.doi.org/10.23736/S2724-5276.20.05609-1. Exclusion reason: No psychometric information

Mills, A. S., Tablon-Modica, P., Mazefksy, C. A., & Weiss, J. A. (2022). Emotion dysregulation in children with autism: A multimethod investigation of the role of child and parent factors. *Research in Autism Spectrum Disorders, 91*, 1-11. doi:https://dx.doi.org/10.1016/j.rasd.2021.101911. Exclusion reason: Not relevant measurement tool;

Molcho-Haimovich, A., Tikotzky, L., Meiri, G., Ilan, M., Michaelovski, A., Schtaierman, H., . . . Dinstein, I. (2023). Sleep disturbances are associated with irritability in ASD children with sensory sensitivities. *Journal of Neurodevelopmental Disorders Vol 15,(1), 2023, ArtID 21, 15*(1). doi:https://dx.doi.org/10.1186/s11689-023-09491-z. Exclusion reason: No psychometric information

Nakua, H., Hawco, C., Forde, N. J., Jacobs, G. R., Joseph, M., Voineskos, A. N., . . . Ameis, S. H. (2022). Cortico-amygdalar connectivity and externalizing/internalizing behavior in children with neurodevelopmental disorders. *Brain Structure & Function, 227*(6), 1963-1979. doi:https://dx.doi.org/10.1007/s00429-022-02483-0. Exclusion reason: Non-ASD sample

Nalbant, K., & Erden, S. (2023). Possible effects of N-acetylcysteine in autism spectrum disorders: major clinical aspects, eating behaviors, and sleeping habits. *Turkish Journal of Pediatrics, 65*(5), 832-844. doi:https://dx.doi.org/10.24953/turkjped.2022.573. Exclusion reason: No psychometric information

Nasiri, M., Parmoon, Z., Farahmand, Y., Moradi, A., Farahmand, K., Moradi, K., . . . Akhondzadeh, S. (2023). l-carnitine adjunct to risperidone for treatment of autism spectrum disorder-associated behaviors: a randomized, double-blind clinical trial. *International Clinical Psychopharmacology, 07*, 07. doi:https://dx.doi.org/10.1097/YIC.0000000000000496. Exclusion reason: No psychometric information;

Navarro, I. P., Martinez-Lorca, M., Criado-Alvarez, J. J., & Martinez-Lorca, A. (2022). The impact of the COVID-19 pandemic on the Spanish children and teenager population with autism spectrum disorder and in their families. *Revista de Psicologia Clinica con Ninos y Adolescentes, 9*(1), 72-81. Exclusion reason: Non-english;

Neuhaus, E., Kang, V. Y., Kresse, A., Corrigan, S., Aylward, E., Bernier, R., . . . Webb, S. J. (2022). Language and aggressive behaviors in male and female youth with autism spectrum disorder. *Journal of Autism and Developmental Disorders, 52*(1), 454-462. doi:https://dx.doi.org/10.1007/s10803-020-04773-0. Exclusion reason: No psychometric information

Nguyen, J., Zhang, B., Hanson, E., Mylonas, D., & Maski, K. (2022). Neurobehavioral Associations with NREM and REM Sleep Architecture in Children with Autism Spectrum Disorder. *Children, 9*(9), 30. doi:https://dx.doi.org/10.3390/children9091322. Exclusion reason: No psychometric information

Ni, H.-C., Chen, Y.-L., Chao, Y.-P., Wu, C.-T., Chen, R.-S., Chou, T.-L., . . . Lin, H.-Y. (2023). A lack of efficacy of continuous theta burst stimulation over the left dorsolateral prefrontal cortex in autism: A double blind randomized sham-controlled trial. *Autism Research, 16*(6), 1247-1262. doi:https://dx.doi.org/10.1002/aur.2954. Exclusion reason: No psychometric information

Oh, J., Kim, K., Kannan, K., Parsons, P. J., Mlodnicka, A., Schmidt, R. J., . . . Bennett, D. H. (2023). Early childhood exposure to environmental phenols and parabens, phthalates, organophosphate pesticides, and trace elements in association with attention deficit hyperactivity disorder (ADHD) symptoms in the CHARGE study. *Research Square, 10*, 10. doi:https://dx.doi.org/10.21203/rs.3.rs-2565914/v1. Exclusion reason: Reviews/theoretical/non-empirical/not original data

Olson, L., Chen, B., Ibarra, C., Wang, T., Mash, L., Linke, A., . . . Fishman, I. (2022). Externalizing behaviors are associated with increased parenting stress in caregivers of young children with Autism. *Journal of Autism and Developmental Disorders, 52*(3), 975-986. doi:https://dx.doi.org/10.1007/s10803-021-04995-w. Exclusion reason: No psychometric information

Oshima, F., Mandy, W., Seto, M., Hongo, M., Tsuchiyagaito, A., Hirano, Y., . . . Shimizu, E. (2023). Cognitive behavior therapy for autistic adolescents, awareness and care for my autistic traits program: A multicenter randomized controlled trial. *BMC Psychiatry Vol 23,(1), 2023, ArtID 661, 23*(1). doi:https://dx.doi.org/10.1186/s12888-023-05075-2. Exclusion reason: No psychometric information

Palmer, M., Carter Leno, V., Hallett, V., Mueller, J. M., Breese, L., Pickles, A., . . . Simonoff, E. (2023). Effects of a parenting intervention for emotional and behavioral problems in young autistic children under conditions of enhanced uncertainty: Two-year follow-up of a pilot randomized controlled trial cohort (ASTAR) during the United Kingdom COVID-19 pandemic. *Journal of the American Academy of Child & Adolescent Psychiatry, 62*(5), 558-567. doi:https://dx.doi.org/10.1016/j.jaac.2022.09.436. Exclusion reason: No psychometric information

Pan, P. Y., & Yeh, C. B. (2023). Characteristic Similarities of Irritability Between Autism and Disruptive Mood Dysregulation Disorder. *Journal of Child & Adolescent Psychopharmacology, 33*(10), 428-432. doi:https://dx.doi.org/10.1089/cap.2023.0035. Exclusion reason: No psychometric information

Pan, Z. Y., Zhong, H. J., Huang, D. N., Wu, L. H., & He, X. X. (2022). Beneficial Effects of Repeated Washed Microbiota Transplantation in Children With Autism. *Frontiers in Pediatrics, 10*, 928785. doi:https://dx.doi.org/10.3389/fped.2022.928785. Exclusion reason: No psychometric information

Pascoe, M. I., Forbes, K., de la Roche, L., Derby, B., Psaradellis, E., Anagnostou, E., . . . Kelley, E. (2023). Exploring the association between social skills struggles and social communication difficulties and depression in youth with autism spectrum disorder. *Autism Research, 16*(11), 2160-2171. doi:https://dx.doi.org/10.1002/aur.3015. Exclusion reason: No psychometric information

Pena-Salazar, C., Arrufat, F., Santos Lopez, J.-M., Fontanet, A., Roura-Poch, P., Gil-Girbau, M., . . . Serrano-Blanco, A. (2022). Intellectual disability, autism spectrum disorders, psychiatric comorbidities and their relationship with challenging behavior. *Journal of Mental Health Research in Intellectual Disabilities, 15*(2), 77-94. doi:https://dx.doi.org/10.1080/19315864.2022.2029641. Exclusion reason: Non-ASD sample

Prince, N., Chu, S. H., Chen, Y., Mendez, K. M., Hanson, E., Green-Snyder, L., . . . Kelly, R. S. (2023). Phenotypically driven subgroups of ASD display distinct metabolomic profiles. *Brain, Behavior, and Immunity, 111*, 21-29. doi:https://dx.doi.org/10.1016/j.bbi.2023.03.026. Exclusion reason: No psychometric information

Randell, E., Wright, M., Milosevic, S., Gillespie, D., Brookes-Howell, L., Busse-Morris, M., . . . McNamara, R. (2022). Sensory integration therapy for children with autism and sensory processing difficulties: the SenITA RCT. *Health Technology Assessment (Winchester, England), 26*(29), 1-140. doi:https://dx.doi.org/10.3310/TQGE0020. Exclusion reason: Not relevant measurement tool

Ratto, A. B., Reimann, G., & Nadwodny, N. (2022). Dual language learning predicts improved executive functioning in youth with autism. *Journal of Autism and Developmental Disorders, 52*(11), 5007-5017. doi:https://dx.doi.org/10.1007/s10803-021-05356-3. Exclusion reason: No psychometric information

Reder, M., & Brzezewska, K. (2022). Developmental and family environment predictors of diagnostic decision-making in children with autism spectrum disorder and comorbid disorders. *Health Psychology Report, 10*(4), 280-293. doi:https://dx.doi.org/10.5114/hpr/151836. Exclusion reason: Not relevant measurement tool

Reyes, N., Soke, G. N., Wiggins, L., Barger, B., Moody, E., Rosenberg, C., . . . Hepburn, S. (2023). Social and language regression: Characteristics of children with autism spectrum disorder in a community-based sample. *Journal of Developmental and Physical Disabilities*, No Pagination Specified. doi:https://dx.doi.org/10.1007/s10882-023-09929-1. Exclusion reason: No psychometric information

Sabzevari, F., Amelirad, O., Moradi, Z., & Habibi, M. (2023). Artificial intelligence evaluation of COVID-19 restrictions and speech therapy effects on the autistic children's behavior. *Scientific Reports, 13*(1), 4312. doi:https://dx.doi.org/10.1038/s41598-022-25902-y. Exclusion reason: No psychometric information;

Samanta, P., Panigrahi, A., Senapati, L. K., Mishra, D. P., Ravan, J. R., & Mishra, J. (2022). Maladaptive Behavior and Associated Factors among Young Children with Autism. *Indian Journal of Pediatrics, 89*(11), 1134-1136. doi:https://dx.doi.org/10.1007/s12098-022-04286-x. Exclusion reason: No psychometric information;

Schiltz, H. K., Fenning, R. M., Erath, S. A., Baucom, B. R., & Baker, J. K. (2022). Electrodermal activity moderates sleep-behavior associations in children with autism spectrum disorder. *Research on Child and Adolescent Psychopathology, 50*(6), 823-835. doi:https://dx.doi.org/10.1007/s10802-022-00900-w. Exclusion reason: Not relevant measurement tool

Schneider, S., Clement, C., Goltzene, M.-A., Meyer, N., Gras-Vincendon, A., Schroder, C. M., & Coutelle, R. (2022). Determinants of the evolutions of behaviours, school adjustment and quality of life in autistic children in an adapted school setting: An exploratory study with the International Classification of Functioning, disability and health (ICF). *BMC Psychiatry Vol 22, 2022, ArtID 323, 22*. doi:https://dx.doi.org/10.1186/s12888-022-03924-0. Exclusion reason: No psychometric information

Schulz, S. E., Kelley, E., Anagnostou, E., Nicolson, R., Georgiades, S., Crosbie, J., . . . Stevenson, R. A. (2023). Sensory processing patterns predict problem behaviours in autism spectrum disorder and attention-deficit/hyperactivity disorder. *Advances in Neurodevelopmental Disorders, 7*(1), 46-58. doi:https://dx.doi.org/10.1007/s41252-022-00269-3. Exclusion reason: No psychometric information

Seguin, D., Pac, S., Wang, J., Nicolson, R., Martinez-Trujillo, J., Anagnostou, E., . . . Duerden, E. G. (2022). Amygdala subnuclei volumes and anxiety behaviors in children and adolescents with autism spectrum disorder, attention deficit hyperactivity disorder, and obsessive-compulsive disorder. *Human Brain Mapping, 43*(16), 4805-4816. doi:https://dx.doi.org/10.1002/hbm.26005. Exclusion reason: Non-ASD sample

Shakibaei, F., & Jelvani, D. (2023). Effect of adding l-carnitine to risperidone on behavioral, cognitive, social, and physical symptoms in children and adolescents with autism: A randomized double-blinded placebo-controlled clinical trial. *Clinical Neuropharmacology, 46*(2), 55-59. doi:https://dx.doi.org/10.1097/WNF.0000000000000544. Exclusion reason: No psychometric information

Shanker, S., & Pradhan, B. (2023). Effect of yoga on the social responsiveness and problem behaviors of children with ASD in special schools: A randomized controlled trial. *Explore: The Journal of Science & Healing, 19*(4), 594-599. doi:https://dx.doi.org/10.1016/j.explore.2022.12.004. Exclusion reason: No psychometric information

Sharpley, C., & Bitsika, V. (2022). The association between self-rated social anxiety, social functioning, and eating disturbances in girls with autism spectrum disorder. *International Journal of Disability, Development and Education, 69*(2), 657-671. doi:https://dx.doi.org/10.1080/1034912X.2020.1731435. Exclusion reason: No psychometric information

Sherman, H. T., Liu, K., Kwong, K., Chan, S. T., Li, A. C., & Kong, X. J. (2022). Carbon monoxide (CO) correlates with symptom severity, autoimmunity, and responses to probiotics treatment in a cohort of children with autism spectrum disorder (ASD): a post-hoc analysis of a randomized controlled trial. *BMC Psychiatry, 22*(1), 536. doi:https://dx.doi.org/10.1186/s12888-022-04151-3. Exclusion reason: No psychometric information

Stankova, M., Kamenski, T., Ivanov, I., & Mihova, P. (2023). Emotional and Behavioral Problems of Children with ASD-The Lessons That We Learned from the Pandemic. *Children, 10*(6), 30. doi:https://dx.doi.org/10.3390/children10060969. Exclusion reason: No psychometric information;.

Staunton, E., Kehoe, C., & Sharkey, L. (2023). Families under pressure: Stress and quality of life in parents of children with an intellectual disability. *Irish Journal of Psychological Medicine, 40*(2), 192-199. doi:https://dx.doi.org/10.1017/ipm.2020.4. Exclusion reason: No psychometric information

Stephenson, K. G., Fenning, R. M., Macklin, E. A., Lu, F., Norris, M., Steinberg-Epstein, R., & Butter, E. M. (2023). Child behavior problems and parenting stress in underserved families of children with ASD: Investigation of family resources and parenting self-efficacy. *Journal of Autism and Developmental Disorders, 53*(10), 3787-3798. doi:https://dx.doi.org/10.1007/s10803-022-05681-1. Exclusion reason: No psychometric information

Tan, S., Pan, N., Xu, X., Li, H., Lin, L., Chen, J., . . . Li, X. (2022). The association between sugar-sweetened beverages and milk intake with emotional and behavioral problems in children with autism spectrum disorder. *Frontiers in Nutrition, 9*, 927212. doi:https://dx.doi.org/10.3389/fnut.2022.927212. Exclusion reason: No psychometric information

ten Hoopen, L. W., de Nijs, P. F., Duvekot, J., Greaves-Lord, K., Hillegers, M. H., Brouwer, W. B., & Hakkaart-van Roijen, L. (2022). Caring for children with an autism spectrum disorder: Factors associating with health- and care-related quality of life of the caregivers. *Journal of Autism and Developmental Disorders, 52*(11), 4665-4678. doi:https://dx.doi.org/10.1007/s10803-021-05336-7. Exclusion reason: Non-ASD sample

Tsang, B., Leung, C. N. W., & Chan, R. W. S. (2022). A feasibility study on social competence intervention for Chinese adolescents and adults with comorbid autism spectrum disorder and intellectual disability. *Journal of Applied Research in Intellectual Disabilities, 35*(5), 1131-1139. doi:https://dx.doi.org/10.1111/jar.12998. Exclusion reason: No psychometric information

Ventura, P., de Giambattista, C., Trerotoli, P., Cavone, M., Di Gioia, A., & Margari, L. (2022). Methylphenidate Use for Emotional Dysregulation in Children and Adolescents with ADHD and ADHD and ASD: A Naturalistic Study. *Journal of Clinical Medicine, 11*(10), 22. doi:https://dx.doi.org/10.3390/jcm11102922. Exclusion reason: No psychometric information

Wright, N., Courchesne, V., Pickles, A., Bedford, R., Duku, E., Kerns, C. M., . . . Elsabbagh, M. (2023). A longitudinal comparison of emotional, behavioral and attention problems in autistic and typically developing children. *Psychological Medicine, 53*(16), 7707-7719. doi:https://dx.doi.org/10.1017/S0033291723001599. Exclusion reason: No psychometric information

Yule, A. M., DiSalvo, M., Biederman, J., Wilens, T. E., Dallenbach, N. T., Taubin, D., & Joshi, G. (2023). Decreased risk for substance use disorders in individuals with high-functioning autism spectrum disorder. *European Child & Adolescent Psychiatry, 32*(2), 257-265. doi:https://dx.doi.org/10.1007/s00787-021-01852-0. Exclusion reason: No psychometric information

**Excluded references from the original search in March 2022:**

Abouzed, M., & Elsherbiny, A. (2018). Prevalence of hoarding in adolescents with high functioning autism. *European Psychiatry, 48(Supplement 1)*, S147. doi:http://dx.doi.org/10.1016/j.eurpsy.2017.12.016.
Exclusion reason: Conference abstract.

Adamek, L., Nichols, S., Tetenbaum, S. P., Bregman, J., Ponzio, C. A., & Carr, E. G. (2011). Individual temperament and problem behavior in children with autism spectrum disorders. *Focus on Autism and Other Developmental Disabilities, 26*(3), 173-183. doi:https://dx.doi.org/10.1177/1088357611405041.
Exclusion reason: No psychometric information.

Adams, H. L., Matson, J. L., & Jang, J. (2014). The relationship between sleep problems and challenging behavior among children and adolescents with autism spectrum disorder. *Research in Autism Spectrum Disorders, 8*(9), 1024-1030. doi:https://dx.doi.org/10.1016/j.rasd.2014.05.008. Exclusion reason: No psychometric information.

Adams, R. E., Fredstrom, B. K., Duncan, A. W., Holleb, L. J., & Bishop, S. L. (2014). Using Self- and parent-reports to test the association between peer victimization and internalizing symptoms in verbally fluent adolescents with ASD. *Journal of Autism and Developmental Disorders, 44(4)*, 861-872. doi:http://dx.doi.org/10.1007/s10803-013-1938-0. Exclusion reason: No psychometric information.

Aerts, C. (2011). Psychiatric Family Treatment Autism (PFA): Hope and reality for families with ASD. *European Child and Adolescent Psychiatry, 1)*, S25. doi:http://dx.doi.org/10.1007/s00787-011-0181-5. Exclusion reason: Conference abstract.

Akhavan, B., Pishyareh, E., & Robubi, H. (2012). The effect of play therapy on communication skills of autistic children. *Iranian Journal of Psychiatry, 1)*, 104. Exclusion reason: Conference abstract

Albaum, C., Tablon, P., Roudbarani, F., & Weiss, J. A. (2020). Predictors and outcomes associated with therapeutic alliance in cognitive behaviour therapy for children with autism. *Autism, 24(1)*, 211-220. doi:http://dx.doi.org/10.1177/1362361319849985. Exclusion reason: No psychometric information.

Allik, H., Larsson, J. O., & Smedje, H. (2006). Health-related quality of life in parents of school-age children with Asperger Syndrome or High-Functioning Autism. *Health & Quality of Life Outcomes, 4*, 1. Exclusion reason: No psychometric information

Allik, H., Larsson, J. O., & Smedje, H. (2006). Insomnia in school-age children with Asperger syndrome or high-functioning autism. *BMC Psychiatry, 6*, 18. Exclusion reason: No psychometric information.

Alostaz, J., Baker, J. K., Fenning, R. M., Neece, C. L., & Zeedyk, S. (2022). Parental coping as a buffer between child factors and emotion-related parenting in families of children with autism spectrum disorder. *Journal of Family Psychology, 36*(1), 153-158. doi:https://dx.doi.org/10.1037/fam0000757. Exclusion reason: No psychometric information.

Althaus, M., Minderaa, R. B., & Dienske, H. (1994). The assessment of individual differences between young children with a pervasive developmental disorder by means of behaviour scales which are derived from direct observation. *Journal of Child Psychology & Psychiatry & Allied Disciplines, 35*(2), 333-349. Exclusion reason: Not relevant measurement tool.

Ameis, S. H., Haltigan, J. D., Lyon, R. E., Sawyer, A., Mirenda, P., Kerns, C. M., . . . Pathways in, A. S. D. S. T. (2021). Middle-childhood executive functioning mediates associations between early-childhood autism symptoms and adolescent mental health, academic and functional outcomes in autistic children. *Journal of Child Psychology & Psychiatry & Allied Disciplines, 12*, 12. doi:https://dx.doi.org/10.1111/jcpp.13493. Exclusion reason: No psychometric information.

Amr, M., Bu Ali, W., Hablas, H., Raddad, D., El-Mehesh, F., El-Gilany, A. H., & Al-Shamy, H. (2012). Sociodemographic factors in Arab children with Autism Spectrum Disorders. *The Pan African Medical Journal, 13*, 65. Exclusion reason: No psychometric information.

Amr, M., Raddad, D., El-Mehesh, F., Mahmoud, E. H., & El-Gilany, A. H. (2011). Sex differences in Arab children with Autism spectrum disorders. *Research in Autism Spectrum Disorders, 5(4)*, 1343-1350. doi:http://dx.doi.org/10.1016/j.rasd.2011.01.015. Exclusion reason: No psychometric information.

Andersen, P. N., Hovik, K. T., Skogli, E. W., & Oie, M. G. (2017). Severity of autism symptoms and degree of attentional difficulties predicts emotional and behavioral problems in children with high-functioning autism. a two-year follow-up study. *Frontiers in Psychology Vol 8 2017, ArtID 2004, 8*. doi:https://dx.doi.org/10.3389/fpsyg.2017.02004. Exclusion reason: No psychometric information.

Anderson, D. K., Maye, M. P., & Lord, C. (2011). Changes in maladaptive behaviors from midchildhood to young adulthood in autism spectrum disorder. *American journal on intellectual and developmental disabilities, 116*(5), 381-397. doi:https://dx.doi.org/10.1352/1944-7558-116.5.381. Exclusion reason: No psychometric information.

Anixt, J. S., Murray, D. S., Coury, D. L., Kuhlthau, K. A., Eskra, D., Seide, J., . . . Lannon, C. (2020). Improving Behavior Challenges and Quality of Life in the Autism Learning Health Network. *Pediatrics, 145*(Suppl 1), S20-S29. doi:https://dx.doi.org/10.1542/peds.2019-1895E. Exclusion reason: No psychometric information.

Aral, A., Bozkurt, A., & Usta, M. B. (2015). Child sleeping behaviors that influence sleep problems in parents of children with autism spectrum disorder: Preliminary study. *Klinik Psikofarmakoloji Bulteni, 1)*, S104-S105. Exclusion reason: Conference abstract.

Argumedes, M., Lanovaz, M. J., & Larivee, S. (2018). Brief report: Impact of challenging behavior on parenting stress in mothers and fathers of children with autism spectrum disorders. *Journal of Autism and Developmental Disorders, 48*(7), 2585-2589. doi:https://dx.doi.org/10.1007/s10803-018-3513-1. Exclusion reason: No psychometric information.

Arias, A. A., Rea, M. M., Adler, E. J., Haendel, A. D., & Van Hecke, A. V. (2021). Utilizing the Child Behavior Checklist (CBCL) as an Autism Spectrum Disorder Preliminary Screener and Outcome Measure for the PEERS R Intervention for Autistic Adolescents. *Journal of Autism & Developmental Disorders, 30*, 30. doi:https://dx.doi.org/10.1007/s10803-021-05103-8. Exclusion reason: Effect study.

Arnold, S. R. C., Uljarevic, M., Hwang, Y. I., Richdale, A. L., Trollor, J. N., & Lawson, L. P. (2020). Brief Report: Psychometric Properties of the Patient Health Questionaire-9 (PHQ-9) in Autistic Adults. *Journal of Autism & Developmental Disorders, 50*(6), 2217-2225. doi:https://dx.doi.org/10.1007/s10803-019-03947-9. Exclusion reason: Not relevant measurement tool.

Ashburner, J., Ziviani, J., & Rodger, S. (2010). Surviving in the mainstream: Capacity of children with autism spectrum disorders to perform academically and regulate their emotions and behavior at school. *Research in Autism Spectrum Disorders, 4*(1), 18-27. doi:https://dx.doi.org/10.1016/j.rasd.2009.07.002. Exclusion reason: No psychometric information

Babinska, K., Pivovarciova, A., Filcikova, D., Tomova, A., & Ostatnikova, D. (2016). Association of conduct problems and gastrointestinal symptoms in individuals with autism spectrum disorders. *Activitas Nervosa Superior Rediviva, 58(3)*, 69-72. Exclusion reason: No psychometric information.

Backner, W., Clark, E., Jenson, W., Gardner, M., & Kahn, J. (2013). An investigation of psychiatric comorbidity and symptom awareness among male adolescents with autism spectrum disorders. *International Journal of School & Educational Psychology, 1*(4), 259-268. doi:https://dx.doi.org/10.1080/21683603.2013.845737. Exclusion reason: No psychometric information.

Bader, S. H., & Barry, T. D. (2014). A longitudinal examination of the relation between parental expressed emotion and externalizing behaviors in children and adolescents with autism spectrum disorder. *Journal of Autism and Developmental Disorders, 44(11)*, 2820-2831. doi:http://dx.doi.org/10.1007/s10803-014-2142-6. Exclusion reason: No psychometric information.

Bader, S. H., Barry, T. D., & Hann, J. A. (2015). The relation between parental expressed emotion and externalizing behaviors in children and adolescents with an autism spectrum disorder. *Focus on Autism and Other Developmental Disabilities, 30*(1), 23-34. doi:https://dx.doi.org/10.1177/1088357614523065. Exclusion reason: No psychometric information.

Baeza-Velasco, C., Michelon, C., Rattaz, C., & Baghdadli, A. (2014). Are Aberrant Behavioral patterns associated with the adaptive behavior trajectories of teenagers with Autism Spectrum Disorders? *Research in Autism Spectrum Disorders, 8(3)*, 304-311. doi:http://dx.doi.org/10.1016/j.rasd.2013.12.004. Exclusion reason: No psychometric information.

Bagaiolo, L. F., Bordini, D., da Cunha, G. R., Sasaki, T. N., Nogueira, M. L. M., Pacifico, C. R., & Braido, M. (2019). Implementing a community-based parent training behavioral intervention for Autism Spectrum Disorder. *Psicologia: Teoria e Pratica, 21*(3), 456-472. doi:https://dx.doi.org/10.5935/1980-6906/psicologia.v21n3p456-472. Exclusion reason: Effect study.

Baker, A. E., Lane, A., Angley, M. T., & Young, R. L. (2008). The relationship between sensory processing patterns and behavioural responsiveness in autistic disorder: A pilot study. *Journal of Autism and Developmental Disorders, 38*(5), 867-875. doi:https://dx.doi.org/10.1007/s10803-007-0459-0. Exclusion reason: No psychometric information.

Baker, B. L., & Blacher, J. (2020). Brief report: Behavior disorders and social skills in adolescents with autism spectrum disorder: Does IQ matter? *Journal of Autism and Developmental Disorders, 50*(6), 2226-2233. doi:https://dx.doi.org/10.1007/s10803-019-03954-w. Exclusion reason: No psychometric information.

Baker, J. K., Fenning, R. M., Howland, M. A., & Huynh, D. (2019). Parental criticism and behavior problems in children with autism spectrum disorder. *Autism, 23(5)*, 1249-1261. doi:http://dx.doi.org/10.1177/1362361318804190. Exclusion reason: No psychometric information.

Baker, J. K., Seltzer, M. M., & Greenberg, J. S. (2011). Longitudinal effects of adaptability on behavior problems and maternal depression in families of adolescents with autism. *Journal of Family Psychology, 25*(4), 601-609. doi:https://dx.doi.org/10.1037/a0024409. Exclusion reason: No psychometric information.

Bal, V. H., Leventhal, B. L., Carter, G., Kim, H., Koh, Y. J., Ha, M., . . . Kim, Y. S. (2021). Parent-Reported Suicidal Ideation in Three Population-Based Samples of School-Aged Korean Children With Autism Spectrum Disorder and Autism Spectrum Screening Questionnaire Screen Positivity. *Archives of Suicide Research*, 1-18. doi:https://dx.doi.org/10.1080/13811118.2020.1868367. Exclusion reason: No psychometric information.

Balboni, G., Bacherini, A., Rebecchini, G., Cagiano, R., Mancini, A., Tancredi, R., . . . Muratori, F. (2021). Individual and Environmental Factors Affecting Adaptive Behavior of Toddlers with Autism Spectrum Disorder: Role of Parents' Socio-cultural Level. *Journal of Autism & Developmental Disorders, 51*(10), 3469-3482. doi:https://dx.doi.org/10.1007/s10803-020-04803-x. Exclusion reason: No psychometric information.

Bangerter, A., Ness, S., Fai Ho, K., Aman, M. G., Esbensen, A., Goodwin, M. S., . . . Pandina, G. J. (2016). The autism behavior inventory: A novel tool for assessing change in core and associated symptoms of autism spectrum disorder. *Journal of the American Academy of Child and Adolescent Psychiatry, 55(10 Supplement 1)*, S109. doi:http://dx.doi.org/10.1016/j.jaac.2016.09.030. Exclusion reason: Conference abstract.

Baptista, J., Sampaio, A., Fachada, I., Osorio, A., Mesquita, A. R., Garayzabal, E., . . . Soares, I. (2019). Maternal Interactive Behaviours in Parenting Children with Williams Syndrome and Autism Spectrum Disorder: Relations with Emotional/Behavioural Problems. *Journal of Autism & Developmental Disorders, 49*(1), 216-226. doi:https://dx.doi.org/10.1007/s10803-018-3715-6. Exclusion reason: No psychometric information.

Battaglia, M., Detrick, S., & Fernandez, A. (2016). Multidisciplinary treatment for adults with autism spectrum disorder and co-occurring mental health disorders: Adapting clinical research tools to everyday clinical practice. *Journal of Mental Health Research in Intellectual Disabilities, 9*(4), 232-249. doi:https://dx.doi.org/10.1080/19315864.2016.1192708. Exclusion reason: No psychometric information.

Baudewijns, L., Ronsse, E., Verstraete, V., Sabbe, B., Morrens, M., & Bertelli, M. O. (2018). Problem behaviours and Major Depressive Disorder in adults with intellectual disability and autism. *Psychiatry Research, 270*, 769-774. doi:https://dx.doi.org/10.1016/j.psychres.2018.10.039. Exclusion reason: Not relevant measurement tool.

Bauminger, N., Solomon, M., & Rogers, S. J. (2010). Externalizing and internalizing behaviors in ASD. *Autism research : Official Journal of the International Society for Autism Research, 3*(3), 101-112. doi:https://dx.doi.org/10.1002/aur.131. Exclusion reason: Not relevant measurement tool.

Bearss, K. (2021). 3.6 Parent Training for Disruptive Behaviors in Children with Autism Spectrum Disorder. *Journal of the American Academy of Child and Adolescent Psychiatry, 60(10 Supplement)*, S126-S127. doi:http://dx.doi.org/10.1016/j.jaac.2021.07.505. Exclusion reason: Conference abstract.

Bechis, D., Baietto, C., Caldarera, A. M., & Vitiello, B. (2021). Psychopathological profile in children with Prader-Willi syndrome as compared with autism spectrum disorder. *Minerva Pediatrics, 26*, 26. doi:https://dx.doi.org/10.23736/S2724-5276.21.06447-8. Exclusion reason: No psychometric information.

Berkovits, L., Eisenhower, A., & Blacher, J. (2017). Emotion regulation in young children with autism spectrum disorders. *Journal of Autism and Developmental Disorders, 47*(1), 68-79. doi:https://dx.doi.org/10.1007/s10803-016-2922-2. Exclusion reason: No psychometric information.

Bhang, S., Kim, J., & Hwang, S. (2015). Assessing problematic behaviors in Korean children with developmental disorders. *European Child and Adolescent Psychiatry, 1)*, S180. doi:http://dx.doi.org/10.1007/s00787-015-0714-4. Exclusion reason: Conference abstract.

Bilenberg, N. (2013). Co-occurring symptoms in a mixed clinical sample of children with autism spectrum disorders. *European Child and Adolescent Psychiatry, 1)*, S208. doi:http://dx.doi.org/10.1007/s00787-013-0423-9. Exclusion reason: Conference abstract.

Bilgi, A., & Uzun, N. (2016). The association of parental psychiatric symptoms and parent-child relationships with behavioral and emotional problems and socioemotional competence in newly diagnosed young children with autism spectrum disorder. *Journal of the American Academy of Child and Adolescent Psychiatry, 55(10 Supplement 1)*, S102. doi:http://dx.doi.org/10.1016/j.jaac.2016.09.010. Exclusion reason: Conference abstract.

Bischof, N. L., Rapee, R. M., Hudry, K., & Bayer, J. K. (2018). Acceptability and caregiver-reported outcomes for young children with autism spectrum disorder whose parents attended a preventative population-based intervention for anxiety: A pilot study. *Autism Research, 11*(8), 1166-1174. doi:https://dx.doi.org/10.1002/aur.1963. Exclusion reason: No psychometric information.

Bitsika, V., Heyne, D. A., & Sharpley, C. F. (2021). Is Bullying Associated with Emerging School Refusal in Autistic Boys? *Journal of Autism & Developmental Disorders, 51*(4), 1081-1092. doi:https://dx.doi.org/10.1007/s10803-020-04610-4. Exclusion reason: No psychometric information.

Blacher, J., & Baker, B. L. (2019). Collateral effects of youth disruptive behavior disorders on mothers' psychological distress: Adolescents with Autism Spectrum Disorder, intellectual disability, or typical development. *Journal of Autism and Developmental Disorders, 49*(7), 2810-2821. Exclusion reason: No psychometric information.

Blacher, J., Howell, E., Lauderdale-Littin, S., Reed, F. D., & Laugeson, E. A. (2014). Autism spectrum disorder and the student teacher relationship: A comparison study with peers with intellectual disability and typical development. *Research in Autism Spectrum Disorders, 8*(3), 324-333. doi:https://dx.doi.org/10.1016/j.rasd.2013.12.008. Exclusion reason: No psychometric information.

Blainey, S. H., Rumball, F., Mercer, L., Evans, L. J., & Beck, A. (2017). An evaluation of the effectiveness of psychological therapy in reducing general psychological distress for adults with autism spectrum conditions and comorbid mental health problems. *Clinical psychology & psychotherapy, 24*(6), O1474-O1484. doi:https://dx.doi.org/10.1002/cpp.2108. Exclusion reason: Not relevant measurement tool.

Blank, E., Dominick, K., Shaffer, R., Erickson, C., & Lamy, M. (2021). 6.19 Outcomes of Specialized Psychiatric Inpatient Crisis Intervention for Children and Adolescents With Autism Spectrum Disorder and Other Neurodevelopmental Disorders. *Journal of the American Academy of Child and Adolescent Psychiatry, 60(10 Supplement)*, S164. doi:http://dx.doi.org/10.1016/j.jaac.2021.09.092. Exclusion reason: Conference abstract.

Bolte, S., Dickhut, H., & Poustka, F. (1999). Patterns of parent-reported problems indicative in autism. *Psychopathology, 32*(2), 93-97. Exclusion reason: No psychometric information.

Boonen, H., Maljaars, J., Lambrechts, G., Zink, I., Van Leeuwen, K., & Noens, I. (2014). Behavior problems among school-aged children with autism spectrum disorder: Associations with children's communication difficulties and parenting behaviors. *Research in Autism Spectrum Disorders, 8*(6), 716-725. doi:https://dx.doi.org/10.1016/j.rasd.2014.03.008. Exclusion reason: No psychometric information.

Bowri, M., Hull, L., Allison, C., Smith, P., Baron-Cohen, S., Lai, M. C., & Mandy, W. (2021). Demographic and psychological predictors of alcohol use and misuse in autistic adults. *Autism, 25(5)*, 1469-1480. doi:http://dx.doi.org/10.1177/1362361321992668. Exclusion reason: Not relevant measurement tool.

Bradley, E., & Bolton, P. (2006). Episodic psychiatric disorders in teenagers with learning disabilities with and without autism. *The British Journal of Psychiatry, 189*(4), 361-366. doi:https://dx.doi.org/10.1192/bjp.bp.105.018127. Exclusion reason: No psychometric information.

Bradley, E. A., Ames, C. S., & Bolton, P. F. (2011). Psychiatric Conditions and Behavioural Problems in Adolescents With Intellectual Disabilities: Correlates With Autism. *Canadian Journal of Psychiatry-Revue Canadienne De Psychiatrie, 56*(2), 102-109. doi:10.1177/070674371105600205. Exclusion reason: No psychometric information.

Bravaccio, C., Marino, M., Lanzara, V., Sperandeo, S., Ferrentino, R. I., & Riccio, M. P. (2013). Identification of specific behavioral, adaptive and cognitive pattern in a cohort of autistic patients: A study of correlation. *European Child and Adolescent Psychiatry, 1)*, S239. doi:http://dx.doi.org/10.1007/s00787-013-0423-9. Exclusion reason: Conference abstract.

Breen, J., & Hare, D. J. (2017). The nature and prevalence of catatonic symptoms in young people with autism. *Journal of Intellectual Disability Research, 61(6)*, 580-593. doi:http://dx.doi.org/10.1111/jir.12362. Exclusion reason: Not relevant measurement tool.

Brei, N. G., Schwarz, G., & Klein-Tasman, B. P. (2015). Predictors of parenting stress in children referred for an autism spectrum disorder diagnostic evaluation. *Journal of Developmental and Physical Disabilities, 27*(5), 617-635. doi:https://dx.doi.org/10.1007/s10882-015-9439-z. Exclusion reason: Sample size = < 20.

Bremer, E., & Lloyd, M. (2021). Baseline behaviour moderates movement skill intervention outcomes among young children with autism spectrum disorder. *Autism, 25(7)*, 2025-2033. doi:http://dx.doi.org/10.1177/13623613211009347. Exclusion reason: Sample size = < 20.

Brenner, J., Pan, Z., Mazefsky, C., Smith, K. A., & Gabriels, R. (2018). Behavioral symptoms of reported abuse in children and adolescents with autism spectrum disorder in inpatient settings. *Journal of Autism and Developmental Disorders, 48*(11), 3727-3735. doi:https://dx.doi.org/10.1007/s10803-017-3183-4. Exclusion reason: No psychometric information.

Brereton, A. V., Tonge, B. J., & Einfeld, S. L. (2006). Psychopathology in children and adolescents with autism compared to young people with intellectual disability. *Journal of Autism & Developmental Disorders, 36*(7), 863-870. Exclusion reason: No psychometric information.

Brewe, A. M., Mazefsky, C. A., & White, S. W. (2021). Therapeutic Alliance Formation for Adolescents and Young Adults with Autism: Relation to Treatment Outcomes and Client Characteristics. *Journal of Autism and Developmental Disorders, 51(5)*, 1446-1457. doi:http://dx.doi.org/10.1007/s10803-020-04623-z. Exclusion reason: No psychometric information.

Bridgemohan, C., Cochran, D. M., Howe, Y. J., Pawlowski, K., Zimmerman, A. W., Anderson, G. M., . . . Neumeyer, A. M. (2019). Investigating Potential Biomarkers in Autism Spectrum Disorder. *Frontiers in Integrative Neuroscience, 13*, 31. doi:https://dx.doi.org/10.3389/fnint.2019.00031. Exclusion reason: No psychometric information.

Brondino, N., Damiani, S., & Politi, P. (2020). Effective strategies for managing covid-19 emergency restrictions for adults with severe asd in a daycare center in Italy. *Brain Sciences, 10(7)*, 1-5. doi:http://dx.doi.org/10.3390/brainsci10070436. Exclusion reason: Sample size = < 20.

Brookman-Frazee, L. I., Taylor, R., & Garland, A. F. (2010). Characterizing community-based mental health services for children with autism spectrum disorders and disruptive behavior problems. *Journal of Autism and Developmental Disorders, 40*(10), 1188-1201. doi:https://dx.doi.org/10.1007/s10803-010-0976-0. Exclusion reason: Sample size = < 20.

Brooks, W., & Butter, E. M. (2012). Distinguishing high functioning ASD from ADHD in a clinical sample. *Journal of Intellectual Disability Research, 56(7-8)*, 690. doi:http://dx.doi.org/10.1111/j.1365-2788.2012.01583_4.x. Exclusion reason: Conference abstract.

Broquere, M., Soussana, M., Michelon, C., Rattaz, C., Brisot, J., & Baghdadli, A. (2016). Impact of anxiety disorders on quality of life of adolescents with autism spectrum disorder without intellectual disability. *Encephale, 42(6)*, 499-505. doi:http://dx.doi.org/10.1016/j.encep.2015.12.025. Exclusion reason: Non-English.

Bryson, S. A., Corrigan, S. K., McDonald, T. P., & Holmes, C. (2008). Characteristics of children with autism spectrum disorders who received services through community mental health centers. *Autism, 12*(1), 65-82. doi:https://dx.doi.org/10.1177/1362361307085214. Exclusion reason: Sample size = < 20.

Butera, C., Ring, P., Sideris, J., Jayashankar, A., Kilroy, E., Harrison, L., . . . Aziz-Zadeh, L. (2020). Impact of Sensory Processing on School Performance Outcomes in High Functioning Individuals with Autism Spectrum Disorder. *Mind Brain & Education, 14*(3), 243-254. doi:https://dx.doi.org/10.1111/mbe.12242. Exclusion reason: No psychometric information.

Buyuktaskin, D., Iseri, E., Guney, E., Gunendi, Z., & Cengiz, B. (2021). Somatosensory Temporal Discrimination in Autism Spectrum Disorder. *Autism research : Official Journal of the International Society for Autism Research, 14*(4), 656-667. doi:https://dx.doi.org/10.1002/aur.2479. Exclusion reason: No psychometric information.

Byars, K. C., Katz, T., Malow, B. A., Shui, A., Connolly, H. V., Carno, M., . . . Glaze, D. (2013). Sleep disordered breathing is associated with worse functioning in children with autism spectrum disorders. *Sleep, 1)*, A378. Exclusion reason: Conference abstract.

Campistol, J., Diez-Juan, M., Callejon, L., Fernandez-De Miguel, A., Casado, M., Garcia Cazorla, A., . . . Artuch, R. (2016). Inborn error metabolic screening in individuals with nonsyndromic autism spectrum disorders. *Developmental Medicine & Child Neurology, 58*(8), 842-847. doi:https://dx.doi.org/10.1111/dmcn.13114. Exclusion reason: No psychometric information.

Capal, J. K., Carosella, C., Corbin, E., Horn, P. S., Caine, R., & Manning-Courtney, P. (2018). EEG endophenotypes in autism spectrum disorder. *Epilepsy & Behavior, 88*, 341-348. doi:https://dx.doi.org/10.1016/j.yebeh.2018.09.036. Exclusion reason: No psychometric information.

Capal, J. K., Macklin, E. A., Lu, F., & Barnes, G. (2020). Factors Associated With Seizure Onset in Children With Autism Spectrum Disorder. *Pediatrics, 145*(Suppl 1), S117-S125. doi:https://dx.doi.org/10.1542/peds.2019-1895O. Exclusion reason: No psychometric information.

Carpenter, K. L. H., Lorenzi, J., Herold, B., McVea, M., Vermeer, S., Franz, L., & Dawson, G. (2019). 1.15 Relationship between Sensory Processing Differences and Clinical Features of Children with Asd. *Journal of the American Academy of Child and Adolescent Psychiatry, 58(10 Supplement)*, S151. doi:http://dx.doi.org/10.1016/j.jaac.2019.08.037. Exclusion reason: Conference abstract.

Carta, A., Fuca, E., Guerrera, S., Napoli, E., Valeri, G., & Vicari, S. (2020). Characterization of Clinical Manifestations in the Co-occurring Phenotype of Attention Deficit/Hyperactivity Disorder and Autism Spectrum Disorder. *Frontiers in Psychology, 11*, 861. doi:https://dx.doi.org/10.3389/fpsyg.2020.00861. Exclusion reason: No psychometric information.

Carter Leno, V., Wright, N., Pickles, A., Bedford, R., Zaidman-Zait, A., Kerns, C., . . . Elsabbagh, M. (2022). Exposure to family stressful life events in autistic children: Longitudinal associations with mental health and the moderating role of cognitive flexibility. *Autism.* doi:https://dx.doi.org/10.1177/13623613211061932. Exclusion reason: No psychometric information.

Carter, M., Gibson, L., & Murray, D. (2022). Characterising the temporal evolution of emotional and behavioural problems (EBP) in an Irish cohort of Autism Spectrum Disorder (ASD) affected children in early childhood. *Developmental Medicine and Child Neurology, 64(SUPPL 1)*, 50. doi:https://dx.doi.org/10.1111/dmcn.15123. Exclusion reason: Conference abstract.

Cervantes, P., Matson, J. L., Tureck, K., & Adams, H. L. (2013). The relationship of comorbid anxiety symptom severity and challenging behaviors in infants and toddlers with autism spectrum disorder. *Research in Autism Spectrum Disorders, 7(12)*, 1528-1534. doi:http://dx.doi.org/10.1016/j.rasd.2013.09.005. Exclusion reason: No psychometric information.

Cervantes, P. E., & Matson, J. L. (2015). The relationship between comorbid psychopathologies, autism, and social skill deficits in young children. *Research in Autism Spectrum Disorders, 10*, 101-108. doi:https://dx.doi.org/10.1016/j.rasd.2014.11.006. Exclusion reason: No psychometric information.

Cervantes, P. E., Matson, J. L., Adams, H. L., & Konst, M. J. (2014). The relationship between cognitive development and conduct problems in young children with autism spectrum disorder. *Research in Autism Spectrum Disorders, 8(10)*, 1287-1294. doi:http://dx.doi.org/10.1016/j.rasd.2014.06.015. Exclusion reason: No psychometric information.

Cervantes, P. E., Matson, J. L., Williams, L. W., & Jang, J. (2014). The effect of cognitive skills and autism spectrum disorder on stereotyped behaviors in infants and toddlers. *Research in Autism Spectrum Disorders, 8(5)*, 502-508. doi:http://dx.doi.org/10.1016/j.rasd.2014.01.008. Exclusion reason: No psychometric information.

Chaidez, V., Hansen, R. L., & Hertz-Picciotto, I. (2014). Gastrointestinal problems in children with autism, developmental delays or typical development. *Journal of Autism & Developmental Disorders, 44*(5), 1117-1127. doi:https://dx.doi.org/10.1007/s10803-013-1973-x. Exclusion reason: No psychometric information.

Chalfant, A. M., Rapee, R., & Carroll, L. (2007). Treating anxiety disorders in children with high functioning autism spectrum disorders: A controlled trial. *Journal of Autism and Developmental Disorders, 37*(10), 1842-1857. doi:https://dx.doi.org/10.1007/s10803-006-0318-4. Exclusion reason: Effect study.

Chan, R. W. S., Leung, C. N. W., Ng, D. C. Y., & Yau, S. S. W. (2018). Validating a Culturally-sensitive Social Competence Training Programme for Adolescents with ASD in a Chinese Context: An Initial Investigation. *Journal of Autism and Developmental Disorders, 48(2)*, 450-460. doi:http://dx.doi.org/10.1007/s10803-017-3335-6. Exclusion reason: Effect study.

Charfi, N., Halayem, S., Touati, M., Mrabet, A., & Bouden, A. (2016). Aggressive behavior risk factors in autistic spectrum disorders. *Neuropsychiatrie de l'Enfance et de l'Adolescence, 64(3)*, 147-154. doi:http://dx.doi.org/10.1016/j.neurenf.2016.02.004. Exclusion reason: Non-English.

Charman, T., Loth, E., Tillmann, J., Crawley, D., Wooldridge, C., Goyard, D., . . . Buitelaar, J. K. (2017). The EU-AIMS Longitudinal European Autism Project (LEAP): clinical characterisation. *Molecular Autism, 8*, 27. doi:https://dx.doi.org/10.1186/s13229-017-0145-9. Exclusion reason: Not relevant measurement tool.

Charman, T., Palmer, M., Stringer, D., Hallett, V., Mueller, J., Romeo, R., . . . Simonoff, E. (2021). A novel group parenting intervention for emotional and behavioral difficulties in young autistic children: Autism Spectrum Treatment and Resilience (ASTAR): A randomized controlled trial. *Journal of the American Academy of Child & Adolescent Psychiatry, 60*(11), 1404-1418. doi:https://dx.doi.org/10.1016/j.jaac.2021.03.024. Exclusion reason: No psychometric information.

Charman, T., Ricketts, J., Dockrell, J. E., Lindsay, G., & Palikara, O. (2015). Emotional and behavioural problems in children with language impairments and children with autism spectrum disorders. *International Journal of Language & Communication Disorders, 50*(1), 84-93. doi:https://dx.doi.org/10.1111/1460-6984.12116. Exclusion reason: No psychometric information.

Chen, J., Liu, Y., & Du, Y. (2018). 5.21 A Preliminary Study on Social Development and Play Behavior of Boys With ASD. *Journal of the American Academy of Child and Adolescent Psychiatry, 57(10 Supplement)*, S234. doi:http://dx.doi.org/10.1016/j.jaac.2018.09.316. Exclusion reason: Conference abstract.

Cheung, R. Y., Leung, S. S., & Mak, W. W. (2019). Role of mindful parenting, affiliate stigma, and parents' well-being in the behavioral adjustment of children with autism spectrum disorder: Testing parenting stress as a mediator. *Mindfulness, 10*(11), 2352-2362. doi:https://dx.doi.org/10.1007/s12671-019-01208-5. Exclusion reason: No psychometric information.

Chiang, H. L., & Gau, S. S. (2016). Comorbid psychiatric conditions as mediators to predict later social adjustment in youths with autism spectrum disorder. *Journal of Child Psychology & Psychiatry & Allied Disciplines, 57*(1), 103-111. doi:https://dx.doi.org/10.1111/jcpp.12450. Exclusion reason: No psychometric information.

Chiang, W.-C., Tseng, M.-H., Fu, C.-P., Chuang, I. C., Lu, L., & Shieh, J.-Y. (2019). Exploring sensory processing dysfunction, parenting stress, and problem behaviors in children with autism spectrum disorder. *American Journal of Occupational Therapy, 73*(1), 1-10. doi:https://dx.doi.org/10.5014/ajot.2019.027607. Exclusion reason: No psychometric information.

Cho, H. N., & Ha, E. H. (2019). A Validation Study of the Korean Child Behavior Checklist 1.5-5 in the Diagnosis of Autism Spectrum Disorder and Non-Autism Spectrum Disorder. *Soa!$ceongsonyeon Jeongsin Yihag, 30*(1), 9-16. doi:https://dx.doi.org/10.5765/jkacap.180018.1. Exclusion reason: No psychometric information.

Cho, I. H. (2011). Aggregation and divergence of ASD-related phenotypes in unaffected siblings of ASD. *European Child and Adolescent Psychiatry, 1)*, S79. doi:http://dx.doi.org/10.1007/s00787-011-0181-5. Exclusion reason: Conference abstract.

Choi, K. Y., & Kovshoff, H. (2013). Do maternal attributions play a role in the acceptability of behavioural interventions for problem behaviour in children with autism spectrum disorders? *Research in Autism Spectrum Disorders, 7*(8), 984-996. doi:https://dx.doi.org/10.1016/j.rasd.2013.04.010. Exclusion reason: No psychometric information.

Chowdhury, M., Aman, M. G., Lecavalier, L., Smith, T., Johnson, C., Swiezy, N., . . . Scahill, L. (2016). Factor structure and psychometric properties of the revised Home Situations Questionnaire for autism spectrum disorder: The Home Situations Questionnaire-Autism Spectrum Disorder. *Autism, 20*(5), 528-537. doi:https://dx.doi.org/10.1177/1362361315593941. Exclusion reason: No psychometric information.

Christopher, K., Bishop, S., Carpenter, L. A., Warren, Z., & Kanne, S. (2021). The Implications of Parent-Reported Emotional and Behavioral Problems on the Modified Checklist for Autism in Toddlers. *Journal of Autism & Developmental Disorders, 51*(3), 884-891. doi:https://dx.doi.org/10.1007/s10803-020-04469-5. Exclusion reason: No psychometric information.

Chung, K.-M., & Jung, D. (2018). "Validity and reliability of the Korean version of autism spectrum disorders-comorbid for children (ASD-CC)": Corrigendum. *Research in Autism Spectrum Disorders, 45*, 42. doi:https://dx.doi.org/10.1016/j.rasd.2017.10.004. Exclusion reason: Not relevant measurement tool.

Clark, M., Barbaro, J., & Dissanayake, C. (2020). Parent and teacher ratings of social skills, peer play and problem behaviours in children with autism spectrum disorder. *International Journal of Disability, Development and Education, 67*(2), 194-207. doi:https://dx.doi.org/10.1080/1034912X.2019.1662891. Exclusion reason: Not relevant measurement tool.

Coales, C., Heaney, N., Ricketts, J., Dockrell, J. E., Lindsay, G., Palikara, O., & Charman, T. (2019). Health-related quality of life in children with autism spectrum disorders and children with developmental language disorders. *Autism & Developmental Language Impairments Vol 4 2019, ArtID 2396941519851225, 4*. doi:https://dx.doi.org/10.1177/2396941519851225. Exclusion reason: No psychometric information.

Cohen, I. L., Tsiouris, J. A., Flory, M. J., Kim, S. Y., Freedland, R., Heaney, G., . . . Brown, W. T. (2010). A large scale study of the psychometric characteristics of the IBR Modified Overt Aggression Scale: findings and evidence for increased self-destructive behaviors in adult females with autism spectrum disorder. *Journal of Autism & Developmental Disorders, 40*(5), 599-609. doi:https://dx.doi.org/10.1007/s10803-009-0908-z. Exclusion reason: Not relevant measurement tool.

Colvert, E., Simonoff, E., Capp, S. J., Ronald, A., Bolton, P., & Happe, F. (2022). Autism Spectrum Disorder and Mental Health Problems: Patterns of Difficulties and Longitudinal Trajectories in a Population-Based Twin Sample. *Journal of Autism & Developmental Disorders, 52*(3), 1077-1091. doi:https://dx.doi.org/10.1007/s10803-021-05006-8. Exclusion reason: No psychometric information.

Conner, C. M., Maddox, B. B., & White, S. W. (2013). Parents' state and trait anxiety: Relationships with anxiety severity and treatment response in adolescents with autism spectrum disorders. *Journal of Autism and Developmental Disorders, 43*(8), 1811-1818. doi:https://dx.doi.org/10.1007/s10803-012-1728-0. Exclusion reason: No psychometric information.

Conner, C. M., White, S. W., Scahill, L., & Mazefsky, C. A. (2020). The role of emotion regulation and core autism symptoms in the experience of anxiety in autism. *Autism, 24(4)*, 931-940. doi:http://dx.doi.org/10.1177/1362361320904217. Exclusion reason: Not relevant measurement tool.

Cooper, K., Smith, L. G., & Russell, A. (2017). Social identity, self-esteem, and mental health in autism. *European Journal of Social Psychology, 47*(7), 844-854. doi:https://dx.doi.org/10.1002/ejsp.2297. Exclusion reason: Not relevant measurement tool.

Copeland, W. E., Simonoff, E., & Stringaris, A. (2016). Disruptive mood dysregulation disorder in children with autism spectrum disorder. *Journal of the American Academy of Child and Adolescent Psychiatry, 55(10 Supplement 1)*, S269-S270. doi:http://dx.doi.org/10.1016/j.jaac.2016.07.164. Exclusion reason: Conference abstract.

Cost, K. T., Bennett, T. A., Duku, E., Georgiades, S., Smith, I. M., Ungar, W. J., . . . Szatmari, P. (2018). 5.32 Quantifying a Qualitative Variable in ASD: What Exactly is a "Good" Outcome? *Journal of the American Academy of Child and Adolescent Psychiatry, 57(10 Supplement)*, S236-S237. doi:http://dx.doi.org/10.1016/j.jaac.2018.09.325. Exclusion reason: Conference abstract.

Cost, K. T., Zaidman-Zait, A., Mirenda, P., Duku, E., Zwaigenbaum, L., Smith, I. M., . . . Vaillancourt, T. (2021). "Best Things": Parents Describe Their Children with Autism Spectrum Disorder Over Time. *Journal of Autism & Developmental Disorders, 51*(12), 4560-4574. doi:https://dx.doi.org/10.1007/s10803-021-04890-4. Exclusion reason: No psychometric information.

Cost, K. T., Zaidman-Zait, A., Mirenda, P., Duku, E., Zwaigenbaum, L., Smith, I. M., . . . Vaillancourt, T. (2021). "Best things": Parents describe their children with autism spectrum disorder over time": Correction. *Journal of Autism and Developmental Disorders, 51*(12), 4575-4576. doi:https://dx.doi.org/10.1007/s10803-021-05144-z. Exclusion reason: No psychometric information.

Coughlan, B., Woolgar, M., van, I. M. H., & Duschinsky, R. (2021). Socioemotional profiles of autism spectrum disorders, attention deficit hyperactivity disorder, and disinhibited and reactive attachment disorders: a symptom comparison and network approach. *Development & Psychopathology*, 1-10. doi:https://dx.doi.org/10.1017/S0954579421000882. Exclusion reason: No psychometric information.

Courtemanche, A. B., Black, W. R., & Meyer, J. S. (2021). Hair cortisol and self-injurious behavior among children with autism spectrum disorder. *American journal on intellectual and developmental disabilities, 126*(2), 158-166. doi:https://dx.doi.org/10.1352/1944-7558-126.2.158. Exclusion reason: No psychometric information.

Craig, F., De Giacomo, A., Operto, F. F., Margari, M., Trabacca, A., & Margari, L. (2019). Association between feeding/mealtime behavior problems and internalizing/externalizing problems in autism spectrum disorder (ASD), other neurodevelopmental disorders (NDDs) and typically developing children. *Minerva Pediatrica, 13*, 13. doi:https://dx.doi.org/10.23736/S0026-4946.19.05371-4. Exclusion reason: No psychometric information.

Craig, F., Lamanna, A. L., Margari, F., Matera, E., Simone, M., & Margari, L. (2015). Overlap Between Autism Spectrum Disorders and Attention Deficit Hyperactivity Disorder: Searching for Distinctive/Common Clinical Features. *Autism research : Official Journal of the International Society for Autism Research, 8*(3), 328-337. doi:https://dx.doi.org/10.1002/aur.1449. Exclusion reason: No psychometric information.

Cresswell, L., & Cage, E. (2019). 'Who am I?': An exploratory study of the relationships between identity, acculturation and mental health in autistic adolescents. *Journal of Autism and Developmental Disorders, 49*(7), 2901-2912. doi:https://dx.doi.org/10.1007/s10803-019-04016-x. Exclusion reason: No psychometric information.

Crippa, A., Colombo, P., De Cosmi, V., Mazzocchi, A., Scaglioni, S., Spolidoro, G. C. I., . . . Agostoni, C. (2022). Understanding feeding problems in autistic children: Exploring the interplay between internalizing symptoms and sensory features. *Autism.* doi:https://dx.doi.org/10.1177/13623613221080227. Exclusion reason: No psychometric information.

Cuccaro, M. L., Hamilton, K. L., Gilbert, J. R., Pericak-Vance, M. A., & Tuchmann, R. F. (2010). Emprirically defining autism-epilepsy phenotypes. *Annals of Neurology, 14)*, S108. doi:http://dx.doi.org/10.1002/ana.22201. Exclusion reason: Conference abstract.

Curran, L. K., Newschaffer, C. J., Lee, L. C., Crawford, S. O., Johnston, M. V., & Zimmerman, A. W. (2007). Behaviors associated with fever in children with autism spectrum disorders. *Pediatrics, 120*(6), e1386-1392. Exclusion reason: Non-ASD sample.

Caamano, M., Boada, L., Merchan-Naranjo, J., Moreno, C., Llorente, C., Moreno, D., . . . Parellada, M. (2013). Psychopathology in children and adolescents with ASD without mental retardation. *Journal of Autism and Developmental Disorders, 43*(10), 2442-2449. doi:https://dx.doi.org/10.1007/s10803-013-1792-0. Exclusion reason: No psychometric information.

Dababnah, S., Olson, E. M., & Nichols, H. M. (2019). Feasibility of the incredible years parent program for preschool children on the autism spectrum in two U.S. Sites. *Research in Autism Spectrum Disorders, 57*, 120-131. doi:https://dx.doi.org/10.1016/j.rasd.2018.10.010. Exclusion reason: Effect study.

Dagdelen, F. (2020). Comparison of social cognition in adolescents diagnosed with attention deficit hyperactivity disorder and autism spectrum disorder. *Dusunen Adam, 33(3)*, 289-295. doi:https://dx.doi.org/10.14744/DAJPNS.2020.00093. Exclusion reason: No psychometric information.

Dagdelen, F. (2021). Decreased theory of mind abilities and increased emotional dysregulation in adolescents with asd and adhd. *Anadolu Psikiyatri Dergisi, 22(2)*, 100-105. doi:http://dx.doi.org/10.5455/apd.135050. Exclusion reason: No psychometric information.

Damiani, S., Leali, P., Nosari, G., Caviglia, M., Puci, M. V., Monti, M. C., . . . Politi, P. (2020). Association of autism onset, epilepsy, and behavior in a community of adults with autism and severe intellectual disability. *Brain Sciences, 10(8)*, 1-7. doi:http://dx.doi.org/10.3390/brainsci10080486. Exclusion reason: No psychometric information.

David Lohr, W., Daniels, K., Wiemken, T., Gail Williams, P., Kelley, R. R., Kuravackel, G., & Sears, L. (2017). The screen for Child Anxiety-Related Emotional Disorders is sensitive but not specific in identifying anxiety in children with high-functioning autism spectrum disorder: A pilot comparison to the achenbach system of empirically based assessment scales. *Frontiers in Psychiatry, 8(AUG) (no pagination)*(138). doi:http://dx.doi.org/10.3389/fpsyt.2017.00138. Exclusion reason: Not relevant measurement tool.

Davis, A. L., & Neece, C. L. (2017). An examination of specific child behavior problems as predictors of parenting stress among families of children with pervasive developmental disorders. *Journal of Mental Health Research in Intellectual Disabilities, 10*(3), 163-177. doi:https://dx.doi.org/10.1080/19315864.2016.1276988. Exclusion reason: No psychometric information.

de Bruin, E. I., de Nijs, P. F., Verheij, F., Hartman, C. A., & Ferdinand, R. F. (2007). Multiple complex developmental disorder delineated from PDD-NOS. *Journal of Autism & Developmental Disorders, 37*(6), 1181-1191. Exclusion reason: No psychometric information.

Delhey, L., Kilinc, E. N., Yin, L., Slattery, J., Tippett, M., Wynne, R., . . . Frye, R. E. (2017). Bioenergetic variation is related to autism symptomatology. *Metabolic Brain Disease, 32*(6), 2021-2031. doi:https://dx.doi.org/10.1007/s11011-017-0087-0. Exclusion reason: No psychometric information.

D'Elia, L., Valeri, G., Sonnino, F., Fontana, I., Mammone, A., & Vicari, S. (2014). A longitudinal study of the TEACCH program in different settings: The potential benefits of low intensity intervention in preschool children with autism spectrum disorder. *Journal of Autism and Developmental Disorders, 44*(3), 615-626. doi:https://dx.doi.org/10.1007/s10803-013-1911-y. Exclusion reason: Sample size = < 20.

Dellapiazza, F., Michelon, C., Oreve, M.-J., Robel, L., Schoenberger, M., Chatel, C., . . . Baghdadli, A. (2020). The impact of atypical sensory processing on adaptive functioning and maladaptive behaviors in autism spectrum disorder during childhood: Results from the ELENA cohort. *Journal of Autism and Developmental Disorders, 50*(6), 2142-2152. doi:https://dx.doi.org/10.1007/s10803-019-03970-w. Exclusion reason: No psychometric information.

Dell'Osso, L., Carpita, B., Muti, D., Morelli, V., Salarpi, G., Salerni, A., . . . Maj, M. (2019). Mood symptoms and suicidality across the autism spectrum. *Comprehensive Psychiatry, 91*, 34-38. doi:https://dx.doi.org/10.1016/j.comppsych.2019.03.004. Exclusion reason: No psychometric information.

Demark, J. L., Feldman, M. A., & Holden, J. J. (2003). Behavioral relationship between autism and fragile x syndrome. *American Journal of Mental Retardation, 108*(5), 314-326. Exclusion reason: No psychometric information.

Demirci, E., Guler, Y., Ozmen, S., Canpolat, M., & Kumandas, S. (2019). Levels of Salivary Sialic Acid in Children with Autism Spectrum Disorder. Could It Be Related to Stereotypes and Hyperactivity? *Clinical Psychopharmacology & Neuroscience, 17*(3), 415-422. doi:https://dx.doi.org/10.9758/cpn.2019.17.3.415. Exclusion reason: No psychometric information.

Dijkxhoorn, Y., & Wulms, G. (2019). Autism spectrum disorder, intellectual disability and challenging behavior: A true challenge. *Journal of Intellectual Disability Research, 63(7)*, 657. doi:http://dx.doi.org/10.1111/jir.12652. Exclusion reason: Conference abstract.

Dimitropoulos, A., Ho, A. Y., Klaiman, C., Koenig, K., & Schultz, R. T. (2009). A comparison of behavioral and emotional characteristics in children with autism, Prader-Willi syndrome, and Williams syndrome. *Journal of Mental Health Research in Intellectual Disabilities, 2*(3), 220-243. doi:https://dx.doi.org/10.1080/19315860903052204. Exclusion reason: No psychometric information.

Dominick, K. C., Davis, N. O., Lainhart, J., Tager-Flusberg, H., & Folstein, S. (2007). Atypical behaviors in children with autism and children with a history of language impairment. *Research in Developmental Disabilities, 28*(2), 145-162. doi:https://dx.doi.org/10.1016/j.ridd.2006.02.003. Exclusion reason: Non-ASD sample.

Dovey, T. M., Kumari, V., Blissett, J., & Mealtime Hostage Parent Science, G. (2019). Eating behaviour, behavioural problems and sensory profiles of children with avoidant/restrictive food intake disorder (ARFID), autistic spectrum disorders or picky eating: Same or different? *European Psychiatry: the Journal of the Association of European Psychiatrists, 61*, 56-62. doi:https://dx.doi.org/10.1016/j.eurpsy.2019.06.008. Exclusion reason: No psychometric information.

Dovgan, K. N., & Mazurek, M. O. (2019). Relations among activity participation, friendship, and internalizing problems in children with autism spectrum disorder. *Autism, 23(3)*, 750-758. doi:http://dx.doi.org/10.1177/1362361318775541. Exclusion reason: No psychometric information.

Duane, D. D., Stadel, A., Tokyuama, M., & Rayes, D. (2014). A retrospective multidimensional comparison of referred DSM-IV autism and asperger prepubertal children: Do they qualify under DSM-5? *Journal of Neuropsychiatry and Clinical Neurosciences, 26(2)*, 14-15. doi:http://dx.doi.org/10.1176/appi.neuropsych.262. Exclusion reason: Conference abstract.

Duncan, A., Liddle, M., & Adams, R. (2021). A cluster analysis of daily living skills in school aged children with autism spectrum disorder. *International Journal of Developmental Disabilities.* doi:http://dx.doi.org/10.1080/20473869.2021.1980350. Exclusion reason: No psychometric information.

Dunn, K., Rydzewska, E., Fleming, M., & Cooper, S. A. (2020). Prevalence of mental health conditions, sensory impairments and physical disability in people with co-occurring intellectual disabilities and autism compared with other people: a cross-sectional total population study in Scotland. *BMJ Open, 10*(4), e035280. doi:https://dx.doi.org/10.1136/bmjopen-2019-035280. Exclusion reason: No psychometric information.

Dy, M., Ballantyne, A., & Trauner, D. (2014). Behavioral profiles of children and adolescents with specific language impairment and high functioning autism. *Neurology. Conference: 66th American Academy of Neurology Annual Meeting, AAN, 82*(10 SUPPL. 1). Exclusion reason: Conference abstract.

Ehrenreich-May, J., Storch, E. A., Queen, A. H., Rodriguez, J. H., Ghilain, C. S., Alessandri, M., . . . Wood, J. J. (2014). An open trial of cognitive-behavioral therapy for anxiety disorders in adolescents with autism spectrum disorders. *Focus on Autism and Other Developmental Disabilities, 29*(3), 145-155. doi:https://dx.doi.org/10.1177/1088357614533381. Exclusion reason: Sample size = < 20.

Eisenhower, A., Baker, B., & Blacher, J. (2005). Preschool children with intellectual disability: Syndrome specificity, behaviour problems, and maternal well-being. *Journal of Intellectual Disability Research, 49*(9), 657-671. doi:https://dx.doi.org/10.1111/j.1365-2788.2005.00699.x. Exclusion reason: Sample size = < 20.

Ekinci, O., Arman, A. R., Isik, U., Bez, Y., & Berkem, M. (2010). EEG abnormalities and epilepsy in autistic spectrum disorders: clinical and familial correlates. *Epilepsy & Behavior, 17*(2), 178-182. doi:https://dx.doi.org/10.1016/j.yebeh.2009.11.014. Exclusion reason: No psychometric information.

Emerson, E., Blacher, J., Einfeld, S., Hatton, C., Robertson, J., & Stancliffe, R. J. (2014). Environmental risk factors associated with the persistence of conduct difficulties in children with intellectual disabilities and autistic spectrum disorders. *Research in Developmental Disabilities, 35*(12), 3508-3517. doi:https://dx.doi.org/10.1016/j.ridd.2014.08.039. Exclusion reason: No psychometric information.

Erermis, S., Ozturk, O., Ozbaran, B., Kose, S., Demiral, N., Kucukkose, M., . . . Tamar, M. (2012). The clinical and demographic related factors with QoL in the children with autism spectrum disorders. *Neuropsychiatrie de l'Enfance et de l'Adolescence, 1)*, S211. doi:http://dx.doi.org/10.1016/j.neurenf.2012.04.447. Exclusion reason: Conference abstract.

Estes, A., Munson, J., Dawson, G., Koehler, E., Zhou, X.-H., & Abbott, R. (2009). Parenting stress and psychological functioning among mothers of preschool children with autism and developmental delay. *Autism, 13*(4), 375-387. doi:https://dx.doi.org/10.1177/1362361309105658. Exclusion reason: No psychometric information.

Estes, A., Munson, J., Rogers, S. J., Greenson, J., Winter, J., & Dawson, G. (2015). Long-term outcomes of early intervention in 6-year-old children with autism spectrum disorder. *Journal of the American Academy of Child & Adolescent Psychiatry, 54*(7), 580-587. doi:https://dx.doi.org/10.1016/j.jaac.2015.04.005. Exclusion reason: No psychometric information.

Estes, A., Rivera, V., Bryan, M., Cali, P., & Dawson, G. (2011). Discrepancies between academic achievement and intellectual ability in higher-functioning school-aged children with autism spectrum disorder. *Journal of Autism and Developmental Disorders, 41*(8), 1044-1052. doi:https://dx.doi.org/10.1007/s10803-010-1127-3. Exclusion reason: No psychometric information.

Estes, A., Shaw, D. W., Sparks, B. F., Friedman, S., Giedd, J. N., Dawson, G., . . . Dager, S. R. (2011). Basal ganglia morphometry and repetitive behavior in young children with autism spectrum disorder. *Autism research : Official Journal of the International Society for Autism Research, 4*(3), 212-220. doi:https://dx.doi.org/10.1002/aur.193. Exclusion reason: No psychometric information.

Esteves, J., Perry, A., Spiegel, R., & Weiss, J. A. (2021). Occurrence and predictors of challenging behavior in youth with intellectual disability with or without autism. *Journal of Mental Health Research in Intellectual Disabilities, 14*(2), 189-201. doi:https://dx.doi.org/10.1080/19315864.2021.1874577. Exclusion reason: No psychometric information.

Ezell, J., Shui, A., Sanders, K., & Veenstra-VanderWeele, J. (2016). Pattern of Diagnosis and Co-occurring Symptoms in Adopted Children With Autism Spectrum Disorder. *Pediatrics, 137 Suppl 2*, S90-97. doi:https://dx.doi.org/10.1542/peds.2015-2851G. Exclusion reason: No psychometric information.

Factor, R. S., Swain, D. M., Antezana, L., Muskett, A., Gatto, A. J., Radtke, S. R., & Scarpa, A. (2019). Teaching emotion regulation to children with autism spectrum disorder: Outcomes of the Stress and Anger Management Program (STAMP). *Bulletin of the Menninger Clinic, 83*(3), 235-258. doi:https://dx.doi.org/10.1521/bumc.2019.83.3.235. Exclusion reason: Effect study.

Fadini, C. C., Lamonica, D. A., Fett-Conte, A. C., Osorio, E., Zuculo, G. M., Giacheti, C. M., & Pinato, L. (2015). Influence of sleep disorders on the behavior of individuals with autism spectrum disorder. *Frontiers in Human Neuroscience Vol 9 2015, ArtID 347, 9*. doi:https://dx.doi.org/10.3389/fnhum.2015.00347. Exclusion reason: No psychometric information.

Falk, N. H., Norris, K., & Quinn, M. G. (2014). The factors predicting stress, anxiety and depression in the parents of children with autism. *Journal of Autism and Developmental Disorders, 44*(12), 3185-3203. doi:https://dx.doi.org/10.1007/s10803-014-2189-4. Exclusion reason: No psychometric information.

Feldman, J. I., Cassidy, M., Liu, Y., Kirby, A. V., Wallace, M. T., & Woynaroski, T. G. (2020). Relations between sensory responsiveness and features of autism in children. *Brain Sciences, 10(11)*, 1-15. doi:http://dx.doi.org/10.3390/brainsci10110775. Exclusion reason: No psychometric information.

Fenning, R. M., Erath, S. A., Baker, J. K., Messinger, D. S., Moffitt, J., Baucom, B. R., & Kaeppler, A. K. (2019). Sympathetic-Parasympathetic Interaction and Externalizing Problems in Children with Autism Spectrum Disorder. *Autism Research, 12*(12), 1805-1816. doi:10.1002/aur.2187. Exclusion reason: No psychometric information.

Ferguson, B. J., Dovgan, K., Takahashi, N., & Beversdorf, D. Q. (2019). The relationship among gastrointestinal symptoms, problem behaviors, and internalizing symptoms in children and adolescents with autism spectrum disorder. *Frontiers in Psychiatry Vol 10 2019, ArtID 194, 10*. doi:https://dx.doi.org/10.3389/fpsyt.2019.00194. Exclusion reason: No psychometric information.

Ferguson, B. J., Marler, S., Altstein, L. L., Lee, E. B., Akers, J., Sohl, K., . . . Beversdorf, D. Q. (2017). Psychophysiological associations with gastrointestinal symptomatology in autism spectrum disorder. *Autism Research, 10*(2), 276-288. doi:https://dx.doi.org/10.1002/aur.1646. Exclusion reason: No psychometric information.

Fernandez-Prieto, M., Moreira, C., Cruz, S., Campos, V., Martinez-Regueiro, R., Taboada, M., . . . Sampaio, A. (2021). Executive Functioning: A Mediator Between Sensory Processing and Behaviour in Autism Spectrum Disorder. *Journal of Autism & Developmental Disorders, 51*(6), 2091-2103. doi:https://dx.doi.org/10.1007/s10803-020-04648-4. Exclusion reason: No psychometric information.

Fidan, T., Asfuroglu, B., & Yanardag, M. (2019). The effect of structured pyhsical activity on sleep and mental health in severe autistic children. *Archives of Disease in Childhood, 104(Supplement 3)*, A116-A117. doi:http://dx.doi.org/10.1136/archdischild-2019-epa.272. Exclusion reason: Conference abstract.

Fink, E., Olthof, T., Goossens, F., van der Meijden, S., & Begeer, S. (2018). Bullying-related behaviour in adolescents with autism: Links with autism severity and emotional and behavioural problems. *Autism, 22*(6), 684-692. doi:https://dx.doi.org/10.1177/1362361316686760. Exclusion reason: No psychometric information.

Flenik, T. M. N., Bara, T. S., & Cordeiro, M. L. (2022). Family Functioning and Emotional Aspects of Children with Autism Spectrum Disorder in Southern Brazil. *Journal of Autism & Developmental Disorders, 17*, 17. doi:https://dx.doi.org/10.1007/s10803-022-05497-z. Exclusion reason: No psychometric information.

Flouri, E., Midouhas, E., Charman, T., & Sarmadi, Z. (2015). Poverty and the growth of emotional and conduct problems in children with autism with and without comorbid ADHD. *Journal of Autism and Developmental Disorders, 45*(9), 2928-2938. doi:https://dx.doi.org/10.1007/s10803-015-2456-z. Exclusion reason: Not relevant measurement tool

Fodstad, J. C., Rojahn, J., & Matson, J. L. (2010). Emergent comorbidity in at risk children with and without autism spectrum disorder-A cross-sectional study. *Journal of Developmental and Physical Disabilities, 22*(4), 381-400. doi:https://dx.doi.org/10.1007/s10882-010-9202-4. Exclusion reason: No psychometric information.

Fodstad, J. C., Rojahn, J., & Matson, J. L. (2012). The emergence of challenging behaviors in at-risk toddlers with and without autism spectrum disorder: A cross-sectional study. *Journal of Developmental and Physical Disabilities, 24*(3), 217-234. doi:https://dx.doi.org/10.1007/s10882-011-9266-9. Exclusion reason: No psychometric information.

Fombonne, E., Croen, L. A., Bulkley, J. E., Varga, A. M., Daida, Y. G., Hatch, B. A., . . . Lynch, F. L. (2021). Emotional and Behavioral Problems in Youth with Autism: High Prevalence and Impact on Functioning. *Journal of Developmental & Behavioral Pediatrics, 21*, 21. doi:https://dx.doi.org/10.1097/DBP.0000000000001028. Exclusion reason: Not relevant measurement tool.

Frazier, T. W., Georgiades, S., Bishop, S. L., & Hardan, A. Y. (2014). Behavioral and cognitive characteristics of females and males with autism in the Simons Simplex Collection. *Journal of the American Academy of Child & Adolescent Psychiatry, 53*(3), 329-340.e321. doi:https://dx.doi.org/10.1016/j.jaac.2013.12.004. Exclusion reason: No psychometric information.

Fulceri, F., Morelli, M., Santocchi, E., Cena, H., Del Bianco, T., Narzisi, A., . . . Muratori, F. (2016). Gastrointestinal symptoms and behavioral problems in preschoolers with Autism Spectrum Disorder. *Digestive and Liver Disease, 48(3)*, 248-254. doi:http://dx.doi.org/10.1016/j.dld.2015.11.026. Exclusion reason: No psychometric information.

Gabriels, R. L., Agnew, J. A., Holt, K. D., Shoffner, A., Zhaoxing, P., Ruzzano, S., . . . Mesibov, G. (2012). Pilot study measuring the effects of therapeutic horseback riding on school-age children and adolescents with autism spectrum disorders. *Research in Autism Spectrum Disorders, 6*(2), 578-588. doi:https://dx.doi.org/10.1016/j.rasd.2011.09.007. Exclusion reason: Effect study.

Gabriels, R. L., Pan, Z., Dechant, B., Agnew, J. A., Brim, N., & Mesibov, G. (2015). Randomized controlled trial of therapeutic horseback riding in children and adolescents with autism spectrum disorder. *Journal of the American Academy of Child & Adolescent Psychiatry, 54*(7), 541-549. doi:https://dx.doi.org/10.1016/j.jaac.2015.04.007. Exclusion reason: Effect study.

Gadke, D. L., McKinney, C., & Oliveros, A. (2016). Autism Spectrum Disorder Symptoms and Comorbidity in Emerging Adults. *Child Psychiatry & Human Development, 47*(2), 194-201. doi:https://dx.doi.org/10.1007/s10578-015-0556-9. Exclusion reason: Non-ASD sample.

Gadow, K. D. (2012). Schizophrenia spectrum and attention-deficit/hyperactivity disorder symptoms in autism spectrum disorder and controls. *Journal of the American Academy of Child and Adolescent Psychiatry, 51(10)*, 1076-1084. doi:http://dx.doi.org/10.1016/j.jaac.2012.08.001. Exclusion reason: No psychometric information.

Gadow, K. D. (2013). Association of schizophrenia spectrum and autism spectrum disorder (ASD) symptoms in children with ASD and clinic controls. *Research in Developmental Disabilities, 34*(4), 1289-1299. doi:https://dx.doi.org/10.1016/j.ridd.2013.01.011. Exclusion reason: No psychometric information.

Gadow, K. D., DeVincent, C., & Schneider, J. (2008). Predictors of psychiatric symptoms in children with an autism spectrum disorder. *Journal of Autism and Developmental Disorders, 38*(9), 1710-1720. doi:https://dx.doi.org/10.1007/s10803-008-0556-8. Exclusion reason: No psychometric information.

Gadow, K. D., Devincent, C. J., & Drabick, D. A. (2008). Oppositional defiant disorder as a clinical phenotype in children with autism spectrum disorder. *Journal of Autism & Developmental Disorders, 38*(7), 1302-1310. doi:https://dx.doi.org/10.1007/s10803-007-0516-8. Exclusion reason: No psychometric information.

Gadow, K. D., Devincent, C. J., Pomeroy, J., & Azizian, A. (2005). Comparison of DSM-IV symptoms in elementary school-age children with PDD versus clinic and community samples. *Autism, 9*(4), 392-415. Exclusion reason: No psychometric information.

Gadow, K. D., & Garman, H. D. (2020). Social anhedonia in children and adolescents with autism spectrum disorder and psychiatry referrals. *Journal of Clinical Child and Adolescent Psychology, 49*(2), 239-250. doi:https://dx.doi.org/10.1080/15374416.2018.1514611. Exclusion reason: No psychometric information.

Gadow, K. D., Perlman, G., Ramdhany, L., & Ruiter, J. (2016). Clinical correlates of co-occurring psychiatric and autism spectrum disorder (ASD) symptom-induced impairment in children with ASD. *Journal of Abnormal Child Psychology, 44*(1), 129-139. doi:https://dx.doi.org/10.1007/s10802-015-9979-9. Exclusion reason: No psychometric information.

Gadow, K. D., Perlman, G., & Weber, R. J. (2017). Parent-reported developmental regression in autism: Epilepsy, IQ, schizophrenia spectrum symptoms, and special education. *Journal of Autism and Developmental Disorders, 47*(4), 918-926. doi:https://dx.doi.org/10.1007/s10803-016-3004-1. Exclusion reason: No psychometric information.

Galligan, M. L., Heyman, M., Bolourian, Y., Stavropoulos, K., & Blacher, J. (2021). Brief Report: Emotional and Behavioral Problems Among Young Children with ASD: An Exploratory Study of ADOS E-Codes and Child Characteristics. *Journal of Autism & Developmental Disorders, 19*, 19. doi:https://dx.doi.org/10.1007/s10803-021-05313-0. Exclusion reason: Not relevant measurement tool.

Garcia-Villamisar, D., & Rojahn, J. (2015). Comorbid psychopathology and stress mediate the relationship between autistic traits and repetitive behaviours in adults with autism. *Journal of Intellectual Disability Research, 59*(2), 116-124. doi:https://dx.doi.org/10.1111/jir.12083. Exclusion reason: No psychometric information.

Gardiner, E., & Iarocci, G. (2018). Everyday executive function predicts adaptive and internalizing behavior among children with and without autism spectrum disorder. *Autism Research, 11(2)*, 284-295. doi:http://dx.doi.org/10.1002/aur.1877. Exclusion reason: No psychometric information.

Gardiner, E., Miller, A. R., & Lach, L. M. (2020). Topography of behavior problems among children with neurodevelopmental conditions: Profile differences and overlaps. *Child: Care, Health & Development, 46*(1), 149-153. doi:https://dx.doi.org/10.1111/cch.12720. Exclusion reason: No psychometric information.

Gau, S. S.-F., Chou, M.-C., Lee, J.-C., Wong, C.-C., Chou, W.-J., Chen, M.-F., . . . Wu, Y.-Y. (2010). Behavioral problems and parenting style among Taiwanese children with autism and their siblings. *Psychiatry and Clinical Neurosciences, 64*(1), 70-78. doi:https://dx.doi.org/10.1111/j.1440-1819.2009.02034.x. Exclusion reason: No psychometric information.

Georgiades, S., Szatmari, P., Duku, E., Zwaigenbaum, L., Bryson, S., Roberts, W., . . . Thompson, A. (2011). Phenotypic overlap between core diagnostic features and emotional/behavioral problems in preschool children with autism spectrum disorder. *Journal of Autism and Developmental Disorders, 41*(10), 1321-1329. doi:https://dx.doi.org/10.1007/s10803-010-1158-9. Exclusion reason: No psychometric information.

Geranmayeh, A., Holding, B. C., Lundmark, I., Fondberg, R., & Axelsson, J. (2018). Sleep and daytime functioning in children with autism. *Journal of Sleep Research, 27(Supplement 1)*, 346. doi:http://dx.doi.org/10.1111/jsr.12751. Exclusion reason: Conference abstract.

Gerber, F., Baud, M. A., Giroud, M., & Galli Carminati, G. (2008). Quality of life of adults with pervasive developmental disorders and intellectual disabilities. *Journal of Autism & Developmental Disorders, 38*(9), 1654-1665. doi:https://dx.doi.org/10.1007/s10803-008-0547-9. Exclusion reason: No psychometric information.

Gerber, F., Bessero, S., Robbiani, B., Courvoisier, D. S., Baud, M. A., Traore, M. C., . . . Galli Carminati, G. (2011). Comparing residential programmes for adults with autism spectrum disorders and intellectual disability: outcomes of challenging behaviour and quality of life. *Journal of Intellectual Disability Research, 55*(9), 918-932. doi:https://dx.doi.org/10.1111/j.1365-2788.2011.01455.x. Exclusion reason: Sample size = < 20.

Gillberg, I., Helles, A., Billstedt, E., & Gillberg, C. (2016). Boys with Asperger syndrome grow up: Psychiatric and neurodevelopmental disorders 20 years after initial diagnosis. *Journal of Autism and Developmental Disorders, 46*(1), 74-82. doi:https://dx.doi.org/10.1007/s10803-015-2544-0. Exclusion reason: No psychometric information.

Gladstone, T. R., Kaushal, S. A., Bertschinger, E. J., Tudor, M. E., & Sukhodolsky, D. G. (2017). Irritability in children with autism referred for treatment of anxiety or aggression. *Journal of the American Academy of Child and Adolescent Psychiatry, 56(10)*, S258. doi:http://dx.doi.org/10.1016/j.jaac.2017.09.298. Exclusion reason: Conference abstract.

Gobrial, E. (2019). Comorbid mental health disorders in children and young people with intellectual disabilities and autism spectrum disorders. *Advances in Mental Health and Intellectual Disabilities, 13*(5), 173-181. doi:https://dx.doi.org/10.1108/AMHID-05-2018-0026. Exclusion reason: Non-ASD sample.

Gobrial, E., & Raghavan, R. (2012). Prevalence of anxiety disorder in children and young people with intellectual disabilities and autism. *Advances in Mental Health and Intellectual Disabilities, 6*(3), 130-140. doi:https://dx.doi.org/10.1108/20441281211227193. Exclusion reason: Not relevant measurement tool.

Goldin, R. L., Matson, J. L., & Cervantes, P. E. (2014). The effect of intellectual disability on the presence of comorbid symptoms in children and adolescents with autism spectrum disorder. *Research in Autism Spectrum Disorders, 8(11)*, 1552-1556. doi:http://dx.doi.org/10.1016/j.rasd.2014.08.006. Exclusion reason: No psychometric information.

Goldin, R. L., Matson, J. L., Konst, M. J., & Adams, H. L. (2014). A comparison of children and adolescents with ASD, atypical development, and typical development on the Behavioral Assessment System for Children, Second Edition (BASC-2). *Research in Autism Spectrum Disorders, 8*(8), 951-957. doi:https://dx.doi.org/10.1016/j.rasd.2014.04.005. Exclusion reason: No psychometric information.

Goldin, R. L., Matson, J. L., Tureck, K., Cervantes, P. E., & Jang, J. (2013). A comparison of tantrum behavior profiles in children with ASD, ADHD and comorbid ASD and ADHD. *Research in Developmental Disabilities, 34*(9), 2669-2675. doi:https://dx.doi.org/10.1016/j.ridd.2013.04.022. Exclusion reason: No psychometric information.

Gotham, K., Brunwasser, S. M., & Lord, C. (2015). Depressive and anxiety symptom trajectories from school age through young adulthood in samples with autism spectrum disorder and developmental delay. *Journal of the American Academy of Child & Adolescent Psychiatry, 54*(5), 369-376.e363. doi:https://dx.doi.org/10.1016/j.jaac.2015.02.005. Exclusion reason: No psychometric information.

Goto, A., Miyawaki, D., Kusaka, H., Okada, Y., Asada, N., Iwakura, Y., . . . Inoue, K. (2015). High Prevalence of Non-psychotic Delusions in Children with High-functioning Pervasive Develonmental Disorder. *Osaka City Medical Journal, 61*(2), 73-80. Exclusion reason: No psychometric information.

Gray, K., Keating, C., Taffe, J., Brereton, A., Einfeld, S., & Tonge, B. (2012). Trajectory of behavior and emotional problems in autism. *American journal on intellectual and developmental disabilities, 117*(2), 121-133. doi:https://dx.doi.org/10.1352/1944-7588-117-2.121. Exclusion reason: No psychometric information.

Gray, K. M., Keating, C. M., Taffe, J. R., Brereton, A. V., Einfeld, S. L., Reardon, T. C., & Tonge, B. J. (2014). Adult outcomes in autism: Community inclusion and living skills. *Journal of Autism and Developmental Disorders, 44*(12), 3006-3015. doi:https://dx.doi.org/10.1007/s10803-014-2159-x. Exclusion reason: No psychometric information.

Gray, K. M., & Tonge, B. J. (2005). Screening for autism in infants and preschool children with developmental delay. *Australian & New Zealand Journal of Psychiatry, 39*(5), 378-386. Exclusion reason: No psychometric information.

Griffiths, D. L., Farrell, L. J., Waters, A. M., & White, S. W. (2017). Clinical correlates of obsessive compulsive disorder and comorbid autism spectrum disorder in youth. *Journal of Obsessive-Compulsive and Related Disorders, 14*, 90-98. doi:https://dx.doi.org/10.1016/j.jocrd.2017.06.006. Exclusion reason: Non-ASD sample.

Guerrera, S., Menghini, D., Napoli, E., Di Vara, S., Valeri, G., & Vicari, S. (2019). Assessment of Psychopathological Comorbidities in Children and Adolescents With Autism Spectrum Disorder Using the Child Behavior Checklist. *Frontiers in psychiatry Frontiers Research Foundation, 10*, 535. doi:https://dx.doi.org/10.3389/fpsyt.2019.00535. Exclusion reason: No psychometric information.

Gulsrud, A., Lin, C. E., Park, M. N., Hellemann, G., & McCracken, J. (2018). Self-injurious behaviours in children and adults with autism spectrum disorder (ASD). *Journal of Intellectual Disability Research, 62*(12), 1030-1042. doi:https://dx.doi.org/10.1111/jir.12490. Exclusion reason: No psychometric information.

Gunes, S., Ekinci, O., & Celik, T. (2017). Iron deficiency parameters in autism spectrum disorder: clinical correlates and associated factors. *Italian Journal of Pediatrics, 43*(1), 86. doi:https://dx.doi.org/10.1186/s13052-017-0407-3. Exclusion reason: No psychometric information.

Gunes, S., Ekinci, O., Feyzioglu, A., Ekinci, N., & Kalinli, M. (2019). Sleep problems in children with autism spectrum disorder: clinical correlates and the impact of attention deficit hyperactivity disorder. *Neuropsychiatric Disease & Treatment, 15*, 763-771. doi:https://dx.doi.org/10.2147/NDT.S195738. Exclusion reason: No psychometric information.

Haem, E., Doostfatemeh, M., Firouzabadi, N., Ghazanfari, N., & Karlsson, M. O. (2020). A longitudinal item response model for Aberrant Behavior Checklist (ABC) data from children with autism. *Journal of Pharmacokinetics & Pharmacodynamics, 47*(3), 241-253. doi:https://dx.doi.org/10.1007/s10928-020-09686-0. Exclusion reason: Pharmacological study.

Haggerty, D. K., Strakovsky, R. S., Talge, N. M., Carignan, C. C., Glazier-Essalmi, A. N., Ingersoll, B. R., . . . Ruden, D. M. (2021). Prenatal phthalate exposures and autism spectrum disorder symptoms in low-risk children. *Neurotoxicology & Teratology, 83*, 106947. doi:https://dx.doi.org/10.1016/j.ntt.2021.106947. Exclusion reason: No psychometric information.

Halayem, S., Charfi, N., Belhaj, A., & Bouden, A. (2015). Functional analysis of aggressive behaviors in autism spectrum disorders. *European Child and Adolescent Psychiatry, 1)*, S200. doi:http://dx.doi.org/10.1007/s00787-015-0714-4. Exclusion reason: Conference abstract.

Halayem, S., Charfi, N., Touati, M., Mrabet, A., & Bouden, A. (2018). Sensitivity to pain in autistic spectrum disorders: Its links with self-gressivity. *Tunisie Medicale, 96*(8-9), 501-504. Exclusion reason: Non-english.

Hallett, V., Ronald, A., Colvert, E., Ames, C., Woodhouse, E., Lietz, S., . . . Happe, F. (2013). Exploring anxiety symptoms in a large-scale twin study of children with autism spectrum disorders, their co-twins and controls. *Journal of Child Psychology & Psychiatry & Allied Disciplines, 54*(11), 1176-1185. Exclusion reason: Not relevant measurement tool.

Halvorsen, M. B., Aman, M. G., Martinussen, M., & Helverschou, S. B. (2021). Psychometric properties of the psychopathology in autism checklist in a neuro-pediatric sample: A pilot study. *Journal of Intellectual Disability Research, 65(8)*, 753. doi:http://dx.doi.org/10.1111/jir.12869. Exclusion reason: Conference abstract.

Han, G., Munson, J., Elder, L., Dawson, G., Dager, S., King, B., & Estes, A. (2012). Patterns and predictors of medication use in children and adolescents with autism spectrum disorder. *Journal of Investigative Medicine, 60(1)*, 220-221. doi:http://dx.doi.org/10.231/JIM.0b013e318240c940. Exclusion reason: No psychometric information.

Handen, B. L., Mazefsky, C. A., Gabriels, R. L., Pedersen, K. A., Wallace, M., & Siegel, M. (2018). Risk factors for self-injurious behavior in an inpatient psychiatric sample of children with autism spectrum disorder: A naturalistic observation study. *Journal of Autism and Developmental Disorders, 48*(11), 3678-3688. doi:https://dx.doi.org/10.1007/s10803-017-3460-2. Exclusion reason: No psychometric information.

Happe, F. G., Mansour, H., Barrett, P., Brown, T., Abbott, P., & Charlton, R. A. (2016). Demographic and Cognitive Profile of Individuals Seeking a Diagnosis of Autism Spectrum Disorder in Adulthood. *Journal of Autism & Developmental Disorders, 46*(11), 3469-3480. Exclusion reason: Not relevant measurement tool.

Hartini, S., Hapsara, S., Herini, S. E., & Takada, S. (2015). Usefulness of the CBCL to evaluate emotional and behavioral problems in Indonesian ASD children. *No To Hattatsu, 47(Supplement 1)*, S214. doi:http://dx.doi.org/10.11251/ojjscn.47.S110. Exclusion reason: Conference abstract.

Hartini, S., Sunartini, Herini, E. S., & Takada, S. (2016). Usefulness of CBCL/6-18 to evaluate emotional and behavioral problems in Indonesian autism spectrum disorder children. *Pediatrics International, 58*(12), 1307-1310. doi:https://dx.doi.org/10.1111/ped.13085. Exclusion reason: No psychometric information.

Hartley, S. L., & Sikora, D. M. (2009). Sex differences in autism spectrum disorder: an examination of developmental functioning, autistic symptoms, and coexisting behavior problems in toddlers. *Journal of Autism & Developmental Disorders, 39*(12), 1715-1722. doi:https://dx.doi.org/10.1007/s10803-009-0810-8. Exclusion reason: No psychometric information.

Hartley, S. L., Sikora, D. M., & McCoy, R. (2008). Prevalence and risk factors of maladaptive behaviour in young children with Autistic Disorder. *Journal of Intellectual Disability Research, 52*(10), 819-829. doi:https://dx.doi.org/10.1111/j.1365-2788.2008.01065.x. Exclusion reason: No psychometric information.

Hartley-McAndrew, M., & Weinstock, A. (2010). Autism Spectrum Disorder: Correlation between aberrant behaviors, EEG abnormalities and seizures. *Neurology International, 2*(1), e10. doi:https://dx.doi.org/10.4081/ni.2010.e10. Exclusion reason: No psychometric information.

Hastings, R., & Petalas, M. (2014). Self-reported behaviour problems and sibling relationship quality by siblings of children with autism spectrum disorder. *Child: care, health and development, 40*(6), 833-839. doi:https://dx.doi.org/10.1111/cch.12131. Exclusion reason: No psychometric information.

Hastings, R. P., Petalas, M. A., Jones, L., & Totsika, V. (2014). Systems analysis of associations over time between maternal and sibling well-being and behavioral and emotional problems of children with autism. *Research in Autism Spectrum Disorders, 8*(11), 1516-1520. doi:https://dx.doi.org/10.1016/j.rasd.2014.07.012. Exclusion reason: No psychometric information

Hattier, M. A., Matson, J. L., Belva, B. C., & Horovitz, M. (2011). The occurrence of challenging behaviours in children with autism spectrum disorders and atypical development. *Developmental neurorehabilitation, 14*(4), 221-229. doi:https://dx.doi.org/10.3109/17518423.2011.573836. Exclusion reason: No psychometric information.

Havdahl, K. A., Hus Bal, V., Huerta, M., Pickles, A., Oyen, A.-S., Stoltenberg, C., . . . Bishop, S. L. (2016). Multidimensional influences on autism symptom measures: Implications for use in etiological research. *Journal of the American Academy of Child & Adolescent Psychiatry, 55*(12), 1054-1063. doi:https://dx.doi.org/10.1016/j.jaac.2016.09.490. Exclusion reason: No psychometric information.

Hedgecock, J. B., Dannemiller, L. A., Shui, A. M., Rapport, M. J., & Katz, T. (2018). Associations of Gross Motor Delay, Behavior, and Quality of Life in Young Children With Autism Spectrum Disorder. *Physical Therapy, 98*(4), 251-259. doi:https://dx.doi.org/10.1093/ptj/pzy006. Exclusion reason: No psychometric information.

Heinrichs, N., Kamp-Becker, I., Bussing, R., Schimek, M., Becker, A., & Briegel, W. (2019). Disruptive behaviors across different disorders: Evaluation of a clinical sample using the eyberg child behavior inventory+. *Zeitschrift fur Kinder- und Jugendpsychiatrie und Psychotherapie, 47(1)*, 35-47. doi:http://dx.doi.org/10.1024/1422-4917/a000601. Exclusion reason: Non-ASD sample.

Helland, W. A., & Helland, T. (2017). Emotional and behavioural needs in children with specific language impairment and in children with autism spectrum disorder: The importance of pragmatic language impairment. *Research in Developmental Disabilities, 70*, 33-39. doi:https://dx.doi.org/10.1016/j.ridd.2017.08.009. Exclusion reason: No psychometric information.

Helverschou, S. B., Bakken, T. L., Berge, H., Bjorgen, T. G., Botheim, H., Hellerud, J. A., . . . Howlin, P. (2021). Preliminary findings from a nationwide, multicenter mental health service for adults and older adolescents with autism spectrum disorder and ID. *Journal of Policy and Practice in Intellectual Disabilities, 18*(2), 162-173. doi:https://dx.doi.org/10.1111/jppi.12366. Exclusion reason: No psychometric information.

Helverschou, S. B., Ludvigsen, L. B., Hove, O., & Kildahl, A. N. (2021). Psychometric properties of the psychopathology in autism checklist (PAC) in adolescences and adults. *Journal of Intellectual Disability Research, 65(8)*, 712. doi:http://dx.doi.org/10.1111/jir.12869. Exclusion reason: Conference abstract.

Hemdi, A., & Daley, D. (2017). The effectiveness of a psychoeducation intervention delivered via whatsapp for mothers of children with autism spectrum disorder (ASD) in the Kingdom of Saudi Arabia: A randomized controlled trial. *Child: care, health and development, 43*(6), 933-941. doi:https://dx.doi.org/10.1111/cch.12520. Exclusion reason: Not relevant measurement tool.

Henderson, J. A., Barry, T. D., Bader, S. H., & Jordan, S. S. (2011). The relation among sleep, routines, and externalizing behavior in children with an autism spectrum disorder. *Research in Autism Spectrum Disorders, 5*(2), 758-767. doi:https://dx.doi.org/10.1016/j.rasd.2010.09.003. Exclusion reason: Not relevant measurement tool.

Herguner, S., & Motavalli, N. (2009). Psychiatric comorbidity in children and adolescents with high functioning Autism. *European Psychiatry, 1)*, S795. Exclusion reason: Non-English.

Herring, S., Gray, K. M., Taffe, J., Tonge, B., Sweeney, D., & Einfeld, S. (2006). Behaviour and emotional problems in toddlers with pervasive developmental disorders and developmental delay: Associations with parental mental health and family functioning. *Journal of Intellectual Disability Research, 50(12)*, 874-882. doi:http://dx.doi.org/10.1111/j.1365-2788.2006.00904.x. Exclusion reason: No psychometric information.

Hess, J. A., Matson, J. L., & Dixon, D. R. (2010). Psychiatric symptom endorsements in children and adolescents diagnosed with Autism Spectrum Disorders: A comparison to typically developing children and adolescents. *Journal of Developmental and Physical Disabilities, 22*(5), 485-496. doi:https://dx.doi.org/10.1007/s10882-009-9185-1. Exclusion reason: No psychometric information.

Hesselmark, E., Eriksson, J. M., Westerlund, J., & Bejerot, S. (2015). Autism Spectrum Disorders and Self-reports: Testing Validity and Reliability Using the NEO-PI-R. *Journal of Autism & Developmental Disorders, 45*(5), 1156-1166. doi:https://dx.doi.org/10.1007/s10803-014-2275-7. Exclusion reason: Non-ASD sample.

Heuer, L., Ashwood, P., Schauer, J., Goines, P., Krakowiak, P., Hertz-Picciotto, I., . . . Van de Water, J. (2008). Reduced levels of immunoglobulin in children with autism correlates with behavioral symptoms. *Autism research : Official Journal of the International Society for Autism Research, 1*(5), 275-283. doi:https://dx.doi.org/10.1002/aur.42. Exclusion reason: No psychometric information.

Hill, A. P., Zuckerman, K. E., Hagen, A. D., Kriz, D. J., Duvall, S. W., van Santen, J., . . . Fombonne, E. (2014). Aggressive Behavior Problems in Children with Autism Spectrum Disorders: Prevalence and Correlates in a Large Clinical Sample. *Research in Autism Spectrum Disorders, 8*(9), 1121-1133. Exclusion reason: No psychometric information.

Hill, J., & Furniss, F. (2006). Patterns of emotional and behavioural disturbance associated with autistic traits in young people with severe intellectual disabilities and challenging behaviours. *Research in Developmental Disabilities, 27*(5), 517-528. Exclusion reason: Non-ASD sample.

Hillier, A., Goldstein, J., Murphy, D., Trietsch, R., Keeves, J., Mendes, E., & Queenan, A. (2018). Supporting university students with autism spectrum disorder. *Autism, 22*(1), 20-28. doi:https://dx.doi.org/10.1177/1362361317699584. Exclusion reason: Not relevant measurement tool.

Hirata, I., Mohri, I., Kato-Nishimura, K., Tachibana, M., Kuwada, A., Kagitani-Shimono, K., . . . Taniike, M. (2016). Sleep problems are more frequent and associated with problematic behaviors in preschoolers with autism spectrum disorder. *Research in Developmental Disabilities, 49-50*, 86-99. doi:https://dx.doi.org/10.1016/j.ridd.2015.11.002. Exclusion reason: No psychometric information.

Hiroshi, Y., Keiko, Y., Hisako, I., & Shigenobu, K. (2012). Multi-axial evaluation of attention deficit/hyperactivity disorder in children with autism spectrum disorder in child psychiatry clinic. *Neuropsychiatrie de l'Enfance et de l'Adolescence, 1)*, S272. doi:http://dx.doi.org/10.1016/j.neurenf.2012.04.726. Exclusion reason: Conference abstract.

Hirota, T., Deserno, M., & McElroy, E. (2020). The Network Structure of Irritability and Aggression in Individuals with Autism Spectrum Disorder. *Journal of Autism & Developmental Disorders, 50*(4), 1210-1220. doi:https://dx.doi.org/10.1007/s10803-019-04354-w. Exclusion reason: No psychometric information.

Hoch, J. D., & Youssef, A. M. (2020). Predictors of Trauma Exposure and Trauma Diagnoses for Children with Autism and Developmental Disorders Served in a Community Mental Health Clinic. *Journal of Autism & Developmental Disorders, 50*(2), 634-649. doi:https://dx.doi.org/10.1007/s10803-019-04331-3. Exclusion reason: Non-ASD sample.

Hoffmann, W., Weber, L., Konig, U., Becker, K., & Kamp-Becker, I. (2016). The role of the CBCL in the assessment of autism spectrum disorders: An evaluation of symptom profiles and screening characteristics. *Research in Autism Spectrum Disorders, 27*, 44-53. doi:http://dx.doi.org/10.1016/j.rasd.2016.04.002. Exclusion reason: No psychometric information.

Hofvander, B., Bering, S., Tarnhall, A., Wallinius, M., & Billstedt, E. (2019). Few Differences in the Externalizing and Criminal History of Young Violent Offenders With and Without Autism Spectrum Disorders. *Frontiers in Psychiatry, 10*, 8. doi:10.3389/fpsyt.2019.00911. Exclusion reason: No psychometric information.

Hofvander, B., Delorme, R., Chaste, P., Nyden, A., Wentz, E., Stahlberg, O., . . . Leboyer, M. (2009). Psychiatric and psychosocial problems in adults with normal-intelligence autism spectrum disorders. *BMC Psychiatry, 9*, 35. doi:https://dx.doi.org/10.1186/1471-244X-9-35. Exclusion reason: No psychometric information.

Hollocks, M. J., Jones, C. R., Pickles, A., Baird, G., Happe, F., Charman, T., & Simonoff, E. (2014). The association between social cognition and executive functioning and symptoms of anxiety and depression in adolescents with autism spectrum disorders. *Autism Research, 7*(2), 216-228. doi:https://dx.doi.org/10.1002/aur.1361. Exclusion reason: No psychometric information.

Holtmann, M., Bolte, S., & Poustka, F. (2007). Attention deficit hyperactivity disorder symptoms in pervasive developmental disorders: association with autistic behavior domains and coexisting psychopathology. *Psychopathology, 40*(3), 172-177. Exclusion reason: No psychometric information.

Holtmann, M., Bolte, S., & Poustka, F. (2007). Autism spectrum disorders: sex differences in autistic behaviour domains and coexisting psychopathology. *Developmental Medicine & Child Neurology, 49*(5), 361-366. Exclusion reason: No psychometric information.

Horiuchi, F., Hasegawa, F., Kawabe, K., Oka, Y., & Ueno, S. (2012). Comparison of clinical features of preschool and school-aged children with autism spectrum disorders. *Neuropsychiatrie de l'Enfance et de l'Adolescence, 1)*, S205. doi:http://dx.doi.org/10.1016/j.neurenf.2012.04.419. Exclusion reason: Conference abstract.

Horiuchi, F., Oka, Y., Uno, H., Kawabe, K., Okada, F., Saito, I., . . . Ueno, S. (2014). Age- and sex-related emotional and behavioral problems in children with autism spectrum disorders: comparison with control children. *Psychiatry & Clinical Neurosciences, 68*(7), 542-550. doi:https://dx.doi.org/10.1111/pcn.12164. Exclusion reason: No psychometric information.

Horiuchi, F., Oka, Y., Uno, H., Kawabe, K., Okada, F., Saito, I., . . . Ueno, S. I. (2014). Age- and sex-related emotional and behavioral problems in children with autism spectrum disorders: Comparison with control children. *Psychiatry and Clinical Neurosciences, 68(7)*, 542-550. doi:http://dx.doi.org/10.1111/pcn.12164. Exclusion reason: No psychometric information.

Horovitz, M., Matson, J. L., Hattier, M. A., Tureck, K., & Bamburg, J. W. (2013). Challenging behaviors in adults with intellectual disability: The effects of race and autism spectrum disorders. *Journal of Mental Health Research in Intellectual Disabilities, 6*(1), 1-13. doi:https://dx.doi.org/10.1080/19315864.2011.605989. Exclusion reason: No psychometric information.

Horovitz, M., Matson, J. L., Rieske, R. D., Kozlowski, A. M., & Sipes, M. (2011). The relationship between race and challenging behaviours in infants and toddlers with autistic disorder and pervasive developmental disordernot otherwise specified. *Developmental neurorehabilitation, 14(4)*, 208-214. doi:http://dx.doi.org/10.3109/17518423.2011.566596. Exclusion reason: Publication withdrawn

Horovitz, M., Matson, J. L., Rieske, R. D., Kozlowski, A. M., & Sipes, M. (2011). The relationship between race and challenging behaviours in infants and toddlers with autistic disorder and pervasive developmental disorder-not otherwise specified. *Developmental neurorehabilitation, 14*(4), 208-214. doi:https://dx.doi.org/10.3109/17518423.2011.566596. Exclusion reason: No psychometric information.

Horovitz, M., Matson, J. L., & Sipes, M. (2011). Gender differences in symptoms of comorbidity in toddlers with ASD using the BISCUIT-Part 2. *Developmental neurorehabilitation, 14*(2), 94-100. doi:https://dx.doi.org/10.3109/17518423.2010.546825. Exclusion reason: No psychometric information.

Horowitz, L. M., Thurm, A., Farmer, C., Mazefsky, C., Lanzillo, E., Bridge, J. A., . . . Siegel, M. (2018). Talking about death or suicide: Prevalence and clinical correlates in youth with autism spectrum disorder in the psychiatric inpatient setting. *Journal of Autism and Developmental Disorders, 48*(11), 3702-3710. doi:https://dx.doi.org/10.1007/s10803-017-3180-7. Exclusion reason: No psychometric information.

Horwitz, E. H., Schoevers, R. A., Greaves-Lord, K., de Bildt, A., & Hartman, C. A. (2020). Adult Manifestation of Milder Forms of Autism Spectrum Disorder. Autistic and Non-autistic Psychopathology. *Journal of Autism & Developmental Disorders, 50*(8), 2973-2986. doi:https://dx.doi.org/10.1007/s10803-020-04403-9. Exclusion reason: No psychometric information.

Hou, Y.-M., Stewart, L., Iao, L.-S., & Wu, C.-C. (2018). Parenting stress and depressive symptoms in Taiwanese mothers of young children with autism spectrum disorder: Association with children's behavioural problems. *Journal of Applied Research in Intellectual Disabilities, 31*(6), 1113-1121. doi:https://dx.doi.org/10.1111/jar.12471. Exclusion reason: No psychometric information.

Hsiao, M. N., Tai, Y. M., Wu, Y. Y., Tsai, W. C., Chiu, Y. N., & Gau, S. S. (2022). Psychopathologies mediate the link between autism spectrum disorder and bullying involvement: A follow-up study. *Journal of the Formosan Medical Association, 27*, 27. doi:https://dx.doi.org/10.1016/j.jfma.2021.12.030. Exclusion reason: No psychometric information.

Hu, H.-F., Liu, T.-L., Hsiao, R. C., Ni, H.-C., Liang, S. H.-Y., Lin, C.-F., . . . Yen, C.-F. (2019). Cyberbullying victimization and perpetration in adolescents with high-functioning autism spectrum disorder: Correlations with depression, anxiety, and suicidality. *Journal of Autism and Developmental Disorders, 49*(10), 4170-4180. doi:https://dx.doi.org/10.1007/s10803-019-04060-7. Exclusion reason: Not relevant measurement tool.

Huang, C. Y., Yen, H. C., Tseng, M. H., Tung, L. C., Chen, Y. D., & Chen, K. L. (2014). Impacts of autistic behaviors, emotional and behavioral problems on parenting stress in caregivers of children with autism. *Journal of Autism & Developmental Disorders, 44*(6), 1383-1390. doi:https://dx.doi.org/10.1007/s10803-013-2000-y. Exclusion reason: No psychometric information.

Hunsche, M. C., Saqui, S., Mirenda, P., Zaidman-Zait, A., Bennett, T., Duku, E., . . . Kerns, C. M. (2020). Parent-Reported Rates and Clinical Correlates of Suicidality in Children with Autism Spectrum Disorder: A Longitudinal Study. *Journal of Autism & Developmental Disorders, 50*(10), 3496-3509. doi:https://dx.doi.org/10.1007/s10803-020-04373-y. Exclusion reason: No psychometric information.

Hwang, S., Kim, Y. S., Koh, Y.-J., & Leventhal, B. L. (2018). Autism spectrum disorder and school bullying: Who is the victim? Who is the perpetrator? *Journal of Autism and Developmental Disorders, 48*(1), 225-238. doi:https://dx.doi.org/10.1007/s10803-017-3285-z. Exclusion reason: No psychometric information.

Hwang, S. S., Park, J., Kim, S., Lee, G., Kim, Y., & Bhang, S. Y. (2018). Validity and Reliability of Korean Version of Behavior Problems Inventory in Autism Spectrum Disorder and/or Intellectual Developmental Disorder. *Soa!$ceongsonyeon Jeongsin Yihag, 29*(1), 7-13. doi:https://dx.doi.org/10.5765/jkacap.2018.29.1.7. Exclusion reason: Non-ASD sample.

Iizuka, C., Yamashita, Y., Nagamitsu, S., Yamashita, T., Araki, Y., Ohya, T., . . . Matsuishi, T. (2010). Comparison of the strengths and difficulties questionnaire (SDQ) scores between children with high-functioning autism spectrum disorder (HFASD) and attention-deficit/hyperactivity disorder (AD/HD). *Brain & Development, 32*(8), 609-612. doi:https://dx.doi.org/10.1016/j.braindev.2009.09.009. Exclusion reason: No psychometric information.

Ingersoll, B., Berger, N., Carlsen, D., & Hamlin, T. (2017). Improving social functioning and challenging behaviors in adolescents with ASD and significant ID: A randomized pilot feasibility trial of reciprocal imitation training in a residential setting. *Developmental neurorehabilitation, 20*(4), 236-246. doi:https://dx.doi.org/10.1080/17518423.2016.1211187. Exclusion reason: Sample size = < 20.

Irwanto, Kahfi, M., Febriyana, N., Hartini, S., & Takada, S. (2019). Emotional and Behavioral Problems of Pre-school Children with Autistic Spectrum Disorder Assessed by the Child Behavior Checklist 11/2-5. *Kobe Journal of Medical Sciences, 64*(5), E170-E173. Exclusion reason: No psychometric information.

Isenberg, B. M., Woodward, D. W., Burke, C. W., Nowinski, L. A., Joshi, G., & Wilens, T. E. (2021). Psychiatric comorbidity associated with co-occurring autism spectrum disorder and substance use disorder. *Research in Autism Spectrum Disorders, 82 (no pagination)*(101728). doi:https://dx.doi.org/10.1016/j.rasd.2021.101728. Exclusion reason: No psychometric information.

Isong, I. A., Rao, S. R., Holifield, C., Iannuzzi, D., Hanson, E., Ware, J., & Nelson, L. P. (2014). Addressing dental fear in children with autism spectrum disorders: A randomized controlled pilot study using electronic screen media. *Clinical Pediatrics, 53(3)*, 230-237. doi:http://dx.doi.org/10.1177/0009922813517169. Exclusion reason: Not relevant measurement tool.

Jacob, S., Francis, S., Rawls, E., Tseng, A., Conelea, C., Grissom, N., . . . Ma, S. (2021). Using causal discovery analysis to explore sex differences in restricted/repetitive behaviors and other features of autism. *Neuropsychopharmacology, 46*, 150. doi:https://dx.doi.org/10.1038/s41386-021-01236-7. Exclusion reason: Conference abstract.

Jang, J., Dixon, D. R., Tarbox, J., & Granpeesheh, D. (2011). Symptom severity and challenging behavior in children with ASD. *Research in Autism Spectrum Disorders, 5(3)*, 1028-1032. doi:http://dx.doi.org/10.1016/j.rasd.2010.11.008. Exclusion reason: No psychometric information.

Jang, J., & Matson, J. L. (2015). Autism severity as a predictor of comorbid conditions. *Journal of Developmental and Physical Disabilities, 27*(3), 405-415. doi:https://dx.doi.org/10.1007/s10882-015-9421-9. Exclusion reason: No psychometric information.

Jang, J., Matson, J. L., Cervantes, P. E., & Goldin, R. L. (2013). The relationship between race and comorbid symptoms in infants and toddlers with autism spectrum disorder. *Research in Autism Spectrum Disorders, 7(11)*, 1433-1438. doi:http://dx.doi.org/10.1016/j.rasd.2013.08.011. Exclusion reason: Publication withdrawn

Jang, J., Matson, J. L., Williams, L. W., Tureck, K., Goldin, R. L., & Cervantes, P. E. (2013). Rates of comorbid symptoms in children with ASD, ADHD, and comorbid ASD and ADHD. *Research in Developmental Disabilities, 34*(8), 2369-2378. doi:https://dx.doi.org/10.1016/j.ridd.2013.04.021. Exclusion reason: No psychometric information.

Jenkins, S. R., & DiGennaro Reed, F. D. (2013). An experimental analysis of the effects of therapeutic horseback riding on the behavior of children with autism. *Research in Autism Spectrum Disorders, 7*(6), 721-740. doi:https://dx.doi.org/10.1016/j.rasd.2013.02.008. Exclusion reason: Sample size = < 20.

Johnson, C. R., Smith, T., DeMand, A., Lecavalier, L., Evans, V., Gurka, M., . . . Scahill, L. (2018). Exploring sleep quality of young children with autism spectrum disorder and disruptive behaviors. *Sleep Medicine, 44*, 61-66. doi:https://dx.doi.org/10.1016/j.sleep.2018.01.008. Exclusion reason: No psychometric information.

Johnson, C. R., Turner, K., Stewart, P. A., Schmidt, B., Shui, A., Macklin, E., . . . Hyman, S. L. (2014). Relationships between feeding problems, behavioral characteristics and nutritional quality in children with ASD. *Journal of Autism and Developmental Disorders, 44*(9), 2175-2184. doi:https://dx.doi.org/10.1007/s10803-014-2095-9. Exclusion reason: No psychometric information.

Joshi, G., Faraone, S. V., Wozniak, J., Petty, C., Fried, R., Galdo, M., . . . Biederman, J. (2014). Examining the clinical correlates of autism spectrum disorder in youth by ascertainment source. *Journal of Autism and Developmental Disorders, 44*(9), 2117-2126. doi:https://dx.doi.org/10.1007/s10803-014-2063-4. Exclusion reason: No psychometric information.

Joshi, G., Gonenc, A., Belser, A., Hoskova, B., De Leon, M., & Biederman, J. (2018). Glutamate anterior cingulate cortex glutamate activity in autism spectrum disorder with and without emotional dysregulation. *European Psychiatry, 48(Supplement 1)*, S90-S91. doi:http://dx.doi.org/10.1016/j.eurpsy.2017.12.022. Exclusion reason: Conference abstract.

Joshi, G., Petty, C., Wozniak, J., Henin, A., Fried, R., Galdo, M., . . . Biederman, J. (2010). The heavy burden of psychiatric comorbidity in youth with autism spectrum disorders: a large comparative study of a psychiatrically referred population. *Journal of Autism & Developmental Disorders, 40*(11), 1361-1370. doi:https://dx.doi.org/10.1007/s10803-010-0996-9. Exclusion reason: No psychometric information.

Joshi, G., Wozniak, J., Fitzgerald, M., Faraone, S., Fried, R., Galdo, M., . . . Biederman, J. (2018). High Risk for Severe Emotional Dysregulation in Psychiatrically Referred Youth with Autism Spectrum Disorder: A Controlled Study. *Journal of Autism & Developmental Disorders, 48*(9), 3101-3115. doi:https://dx.doi.org/10.1007/s10803-018-3542-9. Exclusion reason: No psychometric information.

Joshi, G., Wozniak, J., Petty, C., Martelon, M. K., Fried, R., Bolfek, A., . . . Biederman, J. (2013). Psychiatric comorbidity and functioning in a clinically referred population of adults with autism spectrum disorders: A comparative study. *Journal of Autism and Developmental Disorders, 43*(6), 1314-1325. doi:https://dx.doi.org/10.1007/s10803-012-1679-5. Exclusion reason: Non-ASD sample.

Juranek, J., Felipek, P. A., Berenji, G. R., Modahl, C., Osann, K., & Spence, M. A. (2006). Association between amygdala volume and anxiety level: Magnetic resonance imaging (MRI) study in autistic children. *Journal of Child Neurology, 21(12)*, 1051-1058. doi:http://dx.doi.org/10.1177/7010.2006.00237. Exclusion reason: No psychometric information.

Jussila, K., Ebeling, H., Mattila, M. L., Sanna, K. G., Hurtig, T., Moilanen, I., & Pauls, D. (2011). Autistic traits and overall functioning. *European Child and Adolescent Psychiatry, 1)*, S12. doi:http://dx.doi.org/10.1007/s00787-011-0181-5. Exclusion reason: Conference abstract.

Kaba, D., & Soykan Aysev, A. (2020). Evaluation of Autism Spectrum Disorder in Early Childhood According to the DSM-5 Diagnostic Criteria. *Turk Psikiyatri Dergisi, 31*(2), 106-112. Exclusion reason: No psychometric information.

Kalb, L. G., Hagopian, L. P., Gross, A. L., & Vasa, R. A. (2018). "Psychometric characteristics of the mental health crisis assessment scale in youth with autism spectrum disorder": Erratum. *Journal of Child Psychology and Psychiatry, 59*(7), e1. doi:https://dx.doi.org/10.1111/jcpp.12935. Exclusion reason: No psychometric information.

Kalvin, C., Ibrahim, K., Marsh, C., & Sukhodolsky, D. G. (2019). 1.24 Assessing Irritability in Children with Asd Using the Affective Reactivity Index. *Journal of the American Academy of Child and Adolescent Psychiatry, 58(10 Supplement)*, S154. doi:http://dx.doi.org/10.1016/j.jaac.2019.08.046. Exclusion reason: Conference abstract.

Kamiya, C., Kagitani-Shimono, K., Iwatani, Y., Tachibana, M., Mohri, I., & Taniike, M. (2021). The relationship between multisensory hypersensitivity and behavioral problems in children with autism spectrum disorders. *Journal of Brain Science, 50*, 63-100. doi:https://dx.doi.org/10.20821/jbs.50.0_63. Exclusion reason: No psychometric information.

Kang, E., Gadow, K. D., & Lerner, M. D. (2020). Atypical Communication Characteristics, Differential Diagnosis, and the Autism Spectrum Disorder Phenotype in Youth. *Journal of Clinical Child & Adolescent Psychology, 49*(2), 251-263. doi:https://dx.doi.org/10.1080/15374416.2018.1539912. Exclusion reason: No psychometric information.

Kang, Y. Q., Teo, C. M., Tan, M. L., Aw, M. M., Chan, Y. H., & Chong, S. C. (2022). Feeding difficulties in Asian children with autism spectrum disorder. *Pediatrics & Neonatology, 63*(1), 48-56. doi:https://dx.doi.org/10.1016/j.pedneo.2021.06.015. Exclusion reason: No psychometric information.

Karabekiroglu, K., & Karabekiroglu, A. (2014). Comorbidity in autism spectrum disorders: A sample of 258 cases. *Klinik Psikofarmakoloji Bulteni, 1)*, S216-S217. Exclusion reason: Conference abstract.

Karakoc Demirkaya, S., Tutkunkardas, M. D., & Mukaddes, N. M. (2016). Assessment of suicidality in children and adolescents with diagnosis of high functioning autism spectrum disorder in a Turkish clinical sample. *Neuropsychiatric Disease & Treatment, 12*, 2921-2926. Exclusion reason: No psychometric information.

Kasperzack, D., Schrott, B., Mingebach, T., Becker, K., Burghardt, R., & Kamp-Becker, I. (2020). Effectiveness of the Stepping Stones Triple P group parenting program in reducing comorbid behavioral problems in children with autism. *Autism, 24(2)*, 423-436. doi:http://dx.doi.org/10.1177/1362361319866063. Exclusion reason: Effect study.

Kaushal, S. A., Gladstone, T. R., Bertschinger, E. J., Tudor, M. E., & Sukhodolsky, D. G. (2017). Callous-unemotional traits and aggressive behavior in children with autism spectrum disorder. *Journal of the American Academy of Child and Adolescent Psychiatry, 56(10)*, S259-S260. doi:http://dx.doi.org/10.1016/j.jaac.2017.09.302. Exclusion reason: Conference abstract.

Kawaoka, N., Ohashi, K., Fukuhara, S., Miyachi, T., Asai, T., Imaeda, M., & Saitoh, S. (2021). Impact of School Closures due to COVID-19 on Children with Neurodevelopmental Disorders in Japan. *Journal of Autism & Developmental Disorders, 03*, 03. doi:https://dx.doi.org/10.1007/s10803-021-05119-0. Exclusion reason: No psychometric information.

Kerns, C. M., Kendall, P. C., Zickgraf, H., Franklin, M. E., Miller, J., & Herrington, J. (2015). Not to be overshadowed or overlooked: functional impairments associated with comorbid anxiety disorders in youth with ASD. *Behavior Therapy, 46*(1), 29-39. doi:https://dx.doi.org/10.1016/j.beth.2014.03.005. Exclusion reason: No psychometric information.

Kildahl, A. N., Oddli, H. W., & Helverschou, S. B. (2020). Potentially traumatic experiences and behavioural symptoms in adults with autism and intellectual disability referred for psychiatric assessment. *Research in Developmental Disabilities, 107*, 103788. doi:https://dx.doi.org/10.1016/j.ridd.2020.103788. Exclusion reason: No psychometric information.

Kim, H., Ahn, J., Lee, H., Ha, S., & Cheon, K. A. (2020). Differences in Language Ability and Emotional-Behavioral Problems according to Symptom Severity in Children with Autism Spectrum Disorder. *Yonsei Medical Journal, 61*(10), 880-890. doi:https://dx.doi.org/10.3349/ymj.2020.61.10.880. Exclusion reason: No psychometric information.

Kim, I., Ekas, N. V., & Hock, R. (2016). Associations between child behavior problems, family management, and depressive symptoms for mothers of children with autism spectrum disorder. *Research in Autism Spectrum Disorders, 26*, 80-90. doi:https://dx.doi.org/10.1016/j.rasd.2016.03.009. Exclusion reason: Not relevant measurement tool.

Kim, J. A., Szatmari, P., Bryson, S. E., Streiner, D. L., & Wilson, F. J. (2000). The prevalence of anxiety and mood problems among children with autism and Asperger syndrome. *Autism, 4(2)*, 117-132. doi:http://dx.doi.org/10.1177/1362361300004002002. Exclusion reason: No psychometric information.

Knuppel, A., Telleus, G. K., Jakobsen, H., & Lauritsen, M. B. (2018). Quality of life in adolescents and adults with autism spectrum disorder: Results from a nationwide Danish survey using self-reports and parental proxy-reports. *Research in Developmental Disabilities, 83*, 247-259. doi:http://dx.doi.org/10.1016/j.ridd.2018.09.004. Exclusion reason: Not relevant measurement tool.

Kobayashi, R., & Murata, T. (1998). Behavioral characteristics of 187 young adults with autism. *Psychiatry & Clinical Neurosciences, 52*(4), 383-390. Exclusion reason: No psychometric information.

Koenig, K. P., Buckley-Reen, A., & Garg, S. (2012). Efficacy of the Get Ready to Learn yoga program among children with autism spectrum disorders: a pretest-posttest control group design. *American Journal of Occupational Therapy, 66*(5), 538-546. doi:https://dx.doi.org/10.5014/ajot.2012.004390. Exclusion reason: Effect study.

Konst, M. J., & Matson, J. L. (2014). Comorbid psychopathology symptom rates in infants and toddlers with Autism Spectrum Disorders. *Research in Autism Spectrum Disorders, 8(2)*, 147-155. doi:http://dx.doi.org/10.1016/j.rasd.2013.10.011. Exclusion reason: No psychometric information.

Konst, M. J., & Matson, J. L. (2014). Temporal and diagnostic influences on the expression of comorbid psychopathology symptoms in infants and toddlers with Autism Spectrum Disorder. *Research in Autism Spectrum Disorders, 8(3)*, 200-208. doi:http://dx.doi.org/10.1016/j.rasd.2013.11.009. Exclusion reason: No psychometric information.

Konst, M. J., Matson, J. L., & Turygin, N. (2013). Comparing the rates of tantrum behavior in children with ASD and ADHD as well as children with comorbid ASD and ADHD diagnoses. *Research in Autism Spectrum Disorders, 7(11)*, 1339-1345. doi:http://dx.doi.org/10.1016/j.rasd.2013.07.023. Exclusion reason: No psychometric information.

Konst, M. J., Matson, J. L., & Turygin, N. (2013). Exploration of the correlation between autism spectrum disorder symptomology and tantrum behaviors. *Research in Autism Spectrum Disorders, 7(9)*, 1068-1074. doi:http://dx.doi.org/10.1016/j.rasd.2013.05.006. Exclusion reason: No psychometric information.

Kopp, S., Kelly, K. B., & Gillberg, C. (2010). Girls with social and/or attention deficits: a descriptive study of 100 clinic attenders. *Journal of Attention Disorders, 14*(2), 167-181. doi:https://dx.doi.org/10.1177/1087054709332458. Exclusion reason: No psychometric information.

Kose, S., Kayan, B., Ocakoglu, F. T., Ozbaran, N. B., & Aydin, C. (2015). Reassessment of pervasive developmental disorder-'not otherwise specified cases' outcomes according to DSM IV-TR criteria. *Klinik Psikofarmakoloji Bulteni, 1)*, S105-S106. Exclusion reason: Conference abstract.

Koshy, B., Roshan, R., Devarajan, C. H., & Oommen, S. (2020). Co-morbidity evaluation in the management of ASD: An Indian experience. *Developmental Medicine and Child Neurology, 62(SUPPL 4)*, 57. doi:https://dx.doi.org/10.1111/dmcn.14689. Exclusion reason: Conference abstract.

Kozlowski, A. M., & Matson, J. L. (2012). An examination of challenging behaviors in autistic disorder versus pervasive developmental disorder not otherwise specified: Significant differences and gender effects. *Research in Autism Spectrum Disorders, 6(1)*, 319-325. doi:http://dx.doi.org/10.1016/j.rasd.2011.06.005. Exclusion reason: No psychometric information.

Kozlowski, A. M., Matson, J. L., Belva, B., & Rieske, R. (2012). Feeding and sleep difficulties in toddlers with autism spectrum disorders. *Research in Autism Spectrum Disorders, 6(1)*, 385-390. doi:http://dx.doi.org/10.1016/j.rasd.2011.06.012. Exclusion reason: Non-ASD sample.

Kubo, N., Kitagawa, M., Iwamoto, S., & Kishimoto, T. (2021). Effects of an attachment-based parent intervention on mothers of children with autism spectrum disorder: preliminary findings from a non-randomized controlled trial. *Child & Adolescent Psychiatry & Mental Health [Electronic Resource], 15*(1), 37. doi:https://dx.doi.org/10.1186/s13034-021-00389-z. Exclusion reason: Effect study.

Kucuk, A., Maner, F., & Ceylan, M. E. (2019). Autism spectrum disorders among adolescents and adults and comparison with schizophrenia. *European Research Journal, 5(6)*, 962-968. doi:http://dx.doi.org/10.18621/eurj.441214. Exclusion reason: No psychometric information.

Kuhlthau, K., Kovacs, E., Hall, T., Clemmons, T., Orlich, F., Delahaye, J., & Sikora, D. (2013). Health-related quality of life for children with ASD: Associations with behavioral characteristics. *Research in Autism Spectrum Disorders, 7(9)*, 1035-1042. doi:http://dx.doi.org/10.1016/j.rasd.2013.04.006. Exclusion reason: No psychometric information.

Kuhlthau, K., Orlich, F., Hall, T. A., Sikora, D., Kovacs, E. A., Delahaye, J., & Clemons, T. E. (2010). Health-related quality of life in children with autism spectrum disorders: Results from the Autism Treatment Network. *Journal of Autism and Developmental Disorders, 40*(6), 721-729. doi:https://dx.doi.org/10.1007/s10803-009-0921-2. Exclusion reason: No psychometric information.

Kuhlthau, K. A., McDonnell, E., Coury, D. L., Payakachat, N., & Macklin, E. (2018). Associations of quality of life with health-related characteristics among children with autism. *Autism, 22*(7), 804-813. doi:https://dx.doi.org/10.1177/1362361317704420. Exclusion reason: No psychometric information.

Kumar, S., Devendran, Y., & Amrtavarshini, R. (2017). Prevalence of autism spectrum disorders and its association with Epileptiform activity among children with intellectual disability in a tertiary centre. *Journal of Indian Association for Child and Adolescent Mental Health, 13(1)*, 26-47. Exclusion reason: Not possible to obtain the full text.

Kumar, S., Venkatakrishna, S., & Ruth, S. (2021). Gastrointestinal issues with behavior problems and sleep issues in children and adolescents with autism spectrum disorder (Asd): A preliminary estimate. *Journal of Indian Association for Child and Adolescent Mental Health, 17(3)*, 112-126. Exclusion reason: No psychometric information.

Kusaka, H., Miyawaki, D., Nakai, Y., Okamoto, H., Futoo, E., Goto, A., . . . Inoue, K. (2014). Psychiatric comorbidity in children with high-functioning pervasive developmental disorder. *Osaka City Medical Journal, 60*(1), 1-10. Exclusion reason: No psychometric information.

Kuusikko, S., Pollock-Wurman, R., Jussila, K., Carter, A. S., Mattila, M. L., Ebeling, H., . . . Moilanen, I. (2008). Social anxiety in high-functioning children and adolescents with Autism and Asperger syndrome. *Journal of Autism & Developmental Disorders, 38*(9), 1697-1709. doi:https://dx.doi.org/10.1007/s10803-008-0555-9. Exclusion reason: No psychometric information.

La Malfa, G., Lassi, S., Salvini, R., Giganti, C., Bertelli, M., & Albertini, G. (2007). The relationship between autism and psychiatric disorders in Intellectually Disabled Adults. *Research in Autism Spectrum Disorders, 1(3)*, 218-228. doi:http://dx.doi.org/10.1016/j.rasd.2006.10.004. Exclusion reason: Non-ASD sample.

Lai, M. C., Lombardo, M. V., Pasco, G., Ruigrok, A. N., Wheelwright, S. J., Sadek, S. A., . . . Baron-Cohen, S. (2011). A behavioral comparison of male and female adults with high functioning autism spectrum conditions. *PLoS ONE [Electronic Resource], 6*(6), e20835. doi:https://dx.doi.org/10.1371/journal.pone.0020835. Exclusion reason: Not relevant measurement tool.

Lai, M. C., Lombardo, M. V., Pasco, G., Ruigrok, A. N. V., Wheelwright, S. J., Sadek, S. A., . . . Baron-Cohen, S. (2011). A behavioral comparison of male and female adults with high functioning autism spectrum conditions. *PLoS ONE [Electronic Resource], 6(6) (no pagination)*(e20835). doi:http://dx.doi.org/10.1371/journal.pone.0020835. Exclusion reason: Not relevant measurement tool.

Lakatosova, S., Jansakova, K., Babkova, J., Repiska, G., Belica, I., Vidosovicova, M., & Ostatnikova, D. (2022). The Relationship of Steroid Hormones, Genes Related to Testosterone Metabolism and Behavior in Boys With Autism in Slovakia. *Psychiatry Investigation, 19*(2), 73-84. doi:https://dx.doi.org/10.30773/pi.2021.0094. Exclusion reason: No psychometric information.

Lanyi, J., Mannion, A., Chen, J. L., & Leader, G. (2022). Relationship between Comorbid Psychopathology in Children and Adolescents with Autism Spectrum Disorder and Parental Well-being. *Developmental neurorehabilitation, 25*(3), 151-161. doi:https://dx.doi.org/10.1080/17518423.2021.1922529. Exclusion reason: No psychometric information.

Lauderdale-Littin, S., Howell, E., & Blacher, J. (2013). Educational placement for children with autism spectrum disorders in public and non-public school settings: The impact of social skills and behavior problems. *Education and Training in Autism and Developmental Disabilities, 48*(4), 469-478. Exclusion reason: Conference abstract.

Lawson, R. A., Papadakis, A. A., Higginson, C. I., Barnett, J. E., Wills, M. C., Strang, J. F., . . . Kenworthy, L. (2015). Everyday executive function impairments predict comorbid psychopathology in autism spectrum and attention deficit hyperactivity disorders. *Neuropsychology, 29*(3), 445-453. doi:https://dx.doi.org/10.1037/neu0000145. Exclusion reason: No psychometric information.

Leader, G., Browne, H., Whelan, S., Cummins, H., & Mannion, A. (2022). Affective problems, gastrointestinal symptoms, sleep problems, and challenging behaviour in children and adolescents with autism spectrum disorder. *Research in Autism Spectrum Disorders, 92 (no pagination)*(101915). doi:https://dx.doi.org/10.1016/j.rasd.2022.101915. Exclusion reason: Not relevant measurement tool.

Leader, G., Hogan, A., Chen, J. L., Maher, L., Naughton, K., O'Rourke, N., . . . Mannion, A. (2022). Age of Autism Spectrum Disorder Diagnosis and Comorbidity in Children and Adolescents with Autism Spectrum Disorder. *Developmental neurorehabilitation, 25*(1), 29-37. doi:https://dx.doi.org/10.1080/17518423.2021.1917717. Exclusion reason: No psychometric information.

Leader, G., Mooney, A., Chen, J. L., Whelan, S., Naughton, K., Maher, L., & Mannion, A. (2021). The Co-Occurrence of Autism Spectrum Disorder and Cerebral Palsy and Associated Comorbid Conditions in Children and Adolescents. *Developmental neurorehabilitation*, 1-9. doi:https://dx.doi.org/10.1080/17518423.2021.2011456. Exclusion reason: No psychometric information.

Leader, G., Moore, R., Chen, J. L., Caher, A., Arndt, S., Maher, L., . . . Mannion, A. (2021). Attention deficit hyperactivity disorder (ADHD) symptoms, comorbid psychopathology, behaviour problems and gastrointestinal symptoms in children and adolescents with autism spectrum disorder. *Irish Journal of Psychological Medicine*, 1-11. doi:https://dx.doi.org/10.1017/ipm.2020.135. Exclusion reason: Not relevant measurement tool.

Leader, G., O'Reilly, M., Gilroy, S. P., Chen, J. L., Ferrari, C., & Mannion, A. (2021). Comorbid feeding and gastrointestinal symptoms, challenging behavior, sensory issues, adaptive functioning and quality of life in children and adolescents with autism spectrum disorder. *Developmental neurorehabilitation, 24*(1), 35-44. doi:https://dx.doi.org/10.1080/17518423.2020.1770354. Exclusion reason: No psychometric information.

Leader, G., Tuohy, E., Chen, J. L., Mannion, A., & Gilroy, S. P. (2020). Feeding Problems, Gastrointestinal Symptoms, Challenging Behavior and Sensory Issues in Children and Adolescents with Autism Spectrum Disorder. *Journal of Autism & Developmental Disorders, 50*(4), 1401-1410. doi:https://dx.doi.org/10.1007/s10803-019-04357-7. Exclusion reason: No psychometric information.

Lecavalier, L. (2006). Behavioral and emotional problems in young people with pervasive developmental disorders: relative prevalence, effects of subject characteristics, and empirical classification. *Journal of Autism & Developmental Disorders, 36*(8), 1101-1114. Exclusion reason: No psychometric information.

Lecavalier, L., McCracken, C. E., Aman, M. G., McDougle, C. J., McCracken, J. T., Tierney, E., . . . Scahill, L. (2019). An exploration of concomitant psychiatric disorders in children with autism spectrum disorder. *Comprehensive Psychiatry, 88*, 57-64. doi:https://dx.doi.org/10.1016/j.comppsych.2018.10.012. Exclusion reason: No psychometric information.

Lei, J., Sukhodolsky, D. G., Abdullahi, S. M., Braconnier, M. L., & Ventola, P. (2017). Reduced anxiety following pivotal response treatment in young children with autism spectrum disorder. *Research in Autism Spectrum Disorders, 43-44*, 1-7. doi:http://dx.doi.org/10.1016/j.rasd.2017.09.002. Exclusion reason: No psychometric information.

Lever, A. G., & Geurts, H. M. (2016). Psychiatric co-occurring symptoms and disorders in young, middle-aged, and older adults with autism spectrum disorder. *Journal of Autism and Developmental Disorders, 46*(6), 1916-1930. doi:https://dx.doi.org/10.1007/s10803-016-2722-8. Exclusion reason: No psychometric information.

Levinson, S., Neuspiel, J., Eisenhower, A., & Blacher, J. (2021). Parent-teacher disagreement on ratings of behavior problems in children with ASD: Associations with parental school involvement over time. *Journal of Autism and Developmental Disorders, 51*(6), 1966-1982. doi:https://dx.doi.org/10.1007/s10803-020-04675-1. Exclusion reason: No psychometric information.

Lin, H. Y., Ni, H. C., Tseng, W. Y. I., & Gau, S. S. F. (2020). Characterizing intrinsic functional connectivity in relation to impaired self-regulation in intellectually able male youth with autism spectrum disorder. *Autism, 24(5)*, 1201-1216. doi:http://dx.doi.org/10.1177/1362361319888104. Exclusion reason: No psychometric information.

Lin, J., Magiati, I., Chiong, S. H. R., Singhal, S., Riard, N., Ng, I. H. X., . . . Wong, C. M. (2019). The Relationship Among Screen Use, Sleep, and Emotional/Behavioral Difficulties in Preschool Children with Neurodevelopmental Disorders. *Journal of developmental and behavioral pediatrics : JDBP, 40(7)*, 519-529. doi:http://dx.doi.org/10.1097/DBP.0000000000000683. Exclusion reason: Non-ASD sample.

Lin, Y. N., Iao, L. S., Lee, Y. H., & Wu, C. C. (2021). Parenting Stress and Child Behavior Problems in Young Children with Autism Spectrum Disorder: Transactional Relations Across Time. *Journal of Autism and Developmental Disorders, 51(7)*, 2381-2391. doi:http://dx.doi.org/10.1007/s10803-020-04720-z. Exclusion reason: No psychometric information.

Lin, Y.-N., Iao, L.-S., Lee, Y.-H., & Wu, C.-C. (2021). Parenting stress and child behavior problems in young children with autism spectrum disorder: Transactional relations across time. *Journal of Autism and Developmental Disorders, 51*(7), 2381-2391. doi:https://dx.doi.org/10.1007/s10803-020-04720-z. Exclusion reason: No psychometric information.

Lindly, O. J., Chan, J., Levy, S. E., Parker, R. A., & Kuhlthau, K. A. (2020). Service use classes among school-aged children from the autism treatment network registry. *Pediatrics, 145 (no pagination)*(e20191895Q). doi:http://dx.doi.org/10.1542/peds.2019-1895Q. Exclusion reason: No psychometric information.

Lindor, E., Sivaratnam, C., May, T., Stefanac, N., Howells, K., & Rinehart, N. (2019). Problem Behavior in Autism Spectrum Disorder: Considering Core Symptom Severity and Accompanying Sleep Disturbance. *Frontiers in psychiatry Frontiers Research Foundation, 10*, 487. doi:https://dx.doi.org/10.3389/fpsyt.2019.00487. Exclusion reason: No psychometric information.

Lo, Y. C., Chen, Y. J., Hsu, Y. C., Chien, Y. L., Gau, S. S. F., & Tseng, W. Y. I. (2019). Altered frontal aslant tracts as a heritable neural basis of social communication deficits in autism spectrum disorder: A sibling study using tract-based automatic analysis. *Autism Research, 12(2)*, 225-238. doi:http://dx.doi.org/10.1002/aur.2044. Exclusion reason: No psychometric information.

Lohr, W. D., Daniels, K., Wiemken, T., Williams, P. G., Kelley, R. R., Kuravackel, G., & Sears, L. (2017). The Screen for Child Anxiety-Related Emotional Disorders Is Sensitive but Not Specific in Identifying Anxiety in Children with High-Functioning Autism Spectrum Disorder: A Pilot Comparison to the Achenbach System of Empirically Based Assessment Scales. *Frontiers in psychiatry Frontiers Research Foundation, 8*, 138. doi:https://dx.doi.org/10.3389/fpsyt.2017.00138. Exclusion reason: Not relevant measurement tool.

Lopata, C., Lipinski, A. M., Thomeer, M. L., Rodgers, J. D., Donnelly, J. P., McDonald, C. A., & Volker, M. A. (2017). Open-trial pilot study of a comprehensive outpatient psychosocial treatment for children with high-functioning autism spectrum disorder. *Autism, 21*(1), 108-116. Exclusion reason: Effect study.

Lopata, C., Thomeer, M. L., Volker, M. A., Nida, R. E., & Lee, G. K. (2008). Effectiveness of a manualized summer social treatment program for high-functioning children with autism spectrum disorders. *Journal of Autism and Developmental Disorders, 38*(5), 890-904. doi:https://dx.doi.org/10.1007/s10803-007-0460-7. Exclusion reason: Not relevant measurement tool.

Lorenzi, J., Durham, H. S., Afzal, R., Green, M., & Dawson, G. (2017). Associations between behavioral comorbidities in young children with autism spectrum disorder and parenting stress. *Journal of the American Academy of Child and Adolescent Psychiatry, 56(10)*, S261-S262. doi:http://dx.doi.org/10.1016/j.jaac.2017.09.307. Exclusion reason: Conference abstract.

Louwerse, A., Eussen, M. L., Van der Ende, J., de Nijs, P. F., Van Gool, A. R., Dekker, L. P., . . . Greaves-Lord, K. (2015). ASD Symptom Severity in Adolescence of Individuals Diagnosed with PDD-NOS in Childhood: Stability and the Relation with Psychiatric Comorbidity and Societal Participation. *Journal of Autism & Developmental Disorders, 45*(12), 3908-3918. doi:https://dx.doi.org/10.1007/s10803-015-2595-2. Exclusion reason: No psychometric information.

Louwerse, A., Eussen, M. L. J. M., Van der Ende, J., de Nijs, P. F. A., Van Gool, A. R., Dekker, L. P., . . . Greaves-Lord, K. (2015). ASD Symptom Severity in Adolescence of Individuals Diagnosed with PDD-NOS in Childhood: Stability and the Relation with Psychiatric Comorbidity and Societal Participation. *Journal of Autism and Developmental Disorders, 45(12)*, 3908-3918. doi:http://dx.doi.org/10.1007/s10803-015-2595-2. Exclusion reason: No psychometric information.

Lovell, B., & Wetherell, M. A. (2015). Child behaviour problems mediate the association between coping and perceived stress in caregivers of children with autism. *Research in Autism Spectrum Disorders, 20*, 17-23. doi:https://dx.doi.org/10.1016/j.rasd.2015.08.003. Exclusion reason: No psychometric information.

Lugnegard, T., Hallerback, M. U., & Gillberg, C. (2011). Psychiatric comorbidity in young adults with a clinical diagnosis of Asperger syndrome. *Research in Developmental Disabilities, 32*(5), 1910-1917. doi:https://dx.doi.org/10.1016/j.ridd.2011.03.025. Exclusion reason: No psychometric information.

Lugo-Marin, J., Gisbert-Gustemps, L., Setien-Ramos, I., Espanol-Martin, G., Ibanez-Jimenez, P., Forner-Puntonet, M., . . . Ramos-Quiroga, J. A. (2021). COVID-19 pandemic effects in people with Autism Spectrum Disorder and their caregivers: Evaluation of social distancing and lockdown impact on mental health and general status. *Research in Autism Spectrum Disorders, 83*, 101757. doi:https://dx.doi.org/10.1016/j.rasd.2021.101757. Exclusion reason: No psychometric information.

Lundqvist, L. O., Andersson, G., & Viding, J. (2009). Effects of vibroacoustic music on challenging behaviors in individuals with autism and developmental disabilities. *Research in Autism Spectrum Disorders, 3(2)*, 390-400. doi:http://dx.doi.org/10.1016/j.rasd.2008.08.005. Exclusion reason: Non-ASD sample.

Lundqvist, L.-O., Andersson, G., & Viding, J. (2009). Effects of vibroacoustic music on challenging behaviors in individuals with Autism and developmental disabilities. *Research in Autism Spectrum Disorders, 3*(2), 390-400. doi:https://dx.doi.org/10.1016/j.rasd.2008.08.005. Exclusion reason: Non-ASD sample.

Lundstrom, S., Reichenberg, A., Melke, J., Rastam, M., Kerekes, N., Lichtenstein, P., . . . Anckarsater, H. (2015). Autism spectrum disorders and coexisting disorders in a nationwide Swedish twin study. *Journal of Child Psychology & Psychiatry & Allied Disciplines, 56*(6), 702-710. doi:https://dx.doi.org/10.1111/jcpp.12329. Exclusion reason: No psychometric information.

Luteijn, E., Luteijn, F., Jackson, S., Volkmar, F., & Minderaa, R. (2000). The children's Social Behavior Questionnaire for milder variants of PDD problems: evaluation of the psychometric characteristics. *Journal of Autism & Developmental Disorders, 30*(4), 317-330. Exclusion reason: Not relevant measurement tool.

Lyall, K., Ashwood, P., Van De Water, J., & Hertz-Picciotto, I. (2012). Maternal immune-mediated conditions in association with child immune-related outcomes and autism spectrum disorders. *American Journal of Epidemiology, 11)*, S127. doi:http://dx.doi.org/10.1093/aje/kws258. Exclusion reason: Conference abstract.

Lyall, K., Schweitzer, J. B., Schmidt, R. J., Hertz-Picciotto, I., & Solomon, M. (2017). Inattention and hyperactivity in association with autism spectrum disorders in the CHARGE study. *Research in Autism Spectrum Disorders, 35*, 1-12. doi:https://dx.doi.org/10.1016/j.rasd.2016.11.011. Exclusion reason: No psychometric information.

Macari, S., DiNicola, L., Kane-Grade, F., Prince, E., Vernetti, A., Powell, K., . . . Chawarska, K. (2018). Emotional expressivity in toddlers with autism spectrum disorder. *Journal of the American Academy of Child & Adolescent Psychiatry, 57*(11), 828-836. doi:https://dx.doi.org/10.1016/j.jaac.2018.07.872. Exclusion reason: Not relevant measurement tool.

Machado Junior, S. B., Celestino, M. I., Serra, J. P., Caron, J., & Ponde, M. P. (2016). Risk and protective factors for symptoms of anxiety and depression in parents of children with autism spectrum disorder. *Developmental neurorehabilitation, 19*(3), 146-153. doi:https://dx.doi.org/10.3109/17518423.2014.925519. Exclusion reason: No psychometric information.

MacHado Junior, S. B., Celestino, M. I. O., Serra, J. P. C., Caron, J., & Ponde, M. P. (2016). Risk and protective factors for symptoms of anxiety and depression in parents of children with autism spectrum disorder. *Developmental neurorehabilitation, 19(3)*, 146-153. doi:http://dx.doi.org/10.3109/17518423.2014.925519. Exclusion reason: No psychometric information.

Magiati, I., Chan, J. Y., Tan, W. L. J., & Poon, K. K. (2014). Do non-referred young people with Autism Spectrum Disorders and their caregivers agree when reporting anxiety symptoms? A preliminary investigation using the Spence Children's Anxiety Scale. *Research in Autism Spectrum Disorders, 8(5)*, 546-558. doi:http://dx.doi.org/10.1016/j.rasd.2014.01.015. Exclusion reason: No psychometric information.

Mahan, S., & Matson, J. L. (2011). Children and adolescents with autism spectrum disorders compared to typically developing controls on the Behavioral Assessment System for Children, Second Edition (BASC-2). *Research in Autism Spectrum Disorders, 5*(1), 119-125. doi:https://dx.doi.org/10.1016/j.rasd.2010.02.007. Exclusion reason: No psychometric information.

Makrygianni, M. K., Gena, A., & Reed, P. (2012). The effectiveness of teaching intervention programs for 6.5 to 14 years old children with autism spectrum disorders in Greece. *Advances in psychology research , Vol*, 91 (pp. 25-46). x, 168. Exclusion reason: Published book.

Maljaars, J., Boonen, H., Lambrechts, G., Van Leeuwen, K., & Noens, I. (2014). Maternal parenting behavior and child behavior problems in families of children and adolescents with autism spectrum disorder. *Journal of Autism and Developmental Disorders, 44*(3), 501-512. doi:https://dx.doi.org/10.1007/s10803-013-1894-8. Exclusion reason: No psychometric information.

Malow, B. A., Marzec, M. L., McGrew, S. G., Wang, L., Henderson, L. M., & Stone, W. L. (2006). Characterizing sleep in children with autism spectrum disorders: a multidimensional approach. *Sleep, 29*(12), 1563-1571. Exclusion reason: No psychometric information.

Mamic, D., Fulgosi-Masnjak, R., & Masnjak, L. (2015). The relation between emotional and social maturity and psychopathology-problem behaviors in children and youths with intellectual disability and autism. *Journal of Intellectual Disability Research, 1)*, 112-113. doi:http://dx.doi.org/10.1111/jir.12214. Exclusion reason: Conference abstract.

Mandy, W., Midouhas, E., Hosozawa, M., Cable, N., Sacker, A., & Flouri, E. (2022). Mental health and social difficulties of late-diagnosed autistic children, across childhood and adolescence. *Journal of Child Psychology & Psychiatry & Allied Disciplines, 16*, 16. doi:https://dx.doi.org/10.1111/jcpp.13587. Exclusion reason: No psychometric information.

Mandy, W., Murin, M., Baykaner, O., Staunton, S., Hellriegel, J., Anderson, S., & Skuse, D. (2016). The transition from primary to secondary school in mainstream education for children with autism spectrum disorder. *Autism, 20*(1), 5-13. doi:https://dx.doi.org/10.1177/1362361314562616. Exclusion reason: No psychometric information.

Mandy, W., Roughan, L., & Skuse, D. (2014). Three dimensions of oppositionality in autism spectrum disorder. *Journal of Abnormal Child Psychology, 42*(2), 291-300. doi:https://dx.doi.org/10.1007/s10802-013-9778-0. Exclusion reason: No psychometric information.

Mannion, A., & Leader, G. (2016). An investigation of comorbid psychological disorders, sleep problems, gastrointestinal symptoms and epilepsy in children and adolescents with autism spectrum disorder: A two year follow-up. *Research in Autism Spectrum Disorders, 22*, 20-33. doi:http://dx.doi.org/10.1016/j.rasd.2015.11.002. Exclusion reason: No psychometric information.

Mannion, A., Leader, G., & Healy, O. (2013). An investigation of comorbid psychological disorders, sleep problems, gastrointestinal symptoms and epilepsy in children and adolescents with Autism Spectrum Disorder. *Research in Autism Spectrum Disorders, 7(1)*, 35-42. doi:http://dx.doi.org/10.1016/j.rasd.2012.05.002. Exclusion reason: No psychometric information.

Manolova, H., Hristova, M., & Staykova, S. (2021). The Importance of Early Psychological Assessment for Differential Diagnosis and Detection of Comorbidity in Children With Autism Spectrum Disorder. *Frontiers in psychiatry Frontiers Research Foundation, 12*, 671744. doi:https://dx.doi.org/10.3389/fpsyt.2021.671744. Exclusion reason: Reviews/theoretical/non-empirical/not original data.

Mansour, R., Dovi, A. T., Lane, D. M., Loveland, K. A., & Pearson, D. A. (2017). ADHD severity as it relates to comorbid psychiatric symptomatology in children with Autism Spectrum Disorders (ASD). *Research in Developmental Disabilities, 60*, 52-64. doi:https://dx.doi.org/10.1016/j.ridd.2016.11.009. Exclusion reason: No psychometric information.

Martin-Borreguero, P., Gomez-Fernandez, A. R., De La Torre-Aguilar, M. J., Gil-Campos, M., Flores-Rojas, K., & Perez-Navero, J. L. (2021). Children With Autism Spectrum Disorder and Neurodevelopmental Regression Present a Severe Pattern After a Follow-Up at 24 Months. *Frontiers in psychiatry Frontiers Research Foundation, 12*, 644324. doi:https://dx.doi.org/10.3389/fpsyt.2021.644324. Exclusion reason: No psychometric information.

Martinez-Gonzalez, A. E., Moreno-Amador, B., & Piqueras, J. A. (2021). Differences in emotional state and autistic symptoms before and during confinement due to the COVID-19 pandemic. *Research in Developmental Disabilities, 116 (no pagination)*(104038). doi:https://dx.doi.org/10.1016/j.ridd.2021.104038. Exclusion reason: No psychometric information.

Martini, M. I., Merkelbach, I., & Begeer, S. (2022). Gestational Age in Autistic Children and Adolescents: Prevalence and Effects on Autism Phenotype. *Journal of Autism & Developmental Disorders, 07*, 07. doi:https://dx.doi.org/10.1007/s10803-022-05466-6. Exclusion reason: No psychometric information.

Matson, J. L., Baglio, C. S., Smiroldo, B. B., Hamilton, M., Packlowskyj, T., Williams, D., & Kirkpatrick-Sanchez, S. (1996). Characteristics of autism as assessed by the Diagnostic Assessment for the Severely Handicapped-II (DASH-II). *Research in Developmental Disabilities, 17(2)*, 135-143. doi:http://dx.doi.org/10.1016/0891-4222%2895%2900044-5. Exclusion reason: Non-ASD sample.

Matson, J. L., Boisjoli, J., & Mahan, S. (2009). The relation of communication and challenging behaviors in infants and toddlers with autism spectrum disorders. *Journal of Developmental and Physical Disabilities, 21*(4), 253-261. doi:https://dx.doi.org/10.1007/s10882-009-9140-1. Exclusion reason: No psychometric information.

Matson, J. L., Dempsey, T., & Rivet, T. T. (2009). The interrelationships of psychopathology symptoms on social skills in adults with autism or PDD-NOS and intellectual disability. *Journal of Developmental and Physical Disabilities, 21*(1), 39-55. doi:https://dx.doi.org/10.1007/s10882-008-9124-6. Exclusion reason: No psychometric information.

Matson, J. L., Fodstad, J. C., & Mahan, S. (2009). Cutoffs, norms, and patterns of comorbid difficulties in children with developmental disabilities on the Baby and Infant Screen for Children with aUtIsm Traits (BISCUIT-Part 2). *Research in Developmental Disabilities, 30*(6), 1221-1228. doi:https://dx.doi.org/10.1016/j.ridd.2009.04.004. Exclusion reason: Non-ASD sample.

Matson, J. L., Gonzalez, M. L., & Rivet, T. T. (2008). Reliability of the Autism Spectrum Disorder-Behavior Problems for Children (ASD-BPC). *Research in Autism Spectrum Disorders, 2(4)*, 696-706. doi:http://dx.doi.org/10.1016/j.rasd.2008.02.003. Exclusion reason: Non-ASD sample.

Matson, J. L., Hess, J. A., & Boisjoli, J. A. (2010). Comorbid psychopathology in infants and toddlers with autism and pervasive developmental disorders-not otherwise specified (PDD-NOS). *Research in Autism Spectrum Disorders, 4(2)*, 300-304. doi:http://dx.doi.org/10.1016/j.rasd.2009.10.001. Exclusion reason: No psychometric information.

Matson, J. L., Mahan, S., Fodstad, J. C., Worley, J. A., Neal, D., & Sipes, M. (2011). Effects of symptoms of co-morbid psychopathology on challenging behaviours among infants and toddlers with Autistic Disorder and PDD-NOS as assessed with the Baby and Infant Screen for Children with aUtIsm Traits (BISCUIT). *Developmental neurorehabilitation, 14*(3), 129-139. doi:https://dx.doi.org/10.3109/17518423.2011.557029. Exclusion reason: No psychometric information.

Matson, J. L., Neal, D., Fodstad, J. C., & Hess, J. A. (2010). The relation of social behaviours and challenging behaviours in infants and toddlers with Autism Spectrum Disorders. *Developmental neurorehabilitation, 13*(3), 164-169. doi:https://dx.doi.org/10.3109/17518420903270683. Exclusion reason: No psychometric information.

Matson, J. L., & Rivet, T. T. (2007). A validity study of the Autism Spectrum Disorders--Behavior Problems for Adults (ASD-BPA) scale. *Journal of Developmental and Physical Disabilities, 19*(6), 557-564. Exclusion reason: Non-ASD sample.

Matson, J. L., & Rivet, T. T. (2008). The effects of severity of autism and PDD-NOS symptoms on challenging behaviors in adults with intellectual disabilities. *Journal of Developmental and Physical Disabilities, 20*(1), 41-51. doi:https://dx.doi.org/10.1007/s10882-007-9078-0. Exclusion reason: Non-ASD sample.

Matson, J. L., Wilkins, J., Boisjoli, J. A., & Smith, K. R. (2008). The validity of the autism spectrum disorders-diagnosis for intellectually disabled adults (ASD-DA). *Research in Developmental Disabilities, 29*(6), 537-546. Exclusion reason: Not relevant measurement tool.

Matson, J. L., Wilkins, J., Sevin, J. A., Knight, C., Boisjoli, J. A., & Sharp, B. (2009). Reliability and item content of the Baby and Infant Screen for Children with aUtIsm Traits (BISCUIT): Parts 1-3. *Research in Autism Spectrum Disorders, 3(2)*, 336-344. doi:http://dx.doi.org/10.1016/j.rasd.2008.08.001. Exclusion reason: Non-ASD sample.

Matthews, M., Bell, E., & Mirfin-Veitch, B. (2018). Comparing psychopathology rates across autism spectrum disorders and intellectual disabilities. *Advances in Mental Health and Intellectual Disabilities, 12*(5-6), 163-172. doi:https://dx.doi.org/10.1108/AMHID-04-2018-0023. Exclusion reason: No psychometric information.

May, T., Brignell, A., & Williams, K. (2020). Autism Spectrum Disorder Prevalence in Children Aged 12-13 Years From the Longitudinal Study of Australian Children. *Autism research : Official Journal of the International Society for Autism Research, 13*(5), 821-827. doi:https://dx.doi.org/10.1002/aur.2286. Exclusion reason: No psychometric information.

Mayes, S. D., Calhoun, S. L., Waschbusch, D. A., Breaux, R. P., & Baweja, R. (2017). Reactive attachment/disinhibited social engagement disorders: Callous-unemotional traits and comorbid disorders. *Research in Developmental Disabilities, 63*, 28-37. doi:http://dx.doi.org/10.1016/j.ridd.2017.02.012. Exclusion reason: No psychometric information.

Mayes, S. D., Castagna, P. J., & Waschbusch, D. A. (2020). Sex Differences in Externalizing and Internalizing Symptoms in ADHD, Autism, and General Population Samples. *Journal of Psychopathology and Behavioral Assessment, 42(3)*, 519-526. doi:http://dx.doi.org/10.1007/s10862-020-09798-4. Exclusion reason: No psychometric information.

Mayes, S. D., Waxmonsky, J. G., Baweja, R., Mattison, R. E., Memon, H., Klein, M., . . . Waschbusch, D. (2020). Symptom scores and medication treatment patterns in children with ADHD versus autism. *Psychiatry Research, 288 (no pagination)*(112937). doi:http://dx.doi.org/10.1016/j.psychres.2020.112937. Exclusion reason: Pharmacological study.

Mayhew, E., Stuttard, L., & Beresford, B. (2021). An Assessment of the Psychometric Properties of the GHQ-12 in an English Population of Autistic Adults Without Learning Difficulties. *Journal of Autism & Developmental Disorders, 51*(4), 1093-1106. doi:https://dx.doi.org/10.1007/s10803-020-04604-2. Exclusion reason: Not relevant measurement tool.

Mazefsky, C. A., Anderson, R., Conner, C. M., & Minshew, N. (2011). Child Behavior Checklist Scores for School-Aged Children with Autism: Preliminary Evidence of Patterns Suggesting the Need for Referral. *Journal of Psychopathology & Behavioral Assessment, 33*(1), 31-37. Exclusion reason: No psychometric information.

Mazefsky, C. A., Conner, C. M., & Oswald, D. P. (2010). Association between depression and anxiety in high-functioning children with autism spectrum disorders and maternal mood symptoms. *Autism research : Official Journal of the International Society for Autism Research, 3*(3), 120-127. doi:https://dx.doi.org/10.1002/aur.133. Exclusion reason: No psychometric information.

Mazefsky, C. A., Kao, J., & Oswald, D. P. (2011). Preliminary evidence suggesting caution in the use of psychiatric self-report measures with adolescents with high-functioning autism spectrum disorders. *Research in Autism Spectrum Disorders, 5*(1), 164-174. Exclusion reason: Not relevant measurement tool.

Mazefsky, C. A., Schreiber, D. R., Olino, T. M., & Minshew, N. J. (2014). The association between emotional and behavioral problems and gastrointestinal symptoms among children with high-functioning autism. *Autism, 18*(5), 493-501. doi:https://dx.doi.org/10.1177/1362361313485164. Exclusion reason: No psychometric information.

Mazurek, M. O., & Kanne, S. M. (2010). Friendship and internalizing symptoms among children and adolescents with ASD. *Journal of Autism and Developmental Disorders, 40*(12), 1512-1520. doi:https://dx.doi.org/10.1007/s10803-010-1014-y. Exclusion reason: No psychometric information.

Mazurek, M. O., Keefer, A., Shui, A., & Vasa, R. A. (2014). One-year course and predictors of abdominal pain in children with autism spectrum disorders: The role of anxiety and sensory over-responsivity. *Research in Autism Spectrum Disorders, 8(11)*, 1508-1515. doi:http://dx.doi.org/10.1016/j.rasd.2014.07.018. Exclusion reason: No psychometric information.

Mazurek, M. O., Lu, F., Macklin, E. A., & Handen, B. L. (2019). Factors associated with DSM-5 severity level ratings for autism spectrum disorder. *Autism, 23*(2), 468-476. doi:https://dx.doi.org/10.1177/1362361318755318. Exclusion reason: No psychometric information.

Mazurek, M. O., & Petroski, G. F. (2015). Sleep problems in children with autism spectrum disorder: examining the contributions of sensory over-responsivity and anxiety. *Sleep Medicine, 16*(2), 270-279. doi:https://dx.doi.org/10.1016/j.sleep.2014.11.006. Exclusion reason: No psychometric information.

Mazzone, L., Postorino, V., De Peppo, L., Fatta, L., Lucarelli, V., Reale, L., . . . Vicari, S. (2013). Mood symptoms in children and adolescents with autism spectrum disorders. *Research in Developmental Disabilities, 34*(11), 3699-3708. doi:https://dx.doi.org/10.1016/j.ridd.2013.07.034. Exclusion reason: No psychometric information.

McCarthy, J., Chaplin, E., Underwood, L., Forrester, A., Mills, R., & Murphy, D. (2019). Vulnerabilities of prisoners with autistic traits. *Australian and New Zealand Journal of Psychiatry, 53(Supplement 1)*, 118. doi:http://dx.doi.org/10.1177/0004867419836919. Exclusion reason: Conference abstract.

McCauley, J. B., Elias, R., & Lord, C. (2020). Trajectories of co-occurring psychopathology symptoms in autism from late childhood to adulthood. *Development and psychopathology, 32*(4), 1287-1302. doi:https://dx.doi.org/10.1017/S0954579420000826. Exclusion reason: No psychometric information.

McCormick, C. E. B., Kavanaugh, B. C., Sipsock, D., Righi, G., Oberman, L. M., Moreno De Luca, D., . . . Morrow, E. M. (2020). Autism Heterogeneity in a Densely Sampled U.S. Population: Results From the First 1,000 Participants in the RI-CART Study. *Autism research : Official Journal of the International Society for Autism Research, 13*(3), 474-488. doi:https://dx.doi.org/10.1002/aur.2261. Exclusion reason: No psychometric information.

McCormick, C. E. B., Kavanaugh, B. C., Sipsock, D., Righi, G., Oberman, L. M., Moreno De Luca, D., . . . Morrow, E. M. (2020). Autism Heterogeneity in a Densely Sampled U.S. Population: Results From the First 1,000 Participants in the RI-CART Study. *Autism Research, 13(3)*, 474-488. doi:http://dx.doi.org/10.1002/aur.2261. Exclusion reason: Not relevant measurement tool.

McCrae, C. S., Chan, W. S., Deroche, C. B., Munoz, M., McLean, D., Davenport, M., . . . Mazurek, M. (2018). Cbt for insomnia in children with autism spectrum disorder (ASD). *Sleep, 41(Supplement 1)*, A298. Exclusion reason: Conference abstract.

McDonnell, C. G., DeLucia, E. A., Hayden, E. P., Anagnostou, E., Nicolson, R., Kelley, E., . . . Stevenson, R. A. (2020). An exploratory analysis of predictors of youth suicide-related behaviors in autism spectrum disorder: Implications for prevention science. *Journal of Autism and Developmental Disorders, 50*(10), 3531-3544. doi:https://dx.doi.org/10.1007/s10803-019-04320-6. Exclusion reason: No psychometric information.

McGinley, J. (2016). Why does "clumsiness" matter? motor impairment, function, and emotional behavioral symptoms in autism spectrum disorders. *Journal of the American Academy of Child and Adolescent Psychiatry, 55(10 Supplement 1)*, S265. doi:http://dx.doi.org/10.1016/j.jaac.2016.07.146. Exclusion reason: Conference abstract.

McGrew, S., Malow, B. A., Henderson, L., Wang, L., Song, Y., & Stone, W. L. (2007). Developmental and behavioral questionnaire for autism spectrum disorders. *Pediatric Neurology, 37*(2), 108-116. Exclusion reason: Not relevant measurement tool.

McKenzie, R., Dallos, R., Stedmon, J., Hancocks, H., Vickery, P. J., Barton, A., . . . Ewings, P. (2020). SAFE, a new therapeutic intervention for families of children with autism: a randomised controlled feasibility trial. *BMJ Open, 10*(12), e038411. doi:https://dx.doi.org/10.1136/bmjopen-2020-038411. Exclusion reason: No psychometric information.

McRae, E. M., Stoppelbein, L., O'Kelley, S. E., Fite, P., & Greening, L. (2018). Predicting Internalizing and Externalizing Symptoms in Children with ASD: Evaluation of a Contextual Model of Parental Factors. *Journal of Autism & Developmental Disorders, 48*(4), 1261-1271. doi:https://dx.doi.org/10.1007/s10803-017-3368-x. Exclusion reason: No psychometric information.

McRae, E. M., Stoppelbein, L., O'Kelley, S. E., Fite, P., & Greening, L. (2019). Predicting child behavior: A comparative analysis between Autism Spectrum Disorder and Attention Deficit/Hyperactivity Disorder. *Journal of Child and Family Studies, 28*(3), 668-683. doi:https://dx.doi.org/10.1007/s10826-018-1299-6. Exclusion reason: Non-ASD sample.

McTiernan, A., Leader, G., Healy, O., & Mannion, A. (2011). Analysis of risk factors and early predictors of challenging behavior for children with autism spectrum disorder. *Research in Autism Spectrum Disorders, 5*(3), 1215-1222. doi:https://dx.doi.org/10.1016/j.rasd.2011.01.009. Exclusion reason: No psychometric information.

Medda, J. E., Kitzerow, J., Schlitt, S., Berndt, K., Schwenck, C., Uhlmann, L., & Freitag, C. M. (2021). Pre-Post Effects of the Psychoeducational, Autism-Specific Parent Training FAUT-E. *Zeitschrift fur Kinder-und Jugendpsychiatrie und Psychotherapie, 49*(2), 134-143. doi:https://dx.doi.org/10.1024/1422-4917/a000781. Exclusion reason: Effect study.

Medeiros, K., Kozlowski, A. M., Beighley, J. S., Rojahn, J., & Matson, J. L. (2012). The effects of developmental quotient and diagnostic criteria on challenging behaviors in toddlers with developmental disabilities. *Research in Developmental Disabilities, 33*(4), 1110-1116. doi:https://dx.doi.org/10.1016/j.ridd.2012.02.005. Exclusion reason: Non-ASD sample.

Melville, C. A., Cooper, S.-A., Morrison, J., Smiley, E., Allan, L., Jackson, A., . . . Mantry, D. (2008). The prevalence and incidence of mental ill-health in adults with autism and intellectual disabilities. *Journal of Autism and Developmental Disorders, 38*(9), 1676-1688. doi:https://dx.doi.org/10.1007/s10803-008-0549-7. Exclusion reason: No psychometric information.

Mensi, M. M., Gasparini, L., Chiappedi, M., Guerini, F. R., Orlandi, M., Rogantini, C., & Balottin, U. (2018). Empathy and behavior in children affected by Autism Spectrum Disorders. *Minerva Pediatrica, 02*, 02. doi:https://dx.doi.org/10.23736/S0026-4946.18.05228-3. Exclusion reason: No psychometric information.

Merrick, H., King, C., McConachie, H., Parr, J. R., & Le Couteur, A. (2020). Experience of transfer from child to adult mental health services of young people with autism spectrum disorder. *BJPsych Open Vol 6 2020, ArtID e58, 6*. doi:https://dx.doi.org/10.1192/bjo.2020.41. Exclusion reason: No psychometric information.

Midouhas, E., Yogaratnam, A., Flouri, E., & Charman, T. (2013). Psychopathology trajectories of children with autism spectrum disorder: the role of family poverty and parenting. *Journal of the American Academy of Child & Adolescent Psychiatry, 52*(10), 1057-1065.e1051. doi:https://dx.doi.org/10.1016/j.jaac.2013.07.011. Exclusion reason: No psychometric information.

Miranda, A., Berenguer, C., Rosello, B., & Baixauli, I. (2020). Relationships between the social communication questionnaire and pragmatic language, socialization skills, and behavioral problems in children with autism spectrum disorders. *Applied Neuropsychology. Child, 9*(2), 141-152. doi:https://dx.doi.org/10.1080/21622965.2018.1550403. Exclusion reason: No psychometric information.

Mire, S. S., Anderson, J. R., Manis, J. K., Hughes, K., Raff, N. S., & Goin-Kochel, R. P. (2018). Using teacher ratings to investigate developmental regression as a potential indicator of school-age symptoms in students with autism spectrum disorder. *School Mental Health: A Multidisciplinary Research and Practice Journal, 10*(1), 77-90. doi:https://dx.doi.org/10.1007/s12310-018-9251-x. Exclusion reason: No psychometric information.

Mirenda, P., Smith, I. M., Vaillancourt, T., Georgiades, S., Duku, E., Szatmari, P., . . . Zwaigenbaum, L. (2010). Validating the repetitive behavior scale-revised in young children with autism spectrum disorder. *Journal of Autism and Developmental Disorders, 40(12)*, 1521-1530. doi:http://dx.doi.org/10.1007/s10803-010-1012-0. Exclusion reason: No psychometric information.

Montazeri, F., de Bildt, A., Dekker, V., & Anderson, G. M. (2019). Network Analysis of Anxiety in the Autism Realm. *Journal of Autism & Developmental Disorders, 49*(6), 2219-2230. doi:https://dx.doi.org/10.1007/s10803-018-3474-4. Exclusion reason: Not relevant measurement tool.

Morrier, M. J., Ousley, O. Y., Caceres-Gamundi, G. A., Segall, M. J., Cubells, J. F., Young, L. J., & Andari, E. (2017). Brief Report: Relationship Between ADOS-2, Module 4 Calibrated Severity Scores (CSS) and Social and Non-Social Standardized Assessment Measures in Adult Males with Autism Spectrum Disorder (ASD). *Journal of Autism & Developmental Disorders, 47*(12), 4018-4024. doi:https://dx.doi.org/10.1007/s10803-017-3293-z. Exclusion reason: Not relevant measurement tool.

Mount, R. H., Hastings, R. P., Reilly, S., Cass, H., & Charman, T. (2003). Towards a behavioral phenotype for Rett syndrome. *American Journal of Mental Retardation, 108*(1), 1-12. Exclusion reason: Reviews/theoretical/non-empirical/not original data.

Mughal, R., Wong, S. S., Dimitriou, D., & Halstead, E. (2021). Nightmares in Children with Foetal Alcohol Spectrum Disorders, Autism Spectrum Disorders, and Their Typically Developing Peers. *Clocks & Sleep, 3*(3), 465-481. doi:https://dx.doi.org/10.3390/clockssleep3030033. Exclusion reason: No psychometric information.

Mukaddes, N. M., & Fateh, R. (2010). High rates of psychiatric co-morbidity in individuals with Asperger's disorder. *World Journal of Biological Psychiatry, 11*(2 Pt 2), 486-492. doi:https://dx.doi.org/10.1080/15622970902789130. Exclusion reason: No psychometric information.

Mukaddes, N. M., Hergner, S., & Tanidir, C. (2010). Psychiatric disorders in individuals with high-functioning autism and Asperger's disorder: Similarities and differences. *World Journal of Biological Psychiatry, 11(8)*, 964-971. doi:http://dx.doi.org/10.3109/15622975.2010.507785. Exclusion reason: No psychometric information.

Mukaddes, N. M., Herguner, S., & Tanidir, C. (2010). Psychiatric disorders in individuals with high-functioning autism and Asperger's disorder: similarities and differences. *World Journal of Biological Psychiatry, 11*(8), 964-971. doi:https://dx.doi.org/10.3109/15622975.2010.507785. Exclusion reason: No psychometric information.

Munesue, T., Ono, Y., Mutoh, K., Shimoda, K., Nakatani, H., & Kikuchi, M. (2008). High prevalence of bipolar disorder comorbidity in adolescents and young adults with high-functioning autism spectrum disorder: a preliminary study of 44 outpatients. *Journal of Affective Disorders, 111*(2-3), 170-175. doi:https://dx.doi.org/10.1016/j.jad.2008.02.015. Exclusion reason: No psychometric information.

Muniandy, M., Richdale, A. L., Arnold, S. R. C., Trollor, J. N., & Lawson, L. P. (2021). Factor structure and psychometric properties of the Brief COPE in autistic older adolescents and adults. *Research in Autism Spectrum Disorders, 84 (no pagination)*(101764). doi:http://dx.doi.org/10.1016/j.rasd.2021.101764. Exclusion reason: Not relevant measurement tool.

Munkhaugen, E. K., Torske, T., Gjevik, E., Naerland, T., Pripp, A. H., & Diseth, T. H. (2019). Individual characteristics of students with autism spectrum disorders and school refusal behavior. *Autism, 23*(2), 413-423. doi:https://dx.doi.org/10.1177/1362361317748619. Exclusion reason: No psychometric information.

Muratori, F., Narzisi, A., & Idia, G. (2014). Exploratory study describing 6 month outcomes for young children with autism who receive treatment as usual in Italy. *Neuropsychiatric Disease & Treatment, 10*, 577-586. doi:https://dx.doi.org/10.2147/NDT.S58308. Exclusion reason: Effect study.

Muratori, F., Tonacci, A., Billeci, L., Catalucci, T., Igliozzi, R., Calderoni, S., & Narzisi, A. (2017). Olfactory Processing in Male Children with Autism: Atypical Odor Threshold and Identification. *Journal of Autism & Developmental Disorders, 47*(10), 3243-3251. doi:https://dx.doi.org/10.1007/s10803-017-3250-x. Exclusion reason: No psychometric information.

Muratori, F., Tonacci, A., Billeci, L., Catalucci, T., Igliozzi, R., Calderoni, S., & Narzisi, A. (2017). "Olfactory processing in male children with autism: Atypical odor threshold and identification": Erratum. *Journal of Autism and Developmental Disorders, 47*(10), 3252. doi:https://dx.doi.org/10.1007/s10803-017-3291-1. Exclusion reason: No psychometric information.

Muratori, F., Turi, M., Prosperi, M., Narzisi, A., Valeri, G., Guerrera, S., . . . Vicari, S. (2019). Parental Perspectives on Psychiatric Comorbidity in Preschoolers With Autism Spectrum Disorders Receiving Publicly Funded Mental Health Services. *Frontiers in Psychiatry Frontiers Research Foundation, 10*, 107. doi:https://dx.doi.org/10.3389/fpsyt.2019.00107. Exclusion reason: No psychometric information.

Murphy, J., Zlomke, K., VanOrmer, J., & Swingle, H. (2020). Impact of Disruptive Behavior in Childhood Feeding Difficulties. *Journal of Clinical Psychology in Medical Settings, 27(2)*, 406-415. doi:http://dx.doi.org/10.1007/s10880-019-09646-y. Exclusion reason: No psychometric information.

Murphy, O., Healy, O., & Leader, G. (2009). Risk factors for challenging behaviors among 157 children with autism spectrum disorder in Ireland. *Research in Autism Spectrum Disorders, 3(2)*, 474-482. doi:http://dx.doi.org/10.1016/j.rasd.2008.09.008. Exclusion reason: No psychometric information.

Muskett, A., Capriola-Hall, N., Radtke, S., Factor, R., & Scarpa, A. (2019). Repetitive behaviors in Autism Spectrum Disorder: Associations with depression and anxiety symptoms. *Research in Autism Spectrum Disorders Vol 68 2019, ArtID 101449, 68*. doi:https://dx.doi.org/10.1016/j.rasd.2019.101449. Exclusion reason: No psychometric information.

Muskett, A., Capriola-Hall, N. N., Radtke, S. R., Factor, R., & Scarpa, A. (2019). Repetitive behaviors in Autism Spectrum Disorder: Associations with depression and anxiety symptoms. *Research in Autism Spectrum Disorders, 68 (no pagination)*(101449). doi:http://dx.doi.org/10.1016/j.rasd.2019.101449. Exclusion reason: No psychometric information.

Mutluer, T., Doenyas, C., & Aslan Genc, H. (2020). Behavioral Implications of the Covid-19 Process for Autism Spectrum Disorder, and Individuals' Comprehension of and Reactions to the Pandemic Conditions. *Frontiers in psychiatry Frontiers Research Foundation, 11*, 561882. doi:https://dx.doi.org/10.3389/fpsyt.2020.561882. Exclusion reason: No psychometric information.

Mutluer, T., Karakoc Demirkaya, S., & Abali, O. (2016). Assessment of sleep problems and related risk factors observed in Turkish children with Autism spectrum disorders. *Autism research : Official Journal of the International Society for Autism Research, 9*(5), 536-542. doi:https://dx.doi.org/10.1002/aur.1542. Exclusion reason: No psychometric information.

Myrbakk, E. (2015). Associations between challenging behaviour and mental disorders in individuals with intellectual disability and autism spectrum disorders. *Journal of Intellectual Disability Research, 1)*, 105. doi:http://dx.doi.org/10.1111/jir.12214. Exclusion reason: Conference abstract.

Nasca, B. C., Lopata, C., Donnelly, J. P., Rodgers, J. D., & Thomeer, M. L. (2020). Sex Differences in Externalizing and Internalizing Symptoms of Children with ASD. *Journal of Autism and Developmental Disorders, 50(9)*, 3245-3252. doi:http://dx.doi.org/10.1007/s10803-019-04132-8. Exclusion reason: No psychometric information.

Nasr Esfahani, F., Hakim Shooshtari, M., Shirmohammadi Sosfadi, R., Saeed, F., Jalai, F., Farsham, A., & Bidaki, R. (2018). Internalizing and Externalizing Problems, Empathy Quotient, and Systemizing Quotient in 4 to 11 Years-Old Siblings of Children with Autistic Spectrum Disorder Compared to Control Group. *Iranian Journal of Psychiatry, 13*(3), 191-199. Exclusion reason: Non-ASD sample.

Neuhaus, E., Bernier, R., & Beauchaine, T. P. (2014). Brief report: Social skills, internalizing and externalizing symptoms, and respiratory sinus arrhythmia in autism. *Journal of Autism and Developmental Disorders, 44*(3), 730-737. doi:https://dx.doi.org/10.1007/s10803-013-1923-7. Exclusion reason: Sample size = < 20.

Neuhaus, E., Bernier, R. A., Tham, S. W., & Webb, S. J. (2018). Gastrointestinal and psychiatric symptoms among children and adolescents with autism spectrum disorder. *Frontiers in Psychiatry Vol 9 2018, ArtID 515, 9*. doi:https://dx.doi.org/10.3389/fpsyt.2018.00515. Exclusion reason: No psychometric information.

Neuhaus, E., Webb, S. J., & Bernier, R. A. (2019). Linking social motivation with social skill: The role of emotion dysregulation in autism spectrum disorder. *Development and psychopathology, 31*(3), 931-943. doi:https://dx.doi.org/10.1017/S0954579419000361. Exclusion reason: No psychometric information.

Ni, H. C., Lin, H. Y., Tseng, W. I., Chiu, Y. N., Wu, Y. Y., Tsai, W. C., & Gau, S. S. (2018). Neural correlates of impaired self-regulation in male youths with autism spectrum disorder: A voxel-based morphometry study. *Progress in Neuro-Psychopharmacology & Biological Psychiatry, 82*, 233-241. doi:https://dx.doi.org/10.1016/j.pnpbp.2017.11.008. Exclusion reason: No psychometric information.

Niemczyk, J., Fischer, R., Wagner, C., Burau, A., Link, T., & Von Gontard, A. (2018). Ental psychological problems and parental stress in children with autism spectrum disorder. *Journal of Intellectual Disability Research, 62(8)*, 670. doi:http://dx.doi.org/10.1111/jir.12512. Exclusion reason: Conference abstract.

Niemczyk, J., Fischer, R., Wagner, C., Burau, A., Link, T., & von Gontard, A. (2019). Detailed assessment of incontinence, psychological problems and parental stress in children with autism spectrum disorder. *Journal of Autism and Developmental Disorders, 49*(5), 1966-1975. doi:https://dx.doi.org/10.1007/s10803-019-03885-6. Exclusion reason: No psychometric information.

Nonweiler, J., Rattray, F., Baulcomb, J., Happe, F., & Absoud, M. (2020). Prevalence and Associated Factors of Emotional and Behavioural Difficulties during COVID-19 Pandemic in Children with Neurodevelopmental Disorders. *Children, 7*(9), 04. doi:https://dx.doi.org/10.3390/children7090128. Exclusion reason: No psychometric information.

Noordhof, A., Krueger, R. F., Ormel, J., Oldehinkel, A. J., & Hartman, C. A. (2015). Integrating autism-related symptoms into the dimensional internalizing and externalizing model of psychopathology. The TRAILS study. *Journal of Abnormal Child Psychology, 43*(3), 577-587. doi:https://dx.doi.org/10.1007/s10802-014-9923-4. Exclusion reason: Non-ASD sample.

Nordahl, C. W., Iosif, A.-M., Young, G. S., Hechtman, A., Heath, B., Lee, J. K., . . . Ozonoff, S. (2020). High psychopathology subgroup in young children with autism: Associations with biological sex and amygdala volume. *Journal of the American Academy of Child & Adolescent Psychiatry, 59*(12), 1353-1363. doi:https://dx.doi.org/10.1016/j.jaac.2019.11.022. Exclusion reason: No psychometric information.

Nylander, L., Holmqvist, M., Gustafson, L., & Gillberg, C. (2013). Attention-deficit/hyperactivity disorder (ADHD) and autism spectrum disorder (ASD) in adult psychiatry. A 20-year register study. *Nordic Journal of Psychiatry, 67*(5), 344-350. doi:https://dx.doi.org/10.3109/08039488.2012.748824. Exclusion reason: No psychometric information.

O'Brien, M. J., Pelzel, K. E., Hendrix, N. M., Schieltz, K. M., Miller, K., Call, N. A., . . . Lindgren, S. D. (2021). Parent Ratings of Generalized and Indirect Effects of Functional Communication Training for Children with Autism Spectrum Disorder. *Behavior Modification*, 1454455211018815. doi:https://dx.doi.org/10.1177/01454455211018815. Exclusion reason: Effect study

O'Donnell, S., Deitz, J., Kartin, D., Nalty, T., & Dawson, G. (2012). Sensory processing, problem behavior, adaptive behavior, and cognition in preschool children with autism spectrum disorders. *American Journal of Occupational Therapy, 66*(5), 586-594. doi:https://dx.doi.org/10.5014/ajot.2012.004168. Exclusion reason: No psychometric information.

Okada, Y., Miyawaki, D., Kusaka, H., & Inoue, K. (2016). Bipolar disorder in children with autism spectrum disorder: Clinical study of three-year follow-up. *Journal of the American Academy of Child and Adolescent Psychiatry, 55(10 Supplement 1)*, S103-S104. doi:http://dx.doi.org/10.1016/j.jaac.2016.09.015. Exclusion reason: Conference abstract.

Ooi, Y. P., Rescorla, L., Sung, M., Fung, D. S., Woo, B., & Ang, R. P. (2014). Comparisons between autism spectrum disorders and anxiety disorders: findings from a clinic sample in Singapore. *Asia-Pacific psychiatry : Official Journal of the Pacific Rim College of Psychiatrists, 6*(1), 46-53. doi:https://dx.doi.org/10.1111/j.1758-5872.2012.00228.x. Exclusion reason: No psychometric information.

Ooi, Y. P., Tan, Z. J., Lim, C. X., Goh, T. J., & Sung, M. (2011). Prevalence of behavioural and emotional problems in children with high-functioning autism spectrum disorders. *Australian & New Zealand Journal of Psychiatry, 45*(5), 370-375. doi:https://dx.doi.org/10.3109/00048674.2010.534071. Exclusion reason: No psychometric information.

Operto, F. F., Pastorino, G. M. G., Scuoppo, C., Padovano, C., Vivenzio, V., Pistola, I., . . . Coppola, G. (2021). Adaptive Behavior, Emotional/Behavioral Problems and Parental Stress in Children With Autism Spectrum Disorder. *Frontiers in Neuroscience, 15*, 751465. doi:https://dx.doi.org/10.3389/fnins.2021.751465. Exclusion reason: No psychometric information.

Orinstein, A., Tyson, K. E., Suh, J., Troyb, E., Helt, M., Rosenthal, M., . . . Fein, D. A. (2015). Psychiatric symptoms in youth with a history of autism and optimal outcome. *Journal of Autism and Developmental Disorders, 45*(11), 3703-3714. doi:https://dx.doi.org/10.1007/s10803-015-2520-8. Exclusion reason: No psychometric information.

Osborne, L. A., McHugh, L., Saunders, J., & Reed, P. (2008). The effect of parenting behaviors on subsequent child behavior problems in autistic spectrum conditions. *Research in Autism Spectrum Disorders, 2*(2), 249-263. doi:https://dx.doi.org/10.1016/j.rasd.2007.06.004. Exclusion reason: No psychometric information.

Oubrahim, L., & Combalbert, N. (2019). Frequency and origin (reactive/proactive) of aggressive behavior in young people with intellectual disability and autism spectrum disorder. *International Journal of Developmental Disabilities, 67*(3), 209-216. doi:https://dx.doi.org/10.1080/20473869.2019.1640972. Exclusion reason: No psychometric information.

Overall, J. E., & Campbell, M. (1988). Behavioral assessment of psychopathology in children: infantile autism. *Journal of Clinical Psychology, 44*(5), 708-716. Exclusion reason: Not relevant measurement tool..

Owens, G., Granader, Y., Humphrey, A., & Baron-Cohen, S. (2008). LEGO therapy and the social use of language programme: An evaluation of two social skills interventions for children with high functioning autism and Asperger syndrome. *Journal of Autism and Developmental Disorders, 38*(10), 1944-1957. doi:https://dx.doi.org/10.1007/s10803-008-0590-6. Exclusion reason: Sample size = < 20.

Ozsivadjian, A., Hollocks, M. J., Magiati, I., Happe, F., Baird, G., & Absoud, M. (2021). Is cognitive inflexibility a missing link? The role of cognitive inflexibility, alexithymia and intolerance of uncertainty in externalising and internalising behaviours in young people with autism spectrum disorder. *Journal of Child Psychology and Psychiatry, 62*(6), 715-724. doi:https://dx.doi.org/10.1111/jcpp.13295. Exclusion reason: No psychometric information.

Ozturk, O., Erermis, S., Ercan, E. S., Gulen, F., Basay, B. K., Basay, O., . . . Aydin, C. (2016). Does the quality of life in autism spectrum disorder differ from other chronic disorders and healthy children? *Anadolu Psikiyatri Dergisi, 17*(5), 419-426. doi:https://dx.doi.org/10.5455/apd.185269. Exclusion reason: No psychometric information.

Ozturk, O., Erermis, S., Ercan, E. S., Gulen, F., Kabukcu Basay, B., Basay, O., . . . Aydin, C. (2016). Does the quality of life in autism spectrum disorder differ from other chronic disorders and healthy children? *Anadolu Psikiyatri Dergisi, 17(5)*, 419-426. doi:http://dx.doi.org/10.5455/apd.185269. Exclusion reason: No psychometric information.

Ozturk, O., Erermis, S., Ozbaran, B., Gulen, F., Kose, S., Kabukcu Basay, B., . . . Aydin, C. (2012). Does the quality of life of the children with ASD differ from that of other chronic disorders? *Neuropsychiatrie de l'Enfance et de l'Adolescence, 1)*, S210. doi:http://dx.doi.org/10.1016/j.neurenf.2012.04.446. Exclusion reason: Conference abstract.

Pahnke, J., Lundgren, T., Hursti, T., & Hirvikoski, T. (2014). Outcomes of an acceptance and commitment therapy-based skills training group for students with high-functioning autism spectrum disorder: A quasi-experimental pilot study. *Autism, 18*(8), 953-964. doi:https://dx.doi.org/10.1177/1362361313501091. Exclusion reason: Sample size = < 20.

Palka, T., Beresford, C., Peura, C., Kaplan, D., Verdi, M., Siegel, M., . . . Williams, D. (2018). Sleep Problems and Their Relationship to Maladaptive Behavior Severity in Psychiatrically Hospitalized Children with Autism Spectrum Disorder (ASD). *Journal of Autism and Developmental Disorders, 48(11)*, 3720-3726. doi:http://dx.doi.org/10.1007/s10803-017-3362-3. Exclusion reason: No psychometric information.

Papadopoulos, N., McGinley, J., Tonge, B., Bradshaw, J., Saunders, K., Murphy, A., & Rinehart, N. (2012). Motor proficiency and emotional/behavioural disturbance in autism and Asperger's disorder: another piece of the neurological puzzle? *Autism, 16*(6), 627-640. doi:https://dx.doi.org/10.1177/1362361311418692. Exclusion reason: No psychometric information.

Park, C. J., Yelland, G. W., Taffe, J. R., & Gray, K. M. (2012). Brief report: The relationship between language skills, adaptive behavior, and emotional and behavior problems in pre-schoolers with autism. *Journal of Autism & Developmental Disorders, 42*(12), 2761-2766. doi:https://dx.doi.org/10.1007/s10803-012-1534-8. Exclusion reason: No psychometric information.

Park, J. H., Kim, Y.-S., Koh, Y.-J., Song, J., & Leventhal, B. L. (2014). A contrast of comorbid condition and adaptive function between children with Autism Spectrum Disorder from clinical and non-clinical populations. *Research in Autism Spectrum Disorders, 8*(11), 1471-1481. doi:https://dx.doi.org/10.1016/j.rasd.2014.07.014. Exclusion reason: No psychometric information.

Park, S., Cho, S.-C., Cho, I. H., Kim, B.-N., Kim, J.-W., Shin, M.-S., . . . Yoo, H. J. (2012). Sex differences in children with autism spectrum disorders compared with their unaffected siblings and typically developing children. *Research in Autism Spectrum Disorders, 6*(2), 861-870. doi:https://dx.doi.org/10.1016/j.rasd.2011.11.006. Exclusion reason: No psychometric information.

Park, S., Cho, S.-C., Cho, I. H., Kim, B.-N., Kim, J.-W., Shin, M.-S., . . . Yoo, H. J. (2012). Sleep problems and their correlates and comorbid psychopathology of children with autism spectrum disorders. *Research in Autism Spectrum Disorders, 6*(3), 1068-1072. doi:https://dx.doi.org/10.1016/j.rasd.2012.02.004. Exclusion reason: No psychometric information.

Park, S., Park, M.-H., Kim, H. J., & Yoo, H. J. (2013). Anxiety and depression symptoms in children with Asperger syndrome compared with attention-deficit/hyperactivity disorder and depressive disorder. *Journal of Child and Family Studies, 22*(4), 559-568. doi:https://dx.doi.org/10.1007/s10826-012-9611-3. Exclusion reason: No psychometric information.

Payakachat, N., Tilford, J. M., Kuhlthau, K. A., van Exel, N. J., Kovacs, E., Bellando, J., . . . Brouwer, W. B. (2014). Predicting health utilities for children with autism spectrum disorders. *Autism research : Official Journal of the International Society for Autism Research, 7*(6), 649-663. doi:https://dx.doi.org/10.1002/aur.1409. Exclusion reason: No psychometric information.

Payne, T. D. W. (2018). Social, emotional, and behavioral functioning for transitional-aged youth with Autism. *Dissertation Abstracts International: Section B: The Sciences and Engineering, 78*(10-B(E)), No Pagination Specified. Exclusion reason: PhD dissertation.

Pearson, D. A., Loveland, K. A., Lachar, D., Lane, D. M., Reddoch, S. L., Mansour, R., & Cleveland, L. A. (2006). A Comparison of Behavioral and Emotional Functioning in Children and Adolescents with Autistic Disorder and PDD-NOS. *Child Neuropsychology, 12*(4-5), 321-333. doi:https://dx.doi.org/10.1080/09297040600646847. Exclusion reason: No psychometric information.

Pedersen, K. A., Cook, W. L., Taylor, B., Siegel, M., Touchette, E., Peura, C., & Santangelo, S. L. (2020). 6.12 Examining Parenting Efficacy as a Mediator between Mother and Father Stress and Child Problem Behaviors in Families with a Child with Autism Spectrum Disorder: A Dyadic Structural Model. *Journal of the American Academy of Child and Adolescent Psychiatry, 59(10 Supplement)*, S163. doi:http://dx.doi.org/10.1016/j.jaac.2020.08.105. Exclusion reason: Conference abstract.

Pedersen, K. A., Santangelo, S. L., Gabriels, R. L., Righi, G., Erard, M., & Siegel, M. (2018). Behavioral Outcomes of Specialized Psychiatric Hospitalization in the Autism Inpatient Collection (AIC): A Multisite Comparison. *Journal of Autism & Developmental Disorders, 48*(11), 3658-3667. doi:https://dx.doi.org/10.1007/s10803-017-3366-z. Exclusion reason: No psychometric information.

Perrin, J. M., Coury, D. L., Hyman, S. L., Cole, L., Reynolds, A. M., & Clemons, T. (2012). Complementary and alternative medicine use in a large pediatric autism sample. *Pediatrics, 130 Suppl 2*, S77-82. doi:https://dx.doi.org/10.1542/peds.2012-0900E. Exclusion reason: No psychometric information.

Peters-Scheffer, N., Didden, R., & Korzilius, H. (2012). Maternal stress predicted by characteristics of children with autism spectrum disorder and intellectual disability. *Research in Autism Spectrum Disorders, 6*(2), 696-706. doi:https://dx.doi.org/10.1016/j.rasd.2011.10.003. Exclusion reason: No psychometric information.

Piergies, A. M. H., Hirota, T., Monden, R., & Zheng, S. (2021). Subgrouping School-Aged Children with Autism Spectrum Disorder Based on Co-Occurring Psychopathology. *medRxiv., 22*. doi:https://dx.doi.org/10.1101/2021.07.19.21260784. Exclusion reason: No psychometric information.

Pillay, M., Alderson-Day, B., Wright, B., Williams, C., & Urwin, B. (2011). Autism Spectrum Conditions--enhancing Nurture and Development (ASCEND): an evaluation of intervention support groups for parents. *Clinical Child Psychology & Psychiatry, 16*(1), 5-20. doi:https://dx.doi.org/10.1177/1359104509340945. Exclusion reason: Effect study.

Pinchover, S., & Shulman, C. (2019). Behavioural problems and playfulness of young children with ASD: The moderating role of a teacher's emotional availability. *Early Child Development and Care, 189*(14), 2252-2264. doi:https://dx.doi.org/10.1080/03004430.2018.1447934. Exclusion reason: Non-ASD sample.

Piper, B. J., Gray, H. M., Raber, J., & Birkett, M. A. (2014). Reliability and validity of Brief Problem Monitor, an abbreviated form of the Child Behavior Checklist. *Psychiatry & Clinical Neurosciences, 68*(10), 759-767. doi:https://dx.doi.org/10.1111/pcn.12188. Exclusion reason: Non-ASD sample.

Pisula, E., & Niedzwiecka, A. (2021). Symptoms of Autism, Comorbid Mental Health Conditions and Challenging Behaviors among Toddlers with Down Syndrome at Low Risk for ASD-Characterization Using the BISCUIT-Parts 1-3. *International Journal of Environmental Research & Public Health [Electronic Resource], 18*(20), 12. doi:https://dx.doi.org/10.3390/ijerph182010684. Exclusion reason: Not relevant measurement tool.

Pivovarciova, A., Durdiakova, J., Babinska, K., Kubranska, A., Vokalova, L., Minarik, G., . . . Ostatnikova, D. (2016). Testosterone and Androgen Receptor Sensitivity in Relation to Hyperactivity Symptoms in Boys with Autism Spectrum Disorders. *PLoS ONE [Electronic Resource], 11*(2), e0149657. doi:https://dx.doi.org/10.1371/journal.pone.0149657. Exclusion reason: No psychometric information.

Pivovarciova, A., Durdiakova, J., Hnilicova, S., & Ostatnikova, D. (2015). Are higher testosterone levels in autism related to problem behavior? *European Neuropsychopharmacology, 1)*, S83-S84. Exclusion reason: Conference abstract.

Plesa Skwerer, D., Joseph, R. M., Eggleston, B., Meyer, S. R., & Tager-Flusberg, H. (2019). Prevalence and Correlates of Psychiatric Symptoms in Minimally Verbal Children and Adolescents With ASD. *Frontiers in psychiatry Frontiers Research Foundation, 10*, 43. doi:https://dx.doi.org/10.3389/fpsyt.2019.00043. Exclusion reason: No psychometric information.

Poon, K. K. (2012). Challenging behaviors among children with autism spectrum disorders and multiple disabilities attending special schools in Singapore. *Research in Developmental Disabilities, 33*(2), 578-582. doi:https://dx.doi.org/10.1016/j.ridd.2011.10.025. Exclusion reason: No psychometric information.

Postorino, V., Fatta, L. M., De Peppo, L., Giovagnoli, G., Armando, M., Vicari, S., & Mazzone, L. (2015). Longitudinal comparison between male and female preschool children with autism spectrum disorder. *Journal of Autism and Developmental Disorders, 45*(7), 2046-2055. doi:https://dx.doi.org/10.1007/s10803-015-2366-0. Exclusion reason: No psychometric information.

Postorino, V., Sanges, V., Giovagnoli, G., Fatta, L. M., De Peppo, L., Armando, M., . . . Mazzone, L. (2015). Clinical differences in children with autism spectrum disorder with and without food selectivity. *Appetite, 92*, 126-132. doi:http://dx.doi.org/10.1016/j.appet.2015.05.016. Exclusion reason: No psychometric information.

Pozo, P., & Sarria, E. (2014). Prediction of stress in mothers of children with autism spectrum disorders. *The Spanish Journal of Psychology Vol 17 2014, ArtID E6, 17*. Exclusion reason: No psychometric information.

Prosperi, M., Turi, M., Guerrera, S., Napoli, E., Tancredi, R., Igliozzi, R., . . . Vicari, S. (2020). Sex Differences in Autism Spectrum Disorder: An Investigation on Core Symptoms and Psychiatric Comorbidity in Preschoolers. *Frontiers in Integrative Neuroscience, 14*, 594082. doi:https://dx.doi.org/10.3389/fnint.2020.594082. Exclusion reason: No psychometric information.

Qiu, J., Kong, X., Li, J., Yang, J., Huang, Y., Huang, M., . . . Kong, J. (2021). Transcranial Direct Current Stimulation (tDCS) over the Left Dorsal Lateral Prefrontal Cortex in Children with Autism Spectrum Disorder (ASD). *Neural Plasticity, 2021*, 6627507. doi:https://dx.doi.org/10.1155/2021/6627507. Exclusion reason: No psychometric information.

Quebles, I., Solomon, O., Smith, K. A., Rao, S. R., Lu, F., Azen, C., . . . Yin, L. (2020). Racial and Ethnic Differences in Behavioral Problems and Medication Use Among Children With Autism Spectrum Disorders. *American Journal on Intellectual & Developmental Disabilities, 125*(5), 369-388. doi:https://dx.doi.org/10.1352/1944-7558-125.5.369. Exclusion reason: Not relevant measurement tool.

Quek, L. H., Sofronoff, K., Sheffield, J., White, A., & Kelly, A. (2012). Co-occurring anger in young people with Asperger's syndrome. *Journal of Clinical Psychology, 68*(10), 1142-1148. doi:https://dx.doi.org/10.1002/jclp.21888. Exclusion reason: Not relevant measurement tool.

Rabeyron, T., Robledo Del Canto, J. P., Carasco, E., Bisson, V., Bodeau, N., Vrait, F. X., . . . Bonnot, O. (2020). A randomized controlled trial of 25 sessions comparing music therapy and music listening for children with autism spectrum disorder. *Psychiatry Research, 293*, 113377. doi:https://dx.doi.org/10.1016/j.psychres.2020.113377. Exclusion reason: No psychometric information.

Raddad, D., Chakroun, G., Abu-Hamada, S., Hassan, M., Saqer, L., & Amr, M. (2011). The impact of sociodemographic variables on the autistic symptoms and maladaptive behavior among a sample of Jordanian children with autistic disorder. *Arab Journal of Psychiatry, 22*(2), 126-132. Exclusion reason: No psychometric information.

Randall, M., Sciberras, E., Brignell, A., Ihsen, E., Efron, D., Dissanayake, C., & Williams, K. (2016). Autism spectrum disorder: Presentation and prevalence in a nationally representative Australian sample. *Australian and New Zealand Journal of Psychiatry, 50*(3), 243-253. doi:https://dx.doi.org/10.1177/0004867415595287. Exclusion reason: No psychometric information.

Ratcliffe, B., Wong, M., Dossetor, D., & Hayes, S. (2014). Teaching social-emotional skills to school-aged children with Autism Spectrum Disorder: A treatment versus control trial in 41 mainstream schools. *Research in Autism Spectrum Disorders, 8*(12), 1722-1733. doi:https://dx.doi.org/10.1016/j.rasd.2014.09.010. Exclusion reason: Effect study.

Ratcliffe, B., Wong, M., Dossetor, D., & Hayes, S. (2015). The association between social skills and mental health in school-aged children with autism spectrum disorder, with and without intellectual disability. *Journal of Autism and Developmental Disorders, 45*(8), 2487-2496. doi:https://dx.doi.org/10.1007/s10803-015-2411-z. Exclusion reason: No psychometric information.

Ratcliffe, B., Wong, M., Dossetor, D., & Hayes, S. (2019). Improving Emotional Competence in Children with Autism Spectrum Disorder and Mild Intellectual Disability in Schools: A Preliminary Treatment Versus Waitlist Study. *Behaviour Change, 36(4)*, 216-232. doi:http://dx.doi.org/10.1017/bec.2019.13. Exclusion reason: Effect study.

Rattaz, C., Michelon, C., & Baghdadli, A. (2015). Symptom severity as a risk factor for self-injurious behaviours in adolescents with autism spectrum disorders. *Journal of Intellectual Disability Research, 59*(8), 730-740. doi:https://dx.doi.org/10.1111/jir.12177. Exclusion reason: No psychometric information.

Rattaz, C., Michelon, C., Munir, K., & Baghdadli, A. (2018). Challenging behaviours at early adulthood in autism spectrum disorders: topography, risk factors and evolution. *Journal of Intellectual Disability Research, 62*(7), 637-649. doi:https://dx.doi.org/10.1111/jir.12503. Exclusion reason: No psychometric information.

Rattaz, C., Munir, K., Michelon, C., Picot, M. C., Baghdadli, A., Chabaux, C., . . . Vespirini, S. (2020). School Inclusion in Children and Adolescents with Autism Spectrum Disorders in France: Report from the ELENA French Cohort Study. *Journal of Autism and Developmental Disorders, 50(2)*, 455-466. doi:http://dx.doi.org/10.1007/s10803-019-04273-w. Exclusion reason: No psychometric information.

Rattaz, C., Munir, K., Michelon, C., Picot, M.-C., Baghdadli, A., Baghdadli, A., . . . Vespirini, S. (2020). School inclusion in children and adolescents with autism spectrum disorders in France: Report from the ELENA French cohort study. *Journal of Autism and Developmental Disorders, 50*(2), 455-466. doi:https://dx.doi.org/10.1007/s10803-019-04273-w. Exclusion reason: No psychometric information.

Rauf, N. K., Anis-ul-haq, M., & Khan, S. (2018). Association of autism child characteristics with maternal and paternal stress. *Rawal Medical Journal, 43(2)*, 263-266. Exclusion reason: Non-ASD sample.

Raulston, T. J., Kosty, D., & McIntyre, L. L. (2021). Mindful parenting, caregiver distress, and conduct problems in children with autism. *American journal on intellectual and developmental disabilities, 126*(5), 396-408. doi:https://dx.doi.org/10.1352/1944-7558-126.5.396. Exclusion reason: No psychometric information.

Reale, L., Guarnera, M., Ruta, L., & Mazzone, L. (2011). Depressive symptoms in Asperger Syndrome and high-functioning autism: Prevalence and correlation with global functioning. *European Child and Adolescent Psychiatry, 1)*, S148. doi:http://dx.doi.org/10.1007/s00787-011-0181-5. Exclusion reason: Conference abstract.

Reed, P., Howse, J., Ho, B., & Osborne, L. A. (2017). Relationship between perceived limit-setting abilities, autism spectrum disorder severity, behaviour problems and parenting stress in mothers of children with autism spectrum disorder. *Autism, 21*(8), 952-959. doi:https://dx.doi.org/10.1177/1362361316658775. Exclusion reason: No psychometric information.

Reed, P., Osborne, L. A., & Waddington, E. M. (2012). A comparative study of the impact of mainstream and special school placement on the behaviour of children with Autism Spectrum Disorders. *British Educational Research Journal, 38*(5), 749-763. doi:https://dx.doi.org/10.1080/01411926.2011.580048. Exclusion reason: No psychometric information.

Reynolds, K. C., Patriquin, M., Alfano, C. A., Loveland, K. A., & Pearson, D. A. (2017). Parent-Reported Problematic Sleep Behaviors in Children with Comorbid Autism Spectrum Disorder and Attention-Deficit/Hyperactivity Disorder. *Research in Autism Spectrum Disorders, 39*, 20-32. doi:https://dx.doi.org/10.1016/j.rasd.2017.04.003. Exclusion reason: No psychometric information.

Reynolds, S., Bendixen, R. M., Lawrence, T., & Lane, S. J. (2011). A pilot study examining activity participation, sensory responsiveness, and competence in children with high functioning Autism Spectrum Disorder. *Journal of Autism & Developmental Disorders, 41*(11), 1496-1506. doi:https://dx.doi.org/10.1007/s10803-010-1173-x. Exclusion reason: No psychometric information.

Reynolds, S., Lane, S. J., & Thacker, L. (2012). Sensory processing, physiological stress, and sleep behaviors in children with and without autism spectrum disorders. *OTJR Occupation, Participation and Health, 32(1)*, 246-257. doi:http://dx.doi.org/10.3928/15394492-20110513-02. Exclusion reason: No psychometric information.

Richards, C., Oliver, C., Nelson, L., & Moss, J. (2012). Self-injurious behaviour in individuals with autism spectrum disorder and intellectual disability. *Journal of Intellectual Disability Research, 56*(5), 476-489. doi:https://dx.doi.org/10.1111/j.1365-2788.2012.01537.x. Exclusion reason: No psychometric information.

Richman, D. M., Barnard-Brak, L., Bosch, A., Thompson, S., Grubb, L., & Abby, L. (2013). Predictors of self-injurious behaviour exhibited by individuals with autism spectrum disorder. *Journal of Intellectual Disability Research, 57*(5), 429-439. doi:https://dx.doi.org/10.1111/j.1365-2788.2012.01628.x. Exclusion reason: No psychometric information.

Ridderinkhof, A., Bruin, E. I., Blom, R., & Bogels, S. M. (2018). Mindfulness-based program for children with autism spectrum disorder and their parents: Direct and long-term improvements. *Mindfulness, 9*(3), 773-791. doi:https://dx.doi.org/10.1007/s12671-017-0815-x. Exclusion reason: Effect study.

Rieske, R. D., Matson, J. L., Beighley, J. S., Cervantes, P. E., Goldin, R. L., & Jang, J. (2015). Comorbid psychopathology rates in children diagnosed with autism spectrum disorders according to the DSM-IV-TR and the proposed DSM-5. *Developmental neurorehabilitation, 18*(4), 218-223. doi:https://dx.doi.org/10.3109/17518423.2013.790519. Exclusion reason: No psychometric information.

Rieske, R. D., Matson, J. L., Davis, T. E., 3rd, Konst, M. J., Williams, L. W., & Whiting, S. E. (2013). Examination and validation of a measure of anxiety specific to children with autism spectrum disorders. *Developmental neurorehabilitation, 16*(1), 9-16. doi:https://dx.doi.org/10.3109/17518423.2012.705909. Exclusion reason: No psychometric information.

Rivard, M., Chatenoud, C., Aldersey, H. M., Chiu, C. Y., Turnbull, A., Mestari, Z., . . . Cloran, P. (2019). From early intensive behavioural intervention to school: Assessing the quality of the service trajectory in families with a child with autism spectrum disorder. *Journal of Intellectual Disability Research, 63(7)*, 752. doi:http://dx.doi.org/10.1111/jir.12657. Exclusion reason: Conference abstract.

Rivera, P., Renziehausen, J., & Garcia, J. M. (2020). Effects of an 8-Week Judo Program on Behaviors in Children with Autism Spectrum Disorder: A Mixed-Methods Approach. *Child Psychiatry & Human Development, 51*(5), 734-741. doi:https://dx.doi.org/10.1007/s10578-020-00994-7. Exclusion reason: Effect study

Rodas, N. V., Eisenhower, A., & Blacher, J. (2017). Structural and pragmatic language in children with ASD: Longitudinal impact on anxiety and externalizing behaviors. *Journal of Autism and Developmental Disorders, 47*(11), 3479-3488. doi:https://dx.doi.org/10.1007/s10803-017-3265-3. Exclusion reason: No psychometric information.

Rojahn, J., Wilkins, J., Matson, J. L., & Boisjoli, J. (2010). A comparison of adults with intellectual disabilities with and without ASD on parallel measures of challenging behaviour: The Behavior Problems Inventory-01 (BPI-01) and Autism Spectrum Disorders-Behavior Problems for intellectually disabled adults (ASD-BPA). *Journal of Applied Research in Intellectual Disabilities, 23*(2), 179-185. Exclusion reason: Non-ASD sample.

Romero, M., Aguilar, J. M., Del-Rey-Mejias, A., Mayoral, F., Rapado, M., Pecina, M., . . . Lara, J. P. (2016). Psychiatric comorbidities in autism spectrum disorder: A comparative study between DSM-IV-TR and DSM-5 diagnosis. *International Journal of Clinical & Health Psychology, 16*(3), 266-275. doi:https://dx.doi.org/10.1016/j.ijchp.2016.03.001. Exclusion reason: No psychometric information.

Rosa, M., Puig, O., Lazaro, L., & Calvo, R. (2016). Socioeconomic status and intelligence quotient as predictors of psychiatric disorders in children and adolescents with high-functioning autism spectrum disorder and in their siblings. *Autism, 20*(8), 963-972. doi:https://dx.doi.org/10.1177/1362361315617881. Exclusion reason: No psychometric information.

Rosen, T. E., & Lerner, M. D. (2016). Externalizing and Internalizing Symptoms Moderate Longitudinal Patterns of Facial Emotion Recognition in Autism Spectrum Disorder. *Journal of Autism and Developmental Disorders, 46(8)*, 2621-2634. doi:http://dx.doi.org/10.1007/s10803-016-2800-y. Exclusion reason: Sample size = < 20.

Rosen, T. E., Spaulding, C. J., Gates, J. A., & Lerner, M. D. (2019). Autism severity, co-occurring psychopathology, and intellectual functioning predict supportive school services for youth with autism spectrum disorder. *Autism, 23*(7), 1805-1816. doi:https://dx.doi.org/10.1177/1362361318809690. Exclusion reason: No psychometric information

Rossow, T., MacLennan, K., & Tavassoli, T. (2021). The relationship between sensory reactivity differences and mental health symptoms in preschool-age autistic children. *Autism Research, 14*(8), 1645-1657. doi:https://dx.doi.org/10.1002/aur.2525. Exclusion reason: No psychometric information.

Ruppel, K. W., Hanley, G. P., Landa, R. K., & Rajaraman, A. (2021). An Evaluation of "Balance": a Home-Based, Parent-Implemented Program Addressing Emerging Problem Behavior. *Behavior Analysis in Practice, 14*(2), 324-341. doi:https://dx.doi.org/10.1007/s40617-020-00490-3. Exclusion reason: Sample size = < 20.

Russell, A. J., Murphy, C. M., Wilson, E., Gillan, N., Brown, C., Robertson, D. M., . . . Murphy, D. G. (2016). The mental health of individuals referred for assessment of autism spectrum disorder in adulthood: A clinic report. *Autism, 20*(5), 623-627. doi:https://dx.doi.org/10.1177/1362361315604271. Exclusion reason: Not relevant measurement tool.

Rzepecka, H., McKenzie, K., McClure, I., & Murphy, S. (2011). Sleep, anxiety and challenging behaviour in children with intellectual disability and/or autism spectrum disorder. *Research in Developmental Disabilities, 32*(6), 2758-2766. doi:https://dx.doi.org/10.1016/j.ridd.2011.05.034. Exclusion reason: Non-ASD sample.

Safer-Lichtenstein, J., & McIntyre, L. L. (2020). Comparing autism symptom severity between children with a medical autism diagnosis and an autism special education eligibility. *Focus on Autism and Other Developmental Disabilities, 35*(3), 186-192. doi:https://dx.doi.org/10.1177/1088357620922162. Exclusion reason: No psychometric information.

Sahoo, S. (2019). Parenting stress and behavioural problems among mothers of cerebral palsy and Autistic children. *Indian Journal of Psychiatry, 61(9 Supplement 3)*, S455. Exclusion reason: Conference abstract.

Salayev, K. A., & Sanne, B. (2017). The strengths and difficulties questionnaire (SDQ) in autism spectrum disorders. *International Journal on Disability and Human Development, 16*(3), 275-280. doi:https://dx.doi.org/10.1515/ijdhd-2016-0025. Exclusion reason: No psychometric information.

Salazar, F., Baird, G., Chandler, S., Tseng, E., O'Sullivan, T., Howlin, P., . . . Simonoff, E. (2015). Co-occurring psychiatric disorders in preschool and elementary school-aged children with autism spectrum disorder. *Journal of Autism and Developmental Disorders, 45*(8), 2283-2294. doi:https://dx.doi.org/10.1007/s10803-015-2361-5. Exclusion reason: No psychometric information.

Salehi, P., Herzig, L., Capone, G., Lu, A., Oron, A. P., & Kim, S. J. (2018). Comparison of Aberrant Behavior Checklist profiles across Prader-Willi syndrome, Down syndrome, and autism spectrum disorder. *American Journal of Medical Genetics. Part A, 176*(12), 2751-2759. doi:https://dx.doi.org/10.1002/ajmg.a.40665. Exclusion reason: No psychometric information.

Salomone, E., Leadbitter, K., Aldred, C., Barrett, B., Byford, S., Charman, T., . . . Slonims, V. (2018). The association between child and family characteristics and the mental health and wellbeing of caregivers of children with autism in mid-childhood. *Journal of Autism and Developmental Disorders, 48*(4), 1189-1198. doi:https://dx.doi.org/10.1007/s10803-017-3392-x. Exclusion reason: No psychometric information.

Salter, K., Beamish, W., & Davies, M. (2016). The effects of child-centered play therapy (CCPT) on the social and emotional growth of young Australian children with autism. *International Journal of Play Therapy, 25*(2), 78-90. doi:https://dx.doi.org/10.1037/pla0000012. Exclusion reason: Sample size = < 20.

Samson, A. C., Hardan, A. Y., Lee, I. A., Phillips, J. M., & Gross, J. J. (2015). Maladaptive behavior in autism spectrum disorder: The role of emotion experience and emotion regulation. *Journal of Autism and Developmental Disorders, 45*(11), 3424-3432. doi:https://dx.doi.org/10.1007/s10803-015-2388-7. Exclusion reason: No psychometric information.

Samson, A. C., Wells, W. M., Phillips, J. M., Hardan, A. Y., & Gross, J. J. (2015). Emotion regulation in autism spectrum disorder: evidence from parent interviews and children's daily diaries. *Journal of Child Psychology & Psychiatry & Allied Disciplines, 56*(8), 903-913. doi:https://dx.doi.org/10.1111/jcpp.12370. Exclusion reason: Not relevant measurement tool.

Sannar, E. M., Palka, T., Beresford, C., Peura, C., Kaplan, D., Verdi, M., . . . Developmental Disorders Inpatient Research, C. (2018). Sleep Problems and Their Relationship to Maladaptive Behavior Severity in Psychiatrically Hospitalized Children with Autism Spectrum Disorder (ASD). *Journal of Autism & Developmental Disorders, 48*(11), 3720-3726. doi:https://dx.doi.org/10.1007/s10803-017-3362-3. Exclusion reason: No psychometric information.

Sapmaz, D., Baykal, S., & Akbas, S. (2018). The Clinical Features of Comorbid Pediatric Bipolar Disorder in Children with Autism Spectrum Disorder. *Journal of Autism & Developmental Disorders, 48*(8), 2800-2808. doi:https://dx.doi.org/10.1007/s10803-018-3541-x. Exclusion reason: No psychometric information.

Sapra, S., & Kaushal, T. (2015). Behavioral and neuro-developmental profile of children with Autism Spectrum Disorder in tertiary care center. *Indian Journal of Psychiatry, 1)*, S151. Exclusion reason: Conference abstract.

Sawyer, M. G., Bittman, M., La Greca, A. M., Crettenden, A. D., Harchak, T. F., & Martin, J. (2010). Time demands of caring for children with autism: What are the implications for maternal mental health? *Journal of Autism and Developmental Disorders, 40*(5), 620-628. doi:https://dx.doi.org/10.1007/s10803-009-0912-3. Exclusion reason: No psychometric information.

Scheithauer, M., Call, N. A., Lomas Mevers, J., McCracken, C. E., & Scahill, L. (2021). A Feasibility Randomized Clinical Trial of a Structured Function-Based Intervention for Elopement in Children with Autism Spectrum Disorder. *Journal of Autism & Developmental Disorders, 51*(8), 2866-2875. doi:https://dx.doi.org/10.1007/s10803-020-04753-4. Exclusion reason: Sample size = < 20.

Schreck, K. A. (2021). Sleep quantity and quality as predictors of behavior and mental health issues for children and adolescents with autism. *Research in Autism Spectrum Disorders, 84 (no pagination)*(101767). doi:https://dx.doi.org/10.1016/j.rasd.2021.101767. Exclusion reason: No psychometric information.

Schwartzman, J. M., & Corbett, B. A. (2020). Higher depressive symptoms in early adolescents with Autism Spectrum Disorder by self- and parent-report compared to typically-developing peers. *Research in Autism Spectrum Disorders, 77*. doi:https://dx.doi.org/10.1016/j.rasd.2020.101613. Exclusion reason: No psychometric information.

Serur, Y., Sher-Censor, E., Sofrin-Frumer, D., Daon, K., Sobol-Havia, D., Weinberger, R., . . . Gothelf, D. (2022). Parental Expressed Emotion, Parenting Stress, and Behavioral Problems of Young Children with 22q11.2 Deletion Syndrome and Idiopathic Autism Spectrum Disorder. *Child Psychiatry & Human Development, 27*, 27. doi:https://dx.doi.org/10.1007/s10578-021-01310-7. Exclusion reason: No psychometric information.

Sesso, G., Cristofani, C., Berloffa, S., Cristofani, P., Fantozzi, P., Inguaggiato, E., . . . Masi, G. (2020). Autism Spectrum Disorder and Disruptive Behavior Disorders Comorbidities Delineate Clinical Phenotypes in Attention-Deficit Hyperactivity Disorder: Novel Insights from the Assessment of Psychopathological and Neuropsychological Profiles. *Journal of Clinical Medicine, 9*(12), 26. doi:https://dx.doi.org/10.3390/jcm9123839. Exclusion reason: No psychometric information.

Seymour, M., Wood, C., Giallo, R., & Jellett, R. (2013). Fatigue, stress and coping in mothers of children with an autism spectrum disorder. *Journal of Autism and Developmental Disorders, 43*(7), 1547-1554. doi:https://dx.doi.org/10.1007/s10803-012-1701-y. Exclusion reason: No psychometric information.

Shaffer, R. C., Wink, L. K., Ruberg, J., Pittenger, A., Adams, R., Sorter, M., . . . Erickson, C. A. (2019). Emotion Regulation Intensive Outpatient Programming: Development, Feasibility, and Acceptability. *Journal of Autism and Developmental Disorders, 49(2)*, 495-508. doi:http://dx.doi.org/10.1007/s10803-018-3727-2. Exclusion reason: Non-ASD sample.

Shahrivar, Z., Mahdavi, H., Tehrani-Doost, M., Fatholahi, Y., & Jahanitabesh, A. (2013). Behavioral profile of executive dysfunction in children and adolescents with autism spectrum disorder. *European Child and Adolescent Psychiatry, 1)*, S215-S216. doi:http://dx.doi.org/10.1007/s00787-013-0423-9. Exclusion reason: Conference abstract.

Sices, L., Pawlowski, K., Farfel, L., Phillips, D., Howe, Y., Cochran, D. M., . . . Bridgemohan, C. (2017). Feasibility of Conducting Autism Biomarker Research in the Clinical Setting. *Journal of Developmental & Behavioral Pediatrics, 38*(7), 483-492. doi:https://dx.doi.org/10.1097/DBP.0000000000000470. Exclusion reason: No psychometric information.

Sikora, D., Moran, E., Orlich, F., Hall, T. A., Kovacs, E. A., Delahaye, J., . . . Kuhlthau, K. (2013). The relationship between family functioning and behavior problems in children with autism spectrum disorders. *Research in Autism Spectrum Disorders, 7(2)*, 307-315. doi:http://dx.doi.org/10.1016/j.rasd.2012.09.006. Exclusion reason: No psychometric information.

Sikora, D. M., Johnson, K., Clemons, T., & Katz, T. (2012). The relationship between sleep problems and daytime behavior in children of different ages with autism spectrum disorders. *Pediatrics, 130 Suppl 2*, S83-90. doi:https://dx.doi.org/10.1542/peds.2012-0900F. Exclusion reason: No psychometric information.

Simonoff, E., Jones, C. R., Baird, G., Pickles, A., Happe, F., & Charman, T. (2013). The persistence and stability of psychiatric problems in adolescents with autism spectrum disorders. *Journal of Child Psychology & Psychiatry & Allied Disciplines, 54*(2), 186-194. doi:https://dx.doi.org/10.1111/j.1469-7610.2012.02606.x. Exclusion reason: No psychometric information.

Simonoff, E., Jones, C. R., Pickles, A., Happe, F., Baird, G., & Charman, T. (2012). Severe mood problems in adolescents with autism spectrum disorder. *Journal of Child Psychology and Psychiatry, 53*(11), 1157-1166. doi:https://dx.doi.org/10.1111/j.1469-7610.2012.02600.x. Exclusion reason: No psychometric information.

Simonoff, E., Lukito, S., Baird, G., Charman, T., & Pickles, A. (2015). Shared and specific behavioural and cognitive characteristics in children with ASD with and without ADHD. *European Child and Adolescent Psychiatry, 1)*, S41. doi:http://dx.doi.org/10.1007/s00787-015-0714-4. Exclusion reason: Conference abstract.

Simonoff, E., Pickles, A., Charman, T., Chandler, S., Loucas, T., & Baird, G. (2008). Psychiatric disorders in children with autism spectrum disorders: prevalence, comorbidity, and associated factors in a population-derived sample. *Journal of the American Academy of Child & Adolescent Psychiatry, 47*(8), 921-929. doi:https://dx.doi.org/10.1097/CHI.0b013e318179964f. Exclusion reason: No psychometric information.

Sinzig, J., Vinzelberg, I., Evers, D., & Lehmkuhl, G. (2014). Executive function and attention profiles in preschool and elementary school children with autism spectrum disorders or ADHD. *International Journal of Developmental Disabilities, 60(3)*, 144-154. doi:http://dx.doi.org/10.1179/2047387714Y.0000000040. Exclusion reason: No psychometric information.

Siracusano, M., Segatori, E., Riccioni, A., Emberti Gialloreti, L., Curatolo, P., & Mazzone, L. (2021). The Impact of COVID-19 on the Adaptive Functioning, Behavioral Problems, and Repetitive Behaviors of Italian Children with Autism Spectrum Disorder: An Observational Study. *Children, 8*(2), 02. doi:https://dx.doi.org/10.3390/children8020096. Exclusion reason: No psychometric information.

Siu, Q. K., Yi, H., Chan, R. C., Chio, F. H., Chan, D. F., & Mak, W. W. (2019). The role of child problem behaviors in autism spectrum symptoms and parenting stress: A primary school-based study. *Journal of Autism and Developmental Disorders, 49*(3), 857-870. doi:https://dx.doi.org/10.1007/s10803-018-3791-7. Exclusion reason: Non-ASD sample.

Sivakumar, K., Ning, S. J., Suresh, M. M., Neelothpalam, G. S., Mary, N., & Hareshkumar, N. M. (2019). Exploring the effects using T-jackets on the behaviours of adults with autism spectrum disorders (ASD). *Journal of Intellectual Disability Research, 63(7)*, 662. doi:http://dx.doi.org/10.1111/jir.12652. Exclusion reason: Conference abstract.

Skokauskas, N., & Gallagher, L. (2012). Mental health aspects of autistic spectrum disorders in children. *Journal of Intellectual Disability Research, 56*(3), 248-257. doi:https://dx.doi.org/10.1111/j.1365-2788.2011.01423.x. Exclusion reason: No psychometric information.

Skwerer, D. P., Joseph, R. M., Eggleston, B., Meyer, S. R., & Tager-Flusberg, H. (2019). Prevalence and correlates of psychiatric symptoms in minimally verbal children and adolescents with ASD. *Frontiers in Psychiatry, 10(FEB) (no pagination)*(43). doi:http://dx.doi.org/10.3389/fpsyt.2019.00043. Exclusion reason: No psychometric information.

Smith, I. M., Koegel, K., Koegel, L. K., Openden, D. A., Fossum, K. L., & Bryson, S. E. (2010). Effectiveness of a novel community-based early intervention model for children with autistic spectrum disorder. *American journal on intellectual and developmental disabilities, 115*(6), 504-523. doi:https://dx.doi.org/10.1352/1944-7558-115.6.504. Exclusion reason: Effect study.

Smith, K. R., & Matson, J. L. (2010). Psychopathology: differences among adults with intellectually disabled, comorbid autism spectrum disorders and epilepsy. *Research in Developmental Disabilities, 31*(3), 743-749. doi:https://dx.doi.org/10.1016/j.ridd.2010.01.016. Exclusion reason: No psychometric information.

Smith, K. R. M., & Matson, J. L. (2010). Psychopathology: Differences among adults with intellectually disabled, comorbid autism spectrum disorders and epilepsy. *Research in Developmental Disabilities, 31(3)*, 743-749. doi:http://dx.doi.org/10.1016/j.ridd.2010.01.016. Exclusion reason: No psychometric information.

Sneha, V. (2019). Autism spectrum disorders and its association with metabolic syndrome and atherogenesis-an exploratory case control study. *Indian Journal of Psychiatry, 61(9 Supplement 3)*, S514. Exclusion reason: Conference abstract.

Snow, A. V., & Lecavalier, L. (2011). Comparing autism, PDD-NOS, and other developmental disabilities on parent-reported behavior problems: little evidence for ASD subtype validity. *Journal of Autism & Developmental Disorders, 41*(3), 302-310. doi:https://dx.doi.org/10.1007/s10803-010-1054-3. Exclusion reason: No psychometric information.

Son, J. S., Zheng, L. J., Rowehl, L. M., Tian, X., Zhang, Y., Zhu, W., . . . Li, E. (2015). Comparison of Fecal Microbiota in Children with Autism Spectrum Disorders and Neurotypical Siblings in the Simons Simplex Collection. *PLoS ONE [Electronic Resource], 10*(10), e0137725. doi:https://dx.doi.org/10.1371/journal.pone.0137725. Exclusion reason: No psychometric information.

Song, J., Leventhal, B. L., Koh, Y. J., Cheon, K. A., Hong, H. J., Kim, Y. K., . . . Kim, Y. S. (2017). Cross-Cultural Aspect of Behavior Assessment System for Children-2, Parent Rating Scale-Child: Standardization in Korean Children. *Yonsei Medical Journal, 58*(2), 439-448. doi:https://dx.doi.org/10.3349/ymj.2017.58.2.439. Exclusion reason: No psychometric information.

Sprenger, L., Buhler, E., Poustka, L., Bach, C., Heinzel-Gutenbrunner, M., Kamp-Becker, I., & Bachmann, C. (2013). Impact of ADHD symptoms on autism spectrum disorder symptom severity. *Research in Developmental Disabilities, 34*(10), 3545-3552. doi:https://dx.doi.org/10.1016/j.ridd.2013.07.028. Exclusion reason: No psychometric information.

Sreedaran, P., & Ashok, M. V. (2015). Asperger syndrome in India: findings from a case-series with respect to clinical profile and comorbidity. *Indian Journal of Psychological Medicine, 37*(2), 212-214. doi:https://dx.doi.org/10.4103/0253-7176.155632. Exclusion reason: No psychometric information.

Stahmer, A. C., Akshoomoff, N., & Cunningham, A. B. (2011). Inclusion for toddlers with autism spectrum disorders: the first ten years of a community program. *Autism, 15*(5), 625-641. doi:https://dx.doi.org/10.1177/1362361310392253. Exclusion reason: Effect study.

Stark, K. H., Barnes, J. C., Young, N. D., & Gabriels, R. L. (2015). Brief Report: Understanding Crisis Behaviors in Hospitalized Psychiatric Patients with Autism Spectrum Disorder--Iceberg Assessment Interview. *Journal of Autism & Developmental Disorders, 45*(11), 3468-3474. doi:https://dx.doi.org/10.1007/s10803-015-2552-0. Exclusion reason: No psychometric information.

Steele, M., Uljarevic, M., Rached, G., Frazier, T. W., Phillips, J. M., Libove, R. A., . . . Hardan, A. Y. (2021). Psychiatric Characteristics Across Individuals With PTEN Mutations. *Frontiers in psychiatry Frontiers Research Foundation, 12*, 672070. doi:https://dx.doi.org/10.3389/fpsyt.2021.672070. Exclusion reason: No psychometric information.

Steinhausen, H. C., & Metzke, C. W. (2004). Differentiating the behavioural profile in autism and mental retardation and testing of a screener. *European Child & Adolescent Psychiatry, 13*(4), 214-220. Exclusion reason: No psychometric information.

Sterling, L., Dawson, G., Estes, A., & Greenson, J. (2008). Characteristics associated with presence of depressive symptoms in adults with autism spectrum disorder. *Journal of Autism & Developmental Disorders, 38*(6), 1011-1018. Exclusion reason: No psychometric information.

Sterling, L., Munson, J., Estes, A., Murias, M., Webb, S. J., King, B., & Dawson, G. (2013). Fear-potentiated startle response is unrelated to social or emotional functioning in adolescents with autism spectrum disorders. *Autism Research, 6*(5), 320-331. doi:https://dx.doi.org/10.1002/aur.1289. Exclusion reason: No psychometric information.

Stern, J. A., Gadgil, M. S., Blakeley-Smith, A., Reaven, J. A., & Hepburn, S. L. (2014). Psychometric properties of the SCARED in youth with autism spectrum disorder. *Research in Autism Spectrum Disorders, 8(9)*, 1225-1234. doi:http://dx.doi.org/10.1016/j.rasd.2014.06.008. Exclusion reason: Not relevant measurement tool.

Stewart, M., Schnabel, A., Hallford, D. J., McGillivray, J. A., Forbes, D., Foster, M., . . . Austin, D. W. (2020). Challenging child behaviours positively predict symptoms of posttraumatic stress disorder in parents of children with Autism Spectrum Disorder and Rare Diseases. *Research in Autism Spectrum Disorders, 69 (no pagination)*(101467). doi:http://dx.doi.org/10.1016/j.rasd.2019.101467. Exclusion reason: No psychometric information.

Stoppelbein, L., Biasini, F., Pennick, M., & Greening, L. (2016). Predicting internalizing and externalizing symptoms among children diagnosed with an autism spectrum disorder: The role of routines. *Journal of Child and Family Studies, 25*(1), 251-261. doi:https://dx.doi.org/10.1007/s10826-015-0218-3. Exclusion reason: No psychometric information.

Storch, E. A., Arnold, E. B., Jones, A. M., Ale, C. M., Wood, J. J., Ehrenreich-May, J., . . . Murphy, T. K. (2012). The role of co-occurring disruptive behavior in the clinical presentation of children and adolescents with anxiety in the context of autism spectrum disorders. *Child psychiatry and human development, 43*(5), 734-746. doi:https://dx.doi.org/10.1007/s10578-012-0294-1. Exclusion reason: No psychometric information.

Storch, E. A., Larson, M. J., Ehrenreich-May, J., Arnold, E. B., Jones, A. M., Renno, P., . . . Wood, J. J. (2012). Peer victimization in youth with autism spectrum disorders and co-occurring anxiety: Relations with psychopathology and loneliness. *Journal of Developmental and Physical Disabilities, 24*(6), 575-590. doi:https://dx.doi.org/10.1007/s10882-012-9290-4. Exclusion reason: No psychometric information.

Storch, E. A., Nadeau, J. M., Johnco, C., Timpano, K., McBride, N., Jane Mutch, P., . . . Murphy, T. K. (2016). Hoarding in Youth with Autism Spectrum Disorders and Anxiety: Incidence, Clinical Correlates, and Behavioral Treatment Response. *Journal of Autism and Developmental Disorders, 46(5)*, 1602-1612. doi:http://dx.doi.org/10.1007/s10803-015-2687-z. Exclusion reason: No psychometric information.

Storch, E. A., Sulkowski, M. L., Nadeau, J., Lewin, A. B., Arnold, E. B., Mutch, P., . . . Murphy, T. K. (2013). The phenomenology and clinical correlates of suicidal thoughts and behaviors in youth with autism spectrum disorders. *Journal of Autism and Developmental Disorders, 43*(10), 2450-2459. doi:https://dx.doi.org/10.1007/s10803-013-1795-x. Exclusion reason: No psychometric information.

Storch, E. A., Sulkowski, M. L., Nadeau, J., Lewin, A. B., Arnold, E. B., Mutch, P. J., . . . Murphy, T. K. (2013). The phenomenology and clinical correlates of suicidal thoughts and behaviors in youth with autism spectrum disorders. *Journal of Autism and Developmental Disorders, 43(10)*, 2450-2459. doi:http://dx.doi.org/10.1007/s10803-013-1795-x. Exclusion reason: No psychometric information.

Storch, E. A., Zavrou, S., Collier, A. B., Ung, D., Arnold, E. B., Mutch, P., . . . Murphy, T. K. (2015). Preliminary study of family accommodation in youth with autism spectrum disorders and anxiety: Incidence, clinical correlates, and behavioral treatment response. *Journal of Anxiety Disorders, 34*, 94-99. doi:https://dx.doi.org/10.1016/j.janxdis.2015.06.007. Exclusion reason: No psychometric information.

Strang, J. F., Kenworthy, L., Daniolos, P., Case, L., Wills, M. C., Martin, A., & Wallace, G. L. (2012). Depression and anxiety symptoms in children and adolescents with autism spectrum disorders without intellectual disability. *Research in Autism Spectrum Disorders, 6(1)*, 406-412. doi:http://dx.doi.org/10.1016/j.rasd.2011.06.015. Exclusion reason: No psychometric information.

Strang, J. F., Kenworthy, L., Dominska, A., Sokoloff, J., Kenealy, L. E., Berl, M., . . . Wallace, G. L. (2014). Increased gender variance in autism spectrum disorders and attention deficit hyperactivity disorder. *Archives of Sexual Behavior, 43*(8), 1525-1533. doi:https://dx.doi.org/10.1007/s10508-014-0285-3. Exclusion reason: No psychometric information.

Stratis, E. A., & Lecavalier, L. (2013). Restricted and repetitive behaviors and psychiatric symptoms in youth with autism spectrum disorders. *Research in Autism Spectrum Disorders, 7*(6), 757-766. doi:https://dx.doi.org/10.1016/j.rasd.2013.02.017. Exclusion reason: No psychometric information.

Stringer, D., Kent, R., Briskman, J., Lukito, S., Charman, T., Baird, G., . . . Simonoff, E. (2020). Trajectories of emotional and behavioral problems from childhood to early adult life. *Autism, 24*(4), 1011-1024. doi:https://dx.doi.org/10.1177/1362361320908972. Exclusion reason: No psychometric information.

Strydom, A., Bosco, A., Vickerstaff, V., Hunter, R., Hassiotis, A., Poppe, M., . . . Crawford, M. (2020). Clinical and cost effectiveness of staff training in the delivery of Positive Behaviour Support (PBS) for adults with intellectual disabilities, autism spectrum disorder and challenging behaviour-randomised trial. *BMC Psychiatry, 20(1) (no pagination)*(161). doi:http://dx.doi.org/10.1186/s12888-020-02577-1. Exclusion reason: Effect study.

Suzuki, M., Yamada, A., Watanabe, N., Akechi, T., Katsuki, F., Nishiyama, T., . . . Furukawa, T. A. (2014). A failure to confirm the effectiveness of a brief group psychoeducational program for mothers of children with high-functioning pervasive developmental disorders: a randomized controlled pilot trial. *Neuropsychiatric Disease & Treatment, 10*, 1141-1153. doi:https://dx.doi.org/10.2147/NDT.S60058. Exclusion reason: Effect study.

Suzumura, S. (2015). Quality of life in mothers of preschoolers with high-functioning pervasive developmental disorders. *Pediatrics International, 57*(1), 149-154. doi:https://dx.doi.org/10.1111/ped.12560. Exclusion reason: No psychometric information.

Szatmari, P., Cost, K. T., Duku, E., Bennett, T., Elsabbagh, M., Georgiades, S., . . . Zwaigenbaum, L. (2021). Association of Child and Family Attributes With Outcomes in Children With Autism. *JAMA Network Open, 4*(3), e212530. doi:https://dx.doi.org/10.1001/jamanetworkopen.2021.2530. Exclusion reason: No psychometric information.

Taffe, J. R., Gray, K. M., Einfeld, S. L., Dekker, M. C., Koot, H. M., Emerson, E., . . . Tonge, B. J. (2007). Short form of the developmental behaviour checklist. *American Journal of Mental Retardation, 112*(1), 31-39. Exclusion reason: No psychometric information.

Tajik-Parvinchi, D., Rosenbaum, P., Duku, E., Cooley Hidecker, M., Zwaigenbaum, L., Roncadin, C., . . . Di Rezze, B. (2021). Construct validity of the autism classification system of functioning: Social communication across childhood and adolescence. *Developmental Medicine and Child Neurology, 63(SUPPL 3)*, 14-15. doi:http://dx.doi.org/10.1111/dmcn.15004. Exclusion reason: Conference abstract.

Tajik-Parvinchi, D. J., Farmus, L., Cribbie, R., Albaum, C., & Weiss, J. A. (2020). Clinical and parental predictors of emotion regulation following cognitive behaviour therapy in children with autism. *Autism, 24(4)*, 851-866. doi:http://dx.doi.org/10.1177/1362361320909178. Exclusion reason: No psychometric information.

Takahashi, H., Komatsu, S., Nakahachi, T., Ogino, K., & Kamio, Y. (2016). Relationship of the Acoustic Startle Response and Its Modulation to Emotional and Behavioral Problems in Typical Development Children and Those with Autism Spectrum Disorders. *Journal of Autism & Developmental Disorders, 46*(2), 534-543. doi:https://dx.doi.org/10.1007/s10803-015-2593-4. Exclusion reason: Sample size = < 20.

Tanner, K., Case-Smith, J., Nahikian-Nelms, M., Ratliff-Schaub, K., Spees, C., & Darragh, A. R. (2015). Behavioral and physiological factors associated with selective eating in children with autism spectrum disorder. *American Journal of Occupational Therapy, 69*(6), p1-p8. doi:https://dx.doi.org/10.5014/ajot.2015.019273. Exclusion reason: No psychometric information.

Tateno, Y., Kumagai, K., Monden, R., Nanba, K., Yano, A., Shiraishi, E., . . . Tateno, M. (2021). The Efficacy of Early Start Denver Model Intervention in Young Children with Autism Spectrum Disorder Within Japan: A Preliminary Study. *Soa!$ceongsonyeon Jeongsin Yihag, 32*(1), 35-40. doi:https://dx.doi.org/10.5765/jkacap.200040. Exclusion reason: Effect study.

Taylor, J. L., & Gotham, K. O. (2016). Cumulative life events, traumatic experiences, and psychiatric symptomatology in transition-aged youth with autism spectrum disorder. *Journal of Neurodevelopmental Disorders Vol 8 2016, ArtID 28, 8*. Exclusion reason: No psychometric information.

Taylor, J. L., & Warren, Z. E. (2012). Maternal depressive symptoms following autism spectrum diagnosis. *Journal of Autism & Developmental Disorders, 42*(7), 1411-1418. doi:https://dx.doi.org/10.1007/s10803-011-1375-x. Exclusion reason: No psychometric information.

Taylor, L. J., Luk, S. Y. L., Leadbitter, K., Moore, H. L., & Charman, T. (2021). Are child autism symptoms, developmental level and adaptive function associated with caregiver feelings of wellbeing and efficacy in the parenting role? *Research in Autism Spectrum Disorders, 83 (no pagination)*(101738). doi:http://dx.doi.org/10.1016/j.rasd.2021.101738. Exclusion reason: No psychometric information.

Teague, S. J., Newman, L. K., Tonge, B. J., & Gray, K. M. (2020). Attachment and child behaviour and emotional problems in autism spectrum disorder with intellectual disability. *Journal of Applied Research in Intellectual Disabilities, 33*(3), 475-487. doi:https://dx.doi.org/10.1111/jar.12689. Exclusion reason: No psychometric information.

Tee, A., & Reed, P. (2017). Controlled study of the impact on child behaviour problems of intensive interaction for children with ASD. *Journal of Research in Special Educational Needs, 17*(3), 179-186. doi:https://dx.doi.org/10.1111/1471-3802.12376. Exclusion reason: Effect study.

Thomas, S., Lycett, K., Papadopoulos, N., Sciberras, E., & Rinehart, N. (2018). Exploring behavioral sleep problems in children with ADHD and comorbid autism spectrum disorder. *Journal of Attention Disorders, 22*(10), 947-958. doi:https://dx.doi.org/10.1177/1087054715613439. Exclusion reason: No psychometric information.

Thomas, S., Sciberras, E., Lycett, K., Papadopoulos, N., & Rinehart, N. (2018). Physical Functioning, Emotional, and Behavioral Problems in Children With ADHD and Comorbid ASD: A Cross-Sectional Study. *Journal of Attention Disorders, 22*(10), 1002-1007. doi:https://dx.doi.org/10.1177/1087054715587096. Exclusion reason: No psychometric information.

Thurman, A. J., McDuffie, A., Hagerman, R., & Abbeduto, L. (2014). Psychiatric symptoms in boys with fragile X syndrome: A comparison with nonsyndromic autism spectrum disorder. *Research in Developmental Disabilities, 35*(5), 1072-1086. doi:https://dx.doi.org/10.1016/j.ridd.2014.01.032. Exclusion reason: No psychometric information.

Tick, B., Colvert, E., McEwen, F., Stewart, C., Woodhouse, E., Gillan, N., . . . Rijsdijk, F. (2016). Autism Spectrum Disorders and Other Mental Health Problems: Exploring Etiological Overlaps and Phenotypic Causal Associations. *Journal of the American Academy of Child & Adolescent Psychiatry, 55*(2), 106-113.e104. doi:https://dx.doi.org/10.1016/j.jaac.2015.11.013. Exclusion reason: No psychometric information.

Tipton-Fisler, L. A., Rodriguez, G., Zeedyk, S. M., & Blacher, J. (2018). Stability of bullying and internalizing problems among adolescents with ASD, ID, or typical development. *Research in Developmental Disabilities, 80*, 131-141. doi:http://dx.doi.org/10.1016/j.ridd.2018.06.004. Exclusion reason: No psychometric information.

Tomanik, S., Harris, G. E., & Hawkins, J. (2004). The relationship between behaviours exhibited by children with autism and maternal stress. *Journal of Intellectual and Developmental Disability, 29(1)*, 16-26. doi:http://dx.doi.org/10.1080/13668250410001662892. Exclusion reason: No psychometric information.

Tomeny, T. S., Barry, T. D., & Bader, S. H. (2014). Birth order rank as a moderator of the relation between behavior problems among children with an autism spectrum disorder and their siblings. *Autism, 18*(2), 199-202. doi:https://dx.doi.org/10.1177/1362361312458185. Exclusion reason: No psychometric information.

Tonge, B. J., Brereton, A. V., Gray, K. M., & Einfeld, S. L. (1999). Behavioural and emotional disturbance in high-functioning autism and Asperger syndrome. *Autism, 3(2)*, 117-130. doi:http://dx.doi.org/10.1177/1362361399003002003. Exclusion reason: No psychometric information.

Toseeb, U., McChesney, G., & Wolke, D. (2018). The prevalence and psychopathological correlates of sibling bullying in children with and without autism spectrum disorder. *Journal of Autism and Developmental Disorders, 48*(7), 2308-2318. doi:https://dx.doi.org/10.1007/s10803-018-3484-2. Exclusion reason: No psychometric information.

Totsika, V., Felce, D., Kerr, M., & Hastings, R. P. (2010). Behavior problems, psychiatric symptoms, and quality of life for older adults with intellectual disability with and without autism. *Journal of Autism and Developmental Disorders, 40*(10), 1171-1178. doi:https://dx.doi.org/10.1007/s10803-010-0975-1. Exclusion reason: Non-ASD sample.

Totsika, V., Hastings, R. P., Emerson, E., Berridge, D. M., & Lancaster, G. A. (2011). Behavior problems at 5 years of age and maternal mental health in autism and intellectual disability. *Journal of Abnormal Child Psychology, 39*(8), 1137-1147. doi:https://dx.doi.org/10.1007/s10802-011-9534-2. Exclusion reason: No psychometric information.

Totsika, V., Hastings, R. P., Emerson, E., Lancaster, G. A., & Berridge, D. M. (2011). A population-based investigation of behavioural and emotional problems and maternal mental health: Associations with autism spectrum disorder and intellectual disability. *Journal of Child Psychology and Psychiatry, 52*(1), 91-99. doi:https://dx.doi.org/10.1111/j.1469-7610.2010.02295.x. Exclusion reason: No psychometric information.

Trelles, P., Wilkinson, E., & Kolevzon, A. (2018). 5.11 Characterizing Attention Deficit in Children With ASD. *Journal of the American Academy of Child and Adolescent Psychiatry, 57(10 Supplement)*, S230. doi:http://dx.doi.org/10.1016/j.jaac.2018.09.306. Exclusion reason: Conference abstract.

Tsakanikos, E., Underwood, L., Kravariti, E., Bouras, N., & McCarthy, J. (2011). Gender differences in co-morbid psychopathology and clinical management in adults with autism spectrum disorders. *Research in Autism Spectrum Disorders, 5(2)*, 803-808. doi:http://dx.doi.org/10.1016/j.rasd.2010.09.009. Exclusion reason: No psychometric information.

Tseng, M. H., Fu, C. P., Cermak, S. A., Lu, L., & Shieh, J. Y. (2011). Emotional and behavioral problems in preschool children with autism: Relationship with sensory processing dysfunction. *Research in Autism Spectrum Disorders, 5(4)*, 1441-1450. doi:http://dx.doi.org/10.1016/j.rasd.2011.02.004. Exclusion reason: No psychometric information.

Tseng, M.-H., Fu, C.-P., Cermak, S. A., Lu, L., & Shieh, J.-Y. (2011). Emotional and behavioral problems in preschool children with autism: Relationship with sensory processing dysfunction. *Research in Autism Spectrum Disorders, 5*(4), 1441-1450. doi:https://dx.doi.org/10.1016/j.rasd.2011.02.004. Exclusion reason: No psychometric information.

Tsuji, H., Miyawaki, D., Kawaguchi, T., Matsushima, N., Horino, A., Takahashi, K., . . . Kiriike, N. (2009). Relationship of hypersensitivity to anxiety and depression in children with high-functioning pervasive developmental disorders. *Psychiatry & Clinical Neurosciences, 63*(2), 195-201. doi:https://dx.doi.org/10.1111/j.1440-1819.2008.01916.x. Exclusion reason: No psychometric information.

Tudor, M. E., DeVincent, C. J., & Gadow, K. D. (2012). Prenatal pregnancy complications and psychiatric symptoms: Children with ASD versus clinic controls. *Research in Autism Spectrum Disorders, 6*(4), 1401-1405. doi:https://dx.doi.org/10.1016/j.rasd.2012.06.001. Exclusion reason: No psychometric information.

Tung, L. C., Huang, C. Y., Tseng, M. H., Yen, H. C., Tsai, Y. P., Lin, Y. C., & Chen, K. L. (2014). Correlates of health-related quality of life and the perception of its importance in caregivers of children with autism. *Research in Autism Spectrum Disorders, 8(9)*, 1235-1242. doi:http://dx.doi.org/10.1016/j.rasd.2014.06.010. Exclusion reason: No psychometric information.

Tureck, K., Matson, J. L., Cervantes, P., & Konst, M. J. (2014). An examination of the relationship between autism spectrum disorder, intellectual functioning, and comorbid symptoms in children. *Research in Developmental Disabilities, 35*(7), 1766-1772. doi:https://dx.doi.org/10.1016/j.ridd.2014.02.013. Exclusion reason: No psychometric information.

Tureck, K., Matson, J. L., May, A., Davis, T. E., III, & Whiting, S. E. (2013). Investigation of the rates of comorbid symptoms in children with ADHD compared to children with ASD. *Journal of Developmental and Physical Disabilities, 25*(4), 405-417. doi:https://dx.doi.org/10.1007/s10882-012-9320-2. Exclusion reason: No psychometric information.

Tureck, K., Matson, J. L., May, A., & Turygin, N. (2013). Externalizing and tantrum behaviours in children with ASD and ADHD compared to children with ADHD. *Developmental neurorehabilitation, 16*(1), 52-57. doi:https://dx.doi.org/10.3109/17518423.2012.719245. Exclusion reason: No psychometric information.

Ugur, C., Sertcelik, M., Uneri, O., Senses Dinc, G., Sekmen, E., & Solmaz, E. (2018). Evaluation of serum urotensin-II levels of children with ADHD and autism spectrum disorder. *Anadolu Psikiyatri Dergisi, 19(1)*, 80-86. doi:http://dx.doi.org/10.5455/apd.263095. Exclusion reason: No psychometric information

Ugur, C., Tonyali, A., Goker, Z., & Uneri, O. S. (2019). Birth order and reproductive stoppage in families of children with autism spectrum disorder. *Psychiatry and Clinical Psychopharmacology, 29(4)*, 509-514. doi:http://dx.doi.org/10.1080/24750573.2018.1457489. Exclusion reason: No psychometric information.

Ugur, C., Tunca, H., Sekmen, E., Uneri, O. S., Alisik, M., Erel, O., & Solmaz, E. (2018). A comparative study of the oxidative stress indices of children with autism and healthy children. *Anadolu Psikiyatri Dergisi, 19(3)*, 314-322. doi:http://dx.doi.org/10.5455/apd.276094. Exclusion reason: No psychometric information.

Uljarevic, M., Phillips, J. M., Schuck, R. K., Schapp, S., Solomon, E. M., Salzman, E., . . . Hardan, A. Y. (2020). Exploring Social Subtypes in Autism Spectrum Disorder: A Preliminary Study. *Autism research : Official Journal of the International Society for Autism Research, 13*(8), 1335-1342. doi:https://dx.doi.org/10.1002/aur.2294. Exclusion reason: No psychometric information.

Valentovich, V., Goldberg, W. A., Garfin, D. R., & Guo, Y. (2018). Emotion coregulation processes between mothers and their children with and without autism spectrum disorder: Associations with children's maladaptive behaviors. *Journal of Autism and Developmental Disorders, 48*(4), 1235-1248. doi:https://dx.doi.org/10.1007/s10803-017-3375-y. Exclusion reason: No psychometric information.

Valeri, G., Casula, L., Menghini, D., Amendola, F. A., Napoli, E., Pasqualetti, P., & Vicari, S. (2020). Cooperative parent-mediated therapy for Italian preschool children with autism spectrum disorder: a randomized controlled trial. *European Child and Adolescent Psychiatry, 29(7)*, 935-946. doi:http://dx.doi.org/10.1007/s00787-019-01395-5. Exclusion reason: Sample size = < 20.

Valicenti-McDermott, M., Lawson, K., Hottinger, K., Seijo, R., Schechtman, M., Shulman, L., & Shinnar, S. (2015). Parental Stress in Families of Children With Autism and Other Developmental Disabilities. *Journal of Child Neurology, 30*(13), 1728-1735. doi:https://dx.doi.org/10.1177/0883073815579705. Exclusion reason: No psychometric information.

Valicenti-McDermott, M., Lawson, K., Hottinger, K., Seijo, R., Schechtman, M., Shulman, L., & Shinnar, S. (2019). Sleep Problems in Children With Autism and Other Developmental Disabilities: A Brief Report. *Journal of Child Neurology, 34*(7), 387-393. doi:https://dx.doi.org/10.1177/0883073819836541. Exclusion reason: No psychometric information.

Van Dijck, A., Vulto-van Silfhout, A. T., Cappuyns, E., van der Werf, I. M., Mancini, G. M., Tzschach, A., . . . Kooy, R. F. (2019). Clinical Presentation of a Complex Neurodevelopmental Disorder Caused by Mutations in ADNP. *Biological Psychiatry, 85*(4), 287-297. doi:https://dx.doi.org/10.1016/j.biopsych.2018.02.1173. Exclusion reason: Non-ASD sample.

van Steensel, F. J., Bogels, S. M., & de Bruin, E. I. (2013). Psychiatric Comorbidity in Children with Autism Spectrum Disorders: A Comparison with Children with ADHD. *Journal of Child & Family Studies, 22*(3), 368-376. Exclusion reason: No psychometric information.

Vasa, R. A., Kalb, L., Mazurek, M., Kanne, S., Freedman, B., Keefer, A., . . . Murray, D. (2013). Age-related differences in the prevalence and correlates of anxiety in youth with autism spectrum disorders. *Research in Autism Spectrum Disorders, 7(11)*, 1358-1369. doi:http://dx.doi.org/10.1016/j.rasd.2013.07.005. Exclusion reason: No psychometric information.

Veatch, O. J., Sutcliffe, J. S., Warren, Z. E., Keenan, B. T., Potter, M. H., & Malow, B. A. (2017). Shorter sleep duration is associated with social impairment and comorbidities in ASD. *Autism research : Official Journal of the International Society for Autism Research, 10*(7), 1221-1238. doi:https://dx.doi.org/10.1002/aur.1765. Exclusion reason: No psychometric information.

Verheij, C., Louwerse, A., van der Ende, J., Eussen, M. L., Van Gool, A. R., Verheij, F., . . . Greaves-Lord, K. (2015). The Stability of Comorbid Psychiatric Disorders: A 7 Year Follow Up of Children with Pervasive Developmental Disorder-Not Otherwise Specified. *Journal of Autism & Developmental Disorders, 45*(12), 3939-3948. doi:https://dx.doi.org/10.1007/s10803-015-2592-5. Exclusion reason: No psychometric information.

Verheij, C., Louwerse, A., van der Ende, J., Eussen, M. L. J. M., Van Gool, A. R., Verheij, F., . . . Greaves-Lord, K. (2015). The Stability of Comorbid Psychiatric Disorders: A 7 Year Follow Up of Children with Pervasive Developmental Disorder-Not Otherwise Specified. *Journal of Autism and Developmental Disorders, 45(12)*, 3939-3948. doi:http://dx.doi.org/10.1007/s10803-015-2592-5. Exclusion reason: No psychometric information.

Viscidi, E. W., Johnson, A. L., Spence, S. J., Buka, S. L., Morrow, E. M., & Triche, E. W. (2014). The association between epilepsy and autism symptoms and maladaptive behaviors in children with autism spectrum disorder. *Autism, 18*(8), 996-1006. doi:https://dx.doi.org/10.1177/1362361313508027. Exclusion reason: No psychometric information.

Visser, J. C., Rommelse, N. N. J., Lappenschaar, M., Servatius-Oosterling, I. J., Greven, C. U., & Buitelaar, J. K. (2017). Variation in the Early Trajectories of Autism Symptoms Is Related to the Development of Language, Cognition, and Behavior Problems. *Journal of the American Academy of Child & Adolescent Psychiatry, 56*(8), 659-668. doi:https://dx.doi.org/10.1016/j.jaac.2017.05.022. Exclusion reason: No psychometric information.

Visser, J. C., Smeekens, S., Rommelse, N., Verkes, R. J., van der Gaag, R. J., & Buitelaar, J. K. (2010). Assessment of psychopathology in 2- to 5-year-olds: Applying the Infant-Toddler Social Emotional Assessment. *Infant Mental Health Journal, 31*(6), 611-629. doi:https://dx.doi.org/10.1002/imhj.20273. Exclusion reason: Non-ASD sample.

Vogan, V. M., Leung, R. C., Safar, K., Martinussen, R., Smith, M. L., & Taylor, M. J. (2018). Longitudinal examination of everyday executive functioning in children with ASD: Relations with social, emotional, and behavioral functioning over time. *Frontiers in Psychology Vol 9 2018, ArtID 1774, 9*. doi:https://dx.doi.org/10.3389/fpsyg.2018.01774. Exclusion reason: No psychometric information.

Vohra, R., Madhavan, S., & Khanna, R. (2012). Severity of autism spectrum disorders: Effect on caregiver burden and satisfaction with school district services. *Value in Health, 15(4)*, A90. doi:http://dx.doi.org/10.1016/j.jval.2012.03.494. Exclusion reason: Conference abstract.

Voinea, C., Nicolau, I., Tudosie, V., & Mateescu, L. (2021). P.0650 Adolescence to adulthood 1-10 years follow-up of Asperger's Syndrome and factors related to the long-term outcome. *European Neuropsychopharmacology, 53(Supplement 1)*, S478-S479. doi:https://dx.doi.org/10.1016/j.euroneuro.2021.10.614. Exclusion reason: Conference abstract.

Volker, M. A., Lopata, C., Smerbeck, A. M., Knoll, V. A., Thomeer, M. L., Toomey, J. A., & Rodgers, J. D. (2010). BASC-2 prs profiles for students with high-functioning autism spectrum disorders. *Journal of Autism and Developmental Disorders, 40*(2), 188-199. doi:https://dx.doi.org/10.1007/s10803-009-0849-6. Exclusion reason: No psychometric information.

von Gontard, A., Pirrung, M., Niemczyk, J., & Equit, M. (2015). Incontinence in children with autism spectrum disorder. *Journal of pediatric urology, 11*(5), 264.e261-267. doi:https://dx.doi.org/10.1016/j.jpurol.2015.04.015. Exclusion reason: No psychometric information.

Wade, J. L., Cox, N. B., Reeve, R. E., & Hull, M. (2014). Brief report: Impact of child problem behaviors and parental broad autism phenotype traits on substance use among parents of children with ASD. *Journal of Autism and Developmental Disorders, 44(10)*, 2621-2627. doi:http://dx.doi.org/10.1007/s10803-014-2132-8. Exclusion reason: No psychometric information.

Walsh, C. E., Mulder, E., & Tudor, M. E. (2013). Predictors of parent stress in a sample of children with ASD: Pain, problem behavior, and parental coping. *Research in Autism Spectrum Disorders, 7*(2), 256-264. doi:https://dx.doi.org/10.1016/j.rasd.2012.08.010. Exclusion reason: No psychometric information.

Wang, G., Liu, Z., Lu, N., Lewin, D., Xu, G., & Owens, J. (2014). Sleep disturbances in Chinese children with autism spectrum disorders: Characteristics and associated factors. *Sleep, 1)*, A291. Exclusion reason: No psychometric information.

Wang, G.-f., Li, W.-L., Han, Y., Gao, L., Dai, W., Su, Y.-y., & Zhang, X. (2019). Sensory processing problems and comorbidities in Chinese preschool children with autism spectrum disorders. *Journal of Autism and Developmental Disorders, 49*(10), 4097-4108. doi:https://dx.doi.org/10.1007/s10803-019-04125-7. Exclusion reason: No psychometric information.

Wang, Y., Lin, J., Zeng, Y., Liu, Y., Li, Y., Xia, K., . . . Ou, J. (2020). Effects of Sleep Disturbances on Behavioral Problems in Preschool Children With Autism Spectrum Disorder. *Frontiers in psychiatry Frontiers Research Foundation, 11*, 559694. doi:https://dx.doi.org/10.3389/fpsyt.2020.559694. Exclusion reason: No psychometric information.

Waters, P., & Healy, O. (2012). Investigating the Relationship between Self-Injurious Behavior, Social Deficits, and Cooccurring Behaviors in Children and Adolescents with Autism Spectrum Disorder. *Autism Research & Treatment Print, 2012*, 156481. doi:https://dx.doi.org/10.1155/2012/156481. Exclusion reason: No psychometric information.

Weber, R. J., & Gadow, K. D. (2017). Relation of psychiatric symptoms with epilepsy, asthma, and allergy in youth with ASD vs. psychiatry referrals. *Journal of Abnormal Child Psychology, 45*(6), 1247-1257. doi:https://dx.doi.org/10.1007/s10802-016-0212-2. Exclusion reason: No psychometric information.

Weiss, J. A., Cappadocia, M. C., & Lunsky, Y. (2012). Psychological acceptance and empowerment as mediators of child behaviour problems on parent mental health. *Journal of Intellectual Disability Research, 56(7-8)*, 678. doi:http://dx.doi.org/10.1111/j.1365-2788.2012.01583_4.x. Exclusion reason: Conference abstract.

Weiss, J. A., MacMullin, J. A., & Lunsky, Y. (2015). Empowerment and parent gain as mediators and moderators of distress in mothers of children with autism spectrum disorders. *Journal of Child and Family Studies, 24*(7), 2038-2045. doi:https://dx.doi.org/10.1007/s10826-014-0004-7. Exclusion reason: No psychometric information.

Weissman, A. S., & Bates, M. E. (2010). Increased clinical and neurocognitive impairment in children with autism spectrum disorders and comorbid bipolar disorder. *Research in Autism Spectrum Disorders, 4(4)*, 670-680. doi:http://dx.doi.org/10.1016/j.rasd.2010.01.005. Exclusion reason: Not relevant measurement tool.

White, S. W., & Roberson-Nay, R. (2009). Anxiety, social deficits, and loneliness in youth with autism spectrum disorders. *Journal of Autism and Developmental Disorders, 39*(7), 1006-1013. doi:https://dx.doi.org/10.1007/s10803-009-0713-8. Exclusion reason: No psychometric information.

Wiggins, L. D., DiGuiseppi, C., Schieve, L., Moody, E., Soke, G., Giarelli, E., & Levy, S. (2020). Wandering Among Preschool Children with and Without Autism Spectrum Disorder. *Journal of Developmental & Behavioral Pediatrics, 41*(4), 251-257. doi:https://dx.doi.org/10.1097/DBP.0000000000000780. Exclusion reason: No psychometric information.

Williams, D. L., Siegel, M., & Mazefsky, C. A. (2018). Problem behaviors in autism spectrum disorder: Association with verbal ability and adapting/coping skills. *Journal of Autism and Developmental Disorders, 48*(11), 3668-3677. doi:https://dx.doi.org/10.1007/s10803-017-3179-0. Exclusion reason: No psychometric information.

Williams, K. C., Christofi, F. L., Clemmons, T., Rosenberg, D., & Fuchs, G. J. (2012). Chronic gi symptoms in children with autism spectrum disorders are associated with clinical anxiety. *Gastroenterology, 1)*, S79-S80. Exclusion reason: Conference abstract.

Williams, K. C., Fuchs, G. J., Furuta, G. T., Marcon, M. A., & Coury, D. L. (2010). Clinical features associated with GI symptoms in Autism Spectrum Disorders (ASD). *Gastroenterology, 1)*, S74. Exclusion reason: Conference abstract.

Williams, L. W., Matson, J. L., Beighley, J. S., Rieske, R. D., & Adams, H. L. (2014). Comorbid symptoms in toddlers diagnosed with autism spectrum disorder with the DSM-IV-TR and the DSM-5 criteria. *Research in Autism Spectrum Disorders, 8*(3), 186-192. doi:https://dx.doi.org/10.1016/j.rasd.2013.11.007. Exclusion reason: No psychometric information

Williams, L. W., Matson, J. L., Jang, J., Beighley, J. S., Rieske, R. D., & Adams, H. L. (2013). Challenging behaviors in toddlers diagnosed with autism spectrum disorders with the DSM-IV-TR and the proposed DSM-5 criteria. *Research in Autism Spectrum Disorders, 7(8)*, 966-972. doi:http://dx.doi.org/10.1016/j.rasd.2013.03.010. Exclusion reason: No psychometric information.

Williams, S., Leader, G., Mannion, A., & Chen, J. (2015). An investigation of anxiety in children and adolescents with autism spectrum disorder. *Research in Autism Spectrum Disorders, 10*, 30-40. doi:http://dx.doi.org/10.1016/j.rasd.2014.10.017. Exclusion reason: No psychometric information.

Wilson, C. E., Murphy, C. M., McAlonan, G., Robertson, D. M., Spain, D., Hayward, H., . . . Murphy, D. G. (2016). Does sex influence the diagnostic evaluation of autism spectrum disorder in adults? *Autism, 20*(7), 808-819. doi:https://dx.doi.org/10.1177/1362361315611381. Exclusion reason: Not relevant measurement tool.

Wilson, C. E., Murphy, C. M., McAlonan, G., Robertson, D. M., Spain, D., Hayward, H., . . . Murphy, D. G. M. (2016). Does sex influence the diagnostic evaluation of autism spectrum disorder in adults? *Autism, 20(7)*, 808-819. doi:http://dx.doi.org/10.1177/1362361315611381. Exclusion reason: Not relevant measurement tool.

Witwer, A. N., & Lecavalier, L. (2010). Validity of comorbid psychiatric disorders in youngsters with autism spectrum disorders. *Journal of Developmental and Physical Disabilities, 22*(4), 367-380. doi:https://dx.doi.org/10.1007/s10882-010-9194-0. Exclusion reason: No psychometric information.

Worley, J. A., & Matson, J. L. (2011). Psychiatric symptoms in children diagnosed with an Autism Spectrum Disorder: An examination of gender differences. *Research in Autism Spectrum Disorders, 5(3)*, 1086-1091. doi:http://dx.doi.org/10.1016/j.rasd.2010.12.002. Exclusion reason: No psychometric information.

Wozniak, J., Biederman, J., Faraone, S. V., Frazier, J., Kim, J., Millstein, R., . . . Snyder, J. B. (1997). Mania in children with pervasive developmental disorder revisited. *Journal of the American Academy of Child & Adolescent Psychiatry, 36*(11), 1552-1559. discussion 1559. Exclusion reason: No psychometric information.

Yamada, T., Miura, Y., Oi, M., Akatsuka, N., Tanaka, K., Tsukidate, N., . . . Laugeson, E. A. (2020). Examining the treatment efficacy of PEERS in Japan: Improving social skills among adolescents with autism spectrum disorder. *Journal of Autism and Developmental Disorders, 50*(3), 976-997. doi:https://dx.doi.org/10.1007/s10803-019-04325-1. Exclusion reason: Sample size = < 20.

Yamawaki, K., Ishitsuka, K., Suyama, S., Suzumura, S., Yamashita, H., & Kanba, S. (2020). Clinical characteristics of boys with comorbid autism spectrum disorder and attention deficit/hyperactivity disorder. *Pediatrics International, 62*(2), 151-157. doi:https://dx.doi.org/10.1111/ped.14105. Exclusion reason: No psychometric information.

Yang, Y. J. D., Sukhodolsky, D. G., Lei, J., Dayan, E., Pelphrey, K. A., & Ventola, P. (2017). Distinct neural bases of disruptive behavior and autism symptom severity in boys with autism spectrum disorder. *Journal of Neurodevelopmental Disorders, 9*, 17. doi:10.1186/s11689-017-9183-z. Exclusion reason: No psychometric information.

Yavuz-Kodat, E., Reynaud, E., & Schroder, C. (2020). 38.1 Disturbances of Continuous Sleep and Circadian Rhythms Account for Behavioral Difficulties in Children with Autism Spectrum Disorder. *Journal of the American Academy of Child and Adolescent Psychiatry, 59(10 Supplement)*, S218. doi:http://dx.doi.org/10.1016/j.jaac.2020.08.302. Exclusion reason: Conference abstract.

Yerys, B. E., Wallace, G. L., Sokoloff, J. L., Shook, D. A., James, J. D., & Kenworthy, L. (2009). Attention deficit/hyperactivity disorder symptoms moderate cognition and behavior in children with autism spectrum disorders. *Autism research : Official Journal of the International Society for Autism Research, 2*(6), 322-333. doi:https://dx.doi.org/10.1002/aur.103. Exclusion reason: No psychometric information.

Yildiz, B. B., Mutlu, C., Ocakoglu, F. T., Dogan, E. B., Yalcin, O., Dut, R., & Karacetin, G. (2022). Relationship of Temperament Differences with Diagnosis, Severity and Accompanying Psychiatric Symptoms in Autism Spectrum Disorder. *Noropsikiyatri Arsivi, 59(1)*, 26-32. doi:https://dx.doi.org/10.29399/npa.27830. Exclusion reason: No psychometric information.

Yu, X. T., Lam, H. S., Au, C. T., Chan, S. H. Y., Chan, D. F. Y., & Li, A. M. (2015). Extended parent-based behavioural education improves sleep in children with autism spectrum disorder. *Hong Kong Journal of Paediatrics, 20(4)*, 219-225. Exclusion reason: Effect study.

Yu, Y. W., Chung, K. H., Lee, Y. K., Lam, W. C., & Yiu, M. G. (2016). Prevalence of Maternal Affective Disorders in Chinese Mothers of Preschool Children with Autism Spectrum Disorders. *East Asian Archives of Psychiatry, 26*(4), 121-128. Exclusion reason: No psychometric information.

Yu, Y. W., Chung, K. H., Lee, Y. K., Lam, W. C., & Yiu, M. G. C. (2016). Prevalence of maternal affective disorders in Chinese mothers of preschool children with autism spectrum disorders. *East Asian Archives of Psychiatry, 26(4)*, 121-128. Exclusion reason: No psychometric information.

Yui, K., Imataka, G., Kawasak, Y., & Yamada, H. (2016). Increased omega-3 polyunsaturated fatty acid/arachidonic acid ratios and upregulation of signaling mediator in individuals with autism spectrum disorders. *Life Sciences, 145*, 205-212. doi:https://dx.doi.org/10.1016/j.lfs.2015.12.039. Exclusion reason: Not relevant measurement tool.

Zachor, D., Yang, J. W., Itzchak, E. B., Furniss, F., Pegg, E., Matson, J. L., . . . Jung, W. (2011). Cross-cultural differences in comorbid symptoms of children with autism spectrum disorders: an international examination between Israel, South Korea, the United Kingdom and the United States of America. *Developmental neurorehabilitation, 14*(4), 215-220. doi:https://dx.doi.org/10.3109/17518423.2011.568468. Exclusion reason: Not relevant measurement tool.

Zaidman-Zait, A., Mirenda, P., Duku, E., Szatmari, P., Georgiades, S., Volden, J., . . . Thompson, A. (2014). Examination of bidirectional relationships between parent stress and two types of problem behavior in children with autism spectrum disorder. *Journal of Autism and Developmental Disorders, 44(8)*, 1908-1917. doi:http://dx.doi.org/10.1007/s10803-014-2064-3. Exclusion reason: No psychometric information.

Zaidman-Zait, A., Mirenda, P., Duku, E., Szatmari, P., Georgiades, S., Volden, J., . . . Pathways in, A. S. D. S. T. (2014). Examination of bidirectional relationships between parent stress and two types of problem behavior in children with autism spectrum disorder. *Journal of Autism & Developmental Disorders, 44*(8), 1908-1917. doi:https://dx.doi.org/10.1007/s10803-014-2064-3. Exclusion reason: No psychometric information.

Zaidman-Zait, A., Mirenda, P., Duku, E., Vaillancourt, T., Smith, I. M., Szatmari, P., . . . Thompson, A. (2017). Impact of personal and social resources on parenting stress in mothers of children with autism spectrum disorder. *Autism, 21*(2), 155-166. doi:https://dx.doi.org/10.1177/1362361316633033. Exclusion reason: No psychometric information.

Zaidman-Zait, A., Mirenda, P., Zumbo, B. D., Georgiades, S., Szatmari, P., Bryson, S., . . . Pathways in, A. S. D. S. T. (2011). Factor analysis of the Parenting Stress Index-Short Form with parents of young children with autism spectrum disorders. *Autism research : Official Journal of the International Society for Autism Research, 4*(5), 336-346. doi:https://dx.doi.org/10.1002/aur.213. Exclusion reason: No psychometric information.

Zaidman-Zait, A., Zwaigenbaum, L., Duku, E., Bennett, T., Szatmari, P., Mirenda, P., . . . Roberts, W. (2020). Factor analysis of the children's sleep habits questionnaire among preschool children with autism spectrum disorder. *Research in Developmental Disabilities, 97*, 103548. doi:https://dx.doi.org/10.1016/j.ridd.2019.103548. Exclusion reason: No psychometric information.

Zainal, H., & Magiati, I. (2019). A comparison between caregiver-reported anxiety and other emotional and behavioral difficulties in children and adolescents with Autism Spectrum Disorders attending specialist or mainstream schools. *Journal of Autism and Developmental Disorders, 49*(7), 2653-2663. doi:https://dx.doi.org/10.1007/s10803-016-2792-7. Exclusion reason: Not relevant measurement tool.

Zainal, H., Magiati, I., Tan, J. W. L., Sung, M., Fung, D. S. S., & Howlin, P. (2014). Erratum to: A preliminary investigation of the Spence children's anxiety parent scale as a screening tool for anxiety in young people with autism spectrum disorders (Journal of Autism and Developmental Disorders DOI: 10.1007/s10803-014-2075-0). *Journal of Autism and Developmental Disorders, 44(8)*, 1995. doi:http://dx.doi.org/10.1007/s10803-014-2090-1. Exclusion reason: No psychometric information.

Zainal, H., Magiati, I., Tan, J. W. L., Sung, M., Fung, D. S. S., & Howlin, P. (2014). A preliminary investigation of the Spence children's anxiety parent scale as a screening tool for anxiety in young people with autism spectrum disorders. *Journal of Autism and Developmental Disorders, 44(8)*, 1982-1994. doi:http://dx.doi.org/10.1007/s10803-014-2075-0. Exclusion reason: No psychometric information.

Zheng, L., Grove, R., & Eapen, V. (2019). Predictors of maternal stress in pre-school and school-aged children with autism. *Journal of Intellectual and Developmental Disability, 44*(2), 202-211. doi:https://dx.doi.org/10.3109/13668250.2017.1374931. Exclusion reason: No psychometric information.

Zlomke, K. R., & Jeter, K. (2020). Comparative Effectiveness of Parent-Child Interaction Therapy for Children with and Without Autism Spectrum Disorder. *Journal of Autism and Developmental Disorders, 50(6)*, 2041-2052. doi:http://dx.doi.org/10.1007/s10803-019-03960-y. Exclusion reason: Sample size = < 20.

Zody, M. C. (2018). Acceptance and Commitment Therapy (ACT) as a one-day workshop for parents of children with an Autism Spectrum Disorder. *Dissertation Abstracts International: Section B: The Sciences and Engineering, 78*(10-B(E)), No Pagination Specified. Exclusion reason: PhD dissertation.

Zukerman, G., Yahav, G., & Ben-Itzchak, E. (2019). Increased psychiatric symptoms in university students with autism spectrum disorder are associated with reduced adaptive behavior. *Psychiatry Research, 273*, 732-738. doi:http://dx.doi.org/10.1016/j.psychres.2019.01.098. Exclusion reason: Not relevant measurement tool.

Zwaigenbaum, L., Zaidman-Zait, A., Duku, E., Bennett, T., Mirenda, P., Smith, I., . . . Ungar, W. (2020). Profiles of sleep problems among young children with autism spectrum disorders. *Paediatrics and Child Health (Canada), 25(SUPPL 2)*, e23. Exclusion reason: Conference abstract.

Aathira, R., Gulati, S., Tripathi, M., Shukla, G., Chakrabarty, B., Sapra, S., . . . Pandey, R. M. (2017). Prevalence of Sleep Abnormalities in Indian Children With Autism Spectrum Disorder: A Cross-Sectional Study. *Pediatric Neurology, 74*, 62-67. doi:https://dx.doi.org/10.1016/j.pediatrneurol.2017.05.019. Exclusion reason: No psychometric information.
