# Supplementary material for: Applicability and Psychometric Properties of General Mental Health Assessment Tools in Autistic People: A Systematic Review
Source: J Autism Dev Disord. 2024 Apr 13;55(5):1713–26. doi: 10.1007/s10803-024-06324-3 (PMC12021962; doi:10.1007/s10803-024-06324-3)
Supplement: Supplementary file 4 — Supplementary file4 (DOCX 16 KB) [file 10803_2024_6324_MOESM4_ESM.docx]

**Appendix D**

*Risk of Bias Items and Criteria Explanation*

| Risk of bias items | Criteria description | Description for low-risk rating | Description of high-risk scoring |
| --- | --- | --- | --- |
| 1. Was the participants representative of the participants who will receive the test in practice? | The participants are similar to the sample in whom the test will be used in practice. | The judgement should be based on both the method of recruitment and the characteristics of those recruited. | Participants recruited online and where characteristics are not independently assessed are evaluated as high risk. |
| 1. Were selection criteria clearly   described? | Inclusion and exclusion criteria have to be clearly described. | All relevant information regarding how participants were selected for inclusion in the study has been provided. | Lack of inclusion and exclusion criteria or very unclear inclusion criteria is evaluated as high risk. |
| 1. Did the whole sample or a random selection of the sample, receive verification of the autism diagnosis using a reference standard of diagnosis? | Is the ASD diagnosis confirmed by a standardised assessment tool, screening instrument or clinically confirmed. | It is clear from the study that all participants, or a random selection of participants, who received the scale under review went on to receive verification of their ASD diagnosis using reference standard. | If the ASD diagnosis only is self-report and no verification has been provided, it is evaluated as high risk. |
| 1. Was the execution of the scale under review described in sufficient detail to permit replication of the test? | A sufficient description of the execution of the instrument explaining all the process and instructions in detail. | The study reports sufficient details or citations to permit replication of the scale under review. | Lack of details or descriptions of how the scale under review was performed. |
| 1. Were withdrawals from the study explained? | The process of how participants withdrew from the study is known. | It is clear what happened to all participants who entered the study. | It is unclear whether any participants withdrew from assessment of the index test. |
| 1. Is it clearly stated where the sample was obtained? | The information regarding the place (country, city, centre/ hospital…) appears in the procedure section. | The information is available and where the sample was obtained is clear. | No information on where the study was performed |
| 1. Is it clearly specified when the sample was obtained? | The information regarding the date (month and year) appears in the procedure section. | The information is available and the information is clear. | No information on when the study was performed or unclear when it was performed. |
| 1. Are the statistically analyses fully described? | In the method section, the statistical analysis part fully describes all analysis performed and specifies the correct scores provided. | If the corrected scores facilitated are specified and the statistical analysis performed appear in the specific section, and are clearly explained. | If the statistical analysis is not fully described in the method section or if it is unclear how the analysis was performed. |
| 1. Are the limitations of the article specifically addressed? | The article includes information regarding all study limitations. | If in the discussion a specific part mentions the article limitation and relevant limitations are mentioned. | If limitations are not mentioned in the discussion or if the limitations do not include obvious limitations. |
