# Supplementary material for: Applicability and Psychometric Properties of General Mental Health Assessment Tools in Autistic People: A Systematic Review
Source: J Autism Dev Disord. 2024 Apr 13;55(5):1713–26. doi: 10.1007/s10803-024-06324-3 (PMC12021962; doi:10.1007/s10803-024-06324-3)
Supplement: Supplementary file 5 — Supplementary file5 (DOCX 43 KB) [file 10803_2024_6324_MOESM5_ESM.docx]

**Appendix E**

**Included studies**

Adams, D., Paynter, J., Clark, M., Roberts, J., & Keen, D. (2019). The Developmental Behaviour Checklist (DBC) Profile in Young Children on the Autism Spectrum: The Impact of Child and Family Factors. Journal of Autism & Developmental Disorders, 49(8), 3426-3439. <https://doi.org/https://dx.doi.org/10.1007/s10803-019-04067-0>

Alallawi, B., Hastings, R., & Aabe, N. (2022). Support Needs and Parent Outcomes in Arab Families of Children with Autism Living in the United Kingdom. *Brain Sciences, 12*(8), 22. <https://dx.doi.org/10.3390/brainsci12081114>

Bacherini, A., Igliozzi, R., Cagiano, R., Mancini, A., Tancredi, R., Muratori, F., & Balboni, G. (2021). Behavioral and emotional problems of toddlers with autism spectrum disorder: Effects of parents' sociocultural level and individual factors. Research in Developmental Disabilities, 119, 104106. <https://dx.doi.org/10.1016/j.ridd.2021.104106>

Baker, B. L., & Blacher, J. (2015). Disruptive behavior disorders in adolescents with ASD: Comparisons to youth with intellectual disability or typical cognitive development. Journal of Mental Health Research in Intellectual Disabilities, 8(2), 98-116. <https://dx.doi.org/10.1080/19315864.2015.1018395>

Bakken, T. L., Helverschou, S. B., Eilertsen, D. E., Heggelund, T., Myrbakk, E., & Martinsen, H. (2010). Psychiatric disorders in adolescents and adults with autism and intellectual disability: a representative study in one county in Norway. Research in Developmental Disabilities, 31(6), 1669-1677. <https://dx.doi.org/10.1016/j.ridd.2010.04.009>

Bakken, T. L., Kildahl, A. N., Ludvigsen, L. B., Bjorgen, T. G., Dalhaug, C., Hellerud, J. M. A., . . . Helverschou, S. B. (2023). Schizophrenia in autistic people with intellectual disabilities: Symptom manifestations and identification. *Journal of Applied Research in Intellectual Disabilities, 36*(5), 1076-1091. <https://dx.doi.org/10.1111/jar.13127>

Bangerter, A., Ness, S., Aman, M. G., Esbensen, A. J., Goodwin, M. S., Dawson, G., Hendren, R., Leventhal, B., Khan, A., Opler, M., Harris, A., & Pandina, G. (2017). Autism Behavior Inventory: A Novel Tool for Assessing Core and Associated Symptoms of Autism Spectrum Disorder. Journal of Child & Adolescent Psychopharmacology, 27(9), 814-822. <https://dx.doi.org/10.1089/cap.2017.0018>

Beer, M., Ward, L., & Moar, K. (2013). The relationship between mindful parenting and distress in parents of children with an autism spectrum disorder. Mindfulness, 4(2), 102-112. <https://dx.doi.org/10.1007/s12671-012-0192-4>

Bekhet, A. K. (2016). The mediating effects of positive cognitions on autism caregivers' depression and their children's challenging behaviors. Archives of Psychiatric Nursing, 30(1), 13-18. [/https://dx.doi.org/10.1016/j.apnu.2015.11.001](https://doi.org/https://dx.doi.org/10.1016/j.apnu.2015.11.001)

Benson, P. R. (2015). Longitudinal effects of educational involvement on parent and family functioning among mothers of children with ASD. Research in Autism Spectrum Disorders, 11, 42-55. <https://dx.doi.org/10.1016/j.rasd.2014.11.011>

Bitsika, V., & Sharpley, C. F. (2016). Which aspects of challenging behaviour are associated with anxiety across two age groups of young males with an autism spectrum disorder? Journal of Developmental and Physical Disabilities, 28(5), 685-701. <https://dx.doi.org/10.1007/s10882-016-9502-4>

Bitsika, V., & Sharpley, C. F. (2017). How is challenging behaviour associated with depression in boys with an Autism Spectrum Disorder? International Journal of Disability, Development and Education, 64(4), 391-403. <https://dx.doi.org/10.1080/1034912X.2016.1250872>

Bitsika, V., Sharpley, C. F., Andronicos, N. M., & Agnew, L. L. (2016). Prevalence, structure and correlates of anxiety-depression in boys with an autism spectrum disorder. Research in Developmental Disabilities, 49-50, 302-311. <http://dx.doi.org/10.1016/j.ridd.2015.11.011>

Brinkley, J., Nations, L., Abramson, R. K., Hall, A., Wright, H. H., Gabriels, R., Gilbert, J. R., Pericak-Vance, M. A. O., & Cuccaro, M. L. (2007). Factor analysis of the aberrant behavior checklist in individuals with autism spectrum disorders. Journal of Autism and Developmental Disorders, 37(10), 1949-1959. <http://dx.doi.org/10.1007/s10803-006-0327-3>

Brookman-Frazee, L., Stadnick, N., Chlebowski, C., Baker-Ericzen, M., & Ganger, W. (2018). Characterizing psychiatric comorbidity in children with autism spectrum disorder receiving publicly funded mental health services. Autism, 22(8), 938-952. <https://dx.doi.org/10.1177/1362361317712650>

Buck, T. R., Viskochil, J., Farley, M., Coon, H., McMahon, W. M., Morgan, J., & Bilder, D. A. (2014). Psychiatric comorbidity and medication use in adults with autism spectrum disorder. Journal of Autism and Developmental Disorders, 44(12), 3063-3071. <https://dx.doi.org/10.1007/s10803-014-2170-2>

Burton, T., Ratcliffe, B., Collison, J., Dossetor, D., & Wong, M. (2020). Self-reported emotion regulation in children with autism spectrum disorder, without intellectual disability. *Research in Autism Spectrum Disorders,76, 2020, ArtID 101599, 76*. <https://dx.doi.org/10.1016/j.rasd.2020.101599>

Byrne, G., Ghrada, A. N., & O'Mahony, T. (2023). Parent-led cognitive behavioural therapy for children with autism spectrum conditions. A pilot study. *Journal of Autism and Developmental Disorders, 53*(1), 263-274. https://dx.doi.org/10.1007/s10803-022-05424-2

Chan, K. K. S., Wang, Z., & Li, H. (2022). Longitudinal impact of mindful parenting on internalizing and externalizing symptoms among children with autism spectrum disorder. *Mindfulness, 13*(8), 1984-1993. <https://dx.doi.org/10.1007/s12671-022-01933-4>

Chandler, S., Howlin, P., Simonoff, E., O'Sullivan, T., Tseng, E., Kennedy, J., Charman, T., & Baird, G. (2016). Emotional and behavioural problems in young children with autism spectrum disorder. Developmental Medicine & Child Neurology, 58(2), 202-208. <https://dx.doi.org/10.1111/dmcn.12830>

Charlton, A. S., Smith, I. C., Mazefsky, C. A., & White, S. W. (2020). The role of emotion regulation on co-occurring psychopathology in emerging adults with ASD. Journal of Autism and Developmental Disorders, 50(7), 2585-2592. <https://dx.doi.org/10.1007/s10803-019-03983-5>

Cheng, Y. L., Chu, C. L., & Wu, C. C. (2022). How Do Children with Autism Spectrum Disorder and Children with Developmental Delays Differ on the Child Behavior Checklist 1.5-5 DSM-Oriented Scales? Children, 9(1), 14. <https://dx.doi.org/10.3390/children9010111>

Cheng, Y. L., Iao, L. S., & Wu, C. C. (2021). Comparison of the Factor Structure of the Child Behavior Checklist 1.5-5 between Children with ASD and Children with DD. Research in Autism Spectrum Disorders, 89 (no pagination)(101867). <https://dx.doi.org/10.1016/j.rasd.2021.101867>

Chua, S. Y., Abd Rahman, F. N., & Ratnasingam, S. (2023). Problem behaviours and caregiver burden among children with Autism Spectrum Disorder in Kuching, Sarawak. *Frontiers in psychiatry Frontiers Research Foundation, 14*, 1244164. <https://dx.doi.org/10.3389/fpsyt.2023.1244164>

Chung, K. M., & Jung, D. (2017). Validity and reliability of the Korean version of Autism spectrum disorders-comorbid for children (ASD-CC). Research in Autism Spectrum Disorders, 39, 1-10. <http://dx.doi.org/10.1016/j.rasd.2017.03.006>

Clauser, P., Ding, Y., Chen, E. C., Cho, S.-J., Wang, C., & Hwang, J. (2021). Parenting styles, parenting stress, and behavioral outcomes in children with autism. *School Psychology International, 42*(1), 33-56. <https://dx.doi.org/10.1177/0143034320971675>

Davis, N. O., & Carter, A. S. (2008). Parenting stress in mothers and fathers of toddlers with autism spectrum disorders: associations with child characteristics. Journal of Autism & Developmental Disorders, 38(7), 1278-1291. <https://dx.doi.org/10.1007/s10803-007-0512-z>

Day, T. N., Mazefsky, C. A., Yu, L., Zeglen, K. N., Neece, C. L., & Pilkonis, P. A. (2023). The emotion dysregulation inventory-young child: Psychometric properties and item response theory calibration in 2- to 5-year-olds.PS - First Posting. *Journal of the American Academy of Child & Adolescent Psychiatry*, No Pagination Specified. <https://dx.doi.org/10.1016/j.jaac.2023.04.021>

De Clercq, L., Van der Kaap-Deeder, J., Dieleman, L. M., Soenens, B., Prinzie, P., & De Pauw, S. S. (2019). Parenting and psychosocial development in youth with and without autism spectrum disorder, cerebral palsy, and Down syndrome: A cross-disability comparison. Advances in Neurodevelopmental Disorders, 3(2), 220-234. <https://dx.doi.org/10.1007/s41252-019-00112-2>

De Clercq, L. E., Dieleman, L. M., van der Kaap-Deeder, J., Soenens, B., Prinzie, P., & De Pauw, S. S. (2021). Negative controlling parenting and child personality as modifiers of psychosocial development in youth with autism spectrum disorder: A 9-year longitudinal study at the level of within-person change. Journal of Autism and Developmental Disorders, 51(8), 2891-2907. <https://dx.doi.org/10.1007/s10803-020-04761-4>

De Pauw, S. S., Mervielde, I., Van Leeuwen, K. G., & De Clercq, B. J. (2011). How temperament and personality contribute to the maladjustment of children with autism. Journal of Autism and Developmental Disorders, 41(2), 196-212. <https://dx.doi.org/10.1007/s10803-010-1043-6>

Deniz, E., & Toseeb, U. (2023). A longitudinal study of sibling bullying and mental health in autistic adolescents: The role of self-esteem.DP - Aug 2023. *Autism Research, 16*(8), 1533-1549. <https://dx.doi.org/10.1002/aur.2987>

Dieleman, L. M., De Pauw, S. S., Soenens, B., Beyers, W., & Prinzie, P. (2017). Examining bidirectional relationships between parenting and child maladjustment in youth with autism spectrum disorder: A 9-year longitudinal study. Development and psychopathology, 29(4), 1199-1213. <https://dx.doi.org/10.1017/S0954579416001243>

Dieleman, L. M., De Pauw, S. S., Soenens, B., Mabbe, E., Campbell, R., & Prinzie, P. (2018). Relations between problem behaviors, perceived symptom severity and parenting in adolescents and emerging adults with ASD: The mediating role of parental psychological need frustration. Research in Developmental Disabilities, 73, 21-30. <https://dx.doi.org/10.1016/j.ridd.2017.12.012>

Dovgan, K., Mazurek, M. O., & Hansen, J. (2019). Measurement invariance of the child behavior checklist in children with autism spectrum disorder with and without intellectual disability: Follow-up study. Research in Autism Spectrum Disorders, 58, 19-29. <http://dx.doi.org/10.1016/j.rasd.2018.11.009>

Farmer, C. A., Kaat, A. J., Mazurek, M. O., Lainhart, J. E., DeWitt, M. B., Cook, E. H., Butter, E. M., & Aman, M. G. (2016). Confirmation of the Factor Structure and Measurement Invariance of the Children's Scale of Hostility and Aggression: Reactive/Proactive in Clinic-Referred Children With and Without Autism Spectrum Disorder. Journal of Child & Adolescent Psychopharmacology, 26(1), 10-18. <https://dx.doi.org/10.1089/cap.2015.0098>

Findon, J., Cadman, T., Stewart, C. S., Woodhouse, E., Eklund, H., Hayward, H., De Le Harpe Golden, D., Chaplin, E., Glaser, K., Simonoff, E., Murphy, D., Bolton, P. F., & McEwen, F. S. (2016). Screening for co-occurring conditions in adults with autism spectrum disorder using the strengths and difficulties questionnaire: A pilot study. Autism research : Official Journal of the International Society for Autism Research, 9(12), 1353-1363. <https://dx.doi.org/10.1002/aur.1625>

Firth, I., & Dryer, R. (2013). The predictors of distress in parents of children with autism spectrum disorder. Journal of Intellectual and Developmental Disability, 38(2), 163-171. <https://dx.doi.org/10.3109/13668250.2013.773964>

Fok, M., & Bal, V. H. (2019). Differences in profiles of emotional behavioral problems across instruments in verbal versus minimally verbal children with autism spectrum disorder. Autism research : Official Journal of the International Society for Autism Research, 12(9), 1367-1375. <https://dx.doi.org/10.1002/aur.2126>

Fong, V. C., Gardiner, E., & Iarocci, G. (2020). Can a combination of mental health services and ADL therapies improve quality of life in families of children with autism spectrum disorder? Quality of Life Research, 29(8), 2161-2170. <https://dx.doi.org/10.1007/s11136-020-02440-6>

Fong, V., Gardiner, E., & Iarocci, G. (2021). Satisfaction with informal supports predicts resilience in families of children with autism spectrum disorder. *Autism, 25*(2), 452-463. <https://dx.doi.org/10.1177/1362361320962677>

Gjevik, E., Eldevik, S., Fjaeran-Granum, T., & Sponheim, E. (2011). Kiddie-SADS reveals high rates of DSM-IV disorders in children and adolescents with autism spectrum disorders. Journal of Autism & Developmental Disorders, 41(6), 761-769. <https://dx.doi.org/10.1007/s10803-010-1095-7>

Gjevik, E., Sandstad, B., Andreassen, O. A., Myhre, A. M., & Sponheim, E. (2015). Exploring the agreement between questionnaire information and DSM-IV diagnoses of comorbid psychopathology in children with autism spectrum disorders. Autism, 19(4), 433-442. <https://dx.doi.org/10.1177/1362361314526003>

Graziosi, G., & Perry, A. (2023). Age and sex differences in problem behaviours in youth with autism spectrum disorder. *Research in Autism Spectrum Disorders, 100*, No Pagination Specified. <https://dx.doi.org/10.1016/j.rasd.2022.102075>

Hastings, R. P., Kovshoff, H., Ward, N. J., degli Espinosa, F., Brown, T., & Remington, B. (2005). Systems Analysis of Stress and Positive Perceptions in Mothers and Fathers of Pre-School Children with Autism. Journal of Autism and Developmental Disorders, 35(5), 635-644. <https://dx.doi.org/10.1007/s10803-005-0007-8>

Hastings, S. E., Hastings, R. A., Swales, M. A., & Hughes, J. C. (2022). Emotional and behavioural problems of children with autism spectrum disorder attending mainstream schools. International Journal of Developmental Disabilities. <https://dx.doi.org/10.1080/20473869.2020.1869414>

Helverschou, S. B., Bakken, T. L., & Martinsen, H. (2009). The Psychopathology in Autism Checklist (PAC): A pilot study. Research in Autism Spectrum Disorders, 3(1), 179-195. <http://dx.doi.org/10.1016/j.rasd.2008.05.004>

Helverschou, S. B., Ludvigsen, L. B., Hove, O., & Kildahl, A. N. (2021). Psychometric properties of the Psychopathology in Autism Checklist (PAC). International Journal of Developmental Disabilities, 67(5), 318-326. <https://dx.doi.org/10.1080/20473869.2021.1910779>

Hepburn, S. L., Stern, J. A., Blakeley-Smith, A., Kimel, L. K., & Reaven, J. A. (2014). Complex psychiatric comorbidity of treatment-seeking youth with autism spectrum disorder and anxiety symptoms. Journal of Mental Health Research in Intellectual Disabilities, 7(4), 359-378. <https://dx.doi.org/10.1080/19315864.2014.932476>

Higgins, L., Mannion, A., Chen, J. L., & Leader, G. (2023). Adaptation of parents raising a child with ASD: The role of positive perceptions, coping, self-efficacy, and social support. *Journal of Autism and Developmental Disorders, 53*(3), 1224-1242. <https://dx.doi.org/10.1007/s10803-022-05537-8>

Horovitz, M., & Matson, J. L. (2013). The baby and infant screen for children with aUtIsm Traits-Part 3: The development of age-based scoring procedures. Research in Autism Spectrum Disorders, 7(11), 1291-1299. <http://dx.doi.org/10.1016/j.rasd.2013.07.019>

Horovitz, M., & Matson, J. L. (2015). The baby and infant screen for children with autism traits-part 2: The development of age-based cutoffs. Developmental neurorehabilitation, 18(6), 349-356. <https://dx.doi.org/10.3109/17518423.2013.797031>

Hurtig, T., Kuusikko, S., Mattila, M. L., Haapsamo, H., Ebeling, H., Jussila, K., Joskitt, L., Pauls, D., & Moilanen, I. (2009). Multi-informant reports of psychiatric symptoms among high-functioning adolescents with Asperger syndrome or autism. Autism, 13(6), 583-598. <https://dx.doi.org/10.1177/1362361309335719>

Jellett, R., Wood, C. E., Giallo, R., & Seymour, M. (2015). Family functioning and behaviour problems in children with Autism Spectrum Disorders: The mediating role of parent mental health. Clinical Psychologist, 19(1), 39-48. <https://dx.doi.org/10.1111/cp.12047>

Jepsen, M. I., Gray, K. M., & Taffe, J. R. (2012). Agreement in multi-informant assessment of behaviour and emotional problems and social functioning in adolescents with autistic and Asperger's disorder. Research in Autism Spectrum Disorders, 6(3), 1091-1098. <http://dx.doi.org/10.1016/j.rasd.2012.02.008>

Jeter, K., Zlomke, K., Shawler, P., & Sullivan, M. (2017). Comprehensive Psychometric Analysis of the Eyberg Child Behavior Inventory in Children with Autism Spectrum Disorder. Journal of Autism & Developmental Disorders, 47(5), 1354-1368. <https://dx.doi.org/10.1007/s10803-017-3048-x>

Jones, L., Hastings, R. P., Totsika, V., Keane, L., & Rhule, N. (2014). Child behavior problems and parental well-being in families of children with autism: The mediating role of mindfulness and acceptance. American journal on intellectual and developmental disabilities, 119(2), 171-185. <https://dx.doi.org/10.1352/1944-7558-119.2.171>

Kalb, L. G., Hagopian, L. P., Gross, A. L., & Vasa, R. A. (2018). Psychometric characteristics of the mental health crisis assessment scale in youth with autism spectrum disorder. Journal of Child Psychology & Psychiatry & Allied Disciplines, 59(1), 48-56. <https://dx.doi.org/10.1111/jcpp.12748>

Kang, Y. Q., Song, X. R., Wang, G. F., Su, Y. Y., Li, P. Y., & Zhang, X. (2020). Sleep Problems Influence Emotional/Behavioral Symptoms and Repetitive Behavior in Preschool-Aged Children With Autism Spectrum Disorder in the Unique Social Context of China. Frontiers in psychiatry Frontiers Research Foundation, 11, 273. <https://dx.doi.org/10.3389/fpsyt.2020.00273>

Kanne, S. M., Abbacchi, A. M., & Constantino, J. N. (2009). Multi-informant ratings of psychiatric symptom severity in children with autism spectrum disorders: The importance of environmental context. Journal of Autism and Developmental Disorders, 39(6), 856-864. <https://dx.doi.org/10.1007/s10803-009-0694-7>

Kat, S., Xu, L., Guo, Y., Ma, J., Ma, Z., Tang, X., Yang, Y., Wang, H., Li, X., & Liu, J. (2020). Reliability and Validity of the Simplified Chinese Version of the Aberrant Behavior Checklist in Chinese Autism Population. Frontiers in psychiatry Frontiers Research Foundation, 11, 545445. <https://dx.doi.org/10.3389/fpsyt.2020.545445>

Keefer, A., Singh, V., Kalb, L. G., Mazefsky, C. A., & Vasa, R. A. (2020). Investigating the factor structure of the child behavior checklist dysregulation profile in children and adolescents with autism spectrum disorder. Autism research : Official Journal of the International Society for Autism Research, 13(3), 436-443. <https://dx.doi.org/10.1002/aur.2233>

Khalfe, N., Goetz, A. R., Trent, E. S., Guzick, A. G., Smarason, O., Kook, M., . . . Storch, E. A. (2023). Psychometric properties of the revised children's anxiety and depression scale (RCADS) for autistic youth without co-occurring intellectual disability. *Journal of Mood and Anxiety Disorders, 2*. <https://dx.doi.org/10.1016/j.xjmad.2023.100017>

Khor, A. S., Melvin, G. A., Reid, S. C., & Gray, K. M. (2014). Coping, daily hassles and behavior and emotional problems in adolescents with high-functioning autism/Asperger's disorder. Journal of Autism and Developmental Disorders, 44(3), 593-608. <https://dx.doi.org/10.1007/s10803-013-1912-x>

Kildahl, A. N., & Helverschou, S. B. (2023). Post-traumatic stress disorder and experiences involving violence or sexual abuse in a clinical sample of autistic adults with intellectual disabilities: Prevalence and clinical correlates. *Autism*, 13623613231190948. <https://dx.doi.org/10.1177/13623613231190948>

Kildahl, A. N., Ludvigsen, L. B., Hove, O., & Helverschou, S. B. (2023). Exploring the relationship between challenging behaviour and mental health disorder in autistic individuals with intellectual disabilities. *Research in Autism Spectrum Disorders, 104*, 102147. <https://doi.org/10.1016/j.rasd.2023.102147>

Kim, J. I., Shin, M. S., Lee, Y., Lee, H., Yoo, H. J., Kim, S. Y., Kim, H., Kim, S. J., & Kim, B. N. (2018). Reliability and Validity of a New Comprehensive Tool for Assessing Challenging Behaviors in Autism Spectrum Disorder. Psychiatry Investigation, 15(1), 54-61. <https://dx.doi.org/10.4306/pi.2018.15.1.54>

Kirst, S., Bogl, K., Gross, V. L., Diehm, R., Poustka, L., & Dziobek, I. (2022). Subtypes of aggressive behavior in children with autism in the context of emotion recognition, hostile attribution bias, and dysfunctional emotion regulation. *Journal of Autism and Developmental Disorders, 52*(12), 5367-5382. <https://dx.doi.org/10.1007/s10803-021-05387-w>

Koller, J., David, T., Bar, N., & Lebowitz, E. R. (2022). The role of family accommodation of RRBs in disruptive behavior among children with autism. *Journal of Autism and Developmental Disorders, 52*(6), 2505-2511. <https://dx.doi.org/10.1007/s10803-021-05163-w>

Kaat, A. J., Gadow, K. D., & Lecavalier, L. (2013). Psychiatric symptom impairment in children with autism spectrum disorders. Journal of Abnormal Child Psychology, 41(6), 959-969. <https://dx.doi.org/10.1007/s10802-013-9739-7>

Kaat, A. J., & Lecavalier, L. (2015). Reliability and validity of parent- and child-rated anxiety measures in autism spectrum disorder. Journal of Autism & Developmental Disorders, 45(10), 3219-3231. <https://dx.doi.org/10.1007/s10803-015-2481-y>

Kaat, A. J., Lecavalier, L., & Aman, M. G. (2014). Validity of the aberrant behavior checklist in children with autism spectrum disorder. Journal of Autism & Developmental Disorders, 44(5), 1103-1116. <https://dx.doi.org/10.1007/s10803-013-1970-0>

La Buissonniere Ariza, V., Schneider, S. C., Cepeda, S. L., Wood, J. J., Kendall, P. C., Small, B. J., . . . Storch, E. A. (2022). Predictors of suicidal thoughts in children with autism spectrum disorder and anxiety or obsessive-compulsive disorder: The unique contribution of externalizing behaviors. *Child Psychiatry and Human Development, 53*(2), 223-236. <https://dx.doi.org/10.1007/s10578-020-01114-1>

Lane, B. R., Paynter, J., & Sharman, R. (2013). Parent and teacher ratings of adaptive and challenging behaviours in young children with autism spectrum disorders. Research in Autism Spectrum Disorders, 7(10), 1196-1203. <https://dx.doi.org/10.1016/j.rasd.2013.07.011>

Leader, G., Dooley, E., Whelan, S., Gilroy, S. P., Chen, J. L., Farren Barton, A., Coyne, R., & Mannion, A. (2021). Attention-Deficit/Hyperactivity Disorder Symptoms, Gastrointestinal Symptoms, Sleep Problems, Challenging Behavior, Adaptive Behavior, and Quality of Life in Children and Adolescents with Autism Spectrum Disorder. Developmental neurorehabilitation, 1-12. <https://dx.doi.org/10.1080/17518423.2021.1964005>

Leader, G., Flynn, C., O'Rourke, N., Coyne, R., Caher, A., & Mannion, A. (2021). Comorbid Psychopathology, Challenging Behavior, Sensory Issues, Adaptive Behavior and Quality of Life in Children and Adolescents with Autism Spectrum Disorder. Developmental neurorehabilitation, 24(6), 397-407. <https://dx.doi.org/10.1080/17518423.2021.1898058>

Leader, G., Francis, K., Mannion, A., & Chen, J. (2018). Toileting problems in children and adolescents with parent-reported diagnoses of autism spectrum disorder. Journal of Developmental and Physical Disabilities, 30(3), 307-327. <https://dx.doi.org/10.1007/s10882-018-9587-z>

Leader, G., Moore, R., Chen, J. L., Caher, A., Arndt, S., Maher, L., . . . Mannion, A. (2022). Attention deficit hyperactivity disorder (ADHD) symptoms, comorbid psychopathology, behaviour problems and gastrointestinal symptoms in children and adolescents with autism spectrum disorder. *Irish Journal of Psychological Medicine, 39*(3), 240-250. <https://dx.doi.org/10.1017/ipm.2020.135>

Lecavalier, L., Aman, M. G., Hammer, D., Stoica, W., & Mathews, G. L. (2004). Factor analysis of the Nisonger Child Behavior Rating Form in children with autism spectrum disorders. Journal of Autism & Developmental Disorders, 34(6), 709-721. <https://doi.org/10.1007/s10803-004-5291-1>

Lecavalier, L., Gadow, K. D., DeVincent, C. J., & Edwards, M. C. (2009). Validation of DSM-IV model of psychiatric syndromes in children with autism spectrum disorders. Journal of Autism and Developmental Disorders, 39(2), 278-289. <https://dx.doi.org/10.1007/s10803-008-0622-2>

Lecavalier, L., Gadow, K. D., Devincent, C. J., Houts, C. R., & Edwards, M. C. (2011). Validity of DSM-IV syndromes in preschoolers with autism spectrum disorders. Autism, 15(5), 527-543. <https://dx.doi.org/10.1177/1362361310391115>

Lecavalier, L., Leone, S., & Wiltz, J. (2006). The impact of behaviour problems on caregiver stress in young people with autism spectrum disorders. Journal of Intellectual Disability Research, 50(3), 172-183. <https://dx.doi.org/10.1111/j.1365-2788.2005.00732.x>

Leyfer, O. T., Folstein, S. E., Bacalman, S., Davis, N. O., Dinh, E., Morgan, J., Tager-Flusberg, H., & Lainhart, J. E. (2006). Comorbid psychiatric disorders in children with autism: interview development and rates of disorders. Journal of Autism & Developmental Disorders, 36(7), 849-861. <https://doi.org/10.1007/s10803-006-0123-0>

Lovell, B., & Wetherell, M. A. (2016). Behaviour problems of children with ASD and perceived stress in their caregivers: The moderating role of trait emotional intelligence? Research in Autism Spectrum Disorders, 28, 1-6. <https://dx.doi.org/10.1016/j.rasd.2016.05.002>

Lovell, B., & Wetherell, M. A. (2020). Exploring the moderating role of benefit finding on the relationship between child problematic behaviours and psychological distress in caregivers of children with ASD. Journal of Autism and Developmental Disorders, 50(2), 617-624. <https://dx.doi.org/10.1007/s10803-019-04300-w>

LoVullo, S. V., & Matson, J. L. (2009). Comorbid psychopathology in adults with Autism Spectrum Disorders and intellectual disabilities. Research in Developmental Disabilities, 30(6), 1288-1296. <https://dx.doi.org/10.1016/j.ridd.2009.05.004>

Lu, M., Chen, J., He, W., Pang, F., & Zou, Y. (2021). Association between perceived social support of parents and emotional/behavioral problems in children with ASD: A chain mediation model. Research in Developmental Disabilities, 113, 103933. <https://dx.doi.org/10.1016/j.ridd.2021.103933>

Lu, M., Wang, R., Lin, H., Pang, F., & Chen, X. (2021). Perceived social support and life satisfaction of Chinese parents of children with autism spectrum disorder: Loneliness as a mediator and moderator. Research in Autism Spectrum Disorders, 87 (no pagination)(101829). <http://dx.doi.org/10.1016/j.rasd.2021.101829>

Magiati, I., Ong, C., Lim, X. Y., Tan, J. W.-L., Ong, A. Y. L., Patrycia, F., Fung, D. S. S., Sung, M., Poon, K. K., & Howlin, P. (2016). Anxiety symptoms in young people with autism spectrum disorder attending special schools: Associations with gender, adaptive functioning and autism symptomatology. Autism, 20(3), 306-320. <https://dx.doi.org/10.1177/1362361315577519>

Magyar, C. I., & Pandolfi, V. (2017). Utility of the CBCL DSM-Oriented Scales in Assessing Emotional Disorders in Youth with Autism. Research in Autism Spectrum Disorders, 37, 11-20. <https://dx.doi.org/10.1016/j.rasd.2017.01.009>

Mahan, S., & Matson, J. L. (2011). Convergent and discriminant validity of the Autism Spectrum Disorder-Problem Behavior for Children (ASD-PBC) against the Behavioral Assessment System for Children, Second Edition (BASC-2). Research in Autism Spectrum Disorders, 5(1), 222-229. <https://dx.doi.org/10.1016/j.rasd.2010.04.003>

Manning, M. M., Wainwright, L., & Bennett, J. (2011). The Double ABCX model of adaptation in racially diverse families with a school-age child with autism. Journal of Autism and Developmental Disorders, 41(3), 320-331. <https://dx.doi.org/10.1007/s10803-010-1056-1>

Mannion, A., & Leader, G. (2013). An analysis of the predictors of comorbid psychopathology, gastrointestinal symptoms and epilepsy in children and adolescents with autism spectrum disorder. Research in Autism Spectrum Disorders, 7(12), 1663-1671. <http://dx.doi.org/10.1016/j.rasd.2013.10.002>

Martinez, K., Chlebowski, C., Roesch, S., Stadnick, N. A., Villodas, M., & Brookman-Frazee, L. (2023). Psychometric assessment of the Eyberg Child Behavior Inventory in children with autism in community settings. *Journal of Autism and Developmental Disorders, 53*(4), 1693-1705. <https://dx.doi.org/10.1007/s10803-022-05427-z>

Matson, J. L., Boisjoli, J., Rojahn, J., & Hess, J. (2009). A factor analysis of challenging behaviors assessed with the Baby and Infant Screen for Children with aUtism Traits (BISCUIT-Part 3). Research in Autism Spectrum Disorders, 3(3), 714-722. <http://dx.doi.org/10.1016/j.rasd.2009.01.008>

Matson, J. L., & Boisjoli, J. A. (2008). Autism spectrum disorders in adults with intellectual disability and comorbid psychopathology: Scale development and reliability of the ASD-CA. Research in Autism Spectrum Disorders, 2(2), 276-287. <http://dx.doi.org/10.1016/j.rasd.2007.07.002>

Matson, J. L., Boisjoli, J. A., Hess, J. A., & Wilkins, J. (2011). Comorbid psychopathology factor structure on the Baby and Infant Screen for Children with aUtIsm Traits-Part 2 (BISCUIT-Part 2). Research in Autism Spectrum Disorders, 5(1), 426-432. <http://dx.doi.org/10.1016/j.rasd.2010.06.005>

Matson, J. L., Fodstad, J. C., Mahan, S., & Sevin, J. A. (2009). Cutoffs, norms, and patterns of comorbid difficulties in children with an ASD on the Baby and Infant Screen for Children with aUtIsm Traits (BISCUIT-Part 2). Research in Autism Spectrum Disorders, 3(4), 977-988. <http://dx.doi.org/10.1016/j.rasd.2009.06.001>

Matson, J. L., LoVullo, S. V., Rivet, T. T., & Boisjoli, J. A. (2009). Validity of the Autism Spectrum Disorder-Comorbid for Children (ASD-CC). Research in Autism Spectrum Disorders, 3(2), 345-357. <http://dx.doi.org/10.1016/j.rasd.2008.08.002>

Matson, J. L., & Wilkins, J. (2008). Reliability of the Autism Spectrum Disorders-Comorbid for Children (ASD-CC). Journal of Developmental and Physical Disabilities, 20(4), 327-336. <https://dx.doi.org/10.1007/s10882-008-9100-1>

Mattila, M. L., Hurtig, T., Haapsamo, H., Jussila, K., Kuusikko-Gauffin, S., Kielinen, M., Linna, S. L., Ebeling, H., Bloigu, R., Joskitt, L., Pauls, D. L., & Moilanen, I. (2010). Comorbid psychiatric disorders associated with Asperger syndrome/high-functioning autism: a community- and clinic-based study. Journal of Autism & Developmental Disorders, 40(9), 1080-1093. <https://dx.doi.org/10.1007/s10803-010-0958-2>

Mazefsky, C. A., Borue, X., Day, T. N., & Minshew, N. J. (2014). Emotion regulation patterns in adolescents with high-functioning autism spectrum disorder: Comparison to typically developing adolescents and association with psychiatric symptoms. Autism Research, 7(3), 344-354. <http://dx.doi.org/10.1002/aur.1366>

Mazefsky, C. A., Day, T. N., Siegel, M., White, S. W., Yu, L., & Pilkonis, P. A. (2018). Development of the Emotion Dysregulation Inventory: A PROMISing method for creating sensitive and unbiased questionnaires for autism spectrum disorder. *Journal of Autism and Developmental Disorders, 48*(11), 3736-3746. <https://dx.doi.org/10.1007/s10803-016-2907-1>

Mazefsky, C. A., Oswald, D. P., Day, T. N., Eack, S. M., Minshew, N. J., & Lainhart, J. E. (2012). ASD, a psychiatric disorder, or both? Psychiatric diagnoses in adolescents with high-functioning ASD. Journal of Clinical Child & Adolescent Psychology, 41(4), 516-523. <https://dx.doi.org/10.1080/15374416.2012.686102>

Mazefsky, C. A., Yu, L., White, S. W., Siegel, M., & Pilkonis, P. A. (2018). The emotion dysregulation inventory: Psychometric properties and item response theory calibration in an autism spectrum disorder sample. *Autism research : Official Journal of the International Society for Autism Research, 11*(6), 928-941. <https://dx.doi.org/10.1002/aur.1947>

McIntyre, L. L., Santiago, R. T., Sutherland, M., & Garbacz, S. (2023). Parenting stress and autistic children's emotional problems relate to family-school partnerships and parent mental health. *School Psychology, 38*(5), 273-286. <https://dx.doi.org/10.1037/spq0000531>

Medeiros, K., Mazurek, M. O., & Kanne, S. (2017). Investigating the factor structure of the Child Behavior Checklist in a large sample of children with autism spectrum disorder. Research in Autism Spectrum Disorders, 40, 24-40. <http://dx.doi.org/10.1016/j.rasd.2017.06.001>

Mello, C., Rivard, M., Morin, D., Patel, S., & Morin, M. (2022). Symptom severity, internalized and externalized behavioral and emotional problems: Links with parenting stress in mothers of children recently diagnosed with autism. *Journal of Autism and Developmental Disorders, 52*(6), 2400-2413. <https://dx.doi.org/10.1007/s10803-021-05131-4>

Mihaila, I., & Hartley, S. L. (2018). Parental sleep quality and behavior problems of children with autism. Autism, 22(3), 236-244. <https://dx.doi.org/10.1177/1362361316673570>

Milosavljevic, B., Carter Leno, V., Simonoff, E., Baird, G., Pickles, A., Jones, C. R., Erskine, C., Charman, T., & Happe, F. (2016). Alexithymia in adolescents with autism spectrum disorder: Its relationship to internalising difficulties, sensory modulation and social cognition. Journal of Autism and Developmental Disorders, 46(4), 1354-1367. <https://dx.doi.org/10.1007/s10803-015-2670-8>

Miranda, A., Mira, A., Berenguer, C., Rosello, B., & Baixauli, I. (2019). Parenting Stress in Mothers of Children With Autism Without Intellectual Disability. Mediation of Behavioral Problems and Coping Strategies. Frontiers in Psychology, 10, 464. <https://dx.doi.org/10.3389/fpsyg.2019.00464>

Mohammadi, K., Samavi, A., Mehdiabadi, F. Z., & Samavi, S. A. (2023). Psychometric validation of concerning behavior scale in Iranian children and young people with autism spectrum disorder. *Frontiers in psychiatry Frontiers Research Foundation, 14*, 1153112. https://dx.doi.org/10.3389/fpsyt.2023.1153112

Mosner, M. G., Kinard, J. L., Shah, J. S., McWeeny, S., Greene, R. K., Lowery, S. C., Mazefsky, C. A., & Dichter, G. S. (2019). Rates of Co-occurring Psychiatric Disorders in Autism Spectrum Disorder Using the Mini International Neuropsychiatric Interview. Journal of Autism & Developmental Disorders, 49(9), 3819-3832. <https://dx.doi.org/10.1007/s10803-019-04090-1>

Nadeau, J. M., Arnold, E. B., Keene, A. C., Collier, A. B., Lewin, A. B., Murphy, T. K., & Storch, E. A. (2015). Frequency and Clinical Correlates of Sleep-Related Problems Among Anxious Youth with Autism Spectrum Disorders. Child Psychiatry & Human Development, 46(4), 558-566. <https://dx.doi.org/10.1007/s10578-014-0496-9>

Norris, M., Aman, M. G., Mazurek, M. O., Scherr, J. F., & Butter, E. M. (2019). Psychometric characteristics of the aberrant behavior checklist in a well-defined sample of youth with autism Spectrum disorder. Research in Autism Spectrum Disorders, 62, 1-9. <http://dx.doi.org/10.1016/j.rasd.2019.02.001>

Palmer, M., Paris Perez, J., Tarver, J., Cawthorne, T., Frayne, M., Webb, S., Baker, E., Yorke, I., Hay, D., Slonims, V., Pickles, A., Simonoff, E., Scott, S., & Charman, T. (2021). Development of the Observation Schedule for Children with Autism-Anxiety, Behaviour and Parenting (OSCA-ABP): A New Measure of Child and Parenting Behavior for Use with Young Autistic Children. Journal of Autism and Developmental Disorders, 51(1), 1-14. <http://dx.doi.org/10.1007/s10803-020-04506-3>

Palmer, M., Tarver, J., Carter Leno, V., Paris Perez, J., Frayne, M., Slonims, V., . . . Simonoff, E. (2023). Parent, teacher and observational reports of emotional and behavioral problems in young autistic children. *Journal of Autism and Developmental Disorders, 53*(1), 296-309. <https://dx.doi.org/10.1007/s10803-021-05421-x>

Pandolfi, V., Magyar, C. I., & Dill, C. A. (2009). Confirmatory factor analysis of the child behavior checklist 1.5-5 in a sample of children with autism spectrum disorders. Journal of Autism & Developmental Disorders, 39(7), 986-995. <https://dx.doi.org/10.1007/s10803-009-0716-5>

Pandolfi, V., Magyar, C. I., & Dill, C. A. (2012). An Initial Psychometric Evaluation of the CBCL 6-18 in a Sample of Youth with Autism Spectrum Disorders. Research in Autism Spectrum Disorders, 6(1), 96-108. <https://doi.org/10.1016/j.rasd.2011.03.009>

Pandolfi, V., Magyar, C. I., & Norris, M. (2014). Validity Study of the CBCL 6-18 for the Assessment of Emotional Problems in Youth With ASD. Journal of Mental Health Research in Intellectual Disabilities, 7(4), 306-322. <https://doi.org/10.1080/19315864.2014.930547>

Park, S. H., Song, Y. J. C., Demetriou, E. A., Pepper, K. L., Thomas, E. E., Hickie, I. B., & Guastella, A. J. (2020). Validation of the 21-item Depression, Anxiety, and Stress Scales (DASS-21) in individuals with autism spectrum disorder. Psychiatry Research, 291, 113300. <https://dx.doi.org/10.1016/j.psychres.2020.113300>

Pearson, D. A., Aman, M. G., Arnold, L. E., Lane, D. M., Loveland, K. A., Santos, C. W., Casat, C. D., Mansour, R., Jerger, S. W., Ezzell, S., Factor, P., Vanwoerden, S., Ye, E., Narain, P., & Cleveland, L. A. (2012). High concordance of parent and teacher attention-deficit/hyperactivity disorder ratings in medicated and unmedicated children with autism spectrum disorders. Journal of Child & Adolescent Psychopharmacology, 22(4), 284-291. <https://dx.doi.org/10.1089/cap.2011.0067>

Piro-Gambetti, B., Greenlee, J., Hickey, E. J., Putney, J. M., Lorang, E., & Hartley, S. L. (2023). Parental depression symptoms and internalizing mental health problems in autistic children. *Journal of Autism and Developmental Disorders, 53*(6), 2373-2383. <https://dx.doi.org/10.1007/s10803-022-05518-x>

Pisula, E., Pudlo, M., Slowinska, M., Kawa, R., Strzaska, M., Banasiak, A., & Wolanczyk, T. (2017). Behavioral and emotional problems in high-functioning girls and boys with autism spectrum disorders: Parents' reports and adolescents' self-reports. Autism, 21(6), 738-748. <https://dx.doi.org/10.1177/1362361316675119>

Plak, R., Rippe, R., Merkelbach, I., & Begeer, S. (2023). Psychosocial outcomes in autistic children before and during the covid-19 pandemic. *Journal of Autism and Developmental Disorders*, No Pagination Specified. <https://dx.doi.org/10.1007/s10803-023-06101-8>

Pozo, P., & Sarria, E. (2014). A global model of stress in parents of individuals with autism spectrum disorders (ASD). Anales de Psicologia, 30(1), 181-192. <https://doi.org/10.6018/analesps.30.1.140722>

Pruitt, M. M., Rhoden, M., & Ekas, N. V. (2018). Relationship between the broad autism phenotype, social relationships and mental health for mothers of children with autism spectrum disorder. Autism, 22(2), 171-180. <https://dx.doi.org/10.1177/1362361316669621>

Reyes, N. M., Factor, R., & Scarpa, A. (2020). Emotion regulation, emotionality, and expression of emotions: A link between social skills, behavior, and emotion problems in children with ASD and their peers. *Research in Developmental Disabilities,106, 2020, ArtID 103770, 106*. <https://dx.doi.org/10.1016/j.ridd.2020.103770>

Riek, N. T., Susam, B. T., Hudac, C. M., Conner, C. M., Akcakaya, M., Yun, J., . . . Gable, P. A. (2023). Feedback related negativity amplitude is greatest following deceptive feedback in autistic adolescents. *Journal of Autism and Developmental Disorders*, No Pagination Specified. <https://dx.doi.org/10.1007/s10803-023-06038-y>

Rivard, M., Morin, D., Coulombe, P., Morin, M., & Mello, C. (2023). The diagnostic period for autism: Risk and protective factors for family quality of life in early childhood. *Journal of Autism and Developmental Disorders, 53*(10), 3755-3769. <https://dx.doi.org/10.1007/s10803-022-05686-w>

Rixon, L., Hastings, R. P., Kovshoff, H., & Bailey, T. (2021). Sibling adjustment and sibling relationships associated with clusters of needs in children with autism: A novel methodological approach. Journal of Autism and Developmental Disorders, 51(11), 4067-4076. <https://dx.doi.org/10.1007/s10803-020-04854-0>

Rodriguez, G., Drastal, K., & Hartley, S. L. (2021). Cross-lagged model of bullying victimization and mental health problems in children with autism in middle to older childhood. Autism, 25(1), 90-101. <https://dx.doi.org/10.1177/1362361320947513>

Rodriguez, G., Hartley, S. L., & Bolt, D. (2019). Transactional Relations Between Parenting Stress and Child Autism Symptoms and Behavior Problems. Journal of Autism and Developmental Disorders, 49(5), 1887-1898. <http://dx.doi.org/10.1007/s10803-018-3845-x>

Rohacek, A., Baxter, E. L., Sullivan, W. E., Roane, H. S., & Antshel, K. M. (2023). A preliminary evaluation of a brief behavioral parent training for challenging behavior in autism spectrum disorder. *Journal of Autism and Developmental Disorders, 53*(8), 2964-2974. <https://dx.doi.org/10.1007/s10803-022-05493-3>

Rojahn, J., Matson, J. L., Mahan, S., Fodstad, J. C., Knight, C., Sevin, J. A., & Sharp, B. (2009). Cutoffs, norms, and patterns of problem behaviors in children with an ASD on the Baby and Infant Screen for Children with aUtIsm Traits (BISCUIT-Part 3). Research in Autism Spectrum Disorders, 3(4), 989-998. <http://dx.doi.org/10.1016/j.rasd.2009.06.002>

Saez-Suanes, G. P., Alvarez-Couto, M., & d'Orey Roquete, M. (2020). Depressive symptomatology in adults with autism spectrum disorder and intellectual disability: correlates and predictors. International Journal of Developmental Disabilities. <http://dx.doi.org/10.1080/20473869.2020.1721161>

Salomone, E., Kutlu, B., Derbyshire, K., McCloy, C., Hastings, R. P., Howlin, P., & Charman, T. (2014). Emotional and behavioural problems in children and young people with autism spectrum disorder in specialist autism schools. Research in Autism Spectrum Disorders, 8(6), 661-668. <https://dx.doi.org/10.1016/j.rasd.2014.03.004>

Salomone, E., Settanni, M., Ferrara, F., & Salandin, A. (2019). The interplay of communication skills, emotional and behavioural problems and parental psychological distress. Journal of Autism and Developmental Disorders, 49(11), 4365-4374. <https://dx.doi.org/10.1007/s10803-019-04142-6>

Samadi, S. A., & Rashid, H. M. (2023). Impacts of caregiving for individuals with autism in low-resource settings, a report from the kurdistan region of iraq. *Journal of Autism and Developmental Disorders*, No Pagination Specified. <https://dx.doi.org/10.1007/s10803-023-06165-6>

Schiltz, H. K., & Magnus, B. E. (2020). Gender-based differential item functioning on the child behavior checklist in youth on the autism spectrum: A brief report. Research in Autism Spectrum Disorders, 79 (no pagination)(101669). <https://dx.doi.org/10.1016/j.rasd.2020.101669>

Schiltz, H. K., McVey, A. J., Magnus, B., Dolan, B. K., Willar, K. S., Pleiss, S., Karst, J., Carson, A. M., Caiozzo, C., Vogt, E., & Hecke, A. V. (2018). Examining the links between challenging behaviors in youth with ASD and parental stress, mental health, and involvement: Applying an adaptation of the family stress model to families of youth with ASD. Journal of Autism and Developmental Disorders, 48(4), 1169-1180. <https://dx.doi.org/10.1007/s10803-017-3446-0>

Skwerer, D., Joseph, R. M., Eggleston, B., Meyer, S. R., & Tager-Flusberg, H. (2019). Prevalence and Correlates of Psychiatric Symptoms in Minimally Verbal Children and Adolescents With ASD. *Frontiers in psychiatry Frontiers Research Foundation, 10*, 43. <https://dx.doi.org/10.3389/fpsyt.2019.00043>

Stadnick, N., Chlebowski, C., Baker-Ericzen, M., Dyson, M., Garland, A., & Brookman-Frazee, L. (2017). Psychiatric comorbidity in autism spectrum disorder: Correspondence between mental health clinician report and structured parent interview. Autism, 21(7), 841-851. <https://dx.doi.org/10.1177/1362361316654083>

Sterling, L., Renno, P., Storch, E. A., Ehrenreich-May, J., Lewin, A. B., Arnold, E., Lin, E., & Wood, J. (2015). Validity of the Revised Children's Anxiety and Depression Scale for youth with autism spectrum disorders. Autism, 19(1), 113-117. <https://dx.doi.org/10.1177/1362361313510066>

Stratis, E. A., & Lecavalier, L. (2017). Predictors of Parent-Teacher Agreement in Youth with Autism Spectrum Disorder and Their Typically Developing Siblings. Journal of Autism & Developmental Disorders, 47(8), 2575-2585. <https://dx.doi.org/10.1007/s10803-017-3173-6>

Tarver, J., Vitoratou, S., Mastroianni, M., Heaney, N., Bennett, E., Gibbons, F., Fiori, F., Absoud, M., Ramasubramanian, L., Simonoff, E., & Santosh, P. (2021). Development and Psychometric Properties of a New Questionnaire to Assess Mental Health and Concerning Behaviors in Children and Young People with Autism Spectrum Disorder (ASD): The Assessment of Concerning Behavior (ACB) Scale. Journal of Autism & Developmental Disorders, 51(8), 2812-2828. <https://dx.doi.org/10.1007/s10803-020-04748-1>

Taylor, B. J., Reynolds, C. F., III, & Siegel, M. (2021). Insomnia subtypes and clinical impairment in hospitalized children with autism spectrum disorder. *Autism, 25*(3), 656-666. <https://dx.doi.org/10.1177/1362361320967524>

Taylor, J. M., Volker, M. A., Rispoli, K. M., Rodgers, J. D., Thomeer, M. L., Lopata, C., Chow, S. Y., Toomey, J. A., & Smerbeck, A. (2020). Depression, Anxiety, and Hyperactivity in Youth with HFASD: A Replication and Extension of Symptom Level Differences in Self-Report Versus Parent Report. Journal of Autism and Developmental Disorders, 50(7), 2424-2438. <http://dx.doi.org/10.1007/s10803-018-3779-3>

Thorson, R. T., & Matson, J. L. (2012). Cutoff scores for the Autism Spectrum Disorder - Comorbid for Children (ASD-CC). Research in Autism Spectrum Disorders, 6(1), 556-559. <http://dx.doi.org/10.1016/j.rasd.2011.07.016>

Totsika, V., Hastings, R. P., Emerson, E., Lancaster, G. A., Berridge, D. M., & Vagenas, D. (2013). Is there a bidirectional relationship between maternal well-being and child behavior problems in autism spectrum disorders? Longitudinal analysis of a population-defined sample of young children. Autism Research, 6(3), 201-211. <https://dx.doi.org/10.1002/aur.1279>

Tureck, K., Matson, J. L., May, A., Whiting, S. E., & Davis, T. E., III. (2014). Comorbid symptoms in children with anxiety disorders compared to children with autism spectrum disorders. Journal of Developmental and Physical Disabilities, 26(1), 23-33. <https://dx.doi.org/10.1007/s10882-013-9342-4>

Uljarevic, M., Richdale, A. L., McConachie, H., Hedley, D., Cai, R. Y., Merrick, H., Parr, J. R., & Le Couteur, A. (2018). The Hospital Anxiety and Depression scale: Factor structure and psychometric properties in older adolescents and young adults with autism spectrum disorder. Autism research : Official Journal of the International Society for Autism Research, 11(2), 258-269. <https://dx.doi.org/10.1002/aur.1872>

Ung, D., Arnold, E. B., De Nadai, A. S., Lewin, A. B., Phares, V., Murphy, T. K., & Storch, E. A. (2014). Inter-rater reliability of the Anxiety Disorders Interview Schedule for DSM-IV in high-functioning youth with autism spectrum disorder. Journal of Developmental and Physical Disabilities, 26(1), 53-65. <https://dx.doi.org/10.1007/s10882-013-9343-3>

Ung, D., Boone, D. M., McBride, N., Howie, F., Scalli, L., & Storch, E. A. (2017). Parent and teacher agreement of behavior problems in youth diagnosed with and without autism spectrum disorders. Journal of Child and Family Studies, 26(2), 370-380. <https://dx.doi.org/10.1007/s10826-016-0566-7>

Wang, G., Liu, Z., Xu, G., Jiang, F., Lu, N., Baylor, A., & Owens, J. (2016). Sleep Disturbances and Associated Factors in Chinese Children with Autism Spectrum Disorder: A Retrospective and Cross-Sectional Study. Child Psychiatry & Human Development, 47(2), 248-258. <https://dx.doi.org/10.1007/s10578-015-0561-z>

Wei, Q., Machalicek, W., & Zhu, J. (2023). Treatment acceptability for interventions addressing challenging behavior among Chinese caregivers of children with autism spectrum disorder. *Journal of Autism and Developmental Disorders, 53*(4), 1483-1494. <https://dx.doi.org/10.1007/s10803-021-05196-1>

Weiss, J. A., Cappadocia, M., MacMullin, J. A., Viecili, M., & Lunsky, Y. (2012). The impact of child problem behaviors of children with ASD on parent mental health: The mediating role of acceptance and empowerment. Autism, 16(3), 261-274. <https://dx.doi.org/10.1177/1362361311422708>

Werkman, M., Brouwer, S., Dijkxhoorn, Y., van Berckelaer-Onnes, I., Reijneveld, S., Landsman, J., & Begeer, S. (2020). The moderating effect of cognitive abilities on the association between sensory processing and emotional and behavioural problems and social participation in autistic individuals. Research in Autism Spectrum Disorders Vol 78 2020, ArtID 101663, 78. <https://dx.doi.org/10.1016/j.rasd.2020.101663>

Witwer, A. N., Lecavalier, L., & Norris, M. (2012). Reliability and validity of the children's interview for psychiatric syndromes--parent version in autism spectrum disorders. Journal of Autism & Developmental Disorders, 42(9), 1949-1958. <https://dx.doi.org/10.1007/s10803-012-1442-y>

Xu, Y., Neece, C. L., & Parker, K. H. (2014). Parental depression and child behavior problems: A pilot study examining pathways of influence. Journal of Mental Health Research in Intellectual Disabilities, 7(2), 126-142. <https://dx.doi.org/10.1080/19315864.2013.787479>

Yan, T., Hou, Y., Deng, M., & Han, F. (2023). The effect of family capital on psychological adjustment of chinese children with autism spectrum disorder in the transition from kindergarten to primary school. *International Journal of Developmental Disabilities*, No Pagination Specified. <https://dx.doi.org/10.1080/20473869.2023.2233753>

Yang, C.-J., Jin, J.-Y., & Sun, Y.-W. (2023). The impact of children behavior on depressive symptoms among parents of children with ASD: The mediating role of mindfulness and perceived social support. *Journal of Mental Health Research in Intellectual Disabilities, 16*(2), 92-113. <https://dx.doi.org/10.1080/19315864.2022.2070809>

Yang, Y. J., & Chung, K.-M. (2023). Pilot randomized control trial of an app-based CBT program for reducing anxiety in individuals with ASD without intellectual disability. *Journal of Autism and Developmental Disorders, 53*(4), 1331-1346. <https://dx.doi.org/10.1007/s10803-022-05617-9>
