# Supplementary material for: Applicability and Psychometric Properties of General Mental Health Assessment Tools in Autistic People: A Systematic Review
Source: J Autism Dev Disord. 2024 Apr 13;55(5):1713–26. doi: 10.1007/s10803-024-06324-3 (PMC12021962; doi:10.1007/s10803-024-06324-3)
Supplement: Supplementary file 6 — Supplementary file6 (DOCX 47 KB) [file 10803_2024_6324_MOESM6_ESM.docx]

| **Appendix F**  *Risk of Bias Analysis for Included Papers* | | | | | | | | | | |
| --- | --- | --- | --- | --- | --- | --- | --- | --- | --- | --- |
|  | | | | | | | | | | |
| Paper | Sample selection bias | | | Methodological bias | | | | Results bias | | Total bias score |
|  | 1 | 2 | 3 | 4 | 5 | 6 | 7 | 8 | 9 |  |
| Adams et al. (2019) | 1 | 0 | 0 | 0 | 1 | 0 | 1 | 0 | 1 | 4 |
| Alallwi et al. (2022) | 1 | 0 | 1 | 0 | 1 | 0 | 1 | 0 | 1 | 5 |
| Bacherini et al. (2021) | 1 | 0 | 0 | 0 | 0 | 0 | 0 | 0 | 0 | 1 |
| Baker et al. (2015) | 1 | 0 | 1 | 0 | 1 | 1 | 1 | 0 | 0 | 5 |
| Bakken et al. (2010) | 0 | 0 | 0 | 0 | 0 | 0 | 0 | 0 | 1 | 1 |
| Bakken et al. (2023) | 1 | 0 | 0 | 0 | 1 | 0 | 0 | 0 | 0 | 2 |
| Bangerter et al. (2017) | 1 | 1 | 1 | 0 | 1 | 1 | 1 | 0 | 0 | 6 |
| Beer et al. (2013) | 1 | 0 | 1 | 0 | 1 | 0 | 0 | 0 | 0 | 3 |
| Bekhet (2016) | 1 | 0 | 1 | 0 | 1 | 0 | 0 | 0 | 1 | 4 |
| Benson (2015) | 1 | 0 | 0 | 0 | 0 | 0 | 0 | 0 | 0 | 1 |
| Bitsika & Sharpley (2016) | 1 | 0 | 0 | 0 | 1 | 0 | 1 | 0 | 0 | 3 |
| Bitsika et al. (2016) | 1 | 0 | 0 | 0 | 1 | 0 | 1 | 0 | 0 | 3 |
| Bitsika & Sharpley (2017) | 1 | 0 | 0 | 0 | 1 | 0 | 1 | 0 | 0 | 3 |
| Brinkley et al. (2007) | 1 | 0 | 0 | 0 | 1 | 0 | 1 | 0 | 0 | 3 |
| Brookman-Frazee et al. (2018) | 1 | 0 | 0 | 0 | 0 | 0 | 0 | 0 | 0 | 1 |
| Buck et al. (2014) | 0 | 0 | 0 | 0 | 0 | 0 | 0 | 0 | 1 | 1 |
| Burton et al. (2020) | 1 | 0 | 0 | 0 | 1 | 0 | 1 | 0 | 1 | 4 |
| Byrne et al. (2023) | 1 | 0 | 0 | 0 | 0 | 0 | 0 | 0 | 1 | 2 |
| Chan et al. (2022) | 1 | 0 | 0 | 0 | 0 | 0 | 1 | 0 | 0 | 2 |
| Chandler et al. (2016) | 0 | 0 | 0 | 0 | 0 | 0 | 0 | 0 | 0 | 0 |
| Charlton et al. (2020) | 1 | 0 | 0 | 0 | 1 | 0 | 1 | 0 | 1 | 4 |
| Cheng et al. (2021) | 1 | 0 | 0 | 0 | 1 | 0 | 1 | 0 | 1 | 4 |
| Cheng et al. (2022) | 1 | 0 | 0 | 0 | 1 | 0 | 1 | 0 | 1 | 4 |
| Chua et al. (2023) | 0 | 0 | 1 | 0 | 0 | 0 | 0 | 0 | 0 | 1 |
| Chung & Jung (2017) | 1 | 0 | 0 | 0 | 0 | 0 | 1 | 0 | 0 | 2 |
| Clauser et al. (2021) | 1 | 0 | 0 | 0 | 1 | 1 | 1 | 0 | 0 | 4 |
| Davis & Carter (2008) | 1 | 0 | 0 | 0 | 1 | 1 | 1 | 0 | 1 | 5 |
| Day et al. (2024) | 0 | 0 | 1 | 0 | 1 | 0 | 1 | 0 | 0 | 3 |
| De Clercq et al. (2019) | 1 | 0 | 0 | 0 | 1 | 0 | 1 | 0 | 1 | 4 |
| De Clercq et al. (2021) | 1 | 0 | 0 | 0 | 1 | 0 | 1 | 0 | 1 | 4 |
| De Pauw et al. (2011) | 1 | 0 | 0 | 0 | 0 | 0 | 1 | 0 | 1 | 3 |
| Deniz & Toseeb (2023) | 0 | 0 | 1 | 0 | 1 | 0 | 0 | 0 | 0 | 2 |
| Dieleman et al. (2017) | 1 | 0 | 0 | 0 | 0 | 0 | 0 | 0 | 1 | 2 |
| Dieleman et al. (2018) | 1 | 0 | 1 | 0 | 0 | 1 | 0 | 0 | 1 | 4 |
| Dovgan et al. (2019) | 1 | 0 | 1 | 0 | 1 | 1 | 1 | 0 | 1 | 6 |
| Farmer et al. (2016) | 1 | 0 | 1 | 0 | 1 | 0 | 1 | 0 | 1 | 5 |
| Findon et al. (2016) | 1 | 0 | 0 | 0 | 1 | 0 | 1 | 0 | 0 | 3 |
| Firth & Dryer (2013) | 1 | 0 | 1 | 0 | 1 | 0 | 1 | 0 | 0 | 4 |
| Fok & Bal (2019) | 1 | 1 | 1 | 0 | 1 | 1 | 1 | 0 | 0 | 6 |
| Fong et al. (2020) | 1 | 0 | 0 | 0 | 1 | 0 | 1 | 0 | 1 | 4 |
| Fong et al. (2021) | 1 | 0 | 0 | 0 | 1 | 0 | 1 | 0 | 1 | 4 |
| Gardiner & Iarocci (2015) | 1 | 0 | 0 | 0 | 1 | 0 | 1 | 0 | 1 | 4 |
| Gjevik et al. (2011) | 1 | 0 | 0 | 0 | 1 | 0 | 1 | 0 | 1 | 4 |
| Gjevik et al. (2015) | 1 | 0 | 0 | 0 | 0 | 0 | 1 | 0 | 1 | 3 |
| Graziosi & Perry (2023) | 1 | 0 | 0 | 0 | 1 | 0 | 0 | 0 | 0 | 2 |
| Hastings et al. (2005) | 1 | 1 | 0 | 0 | 0 | 0 | 1 | 0 | 0 | 4 |
| Hastings et al. (2020) | 1 | 0 | 0 | 0 | 1 | 1 | 1 | 0 | 0 | 4 |
| Helverschou et al. (2009) | 1 | 0 | 0 | 0 | 0 | 0 | 1 | 0 | 0 | 2 |
| Helverschou et al. (2021) | 1 | 0 | 0 | 0 | 0 | 0 | 0 | 0 | 0 | 1 |
| Hepburn et al. (2014) | 1 | 0 | 0 | 0 | 0 | 0 | 1 | 0 | 0 | 2 |
| Higgins et al. (2022) | 1 | 0 | 0 | 0 | 1 | 0 | 0 | 0 | 0 | 2 |
| Horovitz & Matson (2013) | 1 | 0 | 1 | 0 | 0 | 0 | 1 | 0 | 1 | 4 |
| Horovitz & Matson (2015) | 1 | 0 | 0 | 0 | 0 | 0 | 1 | 0 | 1 | 3 |
| Hurtig et al. (2009) | 1 | 0 | 0 | 0 | 0 | 0 | 1 | 0 | 1 | 3 |
| Jellett et al. (2015) | 1 | 0 | 1 | 0 | 0 | 0 | 1 | 0 | 0 | 3 |
| Jepsen et al. (2012) | 1 | 0 | 0 | 0 | 0 | 0 | 1 | 0 | 1 | 3 |
| Jeter et al. (2017) | 1 | 0 | 0 | 0 | 1 | 0 | 1 | 0 | 0 | 3 |
| Jones et al. (2014) | 1 | 0 | 1 | 0 | 1 | 0 | 1 | 0 | 0 | 4 |
| Kaat et al. (2013) | 1 | 0 | 0 | 0 | 0 | 1 | 1 | 0 | 0 | 3 |
| Kaat & Lecavalier (2015) | 1 | 0 | 0 | 0 | 1 | 1 | 1 | 0 | 0 | 4 |
| Kaat et al. (2014) | 1 | 0 | 0 | 0 | 1 | 0 | 0 | 0 | 1 | 3 |
| Kalb et al. (2018) | 1 | 0 | 0 | 0 | 0 | 1 | 1 | 0 | 0 | 3 |
| Kang et al. (2020) | 1 | 0 | 0 | 0 | 1 | 0 | 0 | 0 | 0 | 2 |
| Kanne et al. (2009) | 1 | 0 | 0 | 0 | 1 | 0 | 1 | 0 | 1 | 4 |
| Kat et al. (2020) | 1 | 1 | 1 | 0 | 1 | 0 | 0 | 0 | 0 | 4 |
| Keefer et al. (2020) | 1 | 0 | 0 | 0 | 1 | 0 | 0 | 0 | 0 | 2 |
| Khalfe et al. (2023) | 1 | 0 | 0 | 0 | 1 | 1 | 0 | 0 | 0 | 3 |
| Khor et al. (2014) | 1 | 0 | 0 | 0 | 0 | 1 | 1 | 0 | 0 | 3 |
| Kildahl & Helverschou (2023) | 1 | 0 | 0 | 0 | 1 | 0 | 0 | 0 | 0 | 2 |
| Kildahl et al. (2023) | 1 | 0 | 0 | 0 | 1 | 0 | 0 | 0 | 0 | 2 |
| Kim et al. (2018) | 1 | 0 | 1 | 0 | 1 | 0 | 0 | 0 | 0 | 3 |
| Kirst et al. (2022) | 1 | 0 | 0 | 0 | 1 | 0 | 1 | 0 | 1 | 4 |
| Koller et al. (2022) | 1 | 0 | 1 | 0 | 1 | 0 | 0 | 0 | 0 | 3 |
| LaBuissonniere Ariza et al. (2022) | 0 | 0 | 0 | 0 | 0 | 0 | 1 | 0 | 0 | 1 |
| Lane et al. (2013) | 1 | 0 | 0 | 0 | 1 | 0 | 0 | 0 | 0 | 2 |
| Leader, Moore et al. (2022) | 1 | 1 | 0 | 0 | 1 | 1 | 1 | 0 | 1 | 6 |
| Leader, Dooley et al. (2021) | 1 | 0 | 1 | 0 | 1 | 1 | 1 | 0 | 0 | 5 |
| Leader, Flynn et al. (2021) | 1 | 1 | 1 | 0 | 1 | 1 | 1 | 0 | 0 | 6 |
| Leader et al. (2018) | 1 | 0 | 1 | 0 | 1 | 1 | 1 | 0 | 0 | 5 |
| Lecavalier et al. (2004) | 1 | 0 | 0 | 0 | 1 | 0 | 0 | 0 | 1 | 3 |
| Lecavalier et al. (2006) | 1 | 0 | 1 | 0 | 1 | 0 | 0 | 0 | 0 | 3 |
| Lecavalier et al. (2009) | 1 | 0 | 0 | 0 | 1 | 0 | 1 | 0 | 0 | 3 |
| Lecavalier et al. (2011) | 1 | 0 | 0 | 0 | 1 | 0 | 1 | 0 | 0 | 3 |
| Leyfer et al. (2006) | 1 | 0 | 0 | 0 | 1 | 0 | 1 | 1 | 0 | 4 |
| Lovell & Wetherell (2016) | 1 | 0 | 1 | 0 | 0 | 1 | 1 | 0 | 0 | 4 |
| Lovell & Wetherell (2020) | 1 | 0 | 1 | 0 | 0 | 1 | 1 | 0 | 0 | 4 |
| LoVullo & Matson (2009) | 1 | 1 | 1 | 1 | 1 | 0 | 1 | 0 | 1 | 7 |
| Lu, Chen et al. (2021) | 1 | 0 | 1 | 0 | 0 | 0 | 1 | 0 | 1 | 4 |
| Lu, Wang et al. (2021) | 1 | 0 | 1 | 0 | 0 | 0 | 1 | 0 | 1 | 4 |
| Magiati et al. (2016) | 1 | 0 | 1 | 0 | 0 | 0 | 1 | 0 | 0 | 3 |
| Magyar & Pandolfi (2017) | 1 | 0 | 0 | 0 | 0 | 0 | 1 | 0 | 1 | 3 |
| Mahan & Matson (2011) | 1 | 0 | 1 | 0 | 1 | 0 | 1 | 0 | 0 | 4 |
| Manning et al. (2011) | 1 | 0 | 0 | 0 | 0 | 0 | 1 | 0 | 0 | 2 |
| Mannion & Leader (2013) | 1 | 0 | 1 | 0 | 1 | 1 | 1 | 0 | 1 | 6 |
| Martinez et al. (2023) | 1 | 0 | 0 | 0 | 1 | 0 | 1 | 0 | 0 | 3 |
| Matson & Boisjoli (2008) | 1 | 0 | 1 | 0 | 1 | 0 | 1 | 0 | 1 | 5 |
| Matson, Boisjoli et al. (2009) | 1 | 0 | 0 | 0 | 1 | 0 | 1 | 0 | 1 | 4 |
| Matson, Fodstad et al. (2009) | 1 | 0 | 0 | 0 | 1 | 0 | 1 | 0 | 1 | 4 |
| Matson, LoVullo et al. (2009) | 1 | 0 | 1 | 0 | 0 | 0 | 1 | 0 | 1 | 4 |
| Matson et al. (2011) | 1 | 1 | 1 | 0 | 1 | 0 | 1 | 0 | 1 | 6 |
| Matson & Wilkins (2008) | 1 | 0 | 0 | 0 | 1 | 0 | 1 | 0 | 1 | 4 |
| Mattila et al. (2010) | 0 | 0 | 0 | 0 | 0 | 0 | 0 | 0 | 1 | 1 |
| Mazefsky et al. (2012) | 1 | 0 | 0 | 0 | 0 | 0 | 1 | 1 | 0 | 3 |
| Mazefsky et al. (2014) | 1 | 0 | 0 | 0 | 0 | 1 | 1 | 0 | 0 | 3 |
| Mazefsky, Day et al. (2018) | 1 | 0 | 0 | 0 | 0 | 0 | 1 | 0 | 0 | 2 |
| Mazefsky, Yu et al. (2018) | 1 | 0 | 0 | 0 | 1 | 0 | 0 | 0 | 0 | 2 |
| McIntyre et al. (2023) | 1 | 0 | 0 | 0 | 0 | 0 | 1 | 0 | 0 | 2 |
| Medeiros et al. (2017) | 1 | 0 | 0 | 0 | 1 | 0 | 1 | 0 | 0 | 3 |
| Mello et al. (2022) | 1 | 0 | 0 | 0 | 0 | 1 | 0 | 0 | 0 | 2 |
| Mihaila & Harley (2018) | 1 | 0 | 0 | 0 | 1 | 0 | 1 | 0 | 1 | 4 |
| Milosavljevic et al. (2016) | 0 | 0 | 0 | 0 | 0 | 0 | 0 | 0 | 1 | 1 |
| Miranda et al. (2019) | 1 | 0 | 0 | 0 | 1 | 0 | 1 | 0 | 0 | 4 |
| Mohammadi et al. (2023) | 1 | 0 | 1 | 0 | 0 | 0 | 1 | 0 | 0 | 3 |
| Mosner et al. (2019) | 1 | 0 | 0 | 0 | 1 | 0 | 1 | 0 | 1 | 4 |
| Nadeau et al. (2015) | 1 | 1 | 0 | 0 | 0 | 1 | 1 | 0 | 1 | 5 |
| Norris et al. (2019) | 1 | 0 | 0 | 0 | 1 | 0 | 1 | 0 | 0 | 3 |
| Palmer et al. (2021) | 1 | 0 | 0 | 0 | 0 | 0 | 1 | 0 | 1 | 3 |
| Palmer et al. (2023) | 1 | 0 | 0 | 0 | 1 | 0 | 1 | 0 | 0 | 3 |
| Pandolfi et al. (2009) | 1 | 0 | 0 | 0 | 1 | 0 | 0 | 0 | 0 | 2 |
| Pandolfi et al. (2012) | 1 | 1 | 0 | 0 | 1 | 0 | 1 | 0 | 0 | 4 |
| Pandolfi et al. (2014) | 1 | 1 | 0 | 0 | 1 | 0 | 1 | 0 | 0 | 4 |
| Park et al. (2020) | 1 | 0 | 0 | 0 | 0 | 0 | 0 | 0 | 0 | 1 |
| Pearson et al. (2012) | 1 | 0 | 0 | 0 | 1 | 0 | 1 | 0 | 0 | 3 |
| Piro-Gambetti et al. (2023) | 1 | 0 | 0 | 0 | 1 | 0 | 0 | 0 | 0 | 2 |
| Pisula et al. (2017) | 1 | 0 | 0 | 0 | 0 | 1 | 1 | 0 | 0 | 3 |
| Plak et al. (2023) | 1 | 0 | 1 | 0 | 0 | 0 | 0 | 0 | 0 | 2 |
| Pozo & Sarria (2014) | 1 | 0 | 0 | 0 | 1 | 0 | 1 | 0 | 1 | 4 |
| Pruitt et al. (2018) | 1 | 0 | 1 | 0 | 1 | 1 | 1 | 0 | 1 | 6 |
| Reyes et al. (2020) | 1 | 0 | 0 | 0 | 0 | 1 | 1 | 0 | 0 | 3 |
| Riek et al. (2023) | 1 | 0 | 0 | 0 | 1 | 0 | 1 | 0 | 0 | 3 |
| Rivard et al. 2023 | 1 | 1 | 0 | 0 | 1 | 0 | 0 | 0 | 0 | 3 |
| Rixon et al. (2021) | 1 | 1 | 0 | 0 | 0 | 1 | 1 | 0 | 0 | 4 |
| Rohacek et al. (2023) | 0 | 1 | 0 | 0 | 0 | 0 | 1 | 0 | 0 | 2 |
| Rodriguez et al. (2019) | 1 | 0 | 0 | 0 | 0 | 0 | 1 | 0 | 0 | 2 |
| Rodriquez et al. (2021) | 1 | 0 | 0 | 0 | 0 | 1 | 1 | 0 | 0 | 3 |
| Rojahn et al. (2009) | 1 | 0 | 0 | 0 | 1 | 0 | 1 | 0 | 1 | 4 |
| Saez-Suanes et al. (2020) | 1 | 0 | 0 | 0 | 1 | 0 | 1 | 0 | 0 | 3 |
| Salomone et al. (2014) | 1 | 0 | 1 | 0 | 1 | 0 | 1 | 0 | 0 | 4 |
| Salomone et al. (2019) | 1 | 0 | 0 | 0 | 0 | 0 | 1 | 0 | 1 | 3 |
| Samadi & Rashid (2023) | 1 | 0 | 0 | 0 | 0 | 0 | 1 | 1 | 1 | 4 |
| Schiltz & Magnus (2020) | 1 | 0 | 0 | 0 | 0 | 1 | 1 | 0 | 0 | 3 |
| Schiltz et al. (2018) | 1 | 1 | 0 | 0 | 0 | 1 | 1 | 0 | 1 | 5 |
| Stadnick et al. (2017) | 1 | 0 | 0 | 0 | 0 | 0 | 1 | 0 | 0 | 2 |
| Skwerer et al. (2019) | 1 | 0 | 0 | 0 | 1 | 0 | 1 | 0 | 0 | 3 |
| Sterling et al. (2015) | 1 | 0 | 0 | 0 | 1 | 0 | 1 | 0 | 1 | 4 |
| Stratis & Lecavalier (2017) | 1 | 0 | 0 | 0 | 1 | 1 | 0 | 0 | 0 | 3 |
| Tarver et al. (2021) | 1 | 0 | 0 | 0 | 0 | 1 | 0 | 0 | 1 | 3 |
| Taylor et al. (2020) | 1 | 1 | 0 | 0 | 1 | 1 | 1 | 0 | 0 | 5 |
| Taylor et al. (2021) | 1 | 1 | 0 | 0 | 0 | 1 | 1 | 0 | 0 | 4 |
| Thorson & Matson (2012) | 1 | 1 | 0 | 0 | 1 | 0 | 1 | 0 | 1 | 5 |
| Totsika et al. (2013) | 0 | 0 | 1 | 0 | 1 | 0 | 1 | 0 | 1 | 4 |
| Tureck et al. (2014) | 1 | 0 | 0 | 0 | 1 | 1 | 1 | 0 | 1 | 5 |
| Uljarevic et al. (2018) | 1 | 1 | 1 | 0 | 1 | 0 | 1 | 0 | 1 | 6 |
| Ung et al. (2014) | 1 | 0 | 0 | 0 | 0 | 1 | 1 | 0 | 0 | 3 |
| Ung et al. (2017) | 1 | 0 | 0 | 0 | 1 | 1 | 1 | 0 | 0 | 4 |
| Wang et al. (2016) | 1 | 0 | 0 | 0 | 1 | 0 | 0 | 0 | 0 | 2 |
| Wei et al. (2023) | 1 | 1 | 1 | 0 | 0 | 0 | 1 | 0 | 1 | 5 |
| Weiss et al. (2012) | 1 | 0 | 0 | 0 | 1 | 0 | 0 | 0 | 0 | 2 |
| Werkman et al. (2020) | 1 | 0 | 0 | 0 | 0 | 1 | 0 | 0 | 1 | 3 |
| Witwer et al. (2012) | 1 | 0 | 0 | 0 | 1 | 1 | 1 | 0 | 0 | 4 |
| Xu et al. (2014) | 1 | 0 | 0 | 0 | 0 | 0 | 1 | 0 | 0 | 2 |
| Yan et al. (2023) | 1 | 0 | 1 | 0 | 1 | 0 | 1 | 0 | 1 | 5 |
| Yang et al. (2023) | 1 | 0 | 1 | 0 | 0 | 0 | 1 | 0 | 0 | 3 |
| Yang, & Chung (2023) | 1 | 0 | 0 | 0 | 0 | 0 | 1 | 0 | 0 | 2 |

*Note.* Risk of bias was scores “0” (low risk) or “1” (high risk) on nine items based on criteria developed by Villalobos et al. (2022; see also Whiting et al., 2003). For the total bias score lower scores indicate a lower risk of bias. The nine items addressed the following: Sample: 1 = representativeness, 2 = selection criteria, 3 = verification of diagnosis; Methods: 4 = level of detail, 5 = withdrawals, 6 = where sample obtained, 7 = when sample obtained; Result; 8 =statistical analysis, 9 = study limitations
