# Supplementary material for: Applicability and Psychometric Properties of General Mental Health Assessment Tools in Autistic People: A Systematic Review
Source: J Autism Dev Disord. 2024 Apr 13;55(5):1713–26. doi: 10.1007/s10803-024-06324-3 (PMC12021962; doi:10.1007/s10803-024-06324-3)
Supplement: Supplementary file 7 — Supplementary file7 (DOCX 150 KB) [file 10803_2024_6324_MOESM7_ESM.docx]

**Appendix G**

*Overview of Studies: Study Characteristics and Psychometric Data*

| Measure | Author, year | Country | Sample description | IQ/Language | *N* | Study design | Rater | Psychometric properties |
| --- | --- | --- | --- | --- | --- | --- | --- | --- |
| *ABC/*  Community |  |  |  |  |  |  |  |  |
| version | Bakken et al. (2023): Kildahl & Helverschou (2023); Kildahl; Ludvigsen et al. (2023) | Norway | The Autism, ID and Mental Illness study. 69% male. Age 14–68 years. 3 groups: ASD/ID/SchizophreniaID/other MH disorders; ASD/ID/non-MH disorder | All levels of ID (mild–profound).  FSIQ/verbal function NR | 183 | Cross-sectional/ prospective | Caregiver/clinician | Internal consistency; Criterion validity (group differences) |
|  | Bitsika & Sharpley (2016; 2017) | Australia | Support groups/autism services. 100% boys. Age: 6–18 years. | FSIQ >70.  Verbal function: NR | 150 | Cross-sectional | Parent | Internal consistency |
|  |  |  |  |  |  |  |  |  |
|  | Brinkley et al. (2007) | US | Genetic study. 85% boys. Age: 3–21 years. | VABS adaptive composite: *M* = 61 (*SD*=17). Intact language = 73%. Impaired language = 24% | 275 | Cross-sectional | Parent | Factor structure (EFA/CFA) |
|  | Chua et al. (2023) | Malaysia | Clinical sample. 84% boys. Age 4–18 years. | ID =4.3%  FSIQ/verbal function NR | 230 | Cross-sectional | Parent | Internal consistency |
|  |  |  |  |  |  |  |  |  |
|  | Fok & Bal (2019) | US | Simons Complex Collection. 87% boys. Age 6–18 years. | Non-verbal IQ <70 = 25%; Verbally fluent = 69%; Phrase speech = 16%; Minimally verbal = 15% | 1937 | Cross-sectional; multi-site | Parent | Convergent validity (CBCL) |
|  | Graziosi & Perry (2023) | Canada | Autism Inpatient Collection. 79% boys.  Age 4–20 years. | ID = 46.8%  Non-verbal IQ *M* =74.57 (*SD*=29.34) | 457 | Cross-sectional data, multi-site | Parent | Internal consistency |
|  |  |  |  | VABS_II Communication *M* =58.15 (SD=17.57) |  |  |  |  |
|  | Kaat et al. (2014) | US/Canada | Clinical. 83% boys. Age 2–18 years. | FSIQ < 70 = 47%  Verbal function: NR | 1893 | Cross-sectional; multi-site | Parent | Internal consistency; Factor structure (EFA/CFA); Convergent validity (CBCL); Normative data |
|  |  |  |  |  |  |  |  |  |
|  | Kat et al. (2020)  Koller et al. (2022)  Mohammadi et al. (2023) | China  Israel  Iran | Outpatient/special education. 83% male. Age 1.5–33 years.  Community study. 84% boys. Age 2–9 years.  Special school. 87% boys. Age 6–15 years | NR  FSIQ NR. Receptive language ≥ 18 months  NR | 799/  84/  44  90  303 | Cross-sectional/  Cross-sectional  Cross-sectional | Caregiver  Parent  Parent | Internal consistency; Test-retest; Inter-rater; Factor structure (CFA); Convergent validity (CBCL)  Internal consistency  Internal consistency |
|  |  |  |  |  |  |  |  |  |
|  | Norris et al. (2019) | US | Clinical. 82% boys. Age 2–14 years. | NR | 470 | Cross-sectional multi-site | Parent | Internal consistency; Factor structure (CFA); Normative data |
|  | Rohacek et al. (2023) | US | Clinical pilot trial. 86% boys. Age 5–9 years. | NR | 38 | Cross-sectional data | Parent | Internal consistency |
|  | Samadi & Rashid (2023) | Iraq  Kurdish | Convenience sample.75% boys. Age 3–19 years. | FSIQ NR  Non-verbal = 55% | 118 | Cross-sectional | Parent | Internal consistency |
|  | Taylor et al. (2021) | US | Clinical. 81% boys.  Age *M* =12.8 years (*SD*=3.4 years) | Nonverbal IQ *M* =74.9 (*SD*=28.7).  VABS Communication *M* =61.8 (18.1) | 427 | Cross-sectional data | Parent | Internal consistency |
|  | Yang & Chung (2023) | Korea | Clinical RCT trial CBT. 90% boys. Age 15–35 years | FSIQ ≥80 | 30 | Cross-sectional data | Caregiver | Internal consistency |
|  |  |  |  |  |  |  |  |  |
|  |  |  |  |  |  |  |  |  |
|  |  |  |  |  |  |  |  |  |
|  |  |  |  |  |  |  |  |  |
| *ABI*/full form | Bangerter et al. (2017) | US | New measure. Parents of ASD children. Expert practitioners. 78% boys. Age 13–21 years. | FSIQ = NR. Speaks in full sentences = 45%; Uses single words = 14%; uses signs/pictures = 5%; no language = 5% | 353/  43 | Cross-sectional | Parent;  Health care | Internal consistency; Test-retest; Content validity; Factor structure (CFA); Convergent validity (CASI anxiety) |
| *ACB*/ parent version | Mohammadi et al. (2023) | Iran | See under ABC above.  Identical sample. |  |  |  |  | Internal consistency; Factor structure (CFA); Convergent validity (ABC) |
|  | Palmer et al. (2023) | UK | Parent intervention. 85% boys. Age 4–8 years. | FSIQ NR.  47% minimally verbal. Fluent speech =53% | 83 | Cross-sectional | Parent/ teacher | Internal consistency;  Inter-rater |
|  | Tarver et al. (2021) | UK | New measure. Clinic/special needs schools. >75% boys. Age 5–29 years. | Developmental quotient: *M* = 67.3 (*SD*=27.2). Verbal function: NR | 255/ 121 | Cross-sectional | Parent/ caregiver | Internal consistency; Test-retest; Content validity; Factor structure (EFA/CFA); Convergent validity (ASEBA; ABC; Modified Overt Aggression Scale) |
| *ACI-PL*  Present  Lifetime | Leyfer et al. (2006) | US | New measure. Community. 94% boys. Age 5–17 years. | Salt Lake City IQ>65; Boston FSIQ: *M* = 82.55 (*SD*= 23.42)  Verbal function: NR. | 109 | Pilot: Cross-sectional multi-site | Parent | Inter-rater; Content validity;  Convergent validity (ADI-R compulsions); Criterion validity |
|  |  |  |  |  |  |  |  |  |
| Lifetime diagnoses | Mazefsky et al. (2012) | US | Clinical sample.80% boys. Age 10–17 years. | FSIQ *M* =105 (*SD*=17).  Verbal function: NR | 35 | Cross-sectional; retrospective | Parent | Criterion validity (community diagnosis) |
| *ADIS-IV* | Byrne et al. (2023) | Ireland | Clinical CBT trial. 80% boys. Age 5–11 years | FSIQ < 70 =4.7% | 21 | Cross-sectional data; Follow-up | Parent | Inter-rater |
|  | Ung et al. (2014) | US | Convenience sample. 72% boys. Age 7–16 years. | FSIQ > =70  Verbal function: NR | 70 | Cross-sectional | Children  Parent | Inter-rater |
|  |  |  |  |  |  |  |  |  |
|  |  |  |  |  |  |  |  |  |
| *ASD-CA* | LoVullo & Matson (2009) | US | Clinical. 55% male. Age *M* = 49 years (*SD*= 11.5). Adults | Primarily severe and profound ID.  Verbal abilities =25% | 120/  42 | Cross-sectional multi-site | NR | Criterion validity; Normative data |
|  | Matson & Boisjoli (2008) | US | New measure.  Clinical. 57% male. Age 16–78 years | ID: majority profound. Verbal =29% | 169 | Cross-sectional | Care staff | Internal consistency; Test-retest. Inter-rater; Factor structure (EFA) |
|  | Saez-Suanes et al. (2020) | Spain | Community sample. 71% male. Age 18–46 years. | ID: majority profound and severe. Verbal function: NR | 32 | Cross-sectional | Health care | Internal consistency |
| *ASD-CC* | Chung & Jung (2017)  Leader,  Flynn et al. (2021); Leader, Francis et al; (2018); Leader et al. (2022); Mannion & Leader (2013) | South Korea  Ireland | Clinical. 77% boys. Age 2–16 years.  Community sample. >72% boys. Ages 5–17 /2–18years. | NR  ID = 33% (majority mild–moderate). Vocal= 84% | 330/ 63  127/133/89 | Cross-sectional  Cross-sectional | Parent  Parent | Internal consistency; Test-retest; Factor structure (CFA); Convergent validity (BPI; CBCL)  Internal consistency; Convergent validity (BPI); Criterion validity |
|  | Matson et al. (2008,2009) | US | New measure.  Clinic/community sample. 78% boys. Age 2–17 years. | ID = 12%. Verbal skills = 60% | 177/  36 | Cross-sectional | Parent | Internal consistency; Test-retest; Inter-rater; Factor structure (EFA); Convergent validity (BASC-2) |
|  | Thorson & Matson (2012) | US/Canada | Clinic/community sample. 70% boys. Age 2–17 years. | NR | 639 | Cross-sectional | Parent | Norm data |
|  |  |  |  |  |  |  |  |  |
|  | Tureck et al. (2014) | US | Outpatient clinic. Three groups: ASD, anxiety without ASD, and controls. 79.3% boys. Age: 4–14 years. | NR | 29/  25/  31 | Cross-sectional | Parent | Criterion validity (group-differences) |
|  |  |  |  |  |  |  |  |  |
|  |  |  |  |  |  |  |  |  |
|  |  |  |  |  |  |  |  |  |
| *ASD-PBC*  *ASEBA* | Mahan & Matson (2011) | USA | Clinic/community sample. 77% boys. Age 4–16 years. | FSIQ: NR. Verbal = 85% | 49 | Cross-sectional | Parent | Convergent validity  (BASC-2) |
|  |  |  |  |  |  |  |  |  |
|  |  |  |  |  |  |  |  |  |
| CBCL1.5–5 | Bacherini et al. (2021) | Italy | Clinical. 82% boys. Age 18–37 months. | VABS adaptive composite: *M* =74.42 (*SD*= 10.75)  Verbal function NR | 148 | Cross-sectional | Parent | Internal consistency |
| CBCL 6–18 | Baker & Blacher (2015) | US | School sample. 82% boys. Age *M* =13 years (*SD*=NR) | FSIQ *M* = 90.3 (*SD*= 24.0)  Verbal function NR | 58 | Cross-sectional | Parent | Convergent validity (DISC ODD) |
|  |  |  |  |  |  |  |  |  |
| CBCL 6–18 | Chan et al. (2022) | Hong Kong | ASD support centers/special schools.  83% boys. Age *M* = 11.47 years (SD = 3.97) | ID = 77%  FSIQ/Verbal function NR | 441 | Longitudinal study | Parent | Internal consistency |
|  |  |  |  |  |  |  |  |  |
|  |  |  |  |  |  |  |  |  |
| CBCL6–18/ adult version  CBCL1.5–5  CBCL 6–18 | Charlton et al. (2020)  Cheng et al. (2021; 2022)  Clauser et al. (2021) | US  Taiwan, Chinese version  US | Community/clinic sample. 74% male. Age 16–24 years.  Clinical sample. 89% boys. Age *M* = 32.28 months (*SD*= 9.16 months)  Convenience sample. 80% boys. Age 3–18 years. | FSIQ *M* =103.88 (*SD*=10.68)  Verbal function NR  Mental age *M* = 21.02 months (*SD* = 10.03 months)  Verbal function NR  NR | 27  228/215  70 | Cross-sectional  Cross-sectional  Cross-sectional | Parent  Parent  Parent | Internal consistency  Internal consistency  Factor structure (measurement invariance)  Internal consistency |
| CBCL 4–18 | DeClercq et al. (2019;2021); DePauw (2011)  Dieleman et al. (2017, 2018) | Belgium  Deutch version | Longitudinal community study.  83% boys. Age 5.1–16.2 years. | ID =12%. Cooccurring language developmental disorder = 10% | 141 | Cross-sectional data | Parent | Internal consistency |
|  |  |  |  |  |  |  |  |  |
|  |  |  |  |  |  |  |  |  |
| CBCL1.5–5  6–18 | Dovgan et al. (2019) | US | Two academic medical centers. 83% boys. Age 2-17 years. | ASD without ID  ASD with ID.  FSIQ *M* = 55.09 (*SD*=13.39)/  FSIQ *M* = 51.0 (*SD*=13.08) | 33/75  74/226 | Cross-sectional | Parent | Factor structure (measurement invariance; ASD with/without ID) |
|  |  |  |  |  |  |  |  |  |
|  |  |  |  |  |  |  |  |  |
|  |  |  |  |  |  |  |  |  |
| CBCL6–18 | Fok & Bal (2019) | US | See under ABC above. Identical sample. |  |  |  |  | Convergent validity (ABC) |
| CBCL6–18 | Gjevik et al. (2015) | Norway | Special school. 82% boys. Age 6–17 years. | FSIQ *M* = 65.2 (*SD* = 29.6). VABS functional language ≥ 70 = 19%. | 55/71 | Cross-sectional | Parent | Internal consistency  Criterion validity (KSADS) |
|  |  |  |  |  |  |  |  |  |
| CBCL6–18  YSR  TRF | Hurtig et al. (2009) | Finland | Population-based and patients. 74% boys. Age 11–17 years. | FSIQ >70  Verbal function NR | 43 | Cross-sectional | Self-report  Parent  Teacher | Inter-rater |
|  |  |  |  |  |  |  |  |  |
| CBCL6–18  YSR  TRF | Jepsen et al. (2012) | Australia | Community. 88% boys. Age 12–18 years. | FSIQ *M* = 91.13 (*SD*=18.59).  Verbal function NR | 44  45  36 | Cross-sectional | Self-report  Parent  Teacher | Inter-rater |
|  |  |  |  |  |  |  |  |  |
| CBCL1.5–5  6-18  TRF | Kanne et al. 2009 | US | Part of longitudinal study. 85% boys. Age 3–18 years. | NR | 177 | Cross-sectional data reported | Parent  Teacher | Inter-rater |
|  |  |  |  |  |  |  |  |  |
| CBCL6–18 | Keefer et al. (2020) | US | Clinical. 82% boys. Age 6–18 years. | FSIQ subsample (*n*=269): *M* = 90.8 (*SD*= 19.4).Verbal function NR | 727 | Cross-sectional | Parent | Internal consistency;  Factor analysis (CFA) |
| CBCL6–18 | La Buissonniere Ariza et al. (2022) | US | Clinical. 81% boys. Age 7–13 years. Co-occurring anxiety/OCD | FSIQ *M* = 100 (*SD*=16.3); Mild ID = 19% | 166 | Cross-sectional | Parent | Internal consistency |
|  |  |  |  |  |  |  |  |  |
|  |  |  |  |  |  |  |  |  |
|  |  |  |  |  |  |  |  |  |
| CBCL6–18  CBCL6–18 | Magyar & Pandolfi (2017); Pandolfi et al. (2012; 2014)  Manning et al. (2011) | US  US | Archival data from genotype-phenotype study. 82% boys. Age 6–18 years.  Community/ clinic. 83% boys. Age 6–12 years. | FSIQ < 70 = 31.3%  Verbal function NR  NR | 122/76  77/93  195 | Cross-sectional  Cross-sectional | Parent  Parent | Internal consistency;  Factor structure (CFA)  Convergent validity (KSADS); Criterion validity (diagnosis)  Internal consistency |
|  |  |  |  |  |  |  |  |  |
| CBCL6–18  YSR | Mazefsky et al. (2014) | US | Community. 96% boys. Age 12–19 years. | FSIQ *M* =110.48 (*SD*=13.59)  Verbal function NR | 25 | Cross-sectional | Self-report  Parent | Internal consistency; Convergent validity (SCARED C; MFQ) |
|  |  |  |  |  |  |  |  |  |
|  |  |  |  |  |  |  |  |  |
|  |  |  |  |  |  |  |  |  |
| CBCL1.5–5  6–18 | Medeiros et al. (2017) | US | Simons Simplex Collection/Autism Treatment Network Registry study. 83% boys. Age 2–18 years. | FSIQ *M* = 84.01 (*SD*=23.94)  Verbal function NR | 623/225/ 398 | Cross-sectional; multi-site | Parent | Factor structure (EFA/CFA) |
| CBCL1.5–5 | Mello et al. (2022) | US | Clinical sample.  83% boys.  Age *M* =4.3 years (*SD*=1.1) | ID =34.4%  Verbal function NR | 157 | Cross-sectional data | Parent  Teacher | Inter-rater |
|  |  |  |  |  |  |  |  |  |
|  |  |  |  |  |  |  |  |  |
|  |  |  |  |  |  |  |  |  |
| CBCL 6–18 | Nadeau et al. (2015) | US | Clinical sample. 79% boys. Age 7–16 years. | NR | 102 | Cross-sectional | Parent | Internal consistency |
|  |  |  |  |  |  |  |  |  |
| CBCL1.5–5 | Pandolfi et al. (2009) | US | Genotype-phenotype study. 89% boys. Age *M* = 42.43 months (*SD*=10.19) | FSIQ *M* = 62.25 (*SD*=18.96).  VABS Communication: *M* =70.06 (*SD* = 17.13) | 128 | Cross-sectional | Parent | Internal consistency; Factor structure (CFA) |
| CBCL1.5-5/6–18 | Piro-Gambetti et al. (2023) | US | Clinical/community sample. 86% boys. Age 5–12 years. | ID = 34%  Verbal function NR | 188/376 | Cross-sectional data | Parent | Internal consistency |
|  |  |  |  |  |  |  |  |  |
| CBCL4–18  YSR | Pisula et al. (2017) | Poland | Clinical /community sample. Separate girls/boys sample Age 11–18 years. | FSIQ *M* = 102.46/103.94 (*SD*=15.52/13.82)  verbal function NR | 35 girls  35 boys | Cross-sectional | Self-report  Parent | Inter-rater |
| CBCL1.5–5 | Rivard et al. (2023) | Canada | Two clinical sample. 80% boys. 1–7 years. | FSIQ *M*=76.49 (*SD*=21.28). Verbal function NR | 243 | Cross-sectional | Caregiver | Internal consistency |
|  |  |  |  |  |  |  |  |  |
|  |  |  |  |  |  |  |  |  |
|  |  |  |  |  |  |  |  |  |
|  |  |  |  |  |  |  |  |  |
|  |  |  |  |  |  |  |  |  |
| CBCL1.5–5  6–18  TRF | Rodriguez et al. (2019); Rodriguez et al. (2021) |  | Longitudinal community study.86% boys. Age 5–12 years. | ID = 34.6%. Verbal function NR | 188 | Cross-sectional data | Parent  Teacher | Internal consistency |
|  |  |  |  |  |  |  |  |  |
| CBCL 6–18 | Schiltz & Magnus (2020) | US | National database 80% boys. Age *M* =10.89 (*SD*=2.80) | FSIQ *M* = 96.93 (*SD*=20.22). Verbal function NR | 502 | Cross-sectional | Parent | Factor structure (measurement invariance; gender) |
|  |  |  |  |  |  |  |  |  |
|  |  |  |  |  |  |  |  |  |
|  |  |  |  |  |  |  |  |  |
|  |  |  |  |  |  |  |  |  |
| CBCL 6–18  TRF | Stratis & Lecavalier (2017) | US | Simon simplex collection. 85% boys. Age *M* = 125.42 months (*SD*=35.6 months) | FSIQ/verbal function NR. VABS adaptive composite *M* = 71.37 (*SD*=11.8) | 403 | Cross-sectional | Parent  Teacher | Inter-rater |
|  |  |  |  |  |  |  |  |  |
|  |  |  |  |  |  |  |  |  |
| CBCL1.5–5  6–18  TRF | Ung et al. (2017) | US | Clinical chart review. >81% boys. Two samples. Age 2–5 years/6–10 years. | FSIQ *M* =82.80/78.14 (*SD*=11.71/9.19)  Verbal function NR | 26/32 | Cross-sectional | Parent  Teacher | Inter-rater |
|  |  |  |  |  |  |  |  |  |
| CBCL1.5–5 | Xu et al. (2014) | US | Community/clinical sample. 72% boys. Age 2.5–5 years. | FSIQ/verbal function NR. No severe ID. | 33 | Cross-sectional | Parent | Internal consistency |
|  |  |  |  |  |  |  |  |  |
|  |  |  |  |  |  |  |  |  |
|  |  |  |  |  |  |  |  |  |
| *BASC-2* | Lane et al. (2013) | Australia | Early intervention program  82% boys. Age 34–71 months. | VABS-II adaptive composite *M* =70.54 (*SD*=11.40). VABS-II communication *M* = 71.54 (*SD*=18.05). | 22 | Cross-sectional | Parent  Teacher | Inter-rater |
|  |  |  |  |  |  |  |  |  |
|  | Taylor et al. (2020) | US | Sample awaiting social intervention program. 81% boys. Age 8–16 years. | FSIQ *M* =101.66 (*SD*= 15.59)  Verbal function NR | 44 | Cross-sectional | Self-report  Parent | Inter-rater; Convergent validity (Children’s Depression Inventory; The Multidimensional Anxiety Scale for Children) |
| *BISCUIT* Part 2  Comorbid Psycho-pathology | Horovitz & Matson (2015); Matson et al. (2009; 2011) | US | EarlyS teps program.  72% boys. Age 17–37 months. | NR | 270/309/805 | Cross-sectional | Parent | Internal consistency; Factor structure (EFA) Normative data |
| Part 3  Challen-ging Behaviors | Horovitz & Matson, (2013);  Matson et al. (2009); Rojahn et al. (2009); |  | See under BISCUIT Part 2 above. Same sample. |  | 270/312 | Cross-sectional | Parent | Internal consistency; Factor structure (EFA); Normative data |
|  |  |  |  |  |  |  |  |  |
|  |  |  |  |  |  |  |  |  |
| *BPI*  Short form | Higgins et al. (2022); Leader, Dooley et al. (2021); Leader, Flynn et al. (2021); Leader et al. (2022) |  | See under ASD-CC above. Same sample |  | 133/118 |  | Parent | Internal consistency; Convergent validity (ASD-CC) |
|  |  |  |  |  |  |  |  |  |
|  | Pozo & Sarria (2014) | Spain | School sample. 79% male. Age 4–38 years | FSIQ/verbal function NR.25% in ordinary school. | 59 | Cross-sectional | Parent | Internal consistency |
|  | Wei et al. (2023) | China | Convenience sample. 76% boys. Age 2–12 years | ID = 39%. Verbal function NR. | 216 | Cross-sectional | Caregiver | Internal consistency |
|  |  |  |  |  |  |  |  |  |
|  |  |  |  |  |  |  |  |  |
| *CASI* | Bitsika et al. (2016) | Australia | See ABC above. Same sample. |  |  |  | Self-report  Parent | Internal consistency; Inter-rater; Factor structure (EFA) |
|  | Kaat et al. (2013) | US | Clinical sample.86% boys. Age 6–12 years | FSIQ *M* =85.0 (*SD*=23.0). ID = 23%. Verbal function NR | 115/77 | Cross-sectional | Parent  Teacher | Inter-rater |
|  |  |  |  |  |  |  |  |  |
| School version | Lecavalier et al. (2009) | US | Clinical sample. 84% boys. Age 6–12 years. | FSIQ > 70 =73%  Verbal function NR | 498 | Cross-sectional | Parent  Teacher | Factor structure (CFA) |
| Pre-school version | Lecavalier et al. (2011) | US | Clinical sample. 78% boys. Age 3–5 years. | FSIQ > 70 = 66%  Verbal function NR | 229 | Cross-sectional | Parent  Teacher | Inter-rater  Factor structure (CFA) |
|  |  |  |  |  |  |  |  |  |
|  |  |  |  |  |  |  |  |  |
| *ChIPS*  Parent version | Witwer et al. (2012) | US | Clinic/community. 82% boys. Age 6–17 years. | FSIQ *M* =68.4 (*SD*= 23.3). 23% non-conversational language. | 61 | Cross-sectional | Parents | Internal consistency; Inter-rater; Convergent validity (CASI) |
| *C-SHARP* |  |  |  |  |  |  |  |  |
|  | Farmer et al. (2016) | US | Clinic/research centers. 84% boys. Age 2–21 years. | FSIQ > 70 = 48%  Verbal function NR | 406 | Cross-sectional multi-site | Parent | Internal consistency; Factor structure (measurement invariance) |
|  | Kirst et al. (2022) | Germany | Clinical RCT trial. 83% boys. Age 5–10 years. | FSIQ≥70. Verbal function NR | 60 | Cross-sectional  multi-site | Parent | Internal consistency |
|  |  |  |  |  |  |  |  |  |
|  |  |  |  |  |  |  |  |  |
|  |  |  |  |  |  |  |  |  |
|  |  |  |  |  |  |  |  |  |
|  |  |  |  |  |  |  |  |  |
| *CRS-R* |  |  |  |  |  |  |  |  |
|  | Pearson et al. (2012) | US | Community/clinic. 76% boys. Age 6–13 years. | FSIQ *M* = 84.1 (*SD*=19.6). VABS-II Communication *M* =76.2 (*SD*=8.6) | 86 | Cross-sectional | Parent  Teacher | Inter-rater |
| *DASS-21* |  |  |  |  |  |  |  |  |
| *DBC-P* | Park et al. (2020) | Australia | Clinical sample. 69% male. Age 16–46 years | FSIQ = >70.  Verbal function NR | 123 | Cross-sectional | Self-report | Internal consistency; Factor structure (CFA); Convergent validity (Hamilton depression) |
|  |  |  |  |  |  |  |  |  |
|  | Adams et al. (2019) | Australia | Longitudinal: Students with autism. 83% boys. Age 4–5 years | FSIQ/verbal function: NR  VABS-II score <70 on 3 domains = 24.6%. Mainstream school without support = 14.6% | 130 | Cross-sectional data T1 | Parent | Internal consistency |
|  |  |  |  |  |  |  |  |  |
|  | Chandler et al. (2016) | UK | Cohort study.81% boys. Age 4–8 years. | FSIQ *M* = 72.7. FSIQ <70 = 35%. FSIQ <50 =21.6% | 277 | Cross-sectional | Parent  Teacher | Internal consistency; Inter-rater |
|  |  |  |  |  |  |  |  |  |
|  | Hastings et al. (2005) | UK | Part of autism program. 85% boys. Age 28–45 months. | NR | 48 | Cross-sectional | Parent | Inter-rater |
|  |  |  |  |  |  |  |  |  |
| Short form | Jellett et al. (2015) | Australia | Community sample.84% boys. Age 16–71 months. | NR  High functioning = 36.1% | 97 | Cross-sectional | Parent | Internal concistency |
|  |  |  |  |  |  |  |  |  |
|  |  |  |  |  |  |  |  |  |
|  |  |  |  |  |  |  |  |  |
|  | Khor et al. (2014) | Australia | Community sample. 84% boys. Age 12–18 years. | FSIQ *M* = 99.87 (*SD*= 14.33). Verbal IQ *M* = 103.45 (*SD*=14.66) | 31 | Follow-up | Parent | Internal consistency; Test-retest |
|  |  |  |  |  |  |  |  |  |
|  | Magiati et al. (2016) | Singapore | Special schools. 81% boys. Age 5–17 years. | Adaptiv standard score *M* = 58.8 (*SD* = 40.4). Probably some with IQ <70. | 241 | Cross-sectional | Parent | Internal consistency; Convergent validity (Spence Children’s Anxiety Scale) |
|  |  |  |  | Verbal function NR |  |  |  |  |
|  |  |  |  |  |  |  |  |  |
|  |  |  |  |  |  |  |  |  |
|  |  |  |  |  |  |  |  |  |
| *ECBI* | Brookman-Frazee et al. (2018); Martinez et al. (2023) | US | Outpatients/school-based programs. 84% boys. Age 4–14 years. | FSIQ *M* = 88.47 (*SD*=16.53)  Verbal function NR | 201 | Cross-sectional | Parent | Internal consistency; Factor structure (CFA); Convergent validity (MINI; Competing Behavior Scale) |
|  |  |  |  |  |  |  |  |  |
|  | Jeter et al. (2017) | US | Clinical/Autism network. 83% boys. Age 2–12 years. | NR | 335 | Cross-sectional | Parent | Internal consistency; Factor structure (EFA); Convergent validity (BASC-2) |
|  |  |  |  |  |  |  |  |  |
|  |  |  |  |  |  |  |  |  |
|  |  |  |  |  |  |  |  |  |
| *EDI* | Day et al. (2024) | US | Clinical sample. 72% boys. Age 2–5 years | NR | 1369 | Cross-sectional | Parent | Factor structure (EFA/CFA); Convergent validity (Multidimensional Assessment of Profiles Scales; PROMIS-emotion-focused scales) |
|  |  |  |  |  |  |  |  |  |
|  | Mazefsky, Day et al. (2018) | US | New measure.  Clinical inpatients. 80% boys. Age 4–20 years. | FSIQ *M* = 77  Non-/minimally verbal = 48.1%;  Verbal = 51.9% | 219 | Admission/ discharge | Parent | Content validity |
|  | Mazefsky, Yu et al. (2018) | US | Interactive Autism Network. Autism Inpatient Collection. 79% boys. Age 4–20 years | FSIQ ≥70 =27.8%  Verbally fluent = 55.3% | 1755 | Cross-sectional  Follow-up | Parent | Test-retest; Criterion validity (group-differences); Factor structure (EFA/CFA); Convergent validity (ABC; CBCL) |
|  | Riek et al. (2023) | US | Part of clinical trial.84% boys. Age 12–21 years | NR | 52 | Cross-sectional | Caregiver | Internal consistency; Convergent validity (PROMIS anxiety/ depression; CBCL) |
|  | Skwerer et al. (2019) | US | Community.  Age 5–18 years. | Intellectually heterogeneous.  Minimally verbal | 65 | Cross-sectional | Parent | Convergent validity (CASI) |
|  | Taylor et al. (2021) |  | See under ABC above.  Identical sample. |  |  |  |  | Internal consistency |
| *HADS* |  |  |  |  |  |  |  |  |
|  |  |  |  |  |  |  |  |  |
|  |  |  |  |  |  |  |  |  |
|  | Uljarevic et al. (2018) | Australia/UK | Community sample. 69% male. Age Australia *M* =18.35 years (*SD =* 2.55). Age UK *M* =16.04 years (*SD*=1.28). | NR | 151 | Cross-sectional | Self-report | Internal consistency. Factor structure (EFA); Convergent validity (SDQ; DSM-5 DAS anxiety; PHQ-9 depression) |
| *ITSEA* |  |  |  |  |  |  |  |  |
|  | Davis & Carter (2008) | US | Convenience sample. 74% boys. Age *M* =26.9 months (*SD*=4.2) | FSIQ NR.  Mullen scales T-scores Receptive Language *M =* 24.5 (*SD* = 7.5), Expressive Language *M =* 29.9 (*SD* = 10.4) | 54 | Cross-sectional | Parent | Inter-rater |
| *K-CSCB* |  |  |  |  |  |  |  |  |
|  | Kim et al. (2018) | Korea | New measure. Clinical/special schools. 79% boys. Age 5–22 years. | NR | 189/32 | Cross-sectional | Parent | Internal consistency; Test-retest; Convergent validity (BPI; CBCL) |
| *KSADS* |  |  |  |  |  |  |  |  |
| Present | Gjevik et al. (2011) | Norway | See under CBCL above. Same sample. |  | 18 |  | Parent/clinician | Inter-rater |
|  |  |  |  |  |  |  |  |  |
| Present  Lifetime | Hepburn et al. (2014) | US | Anxiety CBT study. 81% boys. Age 8–14 years. | FSIQ *M* = 98.4 (*SD*=15.01). Verbal IQ *M* = 100.1 (*SD*= 13.87) | 42 | Cross-sectional | Parent/clinician | Inter-rater |
|  |  |  |  |  |  |  |  |  |
| Present  Lifetime | Mattila et al. (2010) | Finland | Community/clinical sample. 76% boys. Age 9–16 years. | FSIQ >75  Verbal function NR | 50 | Cross-sectional | Self-report  Parent  Clinician | Inter-rater |
|  |  |  |  |  |  |  |  |  |
| *MCAS* | Kalb et al. (2018) | US | New measure. Interactive Autism network. 83% boys. Age 3–25 years. | NR | 606 | Cross-sectional | Parent | Internal consistency; Content validity; Factor structure (EFA/CFA); Convergent validity; Criterion validity (clinician determination of crises) |
| *MINI* |  |  |  |  |  |  |  |  |
|  | Mosner et al. (2019) | US | Austim Research Registry. >82% boys/male. Children age *M* = 14.03 (*SD* = 1.95).Adults *M* = 20.19 years (*SD* = 2.29) | FSIQ_child_ *M* = 100.85 (*SD*=17.11); FSIQ_adults_ *M* 107.00 (*SD*=13.99).  All participants: fluent phrase speech. | 35/  32 | Cross sectional | Self-report  Parent/clinician | Criterion validity (community diagnosis). |
|  |  |  |  |  |  |  |  |  |
|  | Brookman-Frazee et al. (2018); Stadnic et al. (2017) |  | See under ECBI above. Same sample. |  |  |  | Parents/ clinician | Inter-rater; Convergent validity (ECBI); Criterion validity (clinical diagnosis) |
|  |  |  |  |  |  |  |  |  |
| *MINI-PAS-ADD* |  |  |  |  |  |  |  |  |
|  | Buck et al. (2014) | US | Population-based follow-up study. 75% male. Age 26–54 years. | FSIQ >70 =24%  Mild ID =28.8%  Severe ID=47.9% | 129 | Cross-sectional data | Caregiver | Criterion validity (caregiver reported diagnosis) |
| *Nisonger* |  |  |  | Verbal function NR |  |  |  |  |
| Problem behavior | Beer et al. (2013) | Australia | Clinical sample. 85% boys. Age 3–20 years. | NR | 28 | Cross-sectional | Parent | Internal consistency |
|  |  |  |  |  |  |  |  |  |
|  | Bekhet (2016) | US | Community/research registry. 85% boys. Age 3–17 years. | NR | 117 | Cross-sectional | Parent | Internal consistency |
|  |  |  |  |  |  |  |  |  |
|  | Benson (2015) | US | School sample. Longitudinal study. 85% boys. Age 7–14 years. | FSIQ NR. Primarily nonverbal = 79% | 113 | Cross-sectional data | Parent | Internal consistency |
|  |  |  |  |  |  |  |  |  |
|  | Gardiner & Iarocci (2015) | Canada | School sample. 85% boys. Age 6–18 years. | FSIQ/verbal function NR. No ID. VABS-II adaptive composite *M* = 77.56 (*SD*= 12.59) | 84 | Cross-sectional | Parents | Internal consistency |
|  |  |  |  |  |  |  |  |  |
|  | Firth & Dryer (2013) | Australia | Community sample. 79% boys. Age 4–12 years. | NR | 109 | Cross-sectional | Parent | Internal consistency |
|  |  |  |  |  |  |  |  |  |
|  | Fong et al. (2020); Fong et al. (2021) | Canada | Convenience sample. 87% male. Age 2–35 years. | FSIQ/verbal function NR. Majority non-ID as parent reported. | 164 | Cross-sectional | Parent | Internal consistency |
|  |  |  |  |  |  |  |  |  |
|  | Lecavalier et al. (2004;2006) | US | Clinical/community samples. 84% boys. Age 3–18 years. | FSIQ/verbal function NR. 64% in the range of ID based on adaptive measure | 330 | Cross-sectional; multi-site | Parent  Teacher | Internal consistency; Inter-rater; Factor structure (EFA/CFA) |
|  |  |  |  |  |  |  |  |  |
|  | Weiss et al. (2012) | Canada | Community sample. 81% boys. Age 6–21 years. | FSIQ/verbal function NR | 228 | Cross-sectional | Parent | Internal consistency |
| *OSCA-ABP* |  |  |  |  |  |  |  |  |
|  | Palmer et al. (2021); Palmer et al. (2023) | UK | New measure. Parent intervention. 85% boys. Age 4–8 years. | FSIQ NR.  47% minimally verbal. Fluent speech =53% | 83 | Cross-sectional | Clinician | Inter-rater; Content validity; Convergent validity (ABC) |
|  |  |  |  |  |  |  |  |  |
| *PAC* | Bakken et al. (2010) | Norway | Population based-sample. 73% male. Age 14–57 years. | All ID. FSIQ/verbal function NR. | 62 | Cross-sectional | Caregiver/clinician | Inter-rater |
|  | Bakken et al. (2023);  Helverschou et al., (2021); Kildahl & Helverschou (2023); Kildahl, Ludvigsen et al. (2023) | Norway | The Autism, ID and Mental Illness study. 68% male. Age 16–58 years | All ID. FSIQ/verbal function NR | 183 | Cross-sectional/ | Caregiver/Clinician | Internal consistency  Criterion validity (clinical diagnoses); Convergent validity (ABC) |
|  |  |  |  |  |  |  |  |  |
|  | Helverschou et al. (2009) | Norway | Clinical sample. 74% male. Age 17–56 years. | All ID. FSIQ/verbal function NR. | 35 | Cross-sectional | Caregiver/Clinician | Internal consistency; Inter-rater; Criterion validity (clinical diagnoses) |
|  |  |  |  |  |  |  |  |  |
|  |  |  |  |  |  |  |  |  |
|  |  |  |  |  |  |  |  |  |
| *RCADS* |  |  |  |  |  |  |  |  |
|  | Kaat & Lecavalier (2015) | US | Clinical/community sample. 83% boys. Age 8–16 years. | FSIQ *M* = 90.7 (*SD:* NR). Verbal function NR. | 46 | Cross-sectional/test-retest | Parents/ Youths | Internal consistency; Test-retest; Inter-rater; Convergent validity (CSI-4) |
|  | Khalfe et al. (2023) | US | Psychotherapy trial. 85% male. Age 6–14 years. | FSIQ and verbal comprehension IQ >70. | 74 | Cross-sectional | Self-report  Parent | Internal consistency; Inter-rater; Convergent validity (Pediatric Anxiety Rating Scale; CBCL) |
|  |  |  |  |  |  |  |  |  |
|  | Steerling et al. (2015) | US | Clinical sample. Gender NR. Age 11–15 years. | Sample 1: FSIQ *M* = 87.55 (*SD*=11.79). Sample 2: FSIQ >85 | 67 | Cross-sectional; multi-site | Self-report | Internal consistency; Convergent validity (CBCL; Multi-Dimensional Anxiety Scale; Pediatric Anxiety Rating Scale; ADIS-C/P) |
|  |  |  |  |  |  |  |  |  |
|  |  |  |  |  |  |  |  |  |
| *SDQ* | Alallawi et al. (2022) | UK  Arabic version | Arab families living in the UK.78% boys.  Age 4–15 years. | NR | 100 | Cross-sectional | Parent | Internal consistency |
|  | Burton et al. (2020) | Australia | Non-clinical school-based sample.89% boys. Age 7–13 years. | FSIQ>70. Verbal function NR | 217 | Cross-sectional | Parent  Teacher | Internal consistency |
|  | Deniz & Toseeb (2023) | UK | Millennium Cohort Study.78% boys, Age M =11.20 years | NR | 416 | Cross-sectional data | Self-report | Internal consistency |
|  |  |  |  |  |  |  |  |  |
|  | Findon et al. (2016) | UK | Clinical sample. > 75% male. Age 14–59 years | ID = 8%/ verbal function NR. | 126/79 | Cross-sectional | Self-report  Parent | Internal consistency; Inter-rater; Convergent validity (DAWBA;HADS). Criterion validity (DAWBA) |
|  |  |  |  |  |  |  |  |  |
|  | Hastings et al. (2022) | UK | School sample. 85% boys. Age 5–17 years. | NR | 160 | Cross-sectional | Parent | Internal consistency |
|  |  |  |  |  |  |  |  |  |
|  | Jones et al. (2014) | UK | Community sample.83% boys. Age 7–16 years | FSIQ/verbal function NR. ID =71% | 71 | Cross-sectional | Parent | Internal consistency |
|  |  |  |  |  |  |  |  |  |
|  | Kang et al. (2020) | China | Community sample. 80% boys. Age 3–6 years. | NR | 114 | Cross-sectional | Parent | Internal consistency |
|  |  |  |  |  |  |  |  |  |
|  |  |  |  |  |  |  |  |  |
|  |  |  |  |  |  |  |  |  |
|  | Khor et al. (2014) |  | See under DBC above. Same sample |  |  |  | Self-report | Internal consistency |
|  |  |  |  |  |  |  |  |  |
|  | Lovell et al. (2016; 2020) | UK/USA/ Australia | Convenience community sample. Gender NR. Age 3–19 years. | High functioning = 73%. FSIQ/verbal function NR | 153 | Cross-sectional | Parent | Internal consistency |
|  |  |  |  |  |  |  |  |  |
|  | Lu, Chen et al. (2021); Lu, Wang et al. (2021) | China | Community sample.83% boys. Age *M* =10.56 years (*SD*=5.63) | NR | 306 | Cross-sectional | Parent | Internal consistency |
|  | McIntyre et al. (2023) | US | Community sample.  82% boys. Age *M* =7.72 (*SD*=1.59) | NR | 68 | Cross-sectional | Parent | Internal consistency |
|  |  |  |  |  |  |  |  |  |
|  |  |  |  |  |  |  |  |  |
|  | Milosavljevic et al. (2016) | UK | Special needs and Autism project cohort. 89% boys. Age 14–16 years. | FSIQ *M* =92.77 (*SD*=12.45). Verbal IQ *M* = 86.77 (*SD*=13.47). | 56 | Cross-sectional | Parent | Internal consistency |
|  |  |  |  |  |  |  |  |  |
|  | Miranda et al. (2019) | Spain | Community sample. 92% boys. Age 7–11 years. | FSIQ *M* =101.42 (*SD*=12.65). Vocabulary subtest WISC-IV = 11.51 (*SD*=3.34) | 52 | Cross-sectional | Parent | Internal consistency |
|  | Plak et al. (2023) | The Netherlands | The Netherlands Autism Register. 78% boys. Age *M* =12.6 years (*SD*=3.8). | FSIQ <86 = 27%  Verbal function NR | 226 | Cross-sectional data | Caregiver | Internal consistency |
|  |  |  |  |  |  |  |  |  |
|  | Pruitt et al. (2018) | US | Community sample. 83% boys. Age 2–16 years. | FSIQ/verbal function NR. 25% in ordinary school. | 98 | Cross-sectional. | Parent | Internal consistency |
|  | Reyes et al. (2020) | US | Community sample. 90% boys. Age 42-94 months | A receptive language mental age cut-off of 24-months | 22 | Cross-sectional | Parent | Internal consistency |
|  |  |  |  |  |  |  |  |  |
|  |  |  |  |  |  |  |  |  |
|  |  |  |  |  |  |  |  |  |
|  | Rixon et al. (2021) | UK | Research study on ASD/family. 81% boys. Age 5–17 years. | FSIQ/verbal function NR. Mainstream school with no support = 14%. | 168 | Cross-sectional | Parent | Internal consistency |
|  |  |  |  |  |  |  |  |  |
|  | Salomone et al. (2014) | UK | Specialist ASD schools. 78% boys. Age 4–19 years. | Majority considered to have ID. Verbal level NR. | 615 | Cross-sectional | Parent  Teacher | Internal consistency; Inter-rater |
|  |  |  |  |  |  |  |  |  |
|  | Salomone et al. (2019) | Italy | Neuropsychiatry services. 78% boys. Age 22–61 months. | Developmental age *M* = 23.59 months (*SD*= 6.9) | 82 | Cross-sectional | Parent | Internal consistency |
|  |  |  |  |  |  |  |  |  |
|  |  |  |  |  |  |  |  |  |
|  | Totsika et al. (2013) | UK | Population-defined cohort: longitudinal study. 82% boys. Age 5 years. | ID = 21%. Verbal function NR | 132 | Cross-sectional data | Parent | Internal consistency |
|  |  |  |  |  |  |  |  |  |
|  | Wang et al. (2016)  Werkman  et al. (2020) | China  The Nether-lands | Special schools. 83% boys. Age 6–17 years.  Autism register. 79% male. | FSIQ/verbal function NR.    ID = 25%. Verbal level NR. | 60  241 | Cross-sectional  Cross-sectional | Parent  Caregiver | Internal consistency  Internal consistency |
|  | Yan et al. (2023) | China | Convenience sample.86% boys. Age *M* =7.07 years (*SD*=0.99) | NR | 221 | Cross-sectional | Parent | Internal consistency |
|  | Yang et al. (2023) | China | Special schools/support centers.84% boys. Age *M* = 8.46 years (*SD*= 3.42). | NR | 286 | Cross-sectional | Parent | Internal consistency |
| *SIB-R* |  |  |  |  |  |  |  |  |
|  | Mihaila & Hartley (2018) | US | Longitudinal study: community sample. 86% boys. Age 5–12 years. | ID = 33%. Verbal function NR | 176 | Cross sectional data | Parent | Internal consistency; Inter-rater; Convergent validity (CBCL) |
|  |  |  |  |  |  |  |  |  |
| *SSIS-RS* | Schiltz et al. (2018) | US | PEERS intervention study. 82% boys. Age: *M* =13.58 years (*SD*=1.46) | FSIQ *M* = 99.25 (*SD*=18.01). Verbal function NR. | 77 | Cross-sectional | Parent | Internal consistency |
|  |  |  |  |  |  |  |  |  |
|  |  |  |  |  |  |  |  |  |
|  |  |  |  |  |  |  |  |  |
|  |  |  |  |  |  |  |  |  |
|  |  |  |  |  |  |  |  |  |

*Note**.* ABC = Aberrant Behavior Checklist; ABI = Autism Behavior Inventory; ACB = Assessment of Concerning Behavior Scale; ACI-PL = Autism Comorbidity Interview; ADIS-IV = Anxiety Disorders Interview Schedule for DSM-IV; ASD-CA = Autism Spectrum Disorders Comorbidity for Adults; ASD-CC = Autism Spectrum Comorbid for Children; ASD-PBC = Autism Spectrum Disorder Problem Behavior Child version; ASEBA = Achenbach System of Empirically Based Assessment; BASC-2= Behavioral Assessment System for Children; BISCUIT-Part 2 = Baby and Infant Screen for Children with Autism Traits–Part 2 Comorbid Psychopathology; BISCUIT-Part 3 = Challenging Behavior; BPI = Behavior Problems Inventory; CASI = Child and Adolescent Symptom Inventory; ChIPS = Children’s Interview for Psychiatric Syndromes; C-SHARP = Children’s Scale of Hostility and Aggression; CRS-R = Conners Rating Scale Revised; DASS-21 = Depression, Anxiety, and Stress Scale; DBC = Developmental Behavior Checklist; ECBI = Eyberg Child Behavior Inventory; FSIQ = full-scale IQ; EDI = Emotion Dysregulation Inventory; HADS = Hospital Anxiety and Depression Scale; ITSEA= Infant Toddler Social Emotional Assessment; K-CSCB = Korean Comprehensive Scale for the Assessment of Challenging Behavior in Developmental Disorder; KSADS = Schedule for Affective Disorders and Schizophrenia for School-Age Children; MCAS = Mental Health Crisis Assessment Scale; MINI = Mini International Neuropsychiatric Interview; Nisonger = Nisonger Child Behavior Rating Form; OSCA-ABP = Observation Schedule for Children with Autism; PAC = Psychopathology in Autism Checklist; RCADS = Revised Child Anxiety and Depression Scale; SDQ = Strengths and Difficulties Questionnaire; SIB-R = Scales of Independent Behavior–Revised; SSIS-RS = Social Skills Improvement System-Rating Scales; EFA = exploratory factor analysis; CFA = confirmatory analysis; NR = not reported; CBT = Cognitive behavior therapy
