# Supplementary material for: Applicability and Psychometric Properties of General Mental Health Assessment Tools in Autistic People: A Systematic Review
Source: J Autism Dev Disord. 2024 Apr 13;55(5):1713–26. doi: 10.1007/s10803-024-06324-3 (PMC12021962; doi:10.1007/s10803-024-06324-3)
Supplement: Supplementary file 8 — Supplementary file8 (DOCX 39 KB) [file 10803_2024_6324_MOESM8_ESM.docx]

**Appendix H**

*Description of Included Instruments from all Studies*

| Instrument | Purpose/  composition | Administration and scoring | Framework |
| --- | --- | --- | --- |
| *ASDD/IDD-specific instruments*  *Aberrant Behavior Checklist-Community version* (*ABC*; Aman & Singh, 1986) | Age: Children–adults  58 items, 5 subscales: Irritability, Social withdrawal, Stereotypic behavior, Hyperactivity, Noncompliance, Inappropriate speech | Proxy  4-point scale (0–3) | Empirically derived |
| *Autism Behavior Inventory: MH subscales (ABI;* Bangerter et al., 2017*)* | Age: 3 years–adulthood  New measure.30 items, 3 MH subscales:  Mental health, Self-regulation, Challenging behavior | Proxy  Web-based  6-point scale (0–6)  Frequency/intensity | Literature review. Expert opinion. Feedback from parents. Empirically derived |
| *Assessment of Concerning Behavior (ACB;* Tarver et al., 2021) | Age: Children–adults  New measure.35 items (parent version)/41 items (child version), 2 subscales: Internalizing, Externalizing. Total score | Child self-report (7–11 years)  Self-report (≥12 years)  Teacher  Parent | Literature review. Clinical panel. Focus groups with autistic community.  Empirically derived |
| *Autism Comorbidity Interview Present and Lifetime Version (ACI-PL;* Leyfer et al., 2006) | Age: 5–17 years  New adapted measure from KSADS. Psychiatric diagnoses for major psychiatric disorders: Depression, ADHD, and OCD modules examined in the validation study. | Diagnostic interview administered to parent by clinician | DSM-IV-TR  Modification of the KSADS: includes additional screening  questions and coding options |
| *Autism Spectrum Disorders Comorbidity for Adults (ASD-CA;* Matson & Boisjoli, 2008) | Adults  New measure. 37 items, 5 subscales: Anxiety/Repetitive behaviors, Conduct problems, Irritability/Behavior excesses, Attention/Hyperactivity/Impulsivity, Depressive symptoms | Proxy  2-point scale (0–1) | Comorbid symptoms were obtained from scales (e.g., DASH-II), in addition to DSM-IV-R and ICD-10, and from symptom descriptions in the literature. |
| *Autism Spectrum Disorder Comorbid for Children (ASD-CC;* Matson & Gonzalez, 2007) | Age: 3–17 years  39 items, 7 subscales: Tantrum, Repetitive, Worry/Depressed, Avoidant, Undereating, Conduct, Overeating | Proxy  3-point scale (0–2) | DSM-IV-TR and ICD-10 |
| *Autism Spectrum Disorder-Problem Behavior Child Version (ASD-PBC;* Matson et al., 2008) | Age: 3–17 years  18 items, 2 subscales: Externalizing, Internalizing | Proxy  3-point scale (0–2) | Not reported |
| *Baby and Infant*  *Screen for Children with Autism Traits-Part 2 Comorbid Psychopathology (BISCUIT-Part 2;* Matson et al., 2009) | Age: 17–37 months  57 items, 5 subscales: Tantrum/Conduct, Inattention/Impulsivity, Avoidance, Anxiety/Repetitive, Eating/Sleeping | Proxy  3-point scale (0–2) | Comorbid symptoms were obtained from a review of the literature, DSM-IV-TR and ICD-10, and by expert consensus. |
| *Baby and Infant Screen for*  *Children with Autism Traits Challenging Behavior (BISCUIT-Part 3;* Matson, et al., 2009) | Age: 17–37 months of age  15 items, 3 subscales: Aggressive/Destructive behavior, Stereotypies, Self-injurious behavior | Proxy  3-point scale (0–2) | Comorbid symptoms were obtained from a review of the literature, DSM-IV-TR and ICD-10, and by expert consensus. |
| *Behavior Problems Inventory* (*BPI*-*01*; Rojahn et al., 2001) | Age: Adolescents, adults  49 items, 3 subscales: Self-injurious behavior, Stereotyped behavior, Aggressive/Destructive behavior | Proxy  5-point frequency scale (0–4)  3-point severity scale (0–3) | Empirically derived |
| *Children’s Scale of Hostility and Aggression (C-SHARP;* Farmer & Aman, 2009) | Age: Children to adolescents  51 items, 5 Problem subscales: 1 Provocation scale (reactive, proactive). Verbal aggression, Bullying, Covert aggression, Hostility, Physical aggression | Proxy  4-point scale (Problem subscale)  5-point scale (Provocation scale) | Empirically derived |
| *Developmental Behavior Checklist* (*DBC*; Einfeld & Tonge, 1992) | Age: 4–18 years  96 items, 6 subscales: Disruptive, Self-absorbed, Communication disturbance, Anxiety, Social relating, Antisocial. Total behavior problem score | Proxy: Primary carer and teacher versions  3-point scale (0–2) | Empirically derived |
| *Emotion Dysregulation Inventory (EDI; Day et al., 2024: Mazefsky et al., 2016; Mazefsky et al., 2018)* | Age: ≥ 6 years and 2–5 years  30 items/22 items, 2 subscales: Reactivity and Dysphoria | Proxy  5-point scale (0-4) | Literature review. Conceptual model development. Feedback from parents. Expert panel. Empirically derived |
| *Korean Comprehensive Scale for the Assessment of Challenging Behavior in Developmental Disorder (K-CSCB;* Kim et al., 2018) | Age 5–22 years  New measure.75 items. 6 subscales: Self-harm. Aggressive, Stereotypy, Unresponsiveness, Attention deficit/hyperactivity, Inappropriate word and behavior | Proxy  5-point scale (0–4; frequency)  4-point scale (0–3; severity) | Items obtained from other scales. Information from caregivers and therapists about additional symptoms. Expert consensus. Empirically derived |
| *Mental Health Crisis Assessment Scale (MCAS;* Kalb et al., 2018) | Age: 3–25 years  New measure.  28 items; three sections: First sections; 14-item list of various MH behaviors Second section: Identify “single behavior” that could cause greatest harm  Third section:13-item list of dangerousness of the child’s behavior | Proxy  4-point scale (0–3; first section)  3-point scale (0–2, second section) | Literature review. Expert consensus. Empirically derived |
| *Mini Psychiatric Assessment Schedule for Adults with Developmental Disability* (*MINI-PAS-ADD;* Prosser et al., 1997) | Age: Adults  86 items; 7 domains: Depression, Expansive mood (hypomania/mania), Anxiety disorder, OCD, Psychosis, Autism, Unspecified disorder | Trained informant completed checklist.  4-point scale (0–3) | ICD-10 |
| *Nisonger Child Behavior Rating Form,* *Behavior Section* (*NCBRF*; Aman et al., 1996) | Age: Children and adolescents, 3–16 years of age  60 items; 6 subscales: Conduct problems, Insecure/anxious, Hyperactivity; Self-injury/Stereotypic, Self-isolated/Ritualistic, Overly sensitive. | Proxy: Parent and teacher versions.  4-point scale (0–3) | Modified the Child Behavior Rating Form. Empirically developed |
| *Observation Schedule for Children with Autism-Anxiety, Behavior, and Parenting (OSCA-ABP* (Palmer et al., 2021) | Age: Children (4–8 years)  New measure. Destructive behavior, Physical aggression towards others, Verbal aggression towards others, Physical aggression towards self, Verbal aggression towards self, Frustrated vocalizations, Non-compliance; Avoidance, Reassurance seeking. | Observation Schedule of child (and parenting behaviors)  Frequency/intensity. | Developed in the context of a pilot RCT. A priori defined domains/behaviors based on patient and public involvement. |
| *Psychopathology in Autism Checklist (PAC;* Helverschou et al., 2008) | Age: Older youth and adults  42 items, 5 subscales: General adjustment, Psychosis, Depression, Anxiety, OCD | Proxy  4-point scale (1–4) | DSM-IV/ICD-10 and expert consensus. Empirically developed |
| *Conventional instruments:* |  |  |  |
| *Anxiety Disorders Interview Schedule for DSM-IV–Child and Parent Version (ADIS-IV C/P;* Silverman & Albano, 1996) | Age: Youth  Presence, severity and level of  interference of anxiety disorders and common disorders in youth based upon the criteria set by the DSM-IV-TR | Child/parent interview  9-point scale (0-8; severity rating) | DSM-IV-TR |
| *ASEBA:Child Behavior Checklist* (*CBCL*; Achenbach & Rescorla, 2001) | Age versions: 1.5–5 years and 6–18 years  120 items, 8 syndrome scales: Withdrawn, Somatic Complaints, Anxious/Depressed, Social problems, Thought problems, Attention problems, Delinquent behavior, Aggressive behavior.  2 broadband scales: Internalizing and Externalizing. A total problems score.  The 2001 version includes 6 DSM-oriented subscales: Affective problems, Anxiety problems, Somatic problems, Attention deficit/hyperactivity problems, Oppositional defiant problems, Conduct problems. | Youth Self-Report  Proxy: Parent report (CBCL) and Teacher Report Form report.3-point scale (0–2) | Empirically developed  DSM-oriented scales (latest version) |
| *Behavioral Assessment System for Children, 2^nd^ Ed. (BASC-2;* Reynolds & Kamphaus, 2004*)* | Age: Preschool (2–5 years), children (6–11 years), and adolescent forms (12–21 years).  Behavioral symptoms (atypicality, withdrawal, attention problems).  Internalizing (anxiety, depression, somatization).  Externalizing (hyperactivity, aggression).  Adaptive skills omitted from this presentation. Behavioral symptoms index: Hyperactivity, Aggression, Anxiety, Depression, Atypicality, and Attention problems scales | Proxy (parent/teacher forms)  4-point scale (0–3) | Built around clearly specified constructs with matching item content, developed through a balance of theory and empirical data. |
| *Child and Adolescent Symptom Inventory (CASI;* Gadow & Sprafkin, 2010) | Age: 5–18 years  ADHD inattentive subtype, ADHD hyperactive-impulsive subtype, ADHD combined subtype, Oppositional defiant disorder (ODD), Conduct disorder (CD), Generalized anxiety disorder (GAD), Social phobia, Major depressive episode (MDE), Dysthymia, Manic episode, Schizophrenia, and Separation anxiety disorder | Proxy (parent/teacher forms)  4-point scale (0–3)  Symptom count (categorical)  Symptom severity (dimensional)  Impairment | DSM-IV-TR |
| *Children’s Interview for Psychiatric Syndromes (ChIPS;* Weller et al., 1999) | Age: 6–18 years  Structured interview designed to assess psychiatric diagnoses for major psychiatric disorders. | Diagnostic interview administered to parent/child by trained interviewer. | DSM-IV |
| *Conners’ Rating Scale Revised (CRS-R*; Conners, 1997) | Age: 6–18 years  80 items; 7 subscales  Oppositional, Cognitive Problems/Inattention,  Hyperactivity, Anxious-Shy, Perfectionism, Social problems, Psychosomatic  Index scales: ADHD Index, Conners’ Global Index: Restless-Impulsive, Conners’ Global Index: Emotional  Lability, Conners’ Global Index: Total  DSM-IV subscales for ADHD | Proxy (parent and teacher forms)  4-point scale (0–3) | DSM-IV |
| *Depression, Anxiety, and Stress Scale (DASS-21;*  Lovibond & Lovibond, 1995) | Age: 14 years – adults  21 items; 3 subscales: Anxiety, Depression, Stress | Self-report  4-point scale (0–3) | Empirically derived |
| *Eyberg Child Behavior Inventory (ECBI;* Eyberg & Pincus, 1999) | Age: 2–16 years  36 items; 2 subscales: Intensity scale, Problem scale | Proxy (parent)  7-point scale (1–7; Intensity scale)  2-point scale (0–1; Problem Scale) | Empirically derived |
| *Hospital Anxiety and Depression Scale (HADS;* Zigmond & Snaith, 1983) | Age: Older youths and adults  14 items; 2 subscales: Anxiety, Depression | Self-report  4-point scale (0–3) | Developed to measure anxiety and depressive symptoms (non-physical symptoms) in a general medical population of patients |
| *Infant Toddler Social Emotional Assessment (ITSEA;* Carter & Briggs-Gowan, 2006*)* | Age: 12–35 months  166 items; 4 subscales: Externalizing, Internalizing, Dysregulation, Competence | Proxy (parent)  3-point scale (0–2) | Literature review. DSM-IV-TR/DC:0–3 |
| *Mini International Neuropsychiatric Interview (MINI;* Sheehan et al. 1998, 2010) | Age: 6–18 years/ > 18 years  Psychiatric diagnoses for major psychiatric disorders. | Diagnostic interview administered to parent or self-report (>18 years) by trained interviewer. | DSM-IV/ICD-10 |
| *Revised Child Anxiety and Depression Scale (RCADS;* Chorpita et al. 2000) | Age: 6–18 years  47 items, 6 subscales: Separation anxiety, GAD, Social anxiety, OCD, Panic disorder, Major depression  Total anxiety (composite score)  Total anxiety and depression (composite score) | Self-report (6–18 years)  Proxy (parent)  4-point scale (0–3) | Modified the Spence Children’s Anxiety Scale to correspond to dimensions of several DSM-IV anxiety disorders, and major depression. |
| *Schedule for Affective Disorders and Schizophrenia*  *for School-Age Children-Present and Lifetime version (KSADS-PL*;  Kaufman et al., 1997) | Age: 6–18 years  Psychiatric diagnoses for major psychiatric disorders. | Diagnostic interview administered to parent or child by trained interviewer. | DSM-IV/DSM-5 |
| *Scales of Independent Behavior–Revised:* Behavior problems items *(SIB-R;* Bruininks et al., 1996) | Age: Children and adults  8 behavior problems items | Proxy (parent)  2-point scale (0–1; frequency)  5-point scale (1–5; severity) | Adaptive behavior |
| *Strengths and Difficulties Questionnaire (SDQ*; Goodman, 1997; Youthinmind, 2013). | Age: 4–18 years and adult version  25 items, 5 scales: Conduct problems, Emotional problems, Hyperactivity/Inattention, Peer relationships, Prosocial behavior.  2 broadband scales: Internalizing and Externalizing.  A total difficulties score.  Extended version includes Impact supplement: 6 items | Youth self-report version (≥ 11 years)  Proxy: Parent and teacher versions.  3-point scale (0–2).  4-point scale (0–3). | Modified the Rutter Questionnaire. Empirically developed. |
| *Social Skills Improvement System-Rating Scales:* Competing problem Behaviors *(SSIS-RS*; Gresham & Elliott, 2008) | Age: Student version 13–18 years)  33 items; Competing problem behavior scale  Externalizing, Bullying, Hyperactivity/Inattention,  Internalizing, Autism Spectrum | Proxy  4-poinst scale (0–3) | Problem behavior subscale: Items constructed from DSM-IV-TR and expert consensus. |

*Note*. Framework = derivation of instrument; GAD = generalized anxiety disorder; IDD = intellectual and developmental disabilities; KSADS = Schedule for Affective Disorders and Schizophrenia for School-aged Children; MH = mental health; OCD = obsessive compulsive disorder; Proxy = completed by informant or observer
