# Supplementary material for: Applicability and Psychometric Properties of General Mental Health Assessment Tools in Autistic People: A Systematic Review
Source: J Autism Dev Disord. 2024 Apr 13;55(5):1713–26. doi: 10.1007/s10803-024-06324-3 (PMC12021962; doi:10.1007/s10803-024-06324-3)
Supplement: Supplementary file 9 — Supplementary file9 (DOCX 126 KB) [file 10803_2024_6324_MOESM9_ESM.docx]

**Appendix I**

*Psychometric Assessment of Instruments*

| Instrument/  study | Internal consistency  Max score: 6 | | | Test-retest  Max score: 6 | | | Inter-rater  Max  score: 6 | | | Criterion validity  Max  score: 6 | | | Content validity  Max score: 3 | Construct validity  Max score: 7 | | | | | Normative data  Max score: 6 | | | Sum score/% | |
| --- | --- | --- | --- | --- | --- | --- | --- | --- | --- | --- | --- | --- | --- | --- | --- | --- | --- | --- | --- | --- | --- | --- | --- |
|  | *N* | | Size coeffic. | *N* | | Size coeffic. | *N* | Size coeffic. | | *N* | | Size coeffic. | Content validity | *N* | | FA | Correlation  similar constructs | | *N* | Quality  info. | |  | |
| *ABC* |  | |  |  | |  |  |  | |  | |  |  |  | |  |  | |  |  | |  | |
| Bakken et al. (2023)^1^ | 2 | | 3 | 0 | | 0 | 0 | 0 | | 2 | | 2 | 0 | 0 | | 0 | 0 | | 0 | 0 | | 9/23% | |
| Bitsika & Sharpley (2017)^2^ | 2 | | 3 | 0 | | 0 | 0 | 0 | | 0 | | 0 | 0 | 0 | | 0 | 0 | | 0 | 0 | | 5/13% | |
| Brinkley et al. (2007) | 0 | | 0 | 0 | | 0 | 0 | 0 | | 0 | | 0 | 0 | 3 | | 1 | 0 | | 0 | 0 | | 4/10% | |
| Chua et al. (2023) | 3 | | 3 | 0 | | 0 | 0 | 0 | | 0 | | 0 | 0 | 0 | | 0 | 0 | | 0 | 0 | | 6/15% | |
| Graziosi & Perry (2023) | 3 | | 3 | 0 | | 0 | 0 | 0 | | 0 | | 0 | 0 | 0 | | 0 | 0 | | 0 | 0 | | 6/15% | |
| Folk & Bal (2019) | 0 | | 0 | 0 | | 0 | 0 | 0 | | 0 | | 0 | 0 | 3 | | 0 | 3 | | 0 | 0 | | 6/15% | |
| Kaat et al. (2014) | 3 | | 3 | 0 | | 0 | 0 | 0 | | 0 | | 0 | 0 | 3 | | 1 | 3 | | 3 | 3 | | 19/48% | |
| Kat et al. (2020) | 3 | | 3 | 1 | | 3 | 1 | 3 | | 0 | | 0 | 0 | 3 | | 1 | 3 | | 0 | 0 | | 21/53% | |
| Koller et al. (2022) | 1 | | 3 | 0 | | 0 | 0 | 0 | | 0 | | 0 | 0 | 0 | | 0 | 0 | | 0 | 0 | | 4/10% | |
| Mohammadi et al. (2023) | 3 | | 3 | 0 | | 0 | 0 | 0 | | 0 | | 0 | 0 | 0 | | 0 | 0 | | 0 | 0 | | 6/15% | |
| Norris et al. (2019) | 3 | | 3 | 0 | | 0 | 0 | 0 | | 0 | | 0 | 0 | 3 | | 1 | 0 | | 3 | 3 | | 16/40% | |
| Rohacek et al. (2023) | 1 | | 3 | 0 | | 0 | 0 | 0 | | 0 | | 0 | 0 | 0 | | 0 | 0 | | 0 | 0 | | 4/10% | |
| Samadi &Rashid (2023) | 2 | | 3 | 0 | | 0 | 0 | 0 | | 0 | | 0 | 0 | 0 | | 0 | 0 | | 0 | 0 | | 5/13% | |
| Taylor et al. (2021) | 3 | | 3 | 0 | | 0 | 0 | 0 | | 0 | | 0 | 0 | 0 | | 0 | 0 | | 0 | 0 | | 6/15% | |
| Yang &Chung (2023) | 1 | | 3 | 0 | | 0 | 0 | 0 | | 0 | | 0 | 0 | 0 | | 0 | 0 | | 0 | 0 | | 4/10% | |
| ABC Total score |  | | |  | | |  | | |  | | |  |  | | | | |  | | | | **121** |
| *ABI* |  | | |  | | |  | | |  | | |  |  | | | | |  | | |  | |
| Bangerter et al. (2017) | 3 | | 1 | 1 | | 2 | 0 | 0 | | 0 | | 0 | 3 | 3 | | 1 | 3 | | 0 | 0 | | 17/43% | |
| ABI Total score |  | | |  | | |  | | |  | | |  |  | | | | |  | | | **17** | |
| *ACB* |  | | |  | | |  | | |  | | |  |  | | | | |  | | |  | |
| Mohammadi et al. (2023) | 3 | 3 | | 0 | 0 | | 0 | | 0 | 0 | 0 | | 0 | 3 | 1 | | | 3 | 0 | 0 | | 13/33% | |
| Palmer et al. (2023) parent | 1 | 3 | | 0 | 0 | | 1 | | 1 | 0 | 0 | | 0 | 0 | 0 | | | 0 | 0 | 0 | | 6/15% | |
| Palmer et al. (2023)  teacher | 1 | 3 | | 0 | 0 | | - | | - | 0 | 0 | | 0 | 0 | 0 | | | 0 | 0 | 0 | | 4/10% | |
| Tarver et al. (2021) | 3 | 3 | | 2 | 3 | | 0 | | 0 | 0 | 0 | | 3 | 3 | 1 | | | 3 | 0 | 0 | | 21/53% | |
| ACB Total score |  | | |  | | |  | | |  | | |  |  | | | | |  | | | **44** | |
| *ACI-PL* |  | | |  | | |  | | |  | | |  |  | | | | |  | | |  | |
| Leyfer et al (2006) | 0 | 0 | | 0 | 0 | | 2 | | 3 | 2 | 3 | | 3 | 2 | 0 | | | 2 | 0 | 0 | | 17/43% | |
| Mazefsky et al. (2012) | 0 | 0 | | 0 | 0 | | 0 | | 0 | 1 | 2 | | 0 | 0 | 0 | | | 0 | 0 | 0 | | 3/8% | |
| ACI-PL Total score |  | | |  | | |  | | |  | | |  |  | | | | |  | | | **20** | |
| *ADIS-IV* |  | | |  | | |  | | |  | | |  |  | | | | |  | | |  | |
| Byrne et al. (2023) | 0 | 0 | | 0 | 0 | | 1 | | 3 | 0 | 0 | | 0 | 0 | 0 | | | 0 | 0 | 0 | | 4/10% | |
| Ung et al. (2014) | 0 | 0 | | 0 | 0 | | 1 | | 3 | 0 | 0 | | 0 | 0 | 0 | | | 0 | 0 | 0 | | 4/10% | |
| ADIS-IV Total score |  | | |  | | |  | | |  | | |  |  | | | | |  | | | **8** | |
| *ASD-CA* |  | | |  | | |  | | |  | | |  |  | | | | |  | | |  | |
| LoVullo & Matson (2009) | 0 | 0 | | 0 | 0 | | 0 | | 0 | 1 | 1 | | 0 | 0 | 0 | | | 0 | 1 | 1 | | 4/10% | |
| Matson & Boisjoli (2008) | 2 | 2 | | 1 | 2 | | 1 | | 2 | 0 | 0 | | 0 | 2 | 1 | | | 0 | 0 | 0 | | 13/33% | |
| SaezSuanes et al. (2020) | 1 | 3 | | 0 | 0 | | 0 | | 0 | 0 | 0 | | 0 | 0 | 0 | | | 0 | 0 | 0 | | 4/10% | |
| ASD-CA Total score |  | | |  | | |  | | |  | | |  |  | | | | |  | | | **21** | |
| *ASD-CC* |  | | |  | | |  | | |  | | |  |  | | | | |  | | |  | |
| Chung & Jung (2017) | 3 | 2 | | 1 | 2 | | 0 | | 0 | 0 | 0 | | 0 | 3 | 1 | | | 1 | 0 | 0 | | 13/33% | |
| Leader, Flynn et al. (2021)^3^ | 0 | 0 | | 0 | 0 | | 0 | | 0 | 0 | 0 | | 0 | 2 | 0 | | | 3 | 0 | 0 | | 5/13% | |
| Leader, Francis et al. (2018) | 2 | 3 | | 0 | 0 | | 0 | | 0 | 0 | 0 | | 0 | 0 | 0 | | | 0 | 0 | 0 | | 5/13% | |
| Mannion & Leader (2013) | 0 | 0 | | 0 | 0 | | 0 | | 0 | 1 | 3 | | 0 | 0 | 0 | | | 0 | 0 | 0 | | 4/10% | |
| Matson, LoVullo et al. (2009) | 2 | 2 | | 0 | 0 | | 0 | | 0 | 0 | 0 | | 0 | 2 | 1 | | | 2 | 0 | 0 | | 9/23% | |
| Matson & Wilkins (2008) | 0 | 0 | | 1 | 1 | | 1 | | 1 | 0 | 0 | | 0 | 0 | 0 | | | 0 | 0 | 0 | | 4/10% | |
| Thorson & Matson (2012) | 0 | 0 | | 0 | 0 | | 0 | | 0 | 0 | 0 | | 0 | 0 | 0 | | | 0 | 3 | 1 | | 4/10% | |
| Tureck et al. (2014) | 0 | 0 | | 0 | 0 | | 0 | | 0 | 1 | 3 | | 0 | 0 | 0 | | | 0 | 0 | 0 | | 4/10% | |
| ASD-CC Total |  | | |  | | |  | | |  | | |  |  | | | | |  | | | **48** | |
| *ASD-PBC* |  | | |  | | |  | | |  | | |  |  | | | | |  | | |  | |
| Mahan & Matson (2011) | 0 | 0 | | 0 | 0 | | 0 | | 0 | 0 | 0 | | 0 | 1 | 0 | | | 2 | 0 | | 0 | 3/8% | |
| ASD-PBC Total |  | | |  | | |  | | |  | | |  |  | | | | |  | | | **3** | |
| *ASEBA* |  | | |  | | |  | | |  | | |  |  | | | | |  | | |  | |
| Bacherini et al. (2021) | 2 | 2 | | 0 | 0 | | 0 | | 0 | 0 | 0 | | 0 | 0 | 0 | | | 0 | 0 | | 0 | 4/10% | |
| Baker & Blacher (2015) | 0 | 0 | | 0 | 0 | | 0 | | 0 | 0 | 0 | | 0 | 1 | 0 | | | 1 | 0 | | 0 | 2/5% | |
| Chan et al. (2022) | 3 | 3 | | 0 | 0 | | 0 | | 0 | 0 | 0 | | 0 | 0 | 0 | | | 0 | 0 | | 0 | 6/15% | |
| Charlton et al. (2020) | 1 | 3 | | 0 | 0 | | 0 | | 0 | 0 | 0 | | 0 | 0 | 0 | | | 0 | 0 | | 0 | 4/10% | |
| Cheng et al. (2022) | 3 | 2 | | 0 | 0 | | 0 | | 0 | 0 | 0 | | 0 | 3 | 1 | | | 0 | 0 | | 0 | 9/23% | |
| Cheng et al. (2021) | - | - | | 0 | 0 | | 0 | | 0 | 0 | 0 | | 0 | 3 | 1 | | | 0 | 0 | | 0 | 4/10% | |
| Clauser et al. (2021) | 1 | 3 | | 0 | 0 | | 0 | | 0 | 0 | 0 | | 0 | 0 | 0 | | | 0 | 0 | | 0 | 4/10% | |
| DeClercq et al. (2021)^4^ | 2 | 3 | | 0 | 0 | | 0 | | 0 | 0 | 0 | | 0 | 0 | 0 | | | 0 | 0 | | 0 | 5/13% | |
| Dovgan et al. (2019): CBCL6–18 | 0 | 0 | | 0 | 0 | | 0 | | 0 | 0 | 0 | | 0 | 2 | 1 | | | 0 | 0 | | 0 | 3/8% | |
| Dovgan et al. (2019): CBCL1.5–5 | 0 | 0 | | 0 | 0 | | 0 | | 0 | 0 | 0 | | 0 | 1 | 1 | | | 0 | 0 | | 0 | 2/5% | |
| Fok & Bal (2019) | 0 | 0 | | 0 | 0 | | 0 | | 0 | 0 | 0 | | 0 | 3 | 0 | | | 3 | 0 | | 0 | 6/15% | |
| Gjevik et al. (2015) | 1 | 2 | | 0 | 0 | | 0 | | 0 | 1 | 1 | | 0 | 0 | 0 | | | 0 | 0 | | 0 | 5/13% | |
| Hurtig et al. (2009) | 0 | 0 | | 0 | 0 | | 1 | | 1 | 0 | 0 | | 0 | 0 | 0 | | | 0 | 0 | | 0 | 2/5% | |
| Jepsen et al. (2012) | 0 | 0 | | 0 | 0 | | 1 | | 1 | 0 | 0 | | 0 | 0 | 0 | | | 0 | 0 | | 0 | 2/5% | |
| Kanne et al. (2009) | 0 | 0 | | 0 | 0 | | 2 | | 1 | 0 | 0 | | 0 | 0 | 0 | | | 0 | 0 | | 0 | 3/8% | |
| Keefer et al. (2020) | 3 | 3 | | 0 | 0 | | 0 | | 0 | 0 | 0 | | 0 | 3 | 1 | | | 0 | 0 | | 0 | 10/25% | |
| La Buissonniere Ariza et al. (2022) | 2 | 3 | | 0 | 0 | | 0 | | 0 | 0 | 0 | | 0 | 0 | 0 | | | 0 | 0 | | 0 | 5/13% | |
| Magyar & Pandolfi (2017) | 0 | 0 | | 0 | 0 | | 0 | | 0 | 1 | 2 | | 0 | 1 | 1 | | | 0 | 0 | | 0 | 5/13% | |
| Manning et al. (2011) | 2 | 3 | | 0 | 0 | | 0 | | 0 | 0 | 0 | | 0 | 0 | 0 | | | 0 | 0 | | 0 | 5/13% | |
| Mazefsky et al. (2014): CBCL | 1 | 3 | | 0 | 0 | | 0 | | 0 | 0 | 0 | | 0 | 1 | 0 | | | 1 | 0 | | 0 | 6/15% | |
| Mazefsky et al. (2014): YSR | 1 | 3 | | 0 | 0 | | 0 | | 0 | 0 | 0 | | 0 | 1 | 0 | | | 1 | 0 | | 0 | 6/15% | |
| Medeiros et al. (2017) | 0 | 0 | | 0 | 0 | | 0 | | 0 | 0 | 0 | | 0 | 3 | 1 | | | 0 | 0 | | 0 | 4/10% | |
| Mello et al. (2022) | 0 | 0 | | 0 | 0 | | 2 | | 1 | 0 | 0 | | 0 | 0 | 0 | | | 0 | 0 | | 0 | 3/8% | |
| Nadeau et al. (2015) | 2 | 3 | | 0 | 0 | | 0 | | 0 | 0 | 0 | | 0 | 0 | 0 | | | 0 | 0 | | 0 | 5/13% | |
| Pandolfi et al. (2012) | 2 | 3 | | 0 | 0 | | 0 | | 0 | 1 | 2 | | 0 | 2 | 1 | | | 0 | 0 | | 0 | 11/28% | |
| Pandolfi et al. (2009) | 2 | 2 | | 0 | 0 | | 0 | | 0 | 0 | 0 | | 0 | 2 | 1 | | | 0 | 0 | | 0 | 7/18% | |
| Pandolfi et al. (2014) | - | - | | 0 | 0 | | 0 | | 0 | 0 | 0 | | 0 | 1 | 0 | | | 2 | 0 | | 0 | 3/8% | |
| Piro-Gambetti et al. (2023) | 3 | 3 | | 0 | 0 | | 0 | | 0 | 0 | 0 | | 0 | 0 | 0 | | | 0 | 0 | | 0 | 6/15% | |
| Pisula et al. (2017) | 0 | 0 | | 0 | 0 | | 1 | | 1 | 0 | 0 | | 0 | 0 | 0 | | | 0 | 0 | | 0 | 2/5% | |
| Rivard et al. (2023) | 3 | 3 | | 0 | 0 | | 0 | | 0 | 0 | 0 | | 0 | 0 | 0 | | | 0 | 0 | | 0 | 6/15% | |
| Rodriguez et al. (2021) | 2 | 3 | | 0 | 0 | | 0 | | 0 | 0 | 0 | | 0 | 0 | 0 | | | 0 | 0 | | 0 | 5/13% | |
| Rodriguez et al. (2019) | 2 | 3 | | 0 | 0 | | 0 | | 0 | 0 | 0 | | 0 | 0 | 0 | | | 0 | 0 | | 0 | 5/13% | |
| Schiltz & Magnus (2020) | 0 | 0 | | 0 | 0 | | 0 | | 0 | 0 | 0 | | 0 | 3 | 1 | | | 0 | 0 | | 0 | 4/10% | |
| Stratis & Lecavalier (2017) | 0 | 0 | | 0 | 0 | | 3 | | 1 | 0 | 0 | | 0 | 0 | 0 | | | 0 | 0 | | 0 | 4/10% | |
| Ung et al. (2017): CBCL1.5–5 | 0 | 0 | | 0 | 0 | | 1 | | 1 | 0 | 0 | | 0 | 0 | 0 | | | 0 | 0 | | 0 | 2/5% | |
| Ung et al. (2017): CBCL6–18 | 0 | 0 | | 0 | 0 | | 1 | | 1 | 0 | 0 | | 0 | 0 | 0 | | | 0 | 0 | | 0 | 2/5% | |
| Xu et al. (2014) | 1 | 3 | | 0 | 0 | | 0 | | 0 | 0 | 0 | | 0 | 0 | 0 | | | 0 | 0 | | 0 | 4/10% | |
| ASEBA Total |  | | |  | | |  | | |  | | |  |  | | | | |  | | | **171** | |
| *BASC-2* |  | | |  | | |  | | |  | | |  |  | | | | |  | | |  | |
| Lane et al. (2013) | 0 | 0 | | 0 | 0 | | 1 | | 1 | 0 | 0 | | 0 | 0 | 0 | | | 0 | 0 | | 0 | 2/5% | |
| Taylor et al. (2020) parent | 0 | 0 | | 0 | 0 | | 1 | | 1 | 0 | 0 | | 0 | 1 | 0 | | | 1 | 0 | | 0 | 4/10% | |
| Taylor et al. (2020) self-report | 0 | 0 | | 0 | 0 | | - | | - | 0 | 0 | | 0 | 1 | 0 | | | 1 | 0 | | 0 | 2/5% | |
| BASC-2 Total |  | | |  | | |  | | |  | | |  |  | | | | |  | | | **8** | |
| *BISCUIT-Part 2* |  | | |  | | |  | | |  | | |  |  | | | | |  | | |  | |
| Horovitz & Matson (2015)^5^ | 0 | 0 | | 0 | 0 | | 0 | | 0 | 0 | 0 | | 0 | 0 | 0 | | | 0 | 3 | | 2 | 5/13% | |
| Matson, Boisjoli et al. (2011) | 3 | 3 | | 0 | 0 | | 0 | | 0 | 0 | 0 | | 0 | 3 | 1 | | | 0 | 0 | | 0 | 10/25% | |
| BISCUIT-Part 2 Total |  | | |  | | |  | | |  | | |  |  | | | | |  | | | **15** | |
| *BISCUIT-Part 3* |  | | |  | | |  | | |  | | |  |  | | | | |  | | |  | |
| Horovitz & Matson (2013) | 0 | 0 | | 0 | 0 | | 0 | | 0 | 0 | 0 | | 0 | 0 | 0 | | | 0 | 2 | | 1 | 3/8% | |
| Matson, Boisjoli et al. (2009) | - | - | | 0 | 0 | | 0 | | 0 | 0 | 0 | | 0 | 3 | 1 | | | 0 | 0 | | 0 | 4/10% | |
| Rojahn, Matson et al. (2009) | 3 | 2 | | 0 | 0 | | 0 | | 0 | 0 | 0 | | 0 | 0 | 0 | | | 0 | - | | - | 5/13% | |
| BISCUIT-Part 3 Total |  | | |  | | |  | | |  | | |  |  | | | | |  | | | **12** | |
| *BPI* |  | | |  | | |  | | |  | | |  |  | | | | |  | | |  | |
| Leader, Dooley et al. (2021)^6^ | 2 | 3 | | 0 | 0 | | 0 | | 0 | 0 | 0 | | 0 | 0 | 0 | | | 0 | 0 | | 0 | 5/13% | |
| Leader, Flynn et al. (2021) | 0 | 0 | | 0 | 0 | | 0 | | 0 | 0 | 0 | | 0 | 2 | 0 | | | 3 | 0 | | 0 | 5/13% | |
| Pozo & Sarria (2014) | 1 | 3 | | 0 | 0 | | 0 | | 0 | 0 | 0 | | 0 | 0 | 0 | | | 0 | 0 | | 0 | 4/10% | |
| Wei et al. (2023) | 3 | 3 | | 0 | 0 | | 0 | | 0 | 0 | 0 | | 0 | 0 | 0 | | | 0 | 0 | | 0 | 6/15% | |
| BPI Total |  | | |  | | |  | | |  | | |  |  | | | | |  | | | **20** | |
| *CASI* |  | | |  | | |  | | |  | | |  |  | | | | |  | | |  | |
| Bitsika, Sharpley et al. (2016) parent | 2 | 3 | | 0 | 0 | | 2 | | 2 | 0 | 0 | | 0 | 2 | 1 | | | 0 | 0 | | 0 | 12/30% | |
| Bitsika, Sharpley et al. (2016) self-report | 2 | 2 | | 0 | 0 | | - | | - | 0 | 0 | | 0 | 2 | 1 | | | 0 | 0 | | 0 | 7/18% | |
| Kaat et al. (2013) | 0 | 0 | | 0 | 0 | | 1 | | 1 | 0 |  | | 0 | 0 | 0 | | | 0 | 0 | | 0 | 2/5% | |
| Lecavalier et al. (2009) 6–12 yrs parent | 0 | 0 | | 0 | 0 | | 0 | | 0 | 0 | 0 | | 0 | 3 | 1 | | | 0 | 0 | | 0 | 4/10% | |
| Lecavalier et al. (2009) 6–12 yrs teacher | 0 | 0 | | 0 | 0 | | 0 | | 0 | 0 | 0 | | 0 | 3 | 1 | | | 0 | 0 | | 0 | 4/10% | |
| Lecavalier et al. (2011) 3–5 yrs parent | 0 | 0 | | 0 | 0 | | 2 | | 1 | 0 | 0 | | 0 | 3 | 1 | | | 0 | 0 | | 0 | 7/18% | |
| Lecavalier et al. (2011) 3–5 yrs teacher | 0 | 0 | | 0 | 0 | | - | | - | 0 | 0 | | 0 | 2 | 1 | | | 0 | 0 | | 0 | 3/8% | |
| CASI/CSI Total |  | | |  | | |  | | |  | | |  |  | | | | |  | | | **39** | |
| *chIPS* |  | | |  | | |  | | |  | | |  |  | | | | |  | | |  | |
| Witwer et al. (2012) | 1 | 2 | | 0 | 0 | | 1 | | 2 | 0 | 0 | | 0 | 1 | 0 | | | 2 | 0 | | 0 | 9/23% | |
| ChIPS Total |  | | |  | | |  | | |  | | |  |  | | | | |  | | | **9** | |
| *C-SHARP* |  | | |  | | |  | | |  | | |  |  | | | | |  | | |  | |
| Farmer et al. (2016) | 3 | 3 | | 0 | 0 | | 0 | | 0 | 0 | 0 | | 0 | 3 | 1 | | | 0 | 0 | | 0 | 10/25% | |
| Kirst et al. (2022) | 1 | 2 | | 0 | 0 | | 0 | | 0 | 0 | 0 | | 0 | 0 | 0 | | | 0 | 0 | | 0 | 3/8% | |
| C-SHARP Total |  | | |  | | |  | | |  | | |  |  | | | | |  | | | **13** | |
| *Conners (CRS-R)* |  | | |  | | |  | | |  | | |  |  | | | | |  | | |  | |
| Pearson et al. (2012) | 0 | 0 | | 0 | 0 | | 1 | | 1 | 0 | 0 | | 0 | 0 | 0 | | | 0 | 0 | | 0 | 2/5% | |
| CRS-R Total |  |  | |  |  | |  | |  |  |  | |  |  |  | | |  |  | |  | **2** | |
| *DASS-21* |  |  | |  |  | |  | |  |  |  | |  |  |  | | |  |  | |  |  | |
| Park et al. (2020) | 2 | 3 | | 0 | 0 | | 0 | | 0 | 0 | 0 | | 0 | 2 | 1 | | | 3 | 0 | | 0 | 11/28% | |
| DASS-21 Total |  |  | |  |  | |  | |  |  |  | |  |  |  | | |  |  | |  | **11** | |
| *DBC* |  |  | |  |  | |  | |  |  |  | |  |  |  | | |  |  | |  |  | |
| Adams et al. (2019) | 2 | 3 | | 0 | 0 | | 0 | | 0 | 0 | 0 | | 0 | 0 | 0 | | | 0 | 0 | | 0 | 5/13% | |
| Chandler et al. (2016) | 3 | 3 | | 0 | 0 | | 3 | | 1 | 0 | 0 | | 0 | 0 | 0 | | | 0 | 0 | | 0 | 10/25% | |
| Hastings et al. (2005) | 0 | 0 | | 0 | 0 | | 1 | | 3 | 0 | 0 | | 0 | 0 | 0 | | | 0 | 0 | | 0 | 4/10% | |
| Jellett et al. (2015) | 1 | 3 | | 0 | 0 | | 0 | | 0 | 0 | 0 | | 0 | 0 | 0 | | | 0 | 0 | | 0 | 4/10% | |
| Khor et al. (2014) | 1 | 3 | | 1 | 3 | | 0 | | 0 | 0 | 0 | | 0 | 0 | 0 | | | 0 | 0 | | 0 | 8/20% | |
| Magiati et al. (2016) | 3 | 3 | | 0 | 0 | | 0 | | 0 | 0 | 0 | | 0 | 3 | 0 | | | 2 | 0 | | 0 | 11/28% | |
| DBC Total |  |  | |  |  | |  | |  |  |  | |  |  |  | | |  |  | |  | **42** | |
| *ECBI* |  |  | |  |  | |  | |  |  |  | |  |  |  | | |  |  | |  |  | |
| Brookman-Frazee et al. (2018) | 3 | 3 | | 0 | 0 | | 0 | | 0 | 0 | 0 | | 0 | 3 | 0 | | | 2 | 0 | | 0 | 11/28% | |
| Jeter et al. (2017) | 3 | 3 | | 0 | 0 | | 0 | | 0 | 0 | 0 | | 0 | 3 | 1 | | | 2 | 0 | | 0 | 12/30% | |
| Martinez et al. (2023) | - | - | | 0 | 0 | | 0 | | 0 | 0 | 0 | | 0 | 3 | 1 | | | - | 0 | | 0 | 4/10% | |
| ECBI Total |  |  | |  |  | |  | |  |  |  | |  |  |  | | |  |  | |  | **27** | |
| *EDI* |  |  | |  |  | |  | |  |  |  | |  |  |  | | |  |  | |  |  | |
| Day et al. (2024) | 0 | 0 | | 0 | 0 | | 0 | | 0 | 0 | 0 | | 0 | 3 | 1 | | | 2 | 0 | | 0 | 6/15% | |
| Mazefsky, Day et al. (2018) | 0 | 0 | | 0 | 0 | | 0 | | 0 | 0 | 0 | | 3 | 0 | 0 | | | 0 | 0 | | 0 | 3/8% | |
| Mazefsky, Yu et al. (2018) | 0 | 0 | | 3 | 2 | | 0 | | 0 | 3 | 3 | | 0 | 3 | 1 | | | 3 | 0 | | 0 | 18/45% | |
| Riek et al. (2023) | 1 | 3 | | 0 | 0 | | 0 | | 0 | 0 | 0 | | 0 | 1 | 0 | | | 2 | 0 | | 0 | 7/18% | |
| Skwerer et al. (2019) | 0 | 0 | | 0 | 0 | | 0 | | 0 | 0 | 0 | | 0 | 1 | 0 | | | 1 | 0 | | 0 | 2/5% | |
| Taylor et al. (2021) | 3 | 3 | | 0 | 0 | | 0 | | 0 | 0 | 0 | | 0 | 0 | 0 | | | 0 | 0 | | 0 | 6/15% | |
| EDI Total  *HADS* |  |  | |  |  | |  | |  |  |  | |  |  |  | | |  |  | |  | **42** | |
| Uljarevic et al. (2018) | 2 | 3 | | 0 | 0 | | 0 | | 0 | 0 | 0 | | 0 | 2 | 1 | | | 2 | 0 | | 0 | 10/25% | |
| HADS Total |  |  | |  |  | |  | |  |  |  | |  |  |  | | |  |  | |  | **10** | |
| *ITSEA* |  |  | |  |  | |  | |  |  |  | |  |  |  | | |  |  | |  |  | |
| Davis & Carter (2008) | 0 | 0 | | 0 | 0 | | 1 | | 2 | 0 | 0 | | 0 | 0 | 0 | | | 0 | 0 | | 0 | 3/8% | |
| ITSEA Total |  |  | |  |  | |  | |  |  |  | |  |  |  | | |  |  | |  | **3** | |
| *K-CSCB* |  |  | |  |  | |  | |  |  |  | |  |  |  | | |  |  | |  |  | |
| Kim et al. (2018) | 2 | 3 | | 1 | 1 | | 0 | | 0 | 0 | 0 | | 0 | 2 | 0 | | | 3 | 0 | | 0 | 12/30% | |
| K-CSCB Total |  |  | |  |  | |  | |  |  |  | |  |  |  | | |  |  | |  | **12** | |
| *K-SADS* |  |  | |  |  | |  | |  |  |  | |  |  |  | | |  |  | |  |  | |
| Gjevik et al. (2011) | 0 | 0 | | 0 | 0 | | 1 | | 3 | 0 | 0 | | 0 | 0 | 0 | | | 0 | 0 | | 0 | 4/10% | |
| Hepburn et al. (2014) | 0 | 0 | | 0 | 0 | | 1 | | 3 | 0 | 0 | | 0 | 0 | 0 | | | 0 | 0 | | 0 | 4/10% | |
| Mattila et al. (2010) | 0 | 0 | | 0 | 0 | | 1 | | 3 | 0 | 0 | | 0 | 0 | 0 | | | 0 | 0 | | 0 | 4/10% | |
| K-SADS Total |  |  | |  |  | |  | |  |  |  | |  |  |  | | |  |  | |  | **12** | |
| *MCAS* |  |  | |  |  | |  | |  |  |  | |  |  |  | | |  |  | |  |  | |
| Kalb et al. (2018) | 3 | 3 | | 0 | 0 | | 0 | | 0 | 3 | 3 | | 3 | 3 | 1 | | | 3 | 0 | | 0 | 22/55% | |
| MCAS Total |  |  | |  |  | |  | |  |  |  | |  |  |  | | |  |  | |  |  | |
| *MINI* |  |  | |  |  | |  | |  |  |  | |  |  |  | | |  |  | |  | **22** | |
| Brookman-Frazee et al. (2018) | 0 | 0 | | 0 | 0 | | - | | - | 0 | 0 | | 0 | 2 | 0 | | | 2 | 0 | | 0 | 4/10% | |
| Mosner et al. (2019) | 0 | 0 | | 0 | 0 | | 0 | | 0 | 1 | 2 | | 0 | 0 | 0 | | | 0 | 0 | | 0 | 3/8% | |
| Stadnick et al. (2017) | 0 | 0 | | 0 | 0 | | 1 | | 3 | 2 | 1 | | 0 | 0 | 0 | | | 0 | 0 | | 0 | 7/18% | |
| MINI Total |  |  | |  |  | |  | |  |  |  | |  |  |  | | |  |  | |  | **14** | |
| *MINI-PAS-ADD* |  |  | |  |  | |  | |  |  |  | |  |  |  | | |  |  | |  |  | |
| Buck et al. (2014) | 0 | 0 | | 0 | 0 | | 0 | | 0 | 2 | 2 | | 0 | 0 | 0 | | | 0 | 0 | | 0 | 4/10% | |
| MINI-PASS-ADD Total |  |  | |  |  | |  | |  |  |  | |  |  |  | | |  |  | |  | **4** | |
| *Nisonger* |  |  | |  |  | |  | |  |  |  | |  |  |  | | |  |  | |  |  | |
| Beer et al. (2013) | 1 | 3 | | 0 | 0 | | 0 | | 0 | 0 | 0 | | 0 | 0 | 0 | | | 0 | 0 | | 0 | 4/10% | |
| Bekhet (2016) | 2 | 3 | | 0 | 0 | | 0 | | 0 | 0 | 0 | | 0 | 0 | 0 | | | 0 | 0 | | 0 | 5/13% | |
| Benson (2015) | 2 | 3 | | 0 | 0 | | 0 | | 0 | 0 | 0 | | 0 | 0 | 0 | | | 0 | 0 | | 0 | 5/13% | |
| Gardiner & Iarocci (2015) | 1 | 3 | | 0 | 0 | | 0 | | 0 | 0 | 0 | | 0 | 0 | 0 | | | 0 | 0 | | 0 | 4/10% | |
| Firth & Dryer (2013) | 2 | 3 | | 0 | 0 | | 0 | | 0 | 0 | 0 | | 0 | 0 | 0 | | | 0 | 0 | | 0 | 5/13% | |
| Fong et al. (2020)^7^ | 2 | 3 | | 0 | 0 | | 0 | | 0 | 0 | 0 | | 0 | 0 | 0 | | | 0 | 0 | | 0 | 5/13% | |
| Lecavalier et al. (2004) parent | 3 | 3 | | 0 | 0 | | 0 | | 0 | 0 | 0 | | 0 | 3 | 1 | | | 0 | 0 | | 0 | 10/25% | |
| Lecavalier et al. (2004) teacher | 3 | 3 | | 0 | 0 | | 0 | | 0 | 0 | 0 | | 0 | 3 | 1 | | | 0 | 0 | | 0 | 10/25% | |
| Lecavalier et al. (2006) | 0 | 0 | | 0 | 0 | | 2 | | 1 | 0 | 0 | | 0 | 0 | 0 | | | 0 | 0 | | 0 | 3/8% | |
| Weiss et al. (2012) | 3 | 3 | | 0 | 0 | | 0 | | 0 | 0 | 0 | | 0 | 0 | 0 | | | 0 | 0 | | 0 | 6/15% | |
| Nisonger Total |  |  | |  |  | |  | |  |  |  | |  |  |  | | |  |  | |  | **57** | |
| *OSCA-ABP* |  |  | |  |  | |  | |  |  |  | |  |  |  | | |  |  | |  |  | |
| Palmer et al. (2021)^8^ | 0 | 0 | | 0 | 0 | | 1 | | 3 | 0 | 0 | | 3 | 1 | 0 | | | 1 | 0 | | 0 | 9/23% | |
| OSCA-ABP Total |  |  | |  |  | |  | |  |  |  | |  |  |  | | |  |  | |  | **9** | |
| *PAC* |  |  | |  |  | |  | |  |  |  | |  |  |  | | |  |  | |  |  | |
| Bakken et al. (2010) | 0 | 0 | | 0 | 0 | | 1 | | 2 | 0 | 0 | | 0 | 0 | 0 | | | 0 | 0 | | 0 | 3/8% | |
| Bakken et al. (2023)^1^ | 2 | 2 | | 0 | 0 | | 0 | | 0 | 2 | 2 | | 0 | 0 | 0 | | | 0 | 0 | | 0 | 8/20% | |
| Helverschou et al. (2009) | 1 | 3 | | 0 | 0 | | 1 | | 2 | 1 | 2 | | 0 | 0 | 0 | | | 0 | 0 | | 0 | 10/25% | |
| Helverschou et al. (2021) | 0 | 0 | | 0 | 0 | | 0 | | 0 | 2 | 2 | | 0 | 2 | 0 | | | 1 | 0 | | 0 | 7/18% | |
| PAC Total |  |  | |  |  | |  | |  |  |  | |  |  |  | | |  |  | |  | **28** | |
| *RCADS* |  |  | |  |  | |  | |  |  |  | |  |  |  | | |  |  | |  |  | |
| Kaat et al. (2015) parent | 1 | 3 | | 1 | 3 | | 1 | | 1 | 0 | 0 | | 0 | 1 | 0 | | | 2 | 0 | | 0 | 13/33% | |
| Kaat et al. (2015) youth | 1 | 2 | | 0 | 0 | | - | | - | 0 | 0 | | 0 | 1 | 0 | | | 1 | 0 | | 0 | 5/13% | |
| Khalfe et al. (2023) parent | 1 | 2 | | 0 | 0 | | 1 | | 1 | 0 | 0 | | 0 | 1 | 0 | | | 2 | 0 | | 0 | 8/20% | |
| Khalfe et al. (2023)  self-report | 1 | 3 | | 0 | 0 | | - | | - | 0 | 0 | | 0 | 1 | 0 | | | 1 | 0 | | 0 | 6/15% | |
| Steerling et al. (2015) | 1 | 2 | | 0 | 0 | | 0 | | 0 | 0 | 0 | | 0 | 1 | 0 | | | 1 | 0 | | 0 | 5/13% | |
| RCADS Total |  |  | |  |  | |  | |  |  |  | |  |  |  | | |  |  | |  | **37** | |
| *SDQ* |  |  | |  |  | |  | |  |  |  | |  |  |  | | |  |  | |  |  | |
| Alallawi et al. (2022) | 2 | 2 | | 0 | 0 | | 0 | | 0 | 0 | 0 | | 0 | 0 | 0 | | | 0 | 0 | | 0 | 4/10% | |
| Burton et al. (2020) parent | 3 | 1 | | 0 | 0 | | 0 | | 0 | 0 | 0 | | 0 | 0 | 0 | | | 0 | 0 | | 0 | 4/10% | |
| Burton et al. (2020) teacher | 3 | 2 | | 0 | 0 | | 0 | | 0 | 0 | 0 | | 0 | 0 | 0 | | | 0 | 0 | | 0 | 5/13% | |
| Deniz & Toseeb (2023) | 3 | 3 | | 0 | 0 | | 0 | | 0 | 0 | 0 | | 0 | 0 | 0 | | | 0 | 0 | | 0 | 6/15% | |
| Findon et al. (2016) parent | 2 | 2 | | 0 | 0 | | 1 | | 1 | 2 | 1 | | 0 | 2 | 0 | | | 2 | 0 | | 0 | 13/33% | |
| Findon et al. (2016) self-report | 1 | 1 | | 0 | 0 | | - | | - | 1 | 1 | | 0 | 1 | 0 | | | 1 | 0 | | 0 | 6/15% | |
| Hastings et al. (2022) | 2 | 2 | | 0 | 0 | | 0 | | 0 | 0 | 0 | | 0 | 0 | 0 | | | 0 | 0 | | 0 | 4/10% | |
| Jones et al. (2014) | 1 | 1 | | 0 | 0 | | 0 | | 0 | 0 | 0 | | 0 | 0 | 0 | | | 0 | 0 | | 0 | 2/5% | |
| Kang et al. (2020) | 2 | 1 | | 0 | 0 | | 0 | | 0 | 0 | 0 | | 0 | 0 | 0 | | | 0 | 0 | | 0 | 3/8% | |
| Khor et al. (2014) | 1 | 3 | | 0 | 0 | | 0 | | 0 | 0 | 0 | | 0 | 0 | 0 | | | 0 | 0 | | 0 | 4/10% | |
| Lovell et al. (2016)^9^ | 2 | 1 | | 0 | 0 | | 0 | | 0 | 0 | 0 | | 0 | 0 | 0 | | | 0 | 0 | | 0 | 3/8% | |
| Lu, Wang et al. (2021)^10^ | 3 | 2 | | 0 | 0 | | 0 | | 0 | 0 | 0 | | 0 | 0 | 0 | | | 0 | 0 | | 0 | 5/13% | |
| McIntyre et al. (2023) | 1 | 1 | | 0 | 0 | | 0 | | 0 | 0 | 0 | | 0 | 0 | 0 | | | 0 | 0 | | 0 | 2/5% | |
| Milosavljevic et al. (2016) | 1 | 1 | | 0 | 0 | | 0 | | 0 | 0 | 0 | | 0 | 0 | 0 | | | 0 | 0 | | 0 | 2/5% | |
| Miranda et al. (2019) | 1 | 3 | | 0 | 0 | | 0 | | 0 | 0 | 0 | | 0 | 0 | 0 | | | 0 | 0 | | 0 | 4/10% | |
| Plak et al. (2023) | 3 | 3 | | 0 | 0 | | 0 | | 0 | 0 | 0 | | 0 | 0 | 0 | | | 0 | 0 | | 0 | 6/15% | |
| Pruitt et al. (2018) | 1 | 3 | | 0 | 0 | | 0 | | 0 | 0 | 0 | | 0 | 0 | 0 | | | 0 | 0 | | 0 | 4/10% | |
| Reyes et al. (2020) | 1 | 2 | | 0 | 0 | | 0 | | 0 | 0 | 0 | | 0 | 0 | 0 | | | 0 | 0 | | 0 | 3/8% | |
| Rixon et al. (2021) | 2 | 2 | | 0 | 0 | | 0 | | 0 | 0 | 0 | | 0 | 0 | 0 | | | 0 | 0 | | 0 | 4/10% | |
| Salomone et al. (2014) parent | 3 | 1 | | 0 | 0 | | 3 | | 1 | 0 | 0 | | 0 | 0 | 0 | | | 0 | 0 | | 0 | 8/20% | |
| Salomone et al. (2014) teacher | 3 | 1 | | 0 | 0 | | - | | - | 0 | 0 | | 0 | 0 | 0 | | | 0 | 0 | | 0 | 4/10% | |
| Salomone et al. (2019) | 1 | 2 | | 0 | 0 | | 0 | | 0 | 0 | 0 | | 0 | 0 | 0 | | | 0 | 0 | | 0 | 3/8% | |
| Totsika et al. (2013) | 2 | 3 | | 0 | 0 | | 0 | | 0 | 0 | 0 | | 0 | 0 | 0 | | | 0 | 0 | | 0 | 5/13% | |
| Wang et al. (2016) | 1 | 1 | | 0 | 0 | | 0 | | 0 | 0 | 0 | | 0 | 0 | 0 | | | 0 | 0 | | 0 | 2/5% | |
| Werkman et al. (2020) | 3 | 2 | | 0 | 0 | | 0 | | 0 | 0 | 0 | | 0 | 0 | 0 | | | 0 | 0 | | 0 | 5/13% | |
| Yan et al. (2023) | 3 | 3 | | 0 | 0 | | 0 | | 0 | 0 | 0 | | 0 | 0 | 0 | | | 0 | 0 | | 0 | 6/15% | |
| Yang et al. (2023) | 3 | 2 | | 0 | 0 | | 0 | | 0 | 0 | 0 | | 0 | 0 | 0 | | | 0 | 0 | | 0 | 5/13% | |
| SDQ Total |  |  | |  |  | |  | |  |  |  | |  |  |  | | |  |  | |  | **122** | |
| *SIB-R Behavior Problem scale* |  |  | |  |  | |  | |  |  |  | |  |  |  | | |  |  | |  |  | |
| Mihaila & Hartley et al. (2018) | 2 | 3 | | 0 | 0 | | 2 | | 2 | 0 | 0 | | 0 | 2 | 0 | | | 1 | 0 | | 0 | 12/30% | |
| SIB-R Total |  |  | |  |  | |  | |  |  |  | |  |  |  | | |  |  | |  | **12** | |
| *SSIS-RS Behavior problem scale* |  |  | |  |  | |  | |  |  |  | |  |  |  | | |  |  | |  |  | |
| Schiltz et al. (2018) | 1 | 3 | | 0 | 0 | | 0 | | 0 | 0 | 0 | | 0 | 0 | 0 | | | 0 | 0 | | 0 | 4/10% | |
| SSIS-RS Total |  |  | |  |  | |  | |  |  |  | |  |  |  | | |  |  | |  | **4** | |

*Note*. Scores given for each psychometric property were based on criteria developed by the European Federation of Psychologists’ Association (2013). For each psychometric property including the sum, higher scores indicate better methodological quality of instruments.^1^Kildahl & Helverschou (2023) and Kildahl, Ludvigsen et al. (2023) report overlapping data from the same study;^2^Bitsika & Sharpley (2016) = report overlapping data from the same study; ^3^Leader et al. (2022) report overlapping data from the same study; ^4^DeClerq et al. (2019), DePauw et al. (2021), Dieleman et al. (2017, 2018) = report overlapping data from the same study.^5^Matson, Fodstad et al (2009) report overlapping data from the same study;

^6^Higgins et al. (2022) and Leader et al. (2022) report overlapping data from same study;^7^Fong et al. (2021) report overlapping data from the same study;^8^Palmer et al. (2023) report overlapping data from the same study; ^9^Lovell et al. (2020) report overlapping data from the same study; ^10^Lu, Chen et al. (2021) report overlapping data from the same study; (-) = already evaluated in paper from same study.

ABC = Aberrant Behavior Checklist; ABI = Autism Behavior Inventory; ACB = Assessment of Concerning Behavior Scale; ACI-PL = Autism Comorbidity Interview; ADIS-IV = Anxiety Disorders Interview Schedule for DSM-IV; ASD-CA = Autism Spectrum Disorders Comorbidity for Adults; ASD-CC = Autism Spectrum Comorbid for Children; ASD-PBC = Autism Spectrum Disorder Problem Behavior Child version; ASEBA = Achenbach System of Empirically Based Assessment; BASC-2= Behavioral Assessment System for Children; BISCUIT-Part 2 = Baby and Infant Screen for Children with Autism Traits–Part 2 Comorbid Psychopathology; BISCUIT-Part 3 = Challenging Behavior; BPI = Behavior Problems Inventory; CASI = Child and Adolescent Symptom Inventory; ChIPS = Children’s Interview for Psychiatric Syndromes; C-SHARP = Children’s Scale of Hostility and Aggression; Conners CRS-R = Conners Rating Scale Revised; DASS-21 = Depression, Anxiety, and Stress Scale; DBC = Developmental Behavior Checklist; ECBI = Eyberg Child Behavior Inventory; FA = factor analysis; HADS = Hospital Anxiety and Depression Scale; ITSEA= Infant Toddler Social Emotional Assessment; K-CSCB = Korean Comprehensive Scale for the Assessment of Challenging Behavior in Developmental Disorder; KSADS = Schedule for Affective Disorders and Schizophrenia for School-Age Children; MCAS = Mental Health Crisis Assessment Scale; MINI = Mini International Neuropsychiatric Interview; Nisonger = Nisonger Child Behavior Rating Form; OSCA-ABP = Observation Schedule for Children with Autism; PAC = Psychopathology in Autism Checklist; RCADS = Revised Child Anxiety and Depression Scale; SDQ = Strengths and Difficulties Questionnaire; SIB-R = Scales of Independent Behavior–Revised; SSIS-RS = Social Skills Improvement System-Rating Scales
